# Cascade transformation of 2-(diazoacetyl)-2*H*-azirines to 2-aroyl-3-hydroxy-1*H*-pyrroles via condensation with aromatic aldehydes

**DOI:** 10.3762/bjoc.22.70

**Published:** 2026-06-09

**Authors:** Timur O Zanakhov, Ekaterina E Galenko, Mikhail S Novikov, Alexander F Khlebnikov

**Affiliations:** 1 Saint Petersburg State University, Institute of Chemistry, 7/9 Universitetskaya Naberezhnaya, St. Petersburg 199034, Russia

**Keywords:** azirines, condensation, cyclization, diazo compounds, pyrroles

## Abstract

The Cs_2_CO_3_-induced condensation of 2-(diazoacetyl)-2*H*-azirines with aromatic aldehydes does not result in the formation of an azirinyl-substituted β-hydroxy-α-diazocarbonyl compound, but is accompanied by a tandem intramolecular cyclization involving the hydroxy group and the C=N bond of the azirine to form a bicyclic intermediate, a 4-diazo-2-oxa-7-azabicyclo[4.1.0]heptan-5-one derivative. The acid-catalyzed transformation of which leads to 2-aroyl-3-hydroxy-1*H*-pyrroles.

## Introduction

**Scheme 1:**
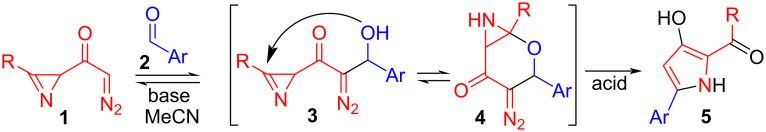
Reaction of 2-(diazoacetyl)-2*H*-azirines **1** with aldehydes **2**.

Diazo compounds play a significant role in the synthesis of heterocyclic compounds, which explains the ever-growing structural diversity of such molecules and the variety of their transformation pathways [1–10]. Azirinyl-substituted diazo compounds, which we have recently introduced into the heterocyclic synthesis arsenal [11–19], are essentially binary synthetic building blocks consisting of a highly strained small ring and an active functional group that are capable of reacting in domino or orthogonal modes. This dual reactivity allows their use in the syntheses of monocyclic heterocycles [11–14], fused heterocycles [15–18], and heterocyclic hybrids [11,19]. However, the diversity of known azirinyl-substituted diazo compounds is currently limited to diazo ketones [11–13,15], 2-(diazoacetyl)-2*H*-azirines, α-diazo-β-ketoesters [14,16–19], and alkyl 2-diazo-3-oxo-3-(2*H*-azirin-2-yl)propanoates. Considering that the condensation of acyldiazomethanes to aldehydes leads to the β-hydroxy-α-diazo carbonyl compound [20–22], we hypothesized that such condensation of aldehydes with 2-(diazoacetyl)-2*H*-azirines could yield the corresponding azirinyl-substituted diazo compounds. This paper describes the reaction of 2-(diazoacetyl)-2*H*-azirines **1** with aromatic aldehydes **2**, which does not terminate in condensation to form an azirinyl-substituted β-hydroxy-α-diazocarbonyl compound **3**, but is accompanied by a tandem intramolecular cyclization involving the hydroxy group and the azirine C=N bond to form a bicyclic intermediate **4**. This acid-catalyzed transformation leads to 3-hydroxy-2-aroyl-1*H*-pyrroles **5** (Scheme 1). To the best of our knowledge, only few examples of such compounds have been so far reported [23–24].

## Results and Discussion

**Scheme 2:**
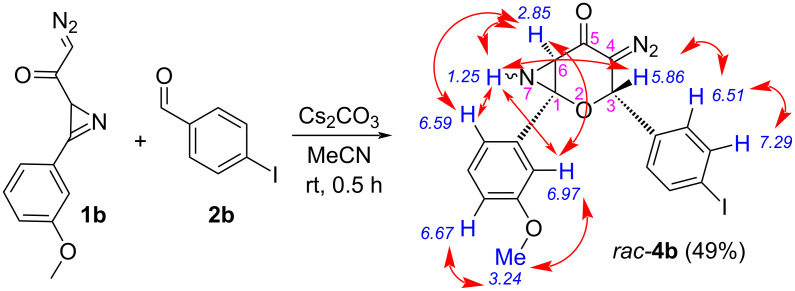
Reaction of 2-(diazoacetyl)-2*H*-azirine **1b** with aldehyde **2b** and the result of 2D NOESY experiment for **4b**.

A test reaction of 2-(2-diazoacetyl)-3-phenyl-2*H*-azirine (**1a**, R = Ph) with 4-fluorobenzaldehyde (**2a**, Ar = 4-FC_6_H_4_) was carried out in MeCN in the presence of various bases. When using amine-type bases soluble in acetonitrile, such as Et_3_N and DMAP, the reaction did not occur (the outcome of the reaction was monitored by TLC), whereas the use of TMG or DBU resulted in resinification of the reaction mixture. The use of K_2_CO_3_ and Cs_2_CO_3_, which are poorly soluble in acetonitrile, gave different results. In the presence of K_2_CO_3_ (suspension in acetonitrile), the reaction did not occur, whereas the outcome of the reaction with a suspension of Cs_2_CO_3_ (suspension in acetonitrile) in MeCN depended on the amount of Cs_2_CO_3_ used: In the presence of 1–2 equivalents, resinification of the reaction mixture occurred, whereas in the presence of 0.5 equivalents, a complete consumption of the starting compounds could be observed, and a product was formed. However, the resulting product (probably **4a**; Scheme 1, R = Ph, Ar = 4-FC_6_H_4_) turned out to be extremely unstable and could not be isolated either by crystallization or by chromatography on silica gel or alumina, which led to its complete decomposition. Fortunately, the corresponding product of the reaction of 2-(2-diazoacetyl)-3-(3-methoxyphenyl)-2*H*-azirine **1b** with 4-iodobenzaldehyde (**2b**) turned out to be well crystallized and precipitated from the reaction medium as a solid (Scheme 2). Although it was also unstable, it was possible to record ^1^H,^1^H 2D NOESY and ^13^C NMR spectra, an IR spectrum in KBr as well as to obtain the accurate mass of the molecular ion by HRMS for this crude product. The IR spectrum of the compound contains a band of the diazo group at 2087 cm^−1^. According to the ^1^H NMR spectrum, the product consists of more than 90% of one diastereoisomer, the spatial structure of which was established using a 2D NOESY experiment (see Supporting Information File 1). The entire set of spectral data allows us to assign the structure (1*RR*,3*SR*,6*RR*)-4-diazo-3-(4-iodophenyl)-1-(3-methoxyphenyl)-2-oxa-7-azabicyclo[4.1.0]heptan-5-one (**4b**) to the main product (Scheme 2). The presence of cross peaks of the HN proton with both the *ortho*-protons of the 3-MeOC_6_H_4_ group and the 3-H, 6-H protons is explained by the rapid inversion of the aziridine nitrogen. According to calculations, the inversion barrier of the aziridine nitrogen in the phenyl analogue of compound **4b** does not exceed 11.5 kcal/mol (see Figure S2 in the Supporting Information File 1).

In the ^1^H NMR spectrum of the crude compound **4b**, in addition to the signals of the main isomer **4b**, there is a set of low-intensity proton signals: 1.34 (d), 2.59 (d), 3.27 (s), 5.16 (s), formally corresponding to the set of signals of the main diastereoisomer **4b**: (1.26 (d), 2.85 (d), 3.24 (s), 5.66 (s)), which may indicate the presence of an impurity of the second, less stable diastereoisomer (5–10%).

**Scheme 3:**
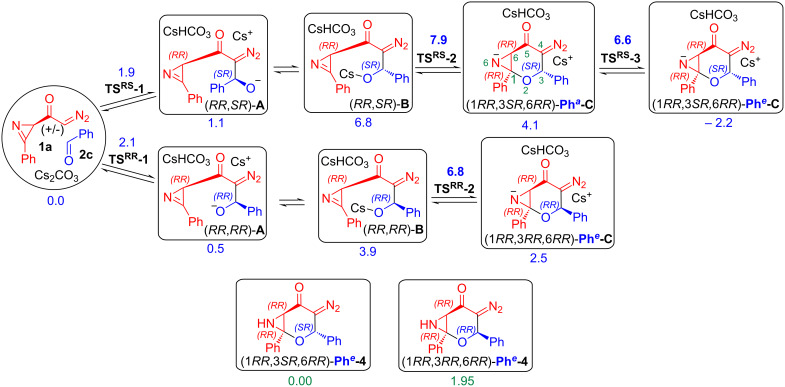
Relative Gibbs free energies for the condensation of diazoacetylazirine **1a** and benzaldehyde (**2c**) in the presence of Cs_2_CO_3_ (in kcal/mol, 298 K, DFT B3LYP-D3/6-311+G(d,p)/ LANL2DZ(Cs) level of theory with a SMD solvent model for MeCN).

The obtained results suggest that the primary product, the Cs-enolate of aldol **3**, cyclizes under the reaction conditions to form an unstable bicyclic product **4**, which undergoes uncontrolled decomposition upon chromatographic isolation. Although theoretically, the reaction of azirine **1** and aldehyde **2** can form four racemic diastereoisomers, in reality in the case of the bicyclic (3,6) system 2-oxa-7-azabicyclo[4.1.0]heptane, only two racemic diastereoisomers can form due to the impossibility of *trans*-fusion. The *trans* configuration forces the bridgehead substituents onto opposite sides of the rings. This rigid geometric requirement severely distorts the sp³ bond angles at the fusion carbons, generating immense, destabilizing angle and torsional strain across the fused system. In fact, to our knowledge, no derivative of *trans*-2-oxa-7-azabicyclo[4.1.0]heptane has been prepared [25–26]. According to our DFT calculations, *trans*-2-oxa-7-azabicyclo[4.1.0]heptane has a 42.6 kcal/mol higher Gibbs energy than the *cis* isomer, and the introduction of two sp^2^-hybridized carbons, as in compounds **4**, increases this difference to 47.4 kcal/mol, making the formation of the discussed *trans*-fused systems impossible (see Figure S3 in Supporting Information File 1). According to the experiment, in the reaction of azirine **1b** with aldehyde **2b**, (1*RR*,3*SR*,6*RR*)-oxazabicyclo[4.1.0]heptanone **4b** is mainly formed (≈90%), while the minor one, based on the arguments presented above, is apparently the (1*RR*,3*RR*,6*RR*)-isomer. To shed light on the mechanism and stereoselectivity of the reaction, DFT calculations were performed at the B3LYP-D3/6-311+G(d,p)/LANL2DZ(Cs) level of theory with a SMD solvent model (for MeCN) for the reaction of azirine **1a** and benzaldehyde (**2c**, Scheme 3). Calculations indicate that condensation and subsequent cyclization should readily occur at room temperature, with only the diastereoisomer (1*RR*,3*SR*,6*RR*)-**Ph*^e^*-C** (with the Ph group from benzaldehyde in the equatorial position) exhibiting a lower relative Gibbs free energy than the starting compounds. According to the calculations, the reaction of azirine **1a** and benzaldehyde **2c** results in the formation of two diastereomers, (*RR,SR*)- and (*RR,RR*)-**A**, which can undergo a conformational transition to intermediates (*RR,SR*)- and (*RR,RR*)-**B**, which are capable of cyclization. The cyclization of (*RR,SR*)-**B** leads to the (1*RR*,3*SR*,6*RR*)-**Ph*^a^*-C** isomer with the Ph group from benzaldehyde in the axial position, which readily transforms into the more stable (1*RR*,3*SR*,6*RR*)-**Ph*^e^*-C** isomer with equatorial Ph group as a result of inversion of the 6-membered ring. The intermediate (*RR,RR*)-**B** immediately yields a more stable conformer with the Ph group from benzaldehyde in the equatorial position (1*RR*,3*RR*,6*RR*)-**Ph*^e^*-C**, but the equilibrium as a whole is shifted towards the most stable isomer (1*RR*,3*SR*,6*RR*)-**Ph*^e^*-C** isomer.

According to the calculation, the diastereoisomer (1*RR*,3*RR*,6*RR*)-**Ph*^e^*-4** has a Gibbs free energy 1.95 kcal/mol higher than the diastereoisomer (1*RR*,3*SR*,6*RR*)-**Ph*^e^*-4** (Scheme 3), i.e., under the conditions of the equilibrium reaction at 298 K, the diastereoisomer (1*RR*,3*SR*,6*RR*)-**Ph*^e^*-4** should be the main product (the ratio of diastereoisomers (1*RR*,3*SR*,6*RR*)-**Ph*^e^*-4**/(1*RR*,3*RR*,6*RR*)-**Ph*^e^*-4** ≈96:4), which corresponds to the results of the reaction of azirine **1b** with aldehyde **2b**.

Further efforts were directed toward finding a method for the controlled transformation of the cyclization product of aldol **3**, bicycle **4**, into a stable product. It turned out that if the reaction mixture obtained as a result of the reaction of azirine **1a** with 4-fluorobenzaldehyde (**2a**) is treated with TsOH after separation of cesium carbonate, 2-benzoyl-3-hydroxypyrrole **5a** is obtained as a product. Since only two examples of pyrroles **5** containing 2-aroyl and 3-hydroxy groups have been described so far [23–24], optimization of the reaction conditions was carried out with the aim of developing a new method for the preparation of 2-aroyl-3-hydroxypyrroles (Table 1).

**Table 1:** Optimization of the synthesis of pyrrole **5a**.^a^

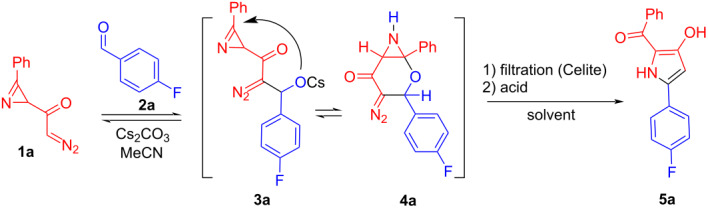					

Entry	Acid (equiv)	Solvent	*T* (°C)	Time (h)	Yield of **5a** (%)^b^

1	TsOH (0.2)	MeCN	80	1	no reaction
2	TsOH (0.1)	MeCN	100	0.5	37
3	TsOH (0.2)	MeCN	100	0.5	37
4	TsOH (0.3)	MeCN	100	0.5	49
**5**	**TsOH (0.4)**	**MeCN**	**100**	**0.5**	**56**
6	TsOH (0.5)	MeCN	100	0.5	52
7	TsOH (0.7)	MeCN	100	0.5	38
8	TsOH (1.0)	MeCN	100	0.5	20
9	TsOH (1.0)	MeCN	100	0.5	20
10	TsOH (0.4)	DCE	100	0.5	32
11	MsOH (0.2)	MeCN	25	2	48
12	MsOH (0.3)	MeCN	0	1	no reaction
**13**	**MsOH (0.4)**	**MeCN**	**25**	**2**	**62**
14	MsOH (1.0)	MeCN	25	1	13
15	BF_3_**⋅**Et_2_O (0.2)	MeCN	25	1	decomposition
16	aq HCl (0.2)	MeCN	100	0.5	35
17	aq HCl (0.2)	MeCN	25	1	31
18	FeCl_3_**⋅**6H_2_O (0.2)	MeCN	100	0.5	29
19	AcOH (0.4)	MeCN	50	0.5	decomposition
20	TFA (0.4)	MeCN	40	2	33
21	TfOH (0.4)	MeCN	25	1	decomposition
22	aq HI (0.4)	MeCN	25	1	16
23	TFAA (0.4)	MeCN	25	1	decomposition

^a^Reaction conditions: **1a** (0.3 mmol), **2a** (0.3 mmol), Cs_2_CO_3_ (0.15 mmol) in 2 mL of solvent. ^b^Isolated yields.

**Scheme 4:**
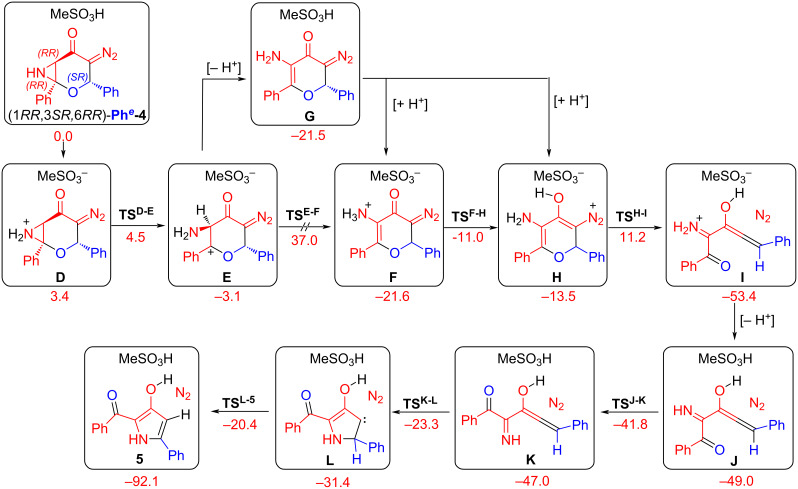
Plausible mechanism and relative Gibbs free energies for the acid-catalyzed transformation of the bicyclic intermediate (1*RR*,3*SR*,6*RR*)-**Ph*^e^*-4** to pyrrole **5** (in kcal/mol, 298 K, DFT B3LYP-D3/6-311+G(d,p) level of theory with a SMD solvent model for MeCN).

Thus, as a result of optimization it was found that the best yields of pyrrole **5a** was achieved by treating the reaction mixture containing **4a** (after separation of Cs_2_CO_3_) with TsOH at 100 °C or MsOH at 25 °C, with the acid clearly acting as a catalyst. Other protic acids, such as aq HCl, aq HI, and TFA, as well as a Lewis acid (FeCl_3_), also facilitated the conversion of **4a** to **5a**. Meanwhile, resinification of the reaction mixture was observed in the presence of AcOH, TfOH, TFAA, and BF_3_⋅Et_2_O. This is clearly due to the complex mechanism of formation of pyrrole **5a**. The role of acid catalysis follows from the plausible mechanism of transformation of the bicyclic intermediate (1*RR*,3*SR*,6*RR*)-**Ph*^e^*-4** to pyrrole **5** shown in Scheme 4, which was confirmed by DFT calculations at the B3LYP-D3/6-311+G(d,p) level of theory with a SMD solvent model for MeCN.

According to the calculations, the aziridine ring of protonated intermediate **D** readily opens to form the more stable cation **E**. The intramolecular H-shift leading to the ammonium cation **F** requires overcoming an excessively high energy barrier, so the conversion of intermediate **E** to intermediate **F** or directly to **H** apparently occurs via proton elimination/addition. The elimination of a nitrogen molecule from intermediate **H** is accompanied by the opening of the 6-membered ring and passes through relatively low barrier (Δ*G*^#^ = 24.7 kcal/mol), yielding intermediate **I**. Deprotonation of the latter leads to ketene **J**, which via rotation across single bond (intermediate **K**) can cyclize to carbene **L**. An intramolecular H-shift to the carbene center in **L** leads to the final pyrrole **5**.

**Scheme 5:**
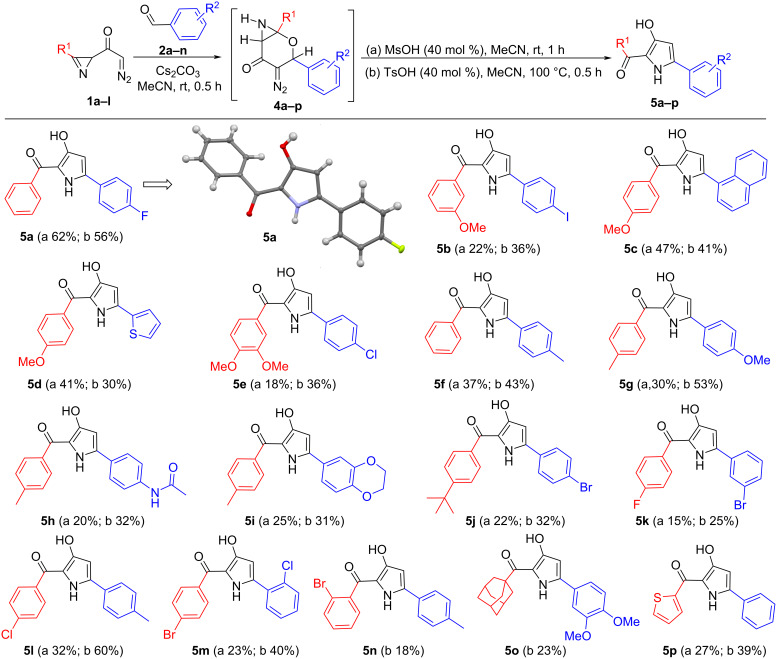
Synthesis of pyrroles **5**. Isolated yield calculated for two synthetic steps based on azirine **1**.

Further, using the found optimal conditions (Table 1), a series of 2-aroyl-3-hydroxypyrroles **5a–p** were synthesized (Scheme 5). The structure of compound **5a** was confirmed by an X-ray structural analysis (CCDC 2536266).

The yield of products **5a–m**,**p** was 25–62% in 2 synthetic steps, with complete conversion of the starting diazoketone **1a–i** and **l**, while under the conditions used it was not possible to achieve complete conversion of diazoketones **1j**,**k** and the yield of compounds **5n**,**o** was 18 and 23%, respectively. The first step of the reaction fails with aliphatic aldehyde (propanal) and the weakly electrophilic *N*-methylpyrrole-2-carbaldehyde and 4-hydroxybenzaldehyde, likely due to a shift in equilibrium toward the starting compounds. The reaction with strongly electrophilic, acceptor-substituted aromatic aldehydes, 4-nitro-, 4-cyanobenzaldehydes, and pyridine-4-carbaldehyde, is accompanied by complete resinification of the reaction medium in the first step.

**Scheme 6:**
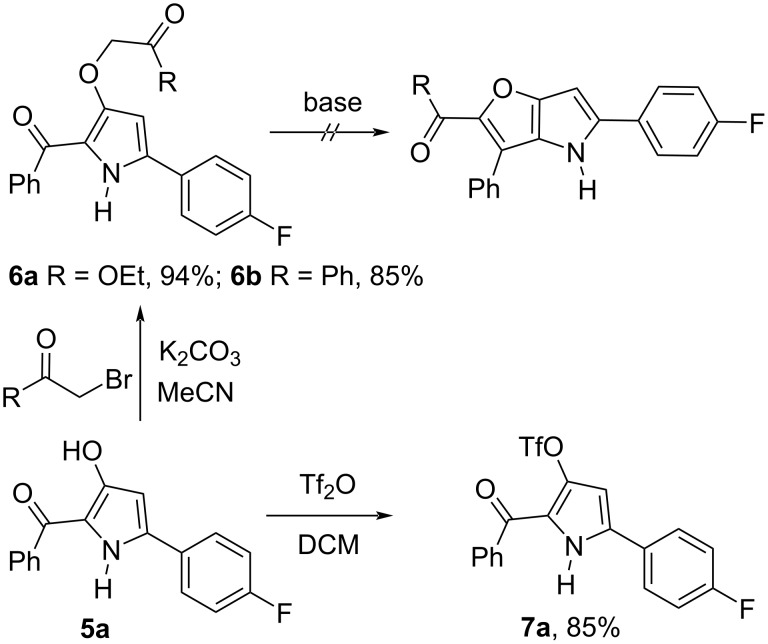
Alkylation and triflation reactions of pyrrole **5a**.

Despite the multifunctionality of compounds **5**, their modification potential proved to be rather limited. Compound **5a** is readily alkylated at the hydroxy group using 2-bromo-1-phenylethan-1-one and ethyl 2-bromoacetate, yielding compound **6a** and **6b** in high yield (Scheme 6). However, attempts to cyclize them using the benzoyl group to form 4*H*-furo[3,2-*b*]pyrrole derivatives were unsuccessful. Triflate **7a** is obtained from pyrrole **5a** by treatment with Tf_2_O in 85% yield, but it proved inert in the Suzuki reaction with 4-chlorophenylboronic acid and 2-(4,4,5,5-tetramethyl-1,3,2-dioxaborolan-2-yl)aniline as well as in the Sonogashira reaction with phenylacetylene.

## Conclusion

The Cs_2_CO_3_-induced condensation of 2-(diazoacetyl)-2*H*-azirines with aromatic aldehydes affords 2-aroyl-3-hydroxy-1*H*-pyrroles in moderate to good yields on two synthetic steps. The reaction proceeds via an azirinyl-substituted β-hydroxy-α-diazocarbonyl compound, which undergoes intramolecular cyclization involving the hydroxy group and the C=N bond of azirine, leading to the formation of a bicyclic intermediate, a 4-diazo-2-oxa-7-azabicyclo[4.1.0]heptan-5-one derivative. Acid-catalyzed transformation of the latter affords 2-aroyl-3-hydroxy-1*H*-pyrrole. A plausible mechanism of the transformation of the bicyclic intermediate to pyrrole was confirmed by DFT calculations.

## Supporting Information

Deposition number CCDC 2536266 (compound **5a**) contains the supplementary crystallographic data for this paper. These data are provided free of charge by the joint Cambridge Crystallographic Data Centre.

File 1 Full experimental details, characterization data and copies of NMR spectra for all new compounds.

File 2 Crystallographic information file for compound **5a**.

## Data Availability

All data that supports the findings of this study is available in the published article and/or the supporting information of this article.
