# Supplementary material for: Cascade transformation of 2-(diazoacetyl)-2H-azirines to 2-aroyl-3-hydroxy-1H-pyrroles via condensation with aromatic aldehydes
Source: Beilstein J Org Chem. 2026 Jun 9;22:897–904. doi: 10.3762/bjoc.22.70 (PMC13267485; doi:10.3762/bjoc.22.70)
Supplement: File 1 — Full experimental details, characterization data and copies of NMR spectra for all new compounds. [file Beilstein_J_Org_Chem-22-897-s001.pdf]

BEILSTEIN JOURNAL OF ORGANIC CHEMISTRY

Supporting Information

for

Cascade transformation of 2-(diazoacetyl)-2*H*-azirines to 2-aroyl-3-hydroxy-1*H*-pyrroles via condensation with aromatic aldehydes

Timur O. Zanakhov, Ekaterina E. Galenko, Mikhail S. Novikov
and Alexander F. Khlebnikov

Beilstein J. Org. Chem. **2026,** *22,* 897–904. doi:10.3762/bjoc.22.70

Full experimental details, characterization data and copies of NMR spectra for all new compounds

License and Terms: This is a supporting information file under the terms of the Creative Commons Attribution License (https://creativecommons.org/licenses/by/4.0). Please note that the reuse, redistribution and reproduction in particular requires that the author(s) and source are credited and that individual graphics may be subject to special legal provisions.
The license is subject to the *Beilstein Journal of Organic Chemistry* terms and conditions: (https://www.beilstein-journals.org/bjoc/terms)

Table of contents

1. General information and methods S2

2. Synthesis of (1*RR*,3*SR*,6*RR*)-4-diazo-3-(4-iodophenyl)-1-(3-methoxyphenyl)-2-oxa-7-azabicyclo[4.1.0]heptan-5-one **4b** S2

3. Synthesis of pyrroles **5a–p** S3

4. Reactions of pyrrole **5a** S12

6. X-ray diffraction experiments S14

7. ^{1}H, ^{13}C and DEPT spectra of new compounds S21

8. IR spectra of compound **4b** S82

9. Computational details S83

10. References S102

1. General information and methods.

Melting points were determined on a melting point apparatus. ^1H (400 MHz) and ^{13}C (100 MHz) NMR spectra were recorded on a NMR spectrometer in $CDCl_3$ or DMSO-d_6. Chemical shifts (δ) are reported in parts per million downfield from tetramethylsilane (TMS, δ = 0.00). ^1H NMR spectra were calibrated according to the residual peak of $CDCl_3$ (7.26 ppm), DMSO-d6 (2.50 ppm) and C_6D_6 (7.16 ppm); $^{13}C\{^1H\}$ and ^{13}C DEPT-135 spectra were calibrated according to the peak of $CDCl_3$ (77.00 ppm), DMSO-d_6 (39.51 ppm) and C_6D_6 (128.06 ppm). Electrospray ionization (ESI) mass spectra were recorded on a mass spectrometer, HRMS-ESI-QTOF. Single crystals of **5a** were obtained by slow crystallization from toluene at room temperature. Single-crystal X-ray data were collected using a Rigaku (Oxford Diffraction) «XtaLAB SuperNova» (Cu Kα, λ = 1.54184 Å, HyPix3000 type detector). Crystallographic data for the structures **5a** (CCDC 2536266) have been deposited with the Cambridge Crystallographic Data Centre. Thin-layer chromatography (TLC) was conducted on aluminum sheets with 0.2 mm silica gel with a fluorescent indicator. All solvents were dried and distilled prior to use. Physical and spectral data of 2-diazoacetyl-2*H*-azirines **1** prepared according to the published procedures were in agreement with previously reported values [1-4].

2. Synthesis of (1*RR*,3*SR*,6*RR*)-4-diazo-3-(4-iodophenyl)-1-(3-methoxyphenyl)-2-oxa-7-azabicyclo[4.1.0]heptan-5-one **4b**

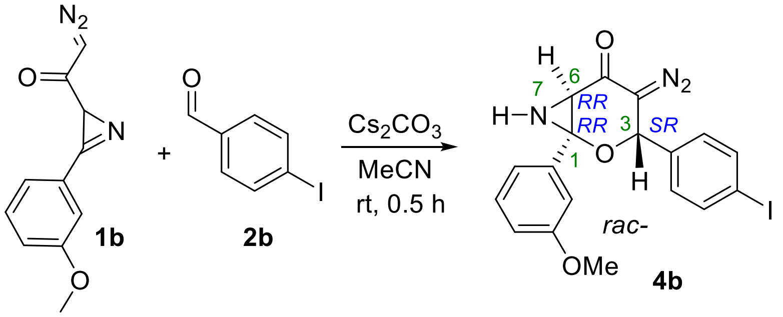

A mixture of 2-diazo-1-(3-(3-methoxyphenyl)-2*H*-azirin-2-yl)ethan-1-one (**1b**, 126 mg, 585 μmol), 4-iodobenzaldehyde (**2b**, 136 mg, 585 μmol) and Cs_2CO_3 (95 mg, 293 μmol) in acetonitrile (8 mL) stirred at rt for 0.5 h (monitored by TLC). The reaction mixture was diluted with DCM, filtered through a pad of Celite and washed with DCM. The solvent was evaporated, the residue was diluted with acetonitrile (4 mL), cooled for 30 min at -18 ºC, and precipitate was filtered and washed with cold acetonitrile (4 mL) to give compound **4b** in 128 mg (49% yield) as a beige solid: mp 127–128 °C (dec., DCM); ^1H NMR (400 MHz, C_6D_6): δ 7.30 (d, *J* = 8.4 Hz, 2H), 7.00-6.98 (m, 2H), 6.69-6.67 (m, 1H), 6.61-6.59 (m, 1H), 6.51 (d, *J* = 8.4 Hz, 2H), 5.86 (s, 1H), 3.24 (s, 3H), 2.85 (d, *J* = 10.5 Hz, 1H), 1.26 (d, *J* = 10.5 Hz, 1H); $^{13}C\{^1H\}$ NMR (100 MHz, C_6D_6): *δ* 187.0 (C), 160.4 (C), 138.2 (CH), 138.1 (C), 135.3 (C), 130.0 (CH), 129.8 (CH), 117.4 (CH), 114.4 (CH), 112.2 (CH), 95.7 (C), 74.0 (C), 69.1 (CH), 65.6 (C), 54.9 (CH_3), 41.7 (CH); IR (KBr, cm^{-1}): 2087 (CN_2); HRMS (ESI) *m/z* $[M - N_2 + H]^+$ calcd for $C_{18}H_{14}INO_3^+$ 420.0091, found 420.0096.

3. Synthesis of pyrroles 5a-p

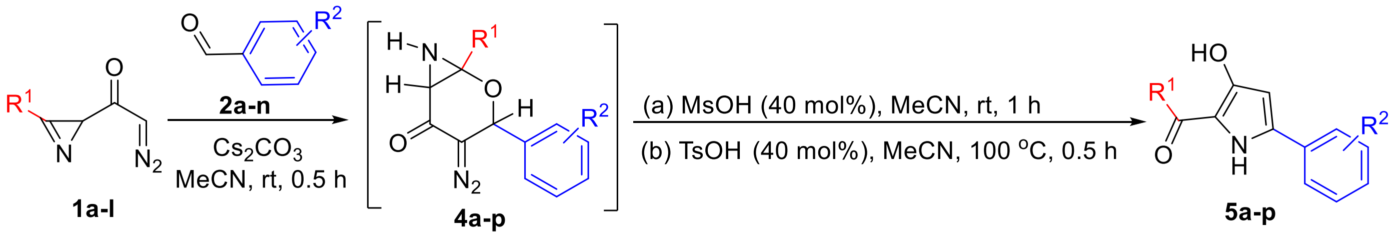

General procedure A for the preparation of pyrroles **5.** A mixture of azirine **1** (1.0 mmol), aldehyde **2** (1 mmol) and Cs_2CO_3 (0.5 mmol) in acetonitrile (4 mL) stirred at rt for 0.5 h (monitored by TLC). The mixture was filtered through a pad of Celite, washed with acetonitrile and TsOH monohydrate (0.4 mmol) was added to the solution. The mixture was stirred at 100 °C (oil bath temperature) for 1 h (monitored by TLC). The solvent was evaporated and the residue was purified by column chromatography on silica gel (light petroleum/ethyl acetate) to give pure compound **5**.

General procedure B for the preparation of pyrroles **5.** A mixture of azirine **1** (1.0 mmol), aldehyde **2** (1 mmol) and Cs_2CO_3 (0.5 mmol) in acetonitrile (4 mL) stirred at rt for 0.5 h (monitored by TLC). The mixture was filtered through a pad of Celite, washed with acetonitrile and MsOH (0.4 mmol) was added to the solution. The mixture was stirred at rt for 2 h (monitored by TLC). The solvent was evaporated and the residue was purified by column chromatography on silica gel (light petroleum/ethyl acetate) to give pure compound **5**.

(5-(4-Fluorophenyl)-3-hydroxy-1*H*-pyrrol-2-yl)(phenyl)methanone (5a)

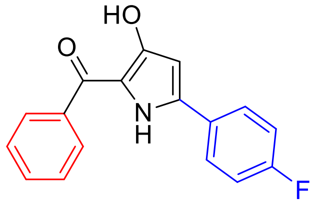

Compound **5a** was prepared following the general procedure A from 2-diazo-1-(3-phenyl-2*H*-azirin-2-yl)ethan-1-one (**1a**, 52 mg, 281 μmol), 4-fluorobenzaldehyde (**2a**, 35 mg, 281 μmol), Cs_2CO_3 (46 mg, 140 μmol) and TsOH monohydrate (21 mg, 112 μmol) in acetonitrile (4 mL) to give pure product in 44 mg (56% yield), after column chromatography on silica (light petroleum/ethyl acetate, 3:1, (v/v)).

Compound **5a** was also prepared following the general procedure B from 2-diazo-1-(3-phenyl-2*H*-azirin-2-yl)ethan-1-one (**1a**, 52 mg, 281 μmol), 4-fluorobenzaldehyde (**2a**, 35 mg, 281 μmol), Cs_2CO_3 (46 mg, 140 μmol) and MsOH (12 mg, 112 μmol) in acetonitrile (4 mL) to give pure product in 48 mg (62% yield), after column chromatography on silica (light petroleum/ethyl acetate, 3:1, (v/v)).

A light brown solid: mp 176–177 °C (light petroleum/ethyl acetate); ^{1}H NMR (400 MHz, $CDCl_3$): δ 10.36 (br. s, 1H), 8.02 (br. s, 1H), 7.82–7.79 (m, 2H), 7.60–7.55 (m, 3H), 7.53.7.48 (m, 2H), 7.16–7.10 (m, 2H), 6.22 (d, J = 2.6 Hz, 1H); ^{13}C{^{1}H} NMR (100 MHz, $CDCl_3$): δ 184.3 (C), 163.2 (C), 163.1 (d, J = 250.1 Hz, C), 138.02 (C), 137.96 (C), 131.7 (CH), 129.2 (CH), 127.5 (CH), 127.17 (d, J = 8.4 Hz, CH), 127.05 (d, J =

3.4 Hz, C), 116.9 (C), 116.3 (d, J = 22.1 Hz, CH), 96.6 (CH); HRMS (ESI) m/z $[M + H]^+$ calcd for $C_{17}H_{13}FNO_2^+$ 282.0925, found 282.0925.

(3-Hydroxy-5-(4-iodophenyl)-1*H*-pyrrol-2-yl)(3-methoxyphenyl)methanone (5b)

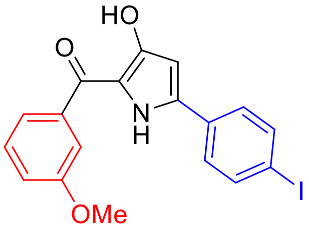

Compound **5b** was prepared following the general procedure A from 2-diazo-1-(3-(3-methoxyphenyl)-2*H*-azirin-2-yl)ethan-1-one (**1b**, 76 mg, 353 μmol), 4-iodobenzaldehyde (**2b**, 82 mg, 353 μmol), Cs_2CO_3 (58 mg, 177 μmol) and TsOH monohydrate (25 mg, 141 μmol) in acetonitrile (4 mL) to give pure product in 53 mg (36% yield), after column chromatography on silica (light petroleum/ethyl acetate, 4:1, (v/v)).

Compound **5b** was also prepared following the general procedure B from 2-diazo-1-(3-(3-methoxyphenyl)-2*H*-azirin-2-yl)ethan-1-one (**1b**, 73 mg, 339 μmol), 4-iodobenzaldehyde (**2b**, 79 mg, 339 μmol), Cs_2CO_3 (55 mg, 170 μmol) and MsOH (13 mg, 136 μmol) in acetonitrile (4 mL) to give pure product in 31 mg (22% yield), after column chromatography on silica (light petroleum/ethyl acetate, 4:1, (v/v)).

A light brown solid: mp 141–142 °C (light petroleum/ethyl acetate); ^1H NMR (400 MHz, $CDCl_3$): δ 10.25 (br. s, 1H), 8.13 (br. s, 1H), 7.76 (d, J = 8.4 Hz, 2H), 7.49–7.45 (m, 1H), 7.37–7.35 (m, 1H), 7.32–7.31 (m, 1H), 7.25 (d, J = 8.4 Hz, 2H), 7.14–7.11 (m, 1H), 6.25 (d, J = 2.8 Hz, 1H), 3.89 (s, 3H); $^{13}C\{^1H\}$ NMR (100 MHz, $CDCl_3$): δ 184.2 (C), 160.3 (C), 159.3 (C), 139.2 (C), 138.3 (CH), 137.6 (C), 130.2 (CH), 130.1 (C), 126.8 (CH), 119.5 (CH), 118.0 (CH), 117.0 (C), 112.6 (CH), 96.8 (CH), 94.6 (C), 55.5 (CH_3); HRMS (ESI) m/z $[M + H]^+$ calcd for $C_{18}H_{15}INO_3^+$ 420.0091, found 420.0095.

(3-Hydroxy-5-(naphthalen-1-yl)-1*H*-pyrrol-2-yl)(4-methoxyphenyl)methanone (5c)

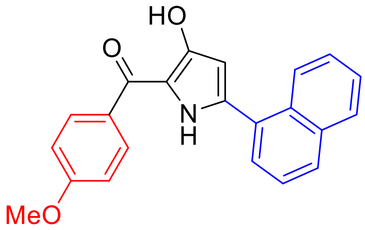

Compound **5c** was prepared following the general procedure A from 2-diazo-1-(3-(4-methoxyphenyl)-2*H*-azirin-2-yl)ethan-1-one (**1c**, 63 mg, 293 μmol), 1-naphthaldehyde (**2d**, 46 mg, 293 μmol), Cs_2CO_3 (48 mg, 146 μmol) and TsOH monohydrate (21 mg, 117 μmol) in acetonitrile (3 mL) to give pure product in 41 mg (41% yield), after column chromatography on silica (light petroleum/ethyl acetate, 3:1, (v/v)).

Compound **5c** was also prepared following the general procedure B from 2-diazo-1-(3-(4-methoxyphenyl)-2*H*-azirin-2-yl)ethan-1-one (**1c**, 62 mg, 288 μmol), 1-naphthaldehyde (**2d**, 45 mg, 288 μmol), Cs_2CO_3 (47 mg, 144 μmol) and MsOH (11 mg, 115 μmol) in acetonitrile (3 mL) to give pure product in 46 mg (47% yield), after column chromatography on silica (light petroleum/ethyl acetate, 3:1, (v/v)).

A light yellow solid: mp 147–148 °C (light petroleum/ethyl acetate); ^{1}H NMR (400 MHz, $CDCl_3$): δ 10.49 (br. s, 1H), 8.23–8.21 (m, 1H), 8.16 (br. s, 1H), 7.93–7.90 (m, 2H), 7.82 (d, J = 8.8 Hz, 2H), 7.58–7.50 (m, 4H), 7.01 (d, J = 8.3 Hz, 2H), 6.28 (d, J = 2.4 Hz, 1H), 3.87 (s, 3H); ^{13}C{^{1}H} NMR (100 MHz, $CDCl_3$): δ 183.6 (C), 162.4 (C), 158.8 (C), 137.7 (C), 133.8 (C), 131.1 (C), 130.5 (C), 129.7 (C), 129.60 (CH), 129.55 (CH), 128.6 (CH), 127.1 (CH), 126.9 (CH), 126.4 (CH), 125.2 (CH), 125.0 (CH), 116.5 (C), 114.3 (CH), 100.2 (CH), 55.4 (CH_3); HRMS (ESI) *m/z* [M + H]$^+$ calcd for $C_{22}H_{18}NO_3^+$ 344.1281, found 344.1279.

(3-Hydroxy-5-(thiophen-2-yl)-1*H*-pyrrol-2-yl)(4-methoxyphenyl)methanone (5d)

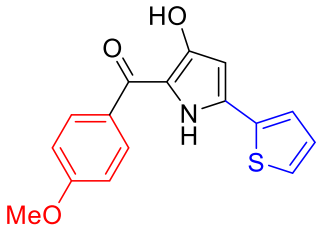

Compound **5d** was prepared following the general procedure A from 2-diazo-1-(3-(4-methoxyphenyl)-2*H*-azirin-2-yl)ethan-1-one (**1c**, 118 mg, 548 μmol), thiophene-2-carbaldehyde (**2e**, 61 mg, 548 μmol), Cs_2CO_3 (89 mg, 274 μmol) and TsOH monohydrate (40 mg, 219 μmol) in acetonitrile (5 mL) to give pure product in 49 mg (30% yield), after column chromatography on silica (light petroleum/ethyl acetate, 5:1, (v/v)).

Compound **5d** was also prepared following the general procedure B from 2-diazo-1-(3-(4-methoxyphenyl)-2*H*-azirin-2-yl)ethan-1-one (**1c**, 74 mg, 344 μmol), thiophene-2-carbaldehyde (**2e**, 39 mg, 344 μmol), Cs_2CO_3 (56 mg, 172 μmol) and MsOH (13 mg, 137 μmol) in acetonitrile (3 mL) to give pure product in 42 mg (41% yield), after column chromatography on silica (light petroleum/ethyl acetate, 5:1, (v/v)).

A light brown solid: mp 169–170 °C (light petroleum/ethyl acetate); ^{1}H NMR (400 MHz, $CDCl_3$): δ 10.45 (br. s, 1H), 8.02 (br. s, 1H), 7.79 (d, J = 8.8 Hz, 2H), 7.33 (dd, J = 5.0, 1.2 Hz, 1H), 7.25 (dd, J = 3.6, 1.2 Hz, 1H), 7.08 (dd, J = 5.0, 3.6 Hz, 1H), 7.04 (d, J = 8.8 Hz, 2H), 6.17 (d, J = 2.6 Hz, 1H), 3.90 (s, 3H); ^{13}C{^{1}H} NMR (100 MHz, $CDCl_3$): δ 183.2 (C), 162.5 (C), 159.3 (C), 134.0 (C), 133.1 (C), 130.4 (C), 129.6 (CH), 128.1 (CH), 126.0 (CH), 124.5 (CH), 116.3 (C), 114.3 (CH), 96.9 (CH), 55.5 (CH_3); HRMS (ESI) *m/z* [M + H]$^+$ calcd for $C_{16}H_{14}NO_3S^+$ 300.0689, found 300.0691.

(5-(4-Chlorophenyl)-3-hydroxy-1*H*-pyrrol-2-yl)(3,4-dimethoxyphenyl)methanone (5e)

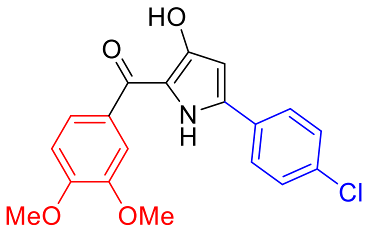

Compound **5e** was prepared following the general procedure A from 2-diazo-1-(3-(3,4-dimethoxyphenyl)-2*H*-azirin-2-yl)ethan-1-one (**1d**, 81 mg, 330 μmol), 4-chlorobenzaldehyde (**2f**, 46 mg, 330 μmol), Cs_2CO_3 (54 mg, 165 μmol) and TsOH monohydrate (24 mg, 132 μmol) in acetonitrile (4 mL) to give pure product in 42 mg (36% yield), after column chromatography on silica (light petroleum/ethyl acetate, 3:1, (v/v)).

Compound **5e** was also prepared following the general procedure B from from 2-diazo-1-(3-(3,4-dimethoxyphenyl)-2*H*-azirin-2-yl)ethan-1-one (**1d**, 83 mg, 338 μmol), 4-chlorobenzaldehyde (**2f**, 48 mg, 338 μmol), Cs_2CO_3 (55 mg, 169 μmol) and MsOH (13 mg, 135 μmol) in acetonitrile (4 mL) to give pure product in 22 mg (18% yield), after column chromatography on silica (light petroleum/ethyl acetate, 3:1, (v/v)).

An orange solid: mp 204–205 °C (light petroleum/ethyl acetate); ^{1}H NMR (400 MHz, DMSO-d_6): δ 11.19 (d, J = 2.9 Hz, 1H), 9.96 (br. s, 1H), 7.87 (d, J = 8.8 Hz, 2H), 7.49–7.44 (m, 3H), 7.42–7.41 (m, 1H), 7.05 (d, J = 8.4 Hz, 1H), 6.24 (d, J = 2.9 Hz, 1H), 3.84 (s, 3H), 3.82 (s, 3H); ^{13}C{^{1}H} NMR (100 MHz, DMSO-d_6): δ 182.0 (C), 152.6 (C), 151.6 (C), 147.9 (C), 135.7 (C), 132.4 (C), 131.1 (C), 129.9 (C), 128.7 (CH), 127.3 (CH), 122.9 (CH), 117.9 (C), 112.4 (CH), 110.6 (CH), 97.0 (CH), 55.6 (CH_3), 55.5 (CH_3); HRMS (ESI) m/z [M + H]$^+$ calcd for $C_{19}H_{17}ClNO_4^+$ 358.0841, found 358.0842.

(3-Hydroxy-5-(*p*-tolyl)-1*H*-pyrrol-2-yl)(phenyl)methanone (5f)

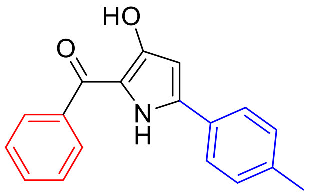

Compound **5f** was prepared following the general procedure A from 2-diazo-1-(3-phenyl-2*H*-azirin-2-yl)ethan-1-one (**1a**, 84 mg, 454 μmol), 4-methylbenzaldehyde (**2g**, 55 mg, 454 μmol), Cs_2CO_3 (74 mg, 227 μmol) and TsOH monohydrate (33 mg, 181 μmol) in acetonitrile (4 mL) to give pure product in 46 mg (37% yield), after column chromatography on silica (light petroleum/ethyl acetate, 5:1, (v/v)).

Compound **5f** was also prepared following the general procedure B from 2-diazo-1-(3-phenyl-2*H*-azirin-2-yl)ethan-1-one (**1a**, 80 mg, 432 μmol), 4-methylbenzaldehyde (**2g**, 52 mg, 432 μmol), Cs_2CO_3 (70 mg, 216 μmol) and MsOH (17 mg, 173 μmol) in acetonitrile (4 mL) to give pure product in 51 mg (43% yield), after column chromatography on silica (light petroleum/ethyl acetate, 5:1, (v/v)).

A light yellow solid: mp 164–165 °C (light petroleum/ethyl acetate); ^{1}H NMR (400 MHz, $CDCl_3$): δ 10.43 (br. s, 1H), 8.07 (br. s, 1H), 7.82–7.79 (m, 2H), 7.59–7.53 (m, 3H), 7.42 (d, J = 8.3 Hz, 2H), 7.23 (d, J = 8.3 Hz, 2H), 6.23 (d, J = 2.6 Hz, 1H), 2.39 (s, 3H); ^{13}C{^{1}H} NMR (100 MHz, $CDCl_3$): δ 184.0 (C), 159.8 (C), 139.3 (C), 139.3 (C), 138.0 (C), 131.6 (CH), 129.8 (CH), 129.1 (CH), 127.8 (C), 127.5 (CH), 125.1 (CH), 116.6 (C), 96.1 (CH), 21.3 (CH_3); HRMS (ESI) m/z [M + H]$^+$ calcd for $C_{18}H_{16}NO_2^+$ 278.1176, found 278.1181.

(3-Hydroxy-5-(4-methoxyphenyl)-1*H*-pyrrol-2-yl)(*p*-tolyl)methanone (5g)

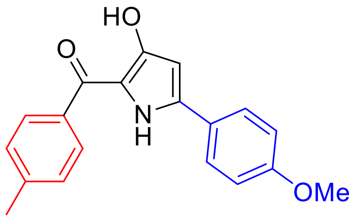

Compound **5g** was prepared following the general procedure A from 2-diazo-1-(3-(*p*-tolyl)-2*H*-azirin-2-yl)ethan-1-one (**1e**, 70 mg, 351 µmol), 4-methoxybenzaldehyde (**2h**, 48 mg, 351 µmol), Cs_2CO_3 (57 mg, 176 µmol) and TsOH monohydrate (25 mg, 141 µmol) in acetonitrile (4 mL) to give pure product in 57 mg (53% yield), after column chromatography on silica (light petroleum/ethyl acetate, 3:1, (v/v)).

Compound **5g** was also prepared following the general procedure B from 2-diazo-1-(3-(*p*-tolyl)-2*H*-azirin-2-yl)ethan-1-one (**1e**, 65 mg, 326 µmol), 4-methoxybenzaldehyde (**2h**, 44 mg, 326 µmol), Cs_2CO_3 (53 mg, 163 µmol) and MsOH (13 mg, 131 µmol) in acetonitrile (4 mL) to give pure product in 32 mg (30% yield), after column chromatography on silica (light petroleum/ethyl acetate, 3:1, (v/v)).

An orange oil; ^{1}H NMR (400 MHz, $CDCl_3$): δ 10.50 (br. s, 1H), 8.05 (br. s, 1H), 7.70 (d, J = 8.3 Hz, 2H), 7.46 (d, J = 8.8 Hz, 2H), 7.34 (d, J = 8.3 Hz, 2H), 6.94 (d, J = 8.8 Hz, 2H), 6.16 (d, J = 2.6 Hz, 1H), 3.84 (s, 3H), 2.45 (s, 3H); ^{13}C{^{1}H} NMR (100 MHz, $CDCl_3$): δ 183.7 (C), 160.3 (C), 142.1 (C), 139.0 (C), 135.4 (C), 129.7 (CH), 127.6 (CH), 126.6 (CH), 123.4 (C), 116.5 (C), 114.6 (CH), 95.7 (CH), 55.4 (CH_3), 21.6 (CH_3); HRMS (ESI) *m/z* $[M + H]^+$ calcd for $C_{19}H_{18}NO_3^+$ 308.1281, found 308.1282.

N-(4-(4-Hydroxy-5-(4-methylbenzoyl)-1*H*-pyrrol-2-yl)phenyl)acetamide (5h)

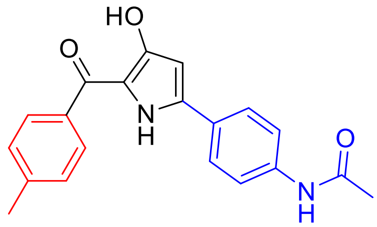

Compound **5h** was prepared following the general procedure A from 2-diazo-1-(3-(*p*-tolyl)-2*H*-azirin-2-yl)ethan-1-one (**1e**, 107 mg, 537 µmol), *N*-(4-formylphenyl)acetamide (**2i**, 88 mg, 537 µmol), Cs_2CO_3 (88 mg, 269 µmol) and TsOH monohydrate (39 mg, 215 µmol) in acetonitrile (5 mL) to give pure product in 57 mg (32% yield), after column chromatography on silica (light petroleum/ethyl acetate, 1:2, (v/v)).

Compound **5h** was also prepared following the general procedure B from 2-diazo-1-(3-(*p*-tolyl)-2*H*-azirin-2-yl)ethan-1-one (**1e**, 103 mg, 517 µmol), *N*-(4-formylphenyl)acetamide (**2i**, 84 mg, 517 µmol), Cs_2CO_3 (84 mg, 259 µmol) and MsOH (20 mg, 207 µmol) in acetonitrile (5 mL) to give pure product in 35 mg (20% yield), after column chromatography on silica (light petroleum/ethyl acetate, 3:1, (v/v)).

A beige solid: mp 265–266 °C (light petroleum/ethyl acetate); ^{1}H NMR (400 MHz, DMSO-d_6): δ 11.02 (d, J = 2.9 Hz, 1H), 10.04 (s, 1H), 7.77 (d, J = 8.8 Hz, 2H), 7.68 (d, J = 8.1 Hz, 2H), 7.60 (d, J = 8.8 Hz, 2H), 7.27 (d, J = 8.1 Hz, 2H), 6.14 (d, J = 2.9 Hz, 1H), 2.38 (s, 3H), 2.06 (s, 3H); ^{13}C{^{1}H} NMR (100 MHz, DMSO-d_6): δ 182.3 (C), 168.5 (C), 153.6 (C), 140.9 (C), 139.2 (C), 137.7 (C), 136.3 (C), 128.9 (CH), 128.4 (CH), 126.2 (CH), 125.7 (C), 118.9 (CH), 117.5 (C), 95.9 (CH), 24.1 (CH_3), 21.1 (CH_3); HRMS (ESI) *m/z* $[M + H]^+$ calcd for $C_{20}H_{19}N_2O_3^+$ 335.1390, found 335.1392.

(5-(2,3-Dihydrobenzo[*b*][1,4]dioxin-6-yl)-3-hydroxy-1*H*-pyrrol-2-yl)(*p*-tolyl)methanone (5i)

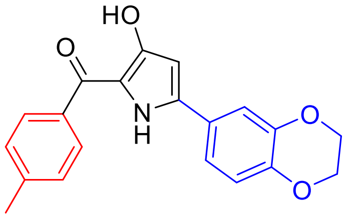

Compound **5i** was prepared following the general procedure A from 2-diazo-1-(3-(*p*-tolyl)-2*H*-azirin-2-yl)ethan-1-one (**1e**, 53 mg, 266 μmol), 2,3-dihydrobenzo[*b*][1,4]dioxine-6-carbaldehyde (**2j**, 44 mg, 266 μmol), Cs_2CO_3 (43 mg, 133 μmol) and TsOH monohydrate (19 mg, 106 μmol) in acetonitrile (3 mL) to give pure product in 28 mg (31% yield), after column chromatography on silica (light petroleum/ethyl acetate, 3:1, (v/v)).

Compound **5i** was also prepared following the general procedure B from 2-diazo-1-(3-(*p*-tolyl)-2*H*-azirin-2-yl)ethan-1-one (**1e**, 50 mg, 251 μmol), 2,3-dihydrobenzo[*b*][1,4]dioxine-6-carbaldehyde (**2j**, 41 mg, 251 μmol), Cs_2CO_3 (41 mg, 126 μmol) and MsOH (10 mg, 100 μmol) in acetonitrile (3 mL) to give pure product in 21 mg (25% yield), after column chromatography on silica (light petroleum/ethyl acetate, 3:1, (v/v)).

An orange oil; ^{1}H NMR (400 MHz, $CDCl_3$): δ 10.43 (br. s, 1H), 7.97 (br. s, 1H), 7.70 (d, J = 8.3 Hz, 2H), 7.35 (d, J = 8.3 Hz, 2H), 7.03–7.00 (m, 2H), 6.92–6.89 (m, 1H), 6.15 (d, J = 2.6 Hz, 1H), 4.29 (s, 4H), 2.46 (s, 3H); ^{13}C{^{1}H} NMR (100 MHz, $CDCl_3$): δ 183.8 (C), 144.5 (C), 143.9 (C), 142.2 (C), 138.7 (C), 135.3 (C), 129.7 (CH), 127.6 (CH), 124.3 (C), 118.7 (CH), 118.0 (CH), 116.6 (C), 114.1 (CH), 95.9 (CH), 64.5 (CH_2), 64.4 (CH_2), 21.5 (CH_3); HRMS (ESI) *m/z* $[M + H]^+$ calcd for $C_{20}H_{18}NO_4^+$ 336.1230, found 336.1233.

(5-(4-Bromophenyl)-3-hydroxy-1*H*-pyrrol-2-yl)(4-(*tert*-butyl)phenyl)methanone (5j)

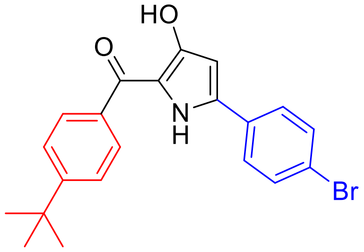

Compound **5j** was prepared following the general procedure A from 1-(3-(4-(*tert*-butyl)phenyl)-2*H*-azirin-2-yl)-2-diazoethan-1-one (**1f**, 72 mg, 298 μmol), 4-bromobenzaldehyde (**2k**, 55 mg, 298 μmol), Cs_2CO_3 (49 mg, 149 μmol) and TsOH monohydrate (22 mg, 119 μmol) in acetonitrile (3 mL) to give pure product in 38 mg (32% yield), after column chromatography on silica (light petroleum/ethyl acetate, 10:1, (v/v)).

Compound **5j** was also prepared following the general procedure B from from 1-(3-(4-(*tert*-butyl)phenyl)-2*H*-azirin-2-yl)-2-diazoethan-1-one (**1f**, 67 mg, 278 μmol), 4-bromobenzaldehyde (**2k**, 51 mg, 278 μmol), Cs_2CO_3 (45 mg, 139 μmol) and MsOH (11 mg, 111 μmol) in acetonitrile (3 mL) to give pure product in 24 mg (22% yield), after column chromatography on silica (light petroleum/ethyl acetate, 10:1, (v/v)).

A yellow solid: mp 217–218 °C (light petroleum/ethyl acetate); ^{1}H NMR (400 MHz, $CDCl_3$): δ 10.33 (br. s, 1H), 8.13 (br. s, 1H), 7.75 (d, J = 8.4 Hz, 2H), 7.59–7.54 (m, 4H), 7.39 (d, J = 8.4 Hz, 2H), 6.25 (d, J = 2.6

Hz, 1H), 1.38 (s, 9H); $^{13}C\{^1H\}$ NMR (100 MHz, $CDCl_3$): δ 184.4 (C), 159.3 (C), 155.5 (C), 137.2 (C), 135.1 (C), 132.3 (CH), 129.7 (C), 127.4 (CH), 126.6 (CH), 126.1 (CH), 123.0 (C), 117.1 (C), 96.8 (CH), 35.1 (C), 31.1 (CH_3); HRMS (ESI) *m/z* $[M + H]^+$ calcd for $C_{21}H_{21}BrNO_2^+$ 398.0750, found 398.0752.

(5-(3-Bromophenyl)-3-hydroxy-1*H*-pyrrol-2-yl)(4-fluorophenyl)methanone (5k)

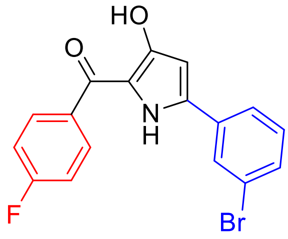

Compound **5k** was prepared following the general procedure A from 2-diazo-1-(3-(4-fluorophenyl)-2*H*-azirin-2-yl)ethan-1-one (**1g**, 84 mg, 413 μmol), 3-bromobenzaldehyde (**2l**, 77 mg, 413 μmol), Cs_2CO_3 (67 mg, 207 μmol) and TsOH monohydrate (15 mg, 83 μmol) in acetonitrile (4 mL) to give pure product in 37 mg (25% yield), after column chromatography on silica (light petroleum/ethyl acetate, 3:1, (v/v)).

Compound **5k** was also prepared following the general procedure B from 2-diazo-1-(3-(4-fluorophenyl)-2*H*-azirin-2-yl)ethan-1-one (**1g**, 102 mg, 502 μmol), 3-bromobenzaldehyde (**2l**, 93 mg, 502 μmol), Cs_2CO_3 (82 mg, 251 μmol) and MsOH (19 mg, 201 μmol) in acetonitrile (4 mL) to give pure product in 28 mg (15% yield), after column chromatography on silica (light petroleum/ethyl acetate, 3:1, (v/v)).

A light yellow solid: mp 165–166 °C (light petroleum/ethyl acetate); ^1H NMR (400 MHz, $CDCl_3$): δ 10.18 (br. s, 1H), 8.09 (d, J = 9.0 Hz, 1H), 7.86–7.82 (m, 2H), 7.66 (s, 1H), 7.52–7.49 (m, 1H), 7.47–7.45 (m, 1H), 7.33–7.31 (m, 1H), 7.29–7.24 (m, 2H), 6.27 (d, J = 2.6 Hz, 1H); $^{13}C\{^1H\}$ NMR (100 MHz, $CDCl_3$): δ 183.2 (C), 164.8 (d, J = 253.2 Hz, C), 137.3 (C), 134.1 (d, J = 3.3 Hz, C), 132.7 (C), 131.9 (CH), 130.7 (CH), 130.0 (d, J = 9.0 Hz, CH), 128.2 (CH), 124.0 (CH), 123.3 (C), 117.0 (C), 116.3 (d, J = 21.9 Hz, CH), 97.4 (CH); HRMS (ESI) *m/z* $[M + H]^+$ calcd for $C_{17}H_{12}BrFNO_2^+$ 360.0030, found 360.0031.

(4-Chlorophenyl)(3-hydroxy-5-(*p*-tolyl)-1*H*-pyrrol-2-yl)methanone (5l).

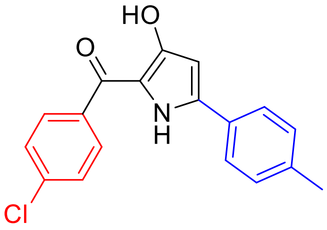

Compound **5l** was prepared following the general procedure A from 1-(3-(4-chlorophenyl)-2*H*-azirin-2-yl)-2-diazoethan-1-one **1h** (62 mg, 282 μmol), 4-methylbenzaldehyde **2g** (34 mg, 282 μmol), Cs_2CO_3 (46 mg, 141 μmol) and TsOH monohydrate (21 mg, 113 μmol) in acetonitrile (4 mL) to give pure product in 26 mg (30% yield), after column chromatography on silica (light petroleum/ethyl acetate, 3:1, (v/v)).

Compound **5l** was also prepared following the general procedure B from 1-(3-(4-chlorophenyl)-2*H*-azirin-2-yl)-2-diazoethan-1-one **1h** (68 mg, 310 μmol), 4-methylbenzaldehyde **2g** (37 mg, 310 μmol), Cs_2CO_3 (50

mg, 155 μmol) and MsOH (12 mg, 124 μmol) in acetonitrile (4 mL) to give pure product in 48 mg (62% yield), after column chromatography on silica (light petroleum/ethyl acetate, 3:1, (v/v)).

A light brown solid: mp 157–157 °C (light petroleum/ethyl acetate); ^{1}H NMR (400 MHz, $CDCl_3$): δ 10.36 (br. s, 1H), 7.99 (br. s, 1H), 7.76 (d, J = 8.4 Hz, 2H), 7.54 (d, J = 8.4 Hz, 2H), 7.42 (d, J = 8.1 Hz, 2H), 7.24 (d, J = 8.1 Hz, 2H), 6.24 (d, J = 2.6 Hz, 1H), 2.39 (s, 3H); ^{13}C{^{1}H} NMR (100 MHz, $CDCl_3$): δ 182.5 (C), 139.9 (C), 139.5 (C), 137.9 (C), 136.3 (C), 129.9 (CH), 129.4 (CH), 129.0 (CH), 127.7 (C), 125.2 (CH), 116.6 (C), 96.3 (CH), 21.3 (CH_3); HRMS (ESI) m/z [M + H]$^+$ calcd for $C_{18}H_{15}ClNO_2^+$ 312.0786, found 312.0783.

(4-Bromophenyl)(5-(2-chlorophenyl)-3-hydroxy-1*H*-pyrrol-2-yl)methanone (5m)

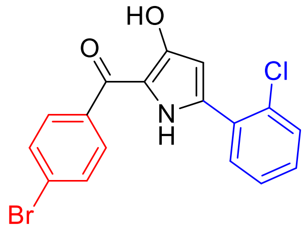

Compound **5m** was prepared following the general procedure A from 1-(3-(4-bromophenyl)-2*H*-azirin-2-yl)-2-diazoethan-1-one (**1i**, 60 mg, 227 μmol), 2-chlorobenzaldehyde (**2m**, 32 mg, 227 μmol), Cs_2CO_3 (37 mg, 114 μmol) and TsOH monohydrate (17 mg, 91 μmol) in acetonitrile (3 mL) to give pure product in 34 mg (40% yield), after column chromatography on silica (light petroleum/ethyl acetate, 3:1, (v/v)).

Compound **5m** was also prepared following the general procedure B from 1-(3-(4-bromophenyl)-2*H*-azirin-2-yl)-2-diazoethan-1-one (**1i**, 63 mg, 239 μmol), 2-chlorobenzaldehyde (**2m**, 32 mg, 239 μmol), Cs_2CO_3 (39 mg, 119 μmol) and MsOH (9 mg, 95 μmol) in acetonitrile (3 mL) to give pure product in 21 mg (23% yield), after column chromatography on silica (light petroleum/ethyl acetate, 3:1, (v/v)).

A light yellow solid: mp 170–171 °C (light petroleum/ethyl acetate); ^{1}H NMR (400 MHz, $CDCl_3$): δ 10.06 (br. s, 1H), 8.84 (br. s, 1H), 7.73–7.68 (m, 4H), 7.59 (dd, J = 7.5, 2.0 Hz, 1H), 7.46 (dd, J = 7.7, 1.7 Hz, 1H), 7.38 – 7.28 (m, 2H), 6.30 (d, J = 2.8 Hz, 1H); ^{13}C{^{1}H} NMR (100 MHz, $CDCl_3$): δ 183.2 (C), 158.5 (C), 136.5 (C), 135.9 (C), 132.4 (CH), 131.2 (CH), 130.7 (C), 130.5 (CH), 129.9 (CH), 129.1 (CH), 128.8 (C), 127.6 (CH), 126.7 (C), 116.5 (C), 99.0 (CH); HRMS (ESI) m/z [M + H]$^+$ calcd for $C_{17}H_{12}BrClNO_2^+$ 375.9735, found 375.9736.

(2-Bromophenyl)(3-hydroxy-5-(*p*-tolyl)-1*H*-pyrrol-2-yl)methanone (5n)

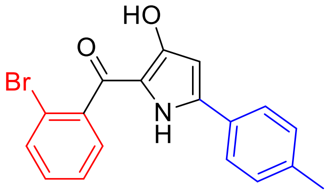

Compound **5n** was prepared following the general procedure A from 1-(3-(2-bromophenyl)-2*H*-azirin-2-yl)-2-diazoethan-1-one (**1j**, 105 mg, 398 μmol), 4-methylbenzaldehyde (**2g**, 48 mg, 398 μmol), Cs_2CO_3 (65 mg, 199 μmol) and TsOH monohydrate (29 mg, 159 μmol) in acetonitrile (5 mL) to give pure product in 25 mg

(18% yield), after column chromatography on silica (light petroleum/ethyl acetate, 5:1, (v/v)) as a light brown solid: mp 192–193 °C (light petroleum/ethyl acetate); ^{1}H NMR (400 MHz, $CDCl_3$): δ 10.01 (br. s, 1H), 7.75 (br. s, 1H), 7.72–7.70 (m, 1H), 7.51–7.44 (m, 2H), 7.40–7.36 (m, 3H), 7.21–7.19 (m, 2H), 6.21 (d, J = 2.6 Hz, 1H), 2.37 (s, 3H); ^{13}C{^{1}H} NMR (100 MHz, $CDCl_3$): δ 183.2 (C), 159.7 (C), 140.0 (C), 139.5 (C), 138.9 (C), 133.8 (CH), 131.6 (CH), 129.8 (CH), 129.0 (CH), 127.7 (C), 127.7 (CH), 125.2 (CH), 119.5 (C), 116.9 (C), 96.1 (CH), 21.3 (CH_3); HRMS (ESI) m/z $[M + H]^+$ calcd for $C_{18}H_{14}BrNO_2^+$ 356.0281, found 356.0273.

(Adamantan-1-yl)(5-(3,4-dimethoxyphenyl)-3-hydroxy-1*H*-pyrrol-2-yl)methanone (5o)

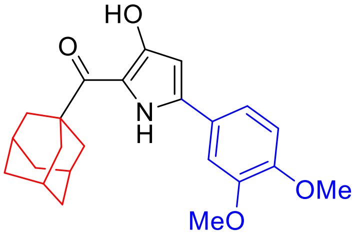

Compound **5o** was prepared following the general procedure A from 1-(3-(adamantan-1-yl)-2*H*-azirin-2-yl)-2-diazoethan-1-one (**1k**, 86 mg, 353 µmol), 3,4-dimethoxybenzaldehyde (**2n**, 59 mg, 353 µmol), Cs_2CO_3 (58 mg, 177 µmol) and TsOH monohydrate (26 mg, 141 µmol) in acetonitrile (4 mL) to give pure product in 31 mg (23% yield), after column chromatography on silica (light petroleum/ethyl acetate, 5:1, (v/v)) as a beige solid: mp 195–196 °C (light petroleum/ethyl acetate); ^{1}H NMR (400 MHz, $CDCl_3$): δ 11.32 (br. s, 1H), 8.09 (br. s, 1H), 7.08 (dd, J = 8.3, 2.2 Hz, 1H), 7.01 (d, J = 2.2 Hz, 1H), 6.93 (d, J = 8.3 Hz, 1H), 6.07 (d, J = 2.6 Hz, 1H), 3.95 (s, 3H), 3.93 (s, 3H), 2.15–2.13 (m, 3H), 2.09–2.08 (m, 6H), 1.86–1.78 (m, 6H); ^{13}C{^{1}H} NMR (100 MHz, $CDCl_3$): δ 195.6 (C), 161.0 (C), 150.0 (C), 149.5 (C), 137.5 (C), 124.2 (C), 117.6 (CH), 115.4 (C), 111.6 (CH), 109.2 (CH), 95.2 (CH), 56.2 (CH_3), 56.0 (CH_3), 44.9 (C), 39.5 (CH_2), 36.7 (CH_2), 28.1 (CH); HRMS (ESI) m/z $[M + H]^+$ calcd for $C_{23}H_{28}NO_4^+$ 382.2013, found 382.2018.

(3-Hydroxy-5-phenyl-1*H*-pyrrol-2-yl)(thiophen-2-yl)methanone (5p)

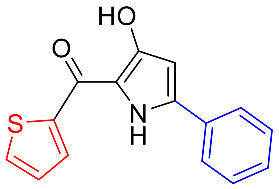

Compound **5p** was prepared following the general procedure A from 2-diazo-1-(3-(thiophen-2-yl)-2*H*-azirin-2-yl)ethan-1-one (**1l**, 81 mg, 423 µmol), benzaldehyde (**2c**, 45 mg, 423 µmol), Cs_2CO_3 (69 mg, 212 µmol) and TsOH monohydrate (30 mg, 169 µmol) in acetonitrile (4 mL) to give pure product in 44 mg (39% yield), after column chromatography on silica (light petroleum/ethyl acetate, 4:1, (v/v)).

Compound **5p** was also prepared following the general procedure B from 2-diazo-1-(3-(thiophen-2-yl)-2*H*-azirin-2-yl)ethan-1-one (**1l**, 77 mg, 403 µmol), benzaldehyde (**2c**, 43 mg, 403 µmol), Cs_2CO_3 (66 mg, 201 µmol) and MsOH (15 mg, 161 µmol) in acetonitrile (4 mL) to give pure product in 29 mg (27% yield), after column chromatography on silica (light petroleum/ethyl acetate, 4:1, (v/v)).

A light brown solid: mp 130–131 °C (light petroleum/ethyl acetate); ^{1}H NMR (400 MHz, $CDCl_3$): δ 10.39 (br. s, 1H), 8.35 (br. s, 1H), 7.80 (dd, J = 3.7, 1.3 Hz, 1H), 7.68 (dd, J = 5.0, 1.3 Hz, 1H), 7.49–7.56 (m, 2H), 7.48–7.44 (m, 2H), 7.42–7.38 (m, 1H), 7.25 (dd, J = 5.0, 3.7 Hz, 1H), 6.28 (d, J = 2.6 Hz, 1H); ^{13}C{^{1}H} NMR (100 MHz, $CDCl_3$): δ 174.9 (C), 160.2 (C), 141.6 (C), 139.5 (C), 131.5 (CH), 130.6 (C), 130.2 (CH), 129.20 (CH), 129.17 (CH), 128.2 (CH), 125.3 (CH), 116.2 (C), 96.8 (CH); HRMS (ESI) *m/z* [M + H]$^+$ calcd for $C_{15}H_{12}NO_2S^+$ 270.0583, found 270.0587.

4. Reactions of pyrrole 5a

Ethyl 2-((2-benzoyl-5-(4-fluorophenyl)-1*H*-pyrrol-3-yl)oxy)acetate (6a)

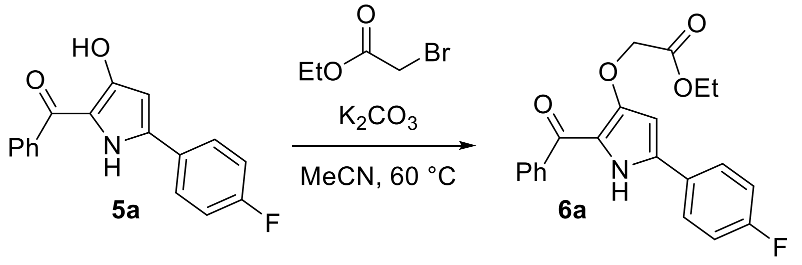

Compound **6a** was prepared following the published procedure [5] from (5-(4-fluorophenyl)-3-hydroxy-1*H*-pyrrol-2-yl)(phenyl)methanone (**5a**, 57 mg, 202 μmol), ethyl 2-bromoacetate (68 mg, 405 μmol), and K_2CO_3 (84 mg, 608 μmol) in acetonitrile (3 mL) to give pure product in 70 mg (94% yield), after column chromatography on silica (light petroleum/ethyl acetate, 3:1, (v/v)) as a beige solid: mp 166–167 °C (light petroleum/ethyl acetate); ^{1}H NMR (400 MHz, DMSO-d_6): δ 11.67 (m, 1H), 7.96–7.93 (m, 2H), 7.82–7.80 (m, 2H), 7.55–7.50 (m, 1H), 7.45–7.41 (m, 2H), 7.29–7.24 (m, 2H), 6.51 (d, J = 2.9 Hz, 1H), 4.66 (s, 2H), 4.14 (q, J = 7.1 Hz, 2H), 1.19 (t, J = 7.1 Hz, 3H); ^{13}C{^{1}H} NMR (100 MHz, DMSO-d_6): δ 182.4 (C), 168.4 (C), 161.9 (d, J = 245.6 Hz, C), 151.7 (C), 139.0 (C), 135.8 (C), 131.1 (CH), 128.9 (CH), 127.7 (d, J = 8.2 Hz, CH), 127.6 (CH), 127.4 (d, J = 3.0 Hz, C), 118.1 (C), 115.6 (d, J = 21.7 Hz, CH), 94.6 (CH), 66.9 (CH_2), 60.6 (CH_2), 14.0 (CH_3); HRMS (ESI) *m/z* [M + H]$^+$ calcd for $C_{21}H_{19}FNO_4^+$ 368.1293, found 368.1294.

2-((2-Benzoyl-5-(4-fluorophenyl)-1*H*-pyrrol-3-yl)oxy)-1-phenylethan-1-one (6b)

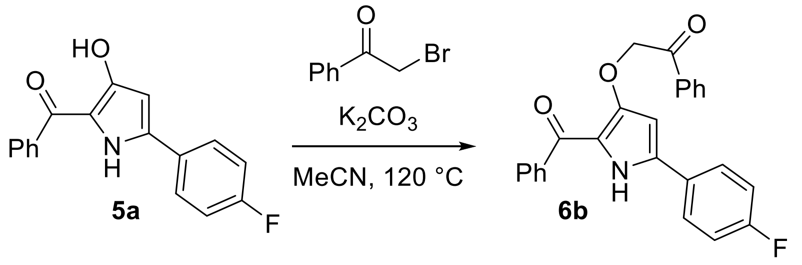

Compound **6b** was prepared following the published procedure [6] from (5-(4-fluorophenyl)-3-hydroxy-1*H*-pyrrol-2-yl)(phenyl)methanone (**5a**, 23 mg, 82 μmol), 2-bromo-1-phenylethan-1-one (33 mg, 164 μmol), and K_2CO_3 (34 mg, 245 μmol) in acetonitrile (2 mL) to give pure product in 28 mg (85% yield), after column chromatography on silica (light petroleum/ethyl acetate, 3:1, (v/v)) as a beige solid: mp 176–175 °C (light petroleum/ethyl acetate); ^{1}H NMR (400 MHz, DMSO-d_6): δ 11.62 (br. s, 1H), 7.95–7.91 (m, 4H),

7.87–7.85 (m, 2H), 7.71–7.66 (m, 1H), 7.57–7.53 (m, 2H), 7.51–7.48 (m, 1H), 7.42–7.38 (m, 2H), 7.26–7.22 (m, 2H), 6.54 (d, J = 2.9 Hz, 1H), 5.47 (s, 2H); $^{13}C\{^{1}H\}$ NMR (100 MHz, DMSO-d_6): δ 194.4 (C), 182.4 (C), 161.9 (d, J = 245.6 Hz, C), 152.3 (C), 139.1 (C), 135.9 (C), 134.3 (C), 133.8 (CH), 131.1 (CH), 128.9 (CH), 128.8 (C), 127.9 (CH), 127.64 (d, J = 8.4 Hz, CH), 127.57 (CH), 127.5 (d, J = 3.3 Hz, C), 118.1 (C), 115.6 (d, J = 21.7 Hz, CH), 94.8 (CH), 72.6 (CH_2); HRMS (ESI) m/z $[M + H]^+$ calcd for $C_{25}H_{19}FNO_3^+$ 422.1163, found 422.1159.

2-Benzoyl-5-(4-fluorophenyl)-1*H*-pyrrol-3-yl trifluoromethanesulfonate (7a)

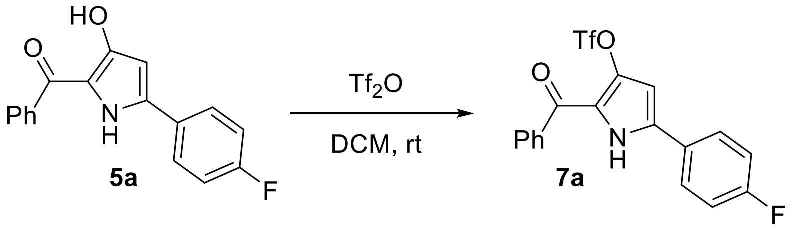

Compound **7a** was prepared following the published procedure [7] from (5-(4-fluorophenyl)-3-hydroxy-1*H*-pyrrol-2-yl)(phenyl)methanone (**5a**, 93 mg, 331 μmol), Et_3N (100 mg, 992 μmol), and Tf_2O (187 mg, 661 μmol) in DCM (3 mL) to give pure product in 116 mg (85% yield), after column chromatography on silica (light petroleum/ethyl acetate, 5:1, (v/v)) as a light yellow solid: mp 182–183 °C (light petroleum/ethyl acetate); ^{1}H NMR (400 MHz, $CDCl_3$): δ 10.04 (br. s, 1H), 7.80–7.77 (m, 2H), 7.65–7.61 (m, 3H), 7.53–7.51 (m, 2H), 7.16–7.12 (m, 2H), 6.53 (d, J = 3.1 Hz, 1H); $^{13}C\{^{1}H\}$ NMR (100 MHz, $CDCl_3$): δ 184.1 (C), 163.3 (d, J = 250.3 Hz, C), 138.4 (C), 137.0 (C), 135.7 (C), 132.9 (CH), 128.9 (CH), 128.5 (CH), 127.3 (d, J = 8.4 Hz, CH), 126.0 (d, J = 3.7 Hz, C), 121.5 (C), 118.3 (q, J = 321.4 Hz, C), 116.5 (d, J = 21.9 Hz, CH), 101.3 (CH); HRMS (ESI) m/z $[M + H]^+$ calcd for $C_{18}H_{12}F_4NO_4S^+$ 436.0237, found 436.0243.

6. X-ray diffraction experiments

Crystal structures of **5a** was determined by single crystal X-ray diffraction analysis. Suitable crystals were selected and fixed on micro-amounts and the diffraction data were collected on diffractometer. The crystals **5a** was measured at temperature 100 K, using monochromated CuKα radiation. The unit cell parameters and refinement characteristics of the crystal structures of **5a** is given below. Using Olex2 [8], the structure was solved with the ShelXT [9] structure solution program using Intrinsic Phasing and refined with the ShelXL [10] refinement package using Least Squares minimization.

(5-(4-Fluorophenyl)-3-hydroxy-1*H*-pyrrol-2-yl)(phenyl)methanone (5a)

Single crystals of **5a** were obtained by slow recrystallization from dichloromethane at room temperature (CCDC 2536266)

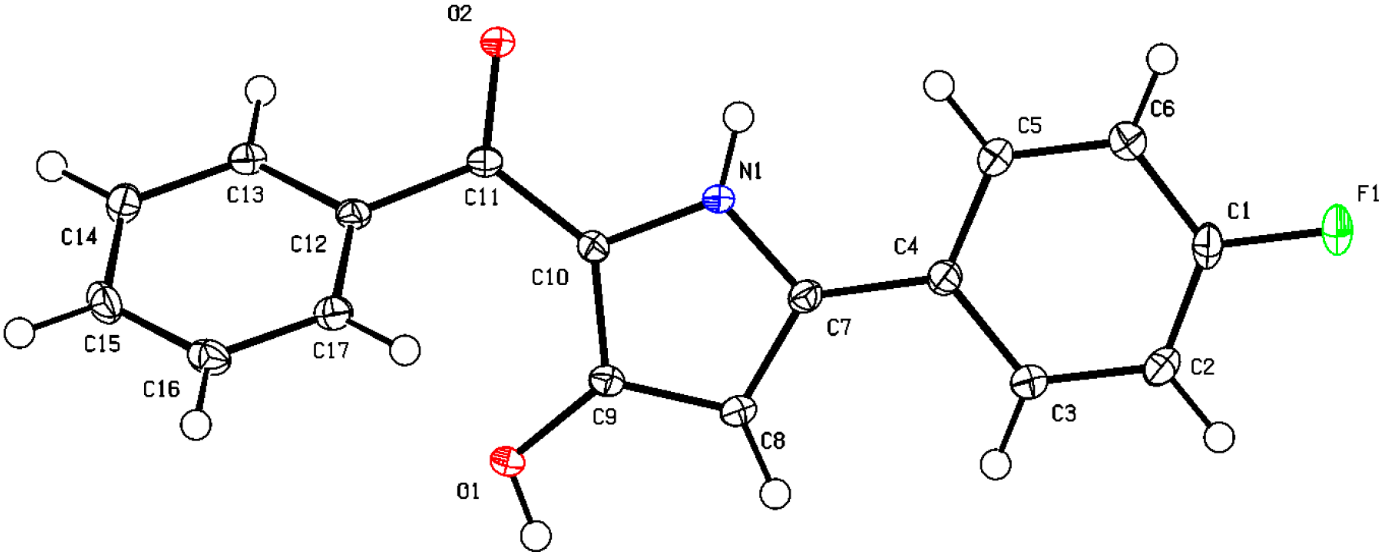

Figure S1. Molecular structure of compound **5a**, displacement parameters are drawn at 50% probability level.

Table S1. Crystal data and structure refinement for 5a.

Identification code	**5a** (TZ_AC)
Empirical formula	$C_{17}H_{12}NO_2F$
Formula weight	281.28
Temperature/K	100.01(10)
Crystal system	monoclinic
Space group	$P2_1/c$
a/Å	13.0439(3)
b/Å	11.4099(3)
c/Å	8.9076(2)
α/°	90
β/°	93.294(2)
γ/°	90
Volume/Å^3	1323.52(6)
Z	4
ρ_{calc}g/cm^3	1.412
μ/mm^{-1}	0.851
F(000)	584.0
Crystal size/mm^3	0.34 × 0.28 × 0.22
Radiation	Cu Kα (λ = 1.54184)
2Θ range for data collection/°	6.788 to 144.968
Index ranges	$-16 \leq h \leq 11$, $-12 \leq k \leq 14$, $-11 \leq l \leq 10$
Reflections collected	4999
Independent reflections	2597 [R_{int} = 0.0206, R_{sigma} = 0.0221]
Data/restraints/parameters	2597/0/191
Goodness-of-fit on F^2	1.027
Final R indexes [I>=2σ (I)]	R_1 = 0.0398, wR_2 = 0.1050
Final R indexes [all data]	R_1 = 0.0418, wR_2 = 0.1071
Largest diff. peak/hole / e Å^{-3}	0.24/-0.32

Table S2. Fractional atomic coordinates ($\times10^4$) and equivalent isotropic displacement parameters ($Å^2\times10^3$) for 5a. U_{eq} is defined as 1/3 of the trace of the orthogonalised U_{IJ} tensor.

**Atom**	***x***	***y***	***z***	**U(eq)**
F1	10767.1(6)	5323.5(8)	2877.1(10)	24.3(2)
O2	4053.7(6)	4438.3(8)	4105.4(10)	13.3(2)
O1	4965.8(7)	1055.5(8)	2403.1(11)	17.1(2)
N1	6076.6(8)	3762.6(9)	3535.6(12)	12.5(2)
C10	5240.8(9)	3002.6(11)	3475.2(14)	12.3(3)
C11	4265.9(9)	3370.1(11)	3950.2(13)	11.5(3)
C9	5568.4(9)	1984.2(11)	2739.0(14)	12.9(3)
C17	3768.3(10)	1418.4(11)	4985.6(14)	14.9(3)
C7	6890.1(9)	3249.2(11)	2928.1(14)	13.3(3)
C4	7904.6(9)	3803.1(11)	2931.3(14)	14.0(3)
C12	3484.7(9)	2476.8(11)	4295.9(13)	12.7(3)
C13	2445.3(9)	2730.7(11)	3987.7(14)	14.8(3)
C8	6588.5(9)	2145.9(11)	2392.7(14)	14.4(3)
C5	8098.2(10)	4885.3(12)	3616.4(15)	16.5(3)
C1	9824.5(10)	4816.9(13)	2903.8(15)	17.9(3)
C14	1704.9(10)	1924.6(12)	4354.0(15)	17.7(3)
C16	3023.3(10)	616.0(12)	5350.9(15)	17.5(3)
C6	9062.5(10)	5398.5(12)	3609.0(15)	17.8(3)
C3	8701.0(10)	3238.1(12)	2229.1(16)	19.3(3)
C15	1992.0(10)	867.7(12)	5034.7(15)	18.9(3)
C2	9669.5(10)	3745.5(12)	2215.4(17)	20.9(3)
F1	10767.1(6)	5323.5(8)	2877.1(10)	24.3(2)
O2	4053.7(6)	4438.3(8)	4105.4(10)	13.3(2)
O1	4965.8(7)	1055.5(8)	2403.1(11)	17.1(2)
N1	6076.6(8)	3762.6(9)	3535.6(12)	12.5(2)
C10	5240.8(9)	3002.6(11)	3475.2(14)	12.3(3)
C11	4265.9(9)	3370.1(11)	3950.2(13)	11.5(3)

Table S3. Anisotropic displacement parameters ($Å^2 \times 10^3$) for 5a. The anisotropic displacement factor exponent takes the form: $-2\pi^2[h^2a^{*2}U_{11}+2hka^*b^*U_{12}+\ldots]$.

Atom	U_{11}	U_{22}	U_{33}	U_{23}	U_{13}	U_{12}
F1	13.7(4)	26.8(5)	33.0(5)	-2.1(4)	6.4(3)	-6.9(3)
O2	13.1(4)	9.5(4)	17.7(4)	-0.8(3)	3.3(3)	0.6(3)
O1	14.9(4)	11.8(5)	25.0(5)	-7.6(4)	5.1(3)	-2.1(3)
N1	11.6(5)	8.6(5)	17.5(5)	-1.3(4)	2.9(4)	0.5(4)
C10	11.6(6)	10.1(6)	15.1(6)	0.7(4)	1.4(4)	-0.3(4)
C11	13.4(6)	10.6(6)	10.3(5)	0.0(4)	-0.1(4)	1.3(4)
C9	14.1(6)	10.4(6)	14.4(6)	0.4(4)	0.9(4)	0.8(4)
C17	16.5(6)	13.5(6)	14.9(6)	-0.6(5)	2.1(4)	1.3(5)
C7	12.5(6)	12.3(6)	15.3(6)	1.5(5)	2.5(4)	2.2(4)
C4	13.4(6)	12.8(6)	16.1(6)	2.6(5)	2.8(4)	1.0(4)
C12	14.8(6)	11.3(6)	12.3(5)	-2.8(4)	3.0(4)	-0.4(4)
C13	16.1(6)	12.2(6)	16.3(6)	-2.1(5)	2.6(5)	1.1(4)
C8	14.2(6)	12.0(6)	17.3(6)	-1.3(5)	3.5(4)	2.2(4)
C5	13.5(6)	17.1(6)	19.2(6)	-0.8(5)	3.7(5)	1.1(5)
C1	11.0(6)	21.0(7)	21.9(6)	3.8(5)	2.8(5)	-2.7(5)
C14	12.9(6)	19.8(7)	20.7(6)	-3.9(5)	2.9(5)	-1.4(5)
C16	24.3(7)	12.1(6)	16.4(6)	1.5(5)	4.2(5)	-0.6(5)
C6	16.5(6)	16.7(6)	20.2(6)	-2.1(5)	1.9(5)	-1.8(5)
C3	16.4(6)	13.4(6)	28.8(7)	-1.4(5)	6.5(5)	0.8(5)
C15	21.3(6)	17.5(6)	18.5(6)	-2.6(5)	7.4(5)	-7.9(5)
C2	15.1(6)	18.5(7)	29.9(7)	0.5(6)	8.6(5)	2.9(5)
F1	13.7(4)	26.8(5)	33.0(5)	-2.1(4)	6.4(3)	-6.9(3)
O2	13.1(4)	9.5(4)	17.7(4)	-0.8(3)	3.3(3)	0.6(3)
O1	14.9(4)	11.8(5)	25.0(5)	-7.6(4)	5.1(3)	-2.1(3)
N1	11.6(5)	8.6(5)	17.5(5)	-1.3(4)	2.9(4)	0.5(4)
C10	11.6(6)	10.1(6)	15.1(6)	0.7(4)	1.4(4)	-0.3(4)
C11	13.4(6)	10.6(6)	10.3(5)	0.0(4)	-0.1(4)	1.3(4)

Table S4. Bond lengths for 5a.

Atom	Atom	Length/Å	Atom	Atom	Length/Å
F1	C1	1.3601(15)	C7	C8	1.3947(18)
O2	C11	1.2592(15)	C4	C5	1.3940(18)
O1	C9	1.3428(15)	C4	C3	1.4003(18)
N1	C10	1.3917(15)	C12	C13	1.3981(17)
N1	C7	1.3520(16)	C13	C14	1.3861(18)
C10	C11	1.4262(16)	C5	C6	1.3878(18)
C10	C9	1.4124(17)	C1	C6	1.3765(19)
C11	C12	1.4860(17)	C1	C2	1.377(2)
C9	C8	1.3954(17)	C14	C15	1.391(2)
C17	C12	1.3953(18)	C16	C15	1.388(2)
C17	C16	1.3873(18)	C3	C2	1.3903(19)
C7	C4	1.4664(17)			

Table S5. Bond angles for 5a.

Atom	Atom	Atom	Angle/°	Atom	Atom	Atom	Angle/°
C7	N1	C10	110.22(10)	C3	C4	C7	119.56(12)
N1	C10	C11	121.01(11)	C17	C12	C11	121.15(11)
N1	C10	C9	105.87(10)	C17	C12	C13	119.70(11)
C9	C10	C11	132.71(11)	C13	C12	C11	119.08(11)
O2	C11	C10	121.51(11)	C14	C13	C12	119.82(12)
O2	C11	C12	118.91(10)	C7	C8	C9	107.31(11)
C10	C11	C12	119.58(11)	C6	C5	C4	120.87(12)
O1	C9	C10	124.32(11)	F1	C1	C6	118.71(12)
O1	C9	C8	127.48(11)	F1	C1	C2	118.63(12)
C8	C9	C10	108.16(11)	C6	C1	C2	122.66(12)
C16	C17	C12	120.19(12)	C13	C14	C15	120.26(12)
N1	C7	C4	122.78(11)	C17	C16	C15	119.96(12)
N1	C7	C8	108.40(11)	C1	C6	C5	118.50(12)
C8	C7	C4	128.78(11)	C2	C3	C4	120.66(13)
C5	C4	C7	121.56(11)	C16	C15	C14	120.07(12)
C5	C4	C3	118.88(12)	C1	C2	C3	118.42(12)

Table S6. Torsion angles for 5a.

A	B	C	D	Angle/°	A	B	C	D	Angle/°
F1	C1	C6	C5	179.18(11)	C9	C10	C11	C12	26.2(2)
F1	C1	C2	C3	-179.20(12)	C17	C12	C13	C14	-0.70(18)
O2	C11	C12	C17	-142.43(12)	C17	C16	C15	C14	-0.1(2)
O2	C11	C12	C13	34.46(16)	C7	N1	C10	C11	-175.53(11)
O1	C9	C8	C7	178.47(12)	C7	N1	C10	C9	-1.99(14)
N1	C10	C11	O2	16.69(18)	C7	C4	C5	C6	-179.95(12)
N1	C10	C11	C12	-162.31(11)	C7	C4	C3	C2	179.94(12)
N1	C10	C9	O1	-177.11(11)	C4	C7	C8	C9	175.76(12)
N1	C10	C9	C8	0.74(14)	C4	C5	C6	C1	0.2(2)
N1	C7	C4	C5	2.3(2)	C4	C3	C2	C1	-0.2(2)
N1	C7	C4	C3	-177.54(12)	C12	C17	C16	C15	-0.23(19)
N1	C7	C8	C9	-1.95(14)	C12	C13	C14	C15	0.37(19)
C10	N1	C7	C4	-175.40(11)	C13	C14	C15	C16	0.0(2)
C10	N1	C7	C8	2.48(14)	C8	C7	C4	C5	-175.09(12)
C10	C11	C12	C17	36.59(16)	C8	C7	C4	C3	5.0(2)
C10	C11	C12	C13	-146.52(12)	C5	C4	C3	C2	0.1(2)
C10	C9	C8	C7	0.71(14)	C16	C17	C12	C11	177.50(11)
C11	C10	C9	O1	-4.6(2)	C16	C17	C12	C13	0.64(18)
C11	C10	C9	C8	173.20(13)	C6	C1	C2	C3	0.4(2)
C11	C12	C13	C14	-177.64(11)	C3	C4	C5	C6	-0.1(2)
C9	C10	C11	O2	-154.84(13)	C2	C1	C6	C5	-0.4(2)

Table S7. Hydrogen atom coordinates (Å × 10^4) and isotropic displacement parameters (Å^2×10^3) for 5a.

**Atom**	***x***	***y***	***z***	**U(eq)**
H1	5295.8	559.97	1927.25	26
H1A	6075.76	4474.87	3914.61	15
H17	4473.64	1246.64	5205.79	18
H13	2247.01	3453.62	3528.47	18
H8	7001.21	1604.82	1886.03	17
H5	7562.96	5276.62	4094.79	20
H14	998.64	2094.47	4139.58	21
H16	3218.67	-105.29	5817.1	21
H6	9193.65	6134.66	4079.9	21
H3	8578.4	2500.58	1757.32	23
H15	1481.93	317.73	5283.31	23
H2	10211.47	3362.36	1742.5	25

7. ^{1}H, ^{13}C and DEPT spectra of new compounds

(1*RR*,3*SR*,6*RR*)-4-Diazo-3-(4-iodophenyl)-1-(3-methoxyphenyl)-2-oxa-7-azabicyclo[4.1.0]heptan-5-one (4b), ^{1}H NMR, C_6D_6, 400 MHz

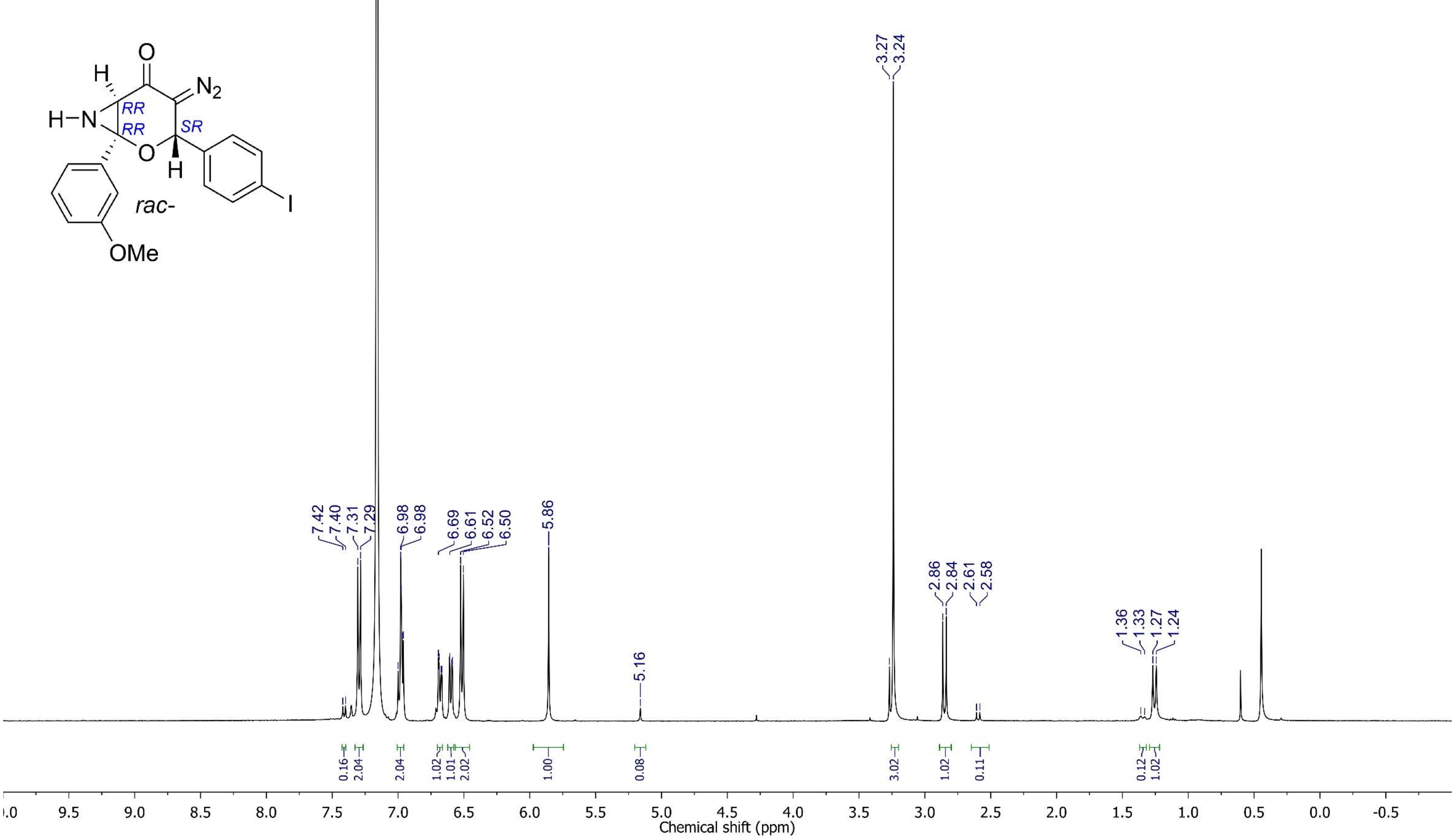

(1*RR*,3*SR*,6*RR*)-4-Diazo-3-(4-iodophenyl)-1-(3-methoxyphenyl)-2-oxa-7-azabicyclo[4.1.0]heptan-5-one (4b), $^{13}C\{^1H\}$ NMR, C_6D_6, 100 MHz

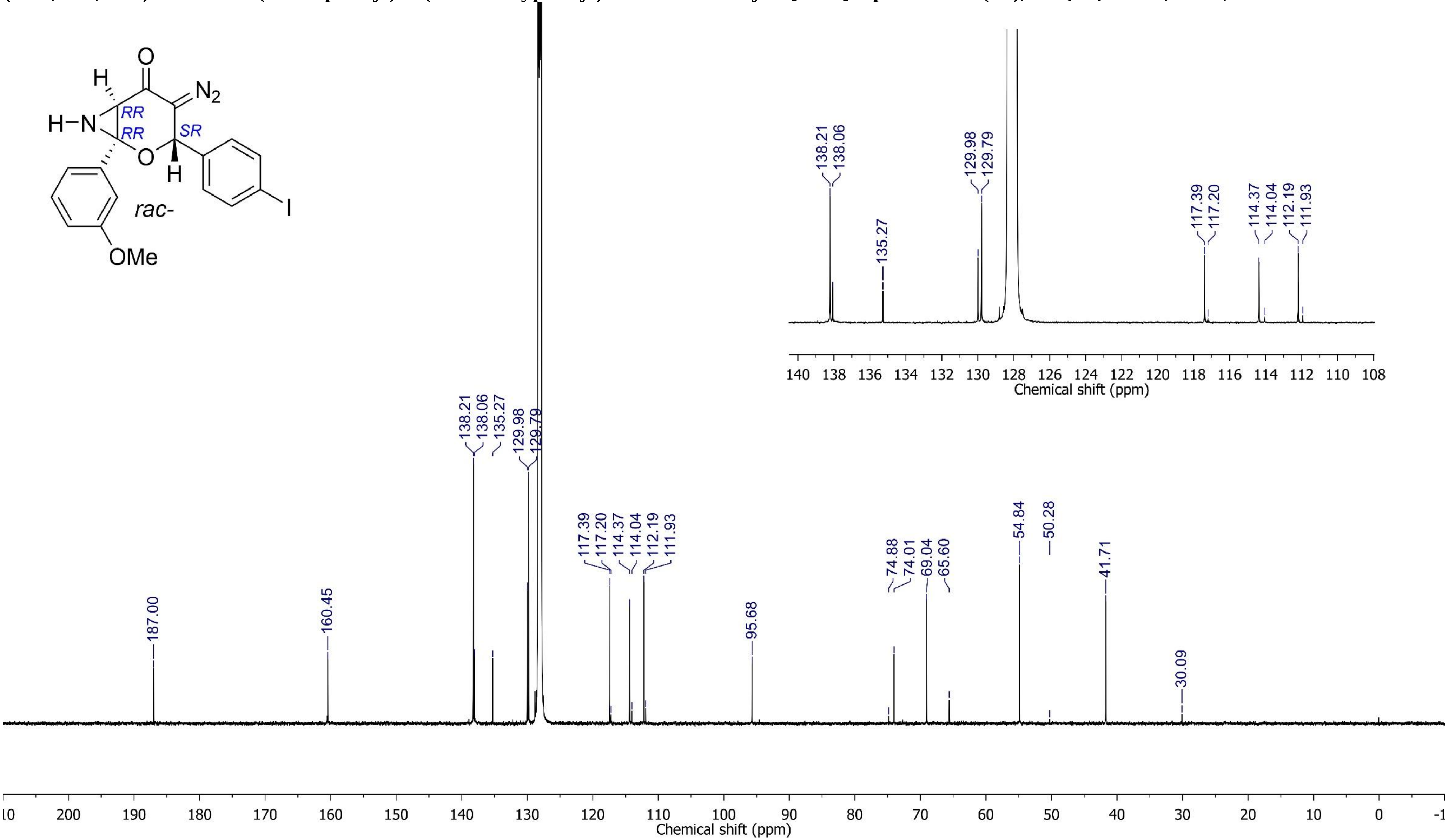

(1*RR*,3*SR*,6*RR*)-4-Diazo-3-(4-iodophenyl)-1-(3-methoxyphenyl)-2-oxa-7-azabicyclo[4.1.0]heptan-5-one (4b), DEPT, C_6D_6, 100 MHz

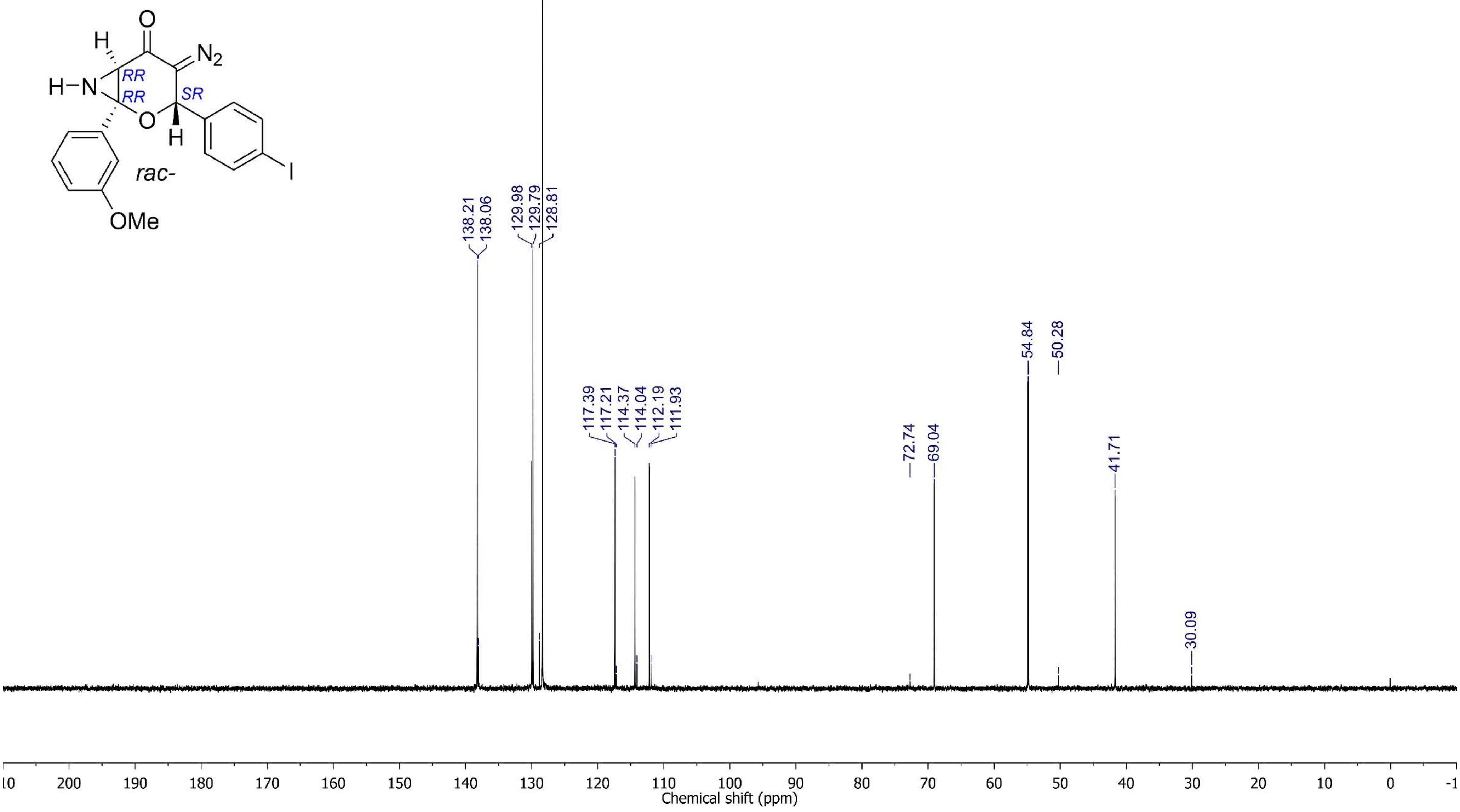

(1*RR*,3*SR*,6*RR*)-4-Diazo-3-(4-iodophenyl)-1-(3-methoxyphenyl)-2-oxa-7-azabicyclo[4.1.0]heptan-5-one (4b), ^{1}H 2D-NOESY, C_6D_6, 400 MHz

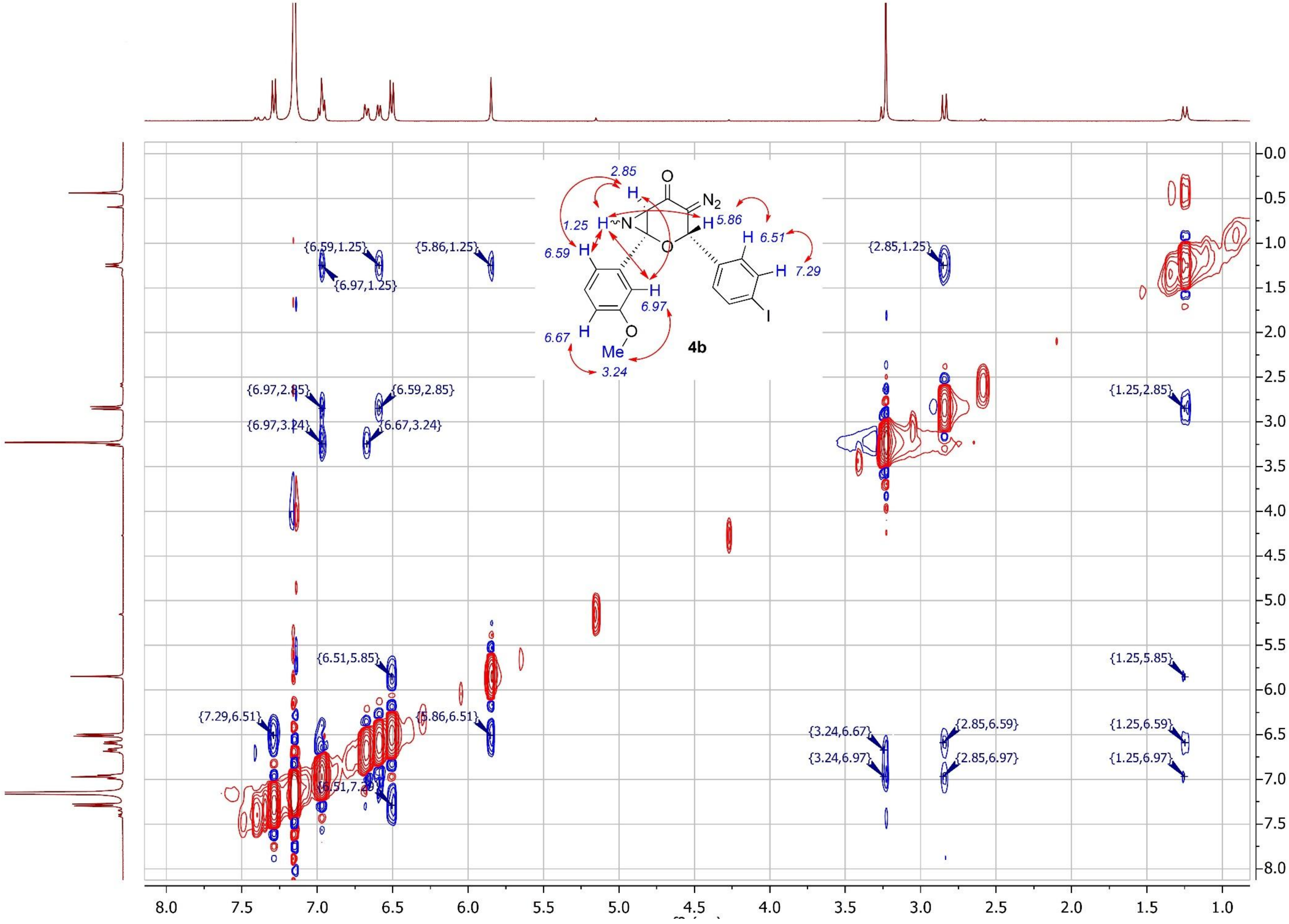

(5-(4-Fluorophenyl)-3-hydroxy-1*H*-pyrrol-2-yl)(phenyl)methanone (5a), ^{1}H NMR, $CDCl_3$, 400 MHz

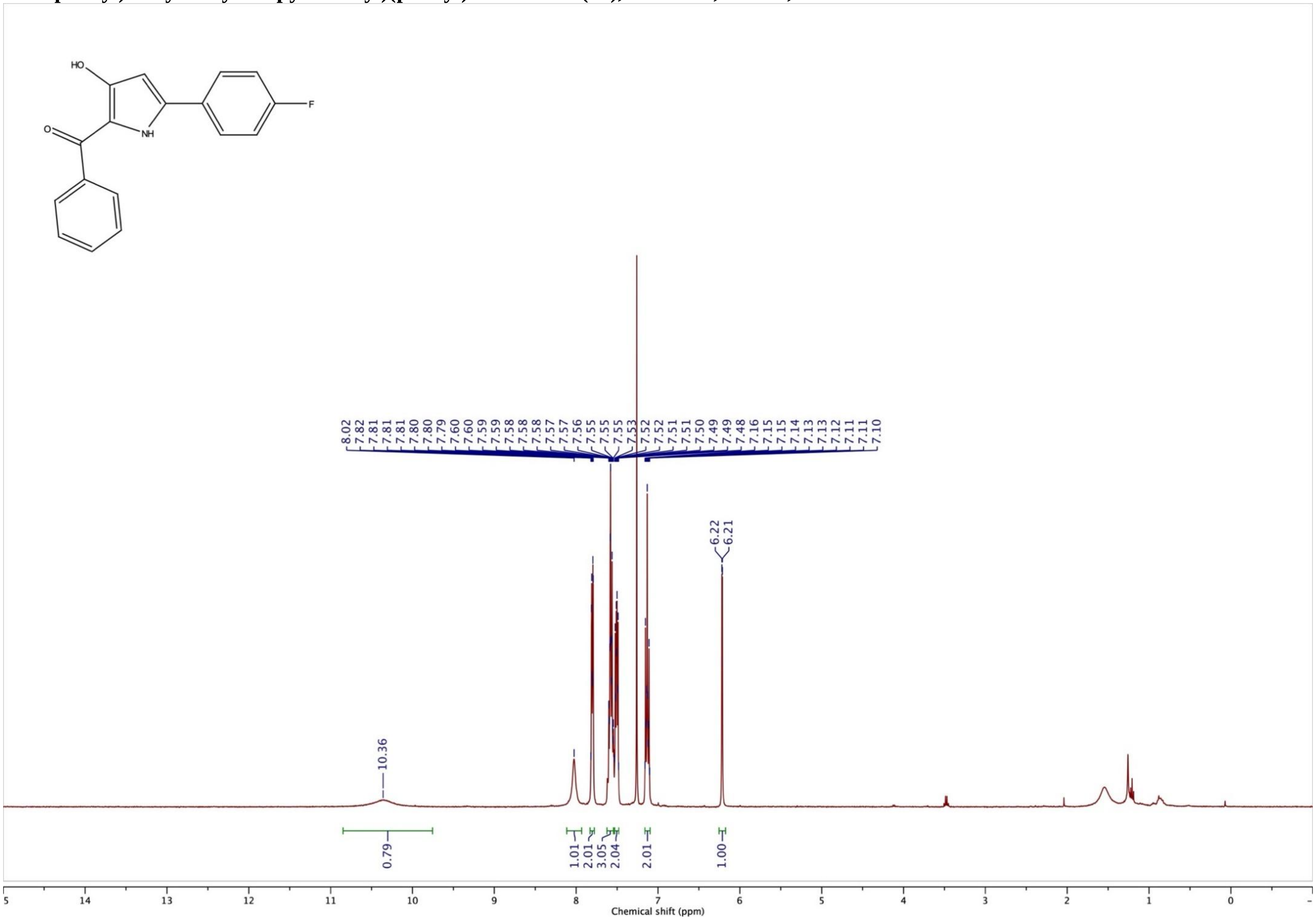

(5-(4-Fluorophenyl)-3-hydroxy-1*H*-pyrrol-2-yl)(phenyl)methanone (5a), $^{13}C\{^1H\}$ NMR, $CDCl_3$, 100 MHz

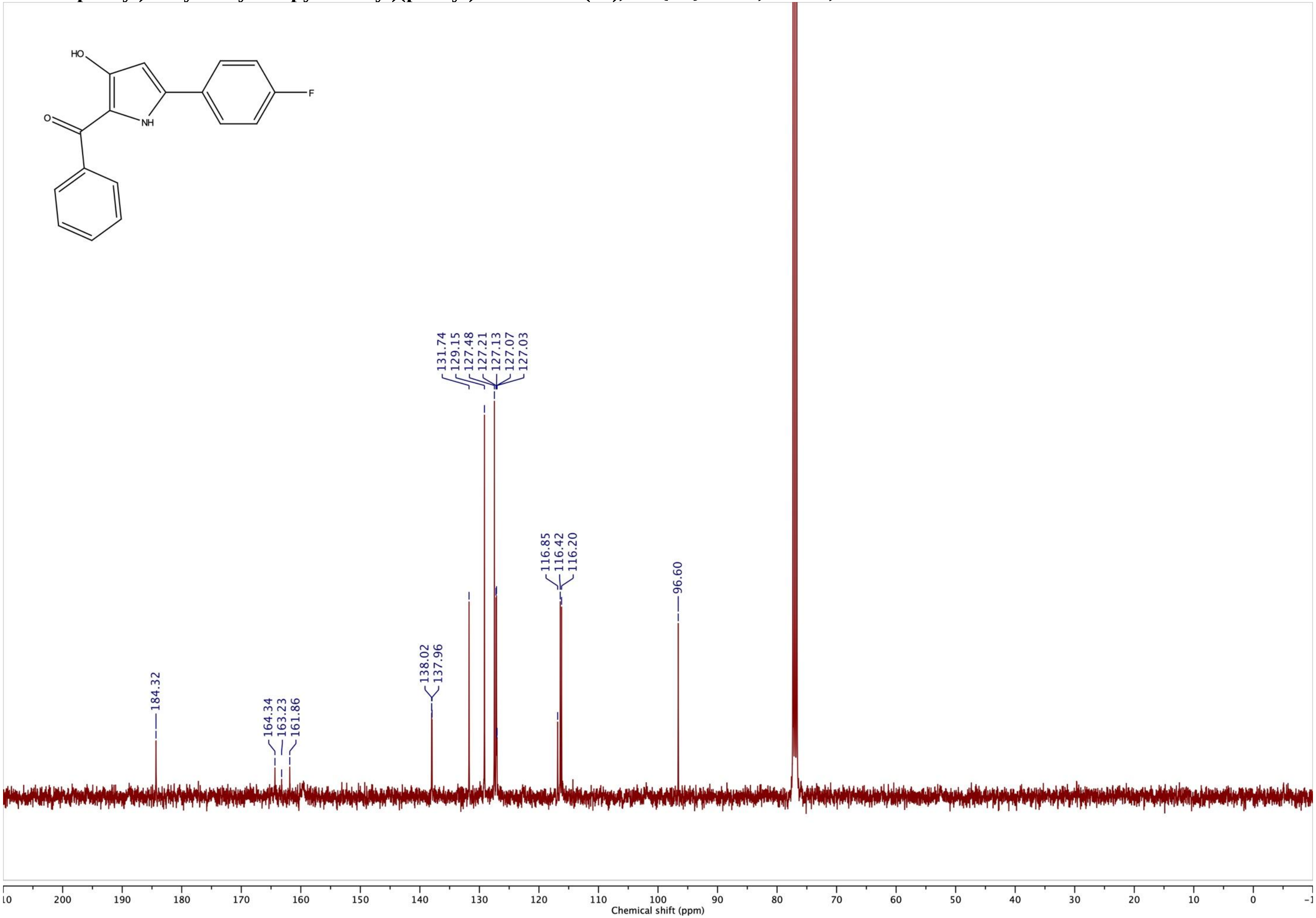

(5-(4-Fluorophenyl)-3-hydroxy-1*H*-pyrrol-2-yl)(phenyl)methanone (5a), DEPT, $CDCl_3$, 100 MHz

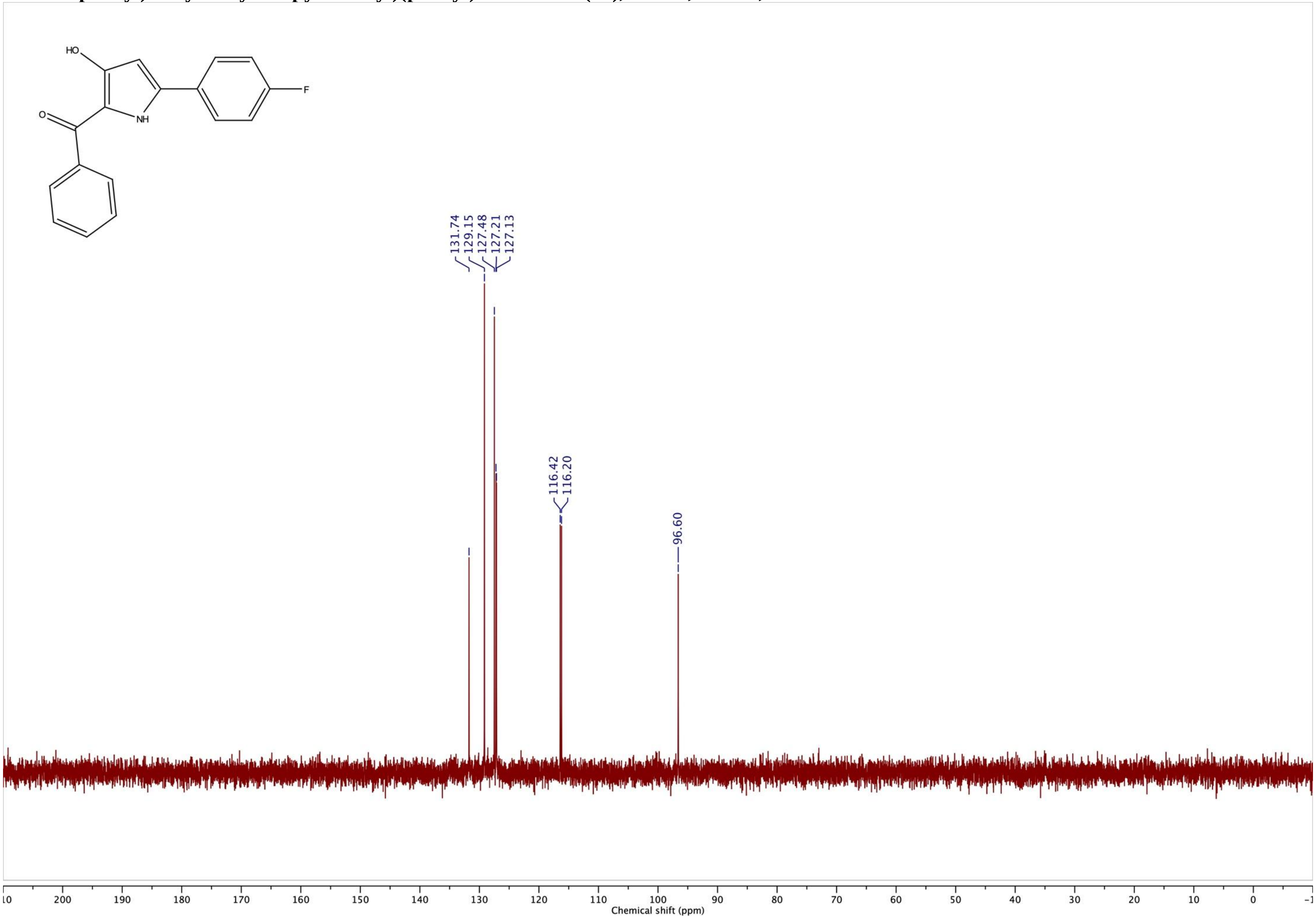

(3-Hydroxy-5-(4-iodophenyl)-1*H*-pyrrol-2-yl)(3-methoxyphenyl)methanone (5b), ^{1}H NMR, $CDCl_3$, 400 MHz

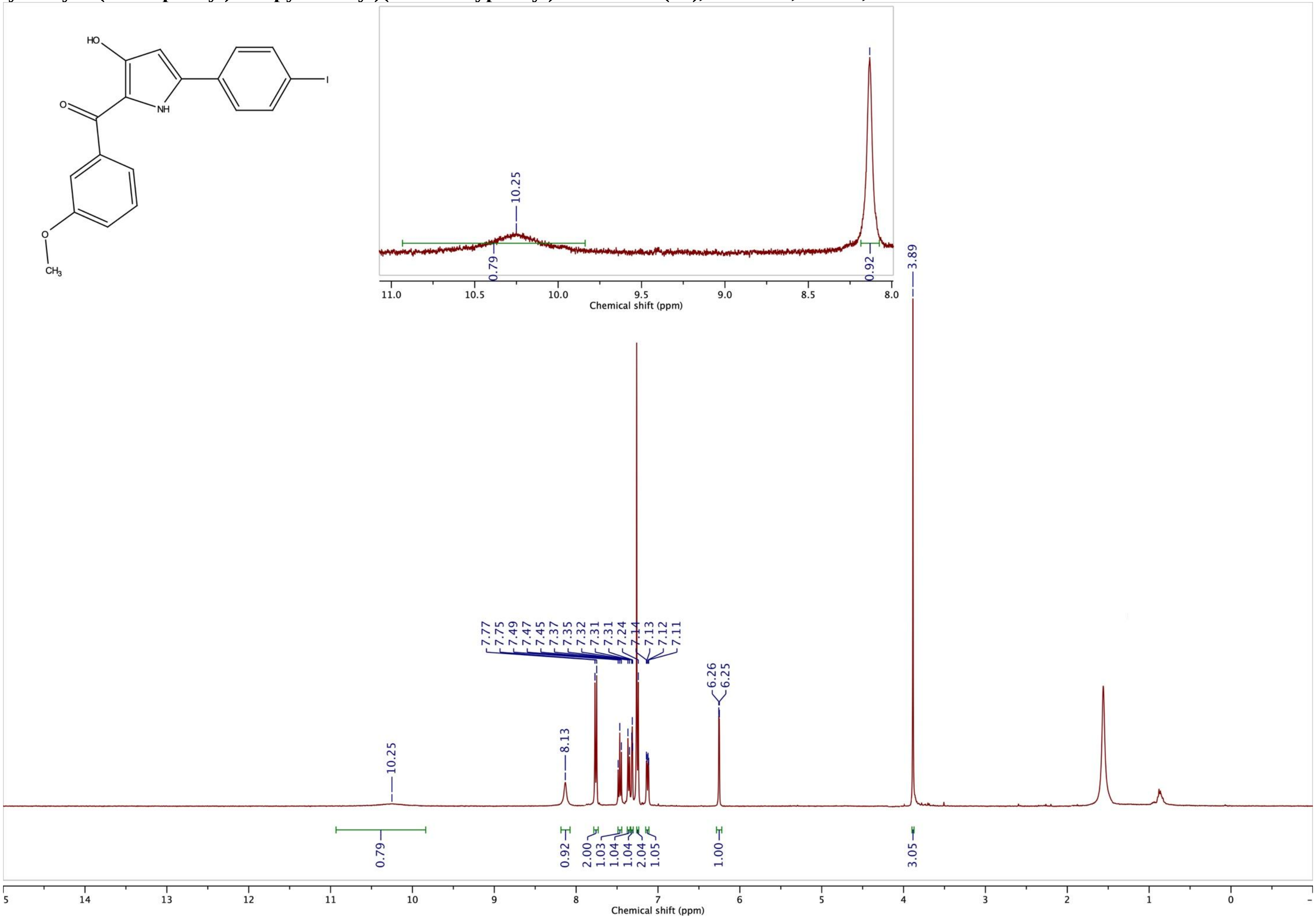

(3-Hydroxy-5-(4-iodophenyl)-1*H*-pyrrol-2-yl)(3-methoxyphenyl)methanone (5b), $^{13}C\{^{1}H\}$ NMR, $CDCl_3$, 100 MHz

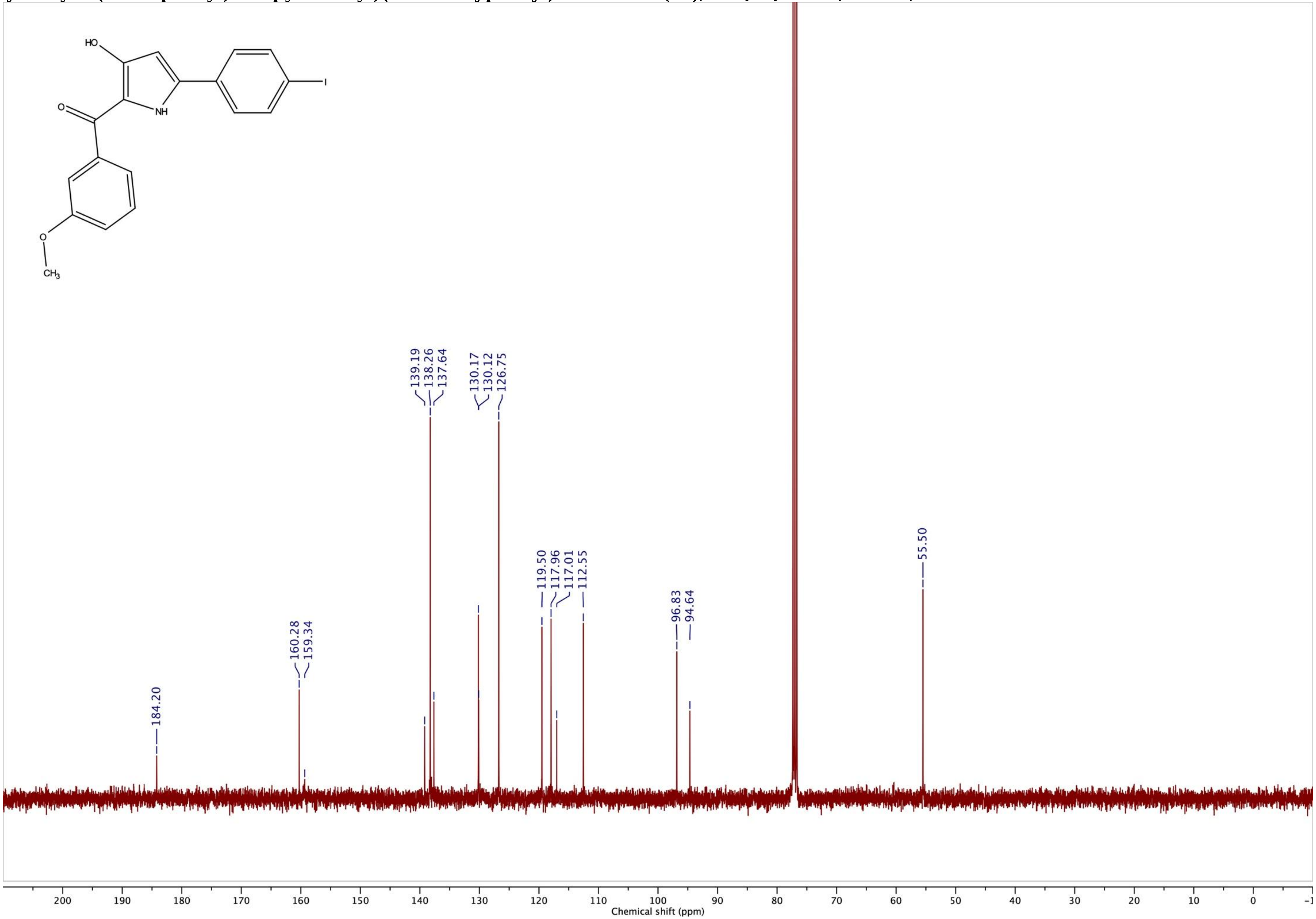

(3-Hydroxy-5-(4-iodophenyl)-1*H*-pyrrol-2-yl)(3-methoxyphenyl)methanone (5b), DEPT, $CDCl_3$, 100 MHz

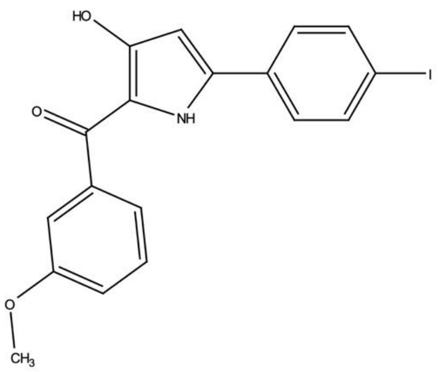

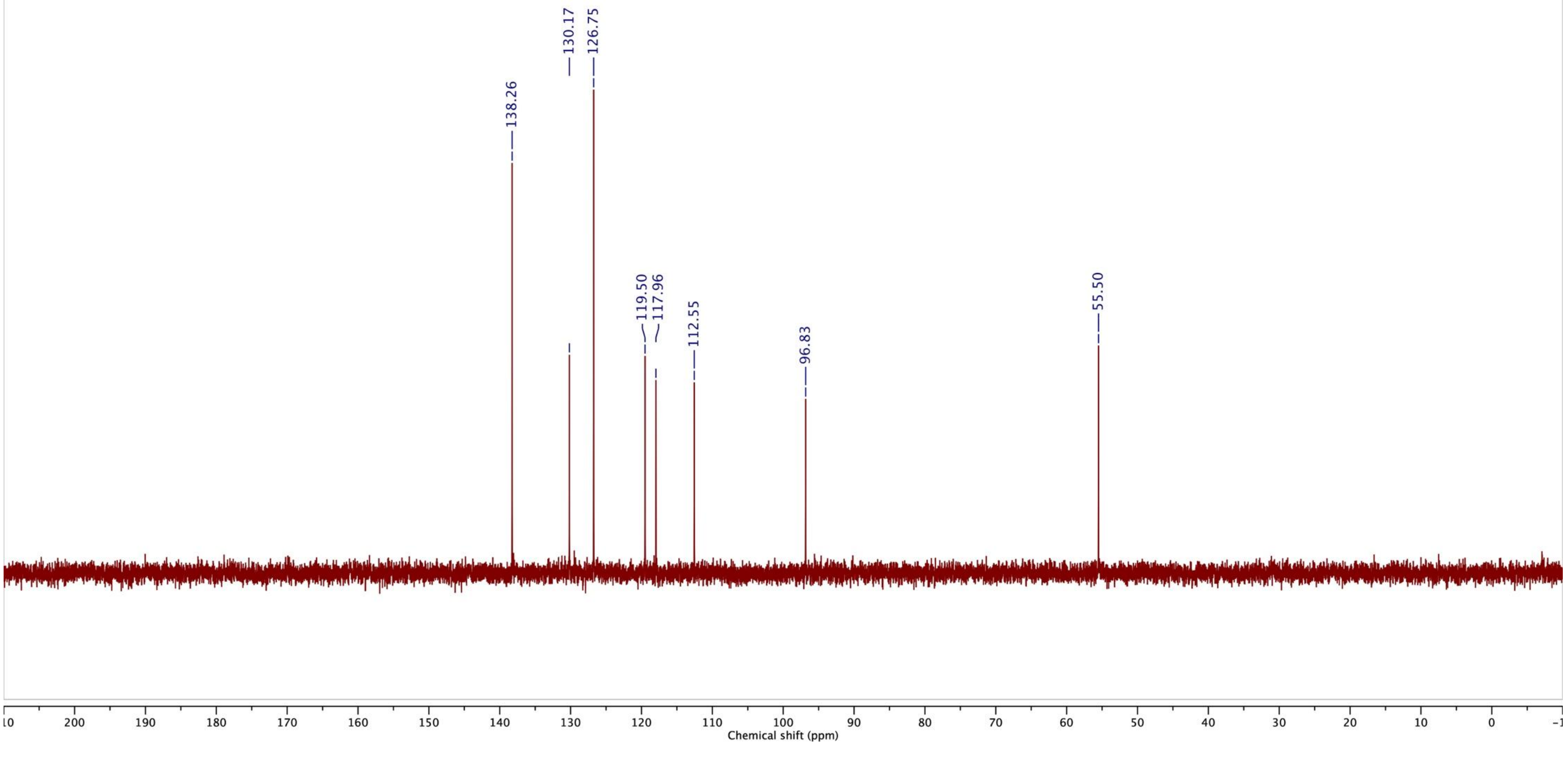

(3-Hydroxy-5-(naphthalen-1-yl)-1*H*-pyrrol-2-yl)(4-methoxyphenyl)methanone (5c), ^{1}H NMR, $CDCl_3$, 400 MHz

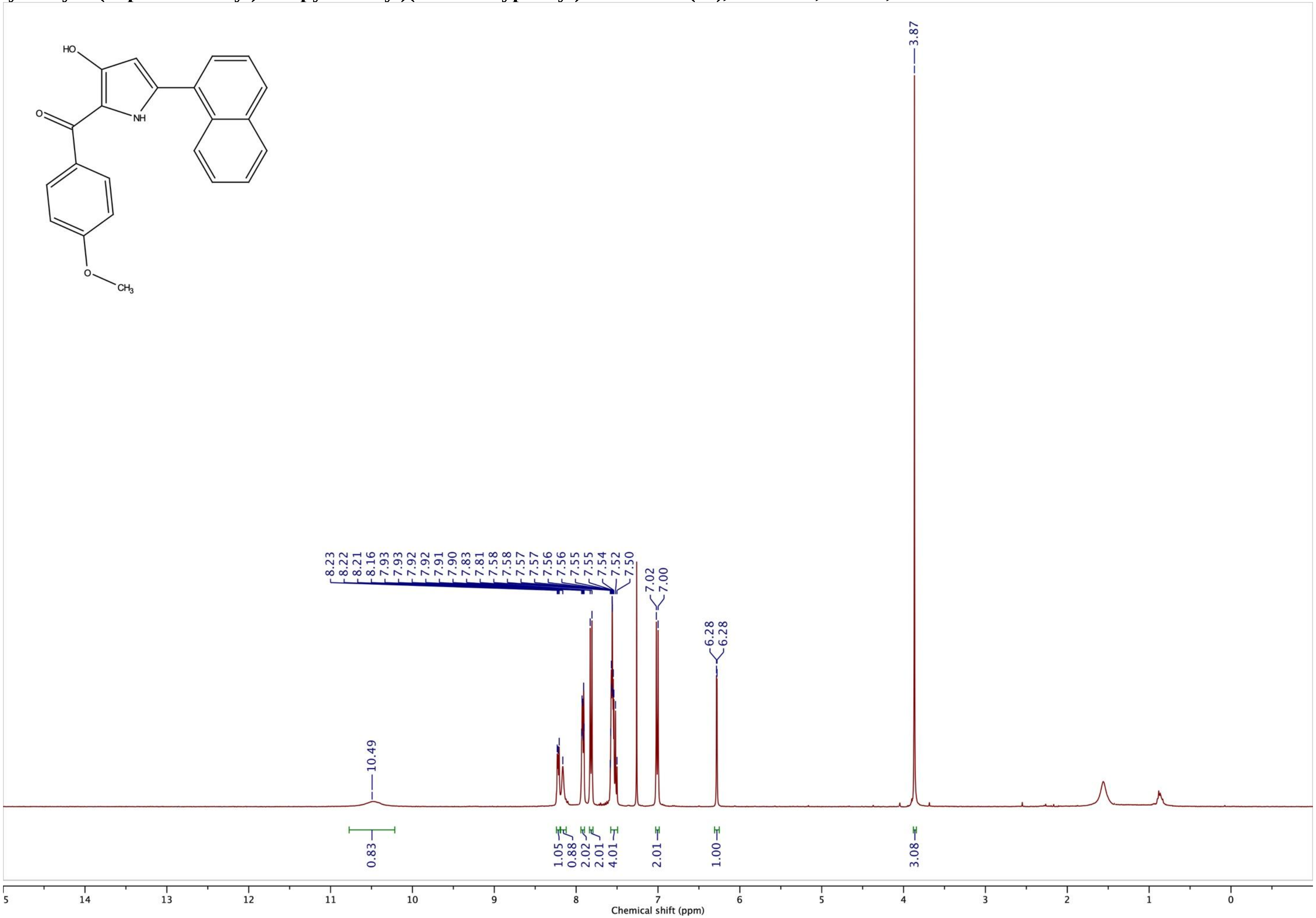

(3-Hydroxy-5-(naphthalen-1-yl)-1*H*-pyrrol-2-yl)(4-methoxyphenyl)methanone (5c), $^{13}C\{^{1}H\}$ NMR, $CDCl_3$, 100 MHz

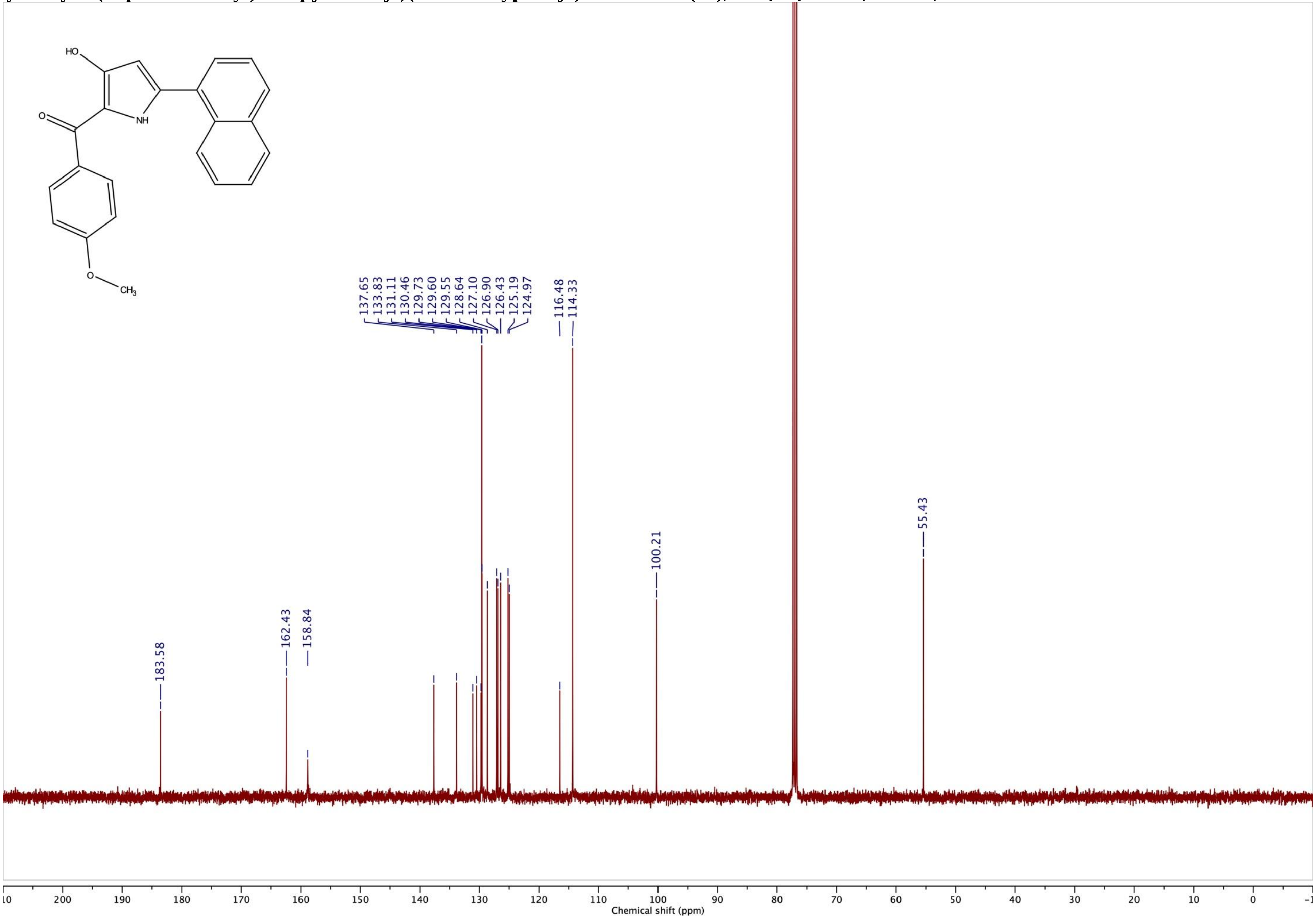

(3-Hydroxy-5-(naphthalen-1-yl)-1*H*-pyrrol-2-yl)(4-methoxyphenyl)methanone (5c), DEPT, $CDCl_3$, 100 MHz

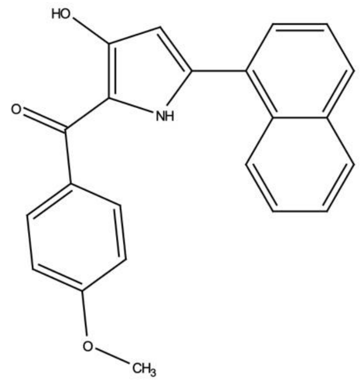

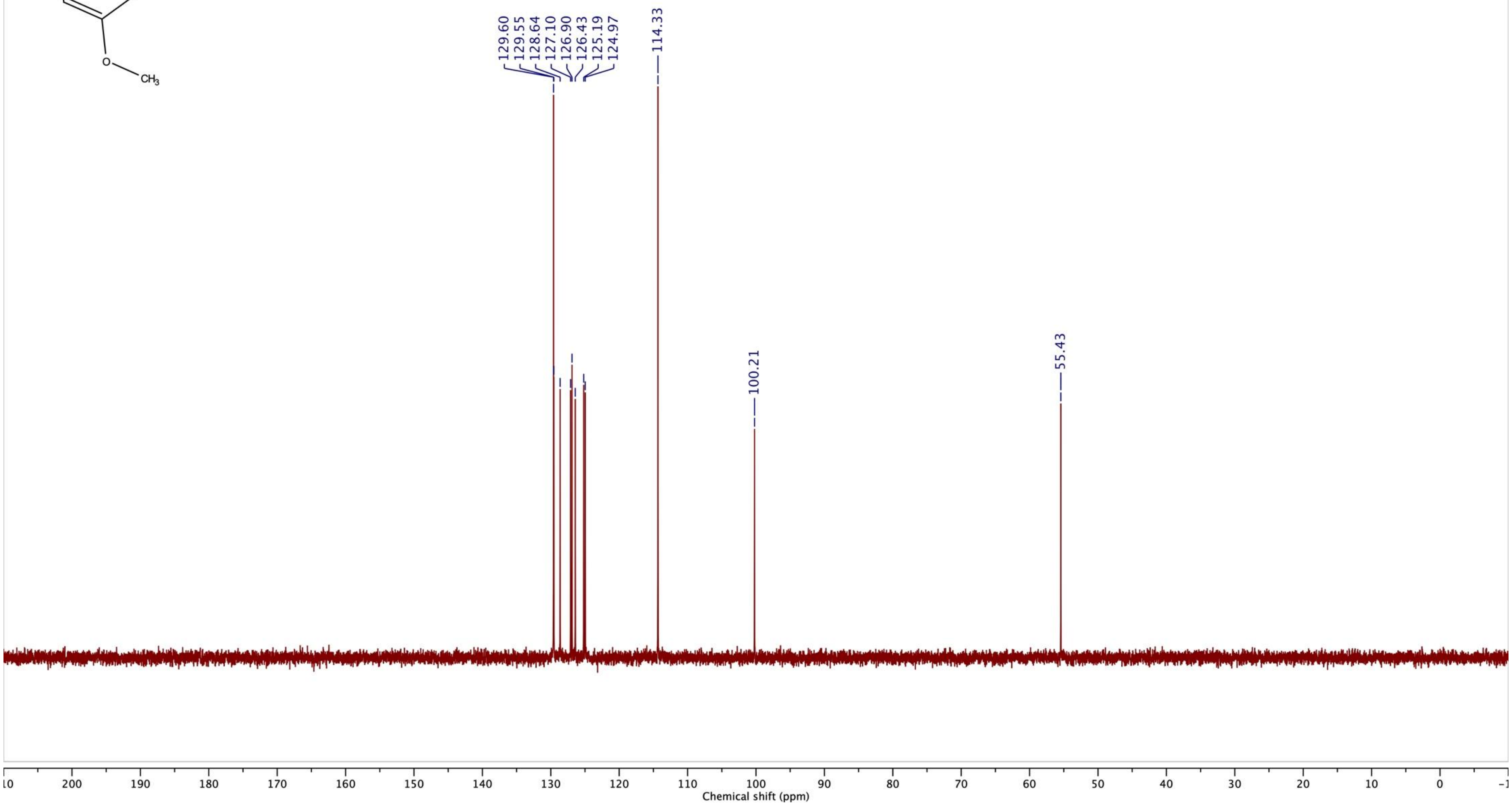

(3-Hydroxy-5-(thiophen-2-yl)-1*H*-pyrrol-2-yl)(4-methoxyphenyl)methanone (5d), ^{1}H NMR, $CDCl_3$, 400 MHz

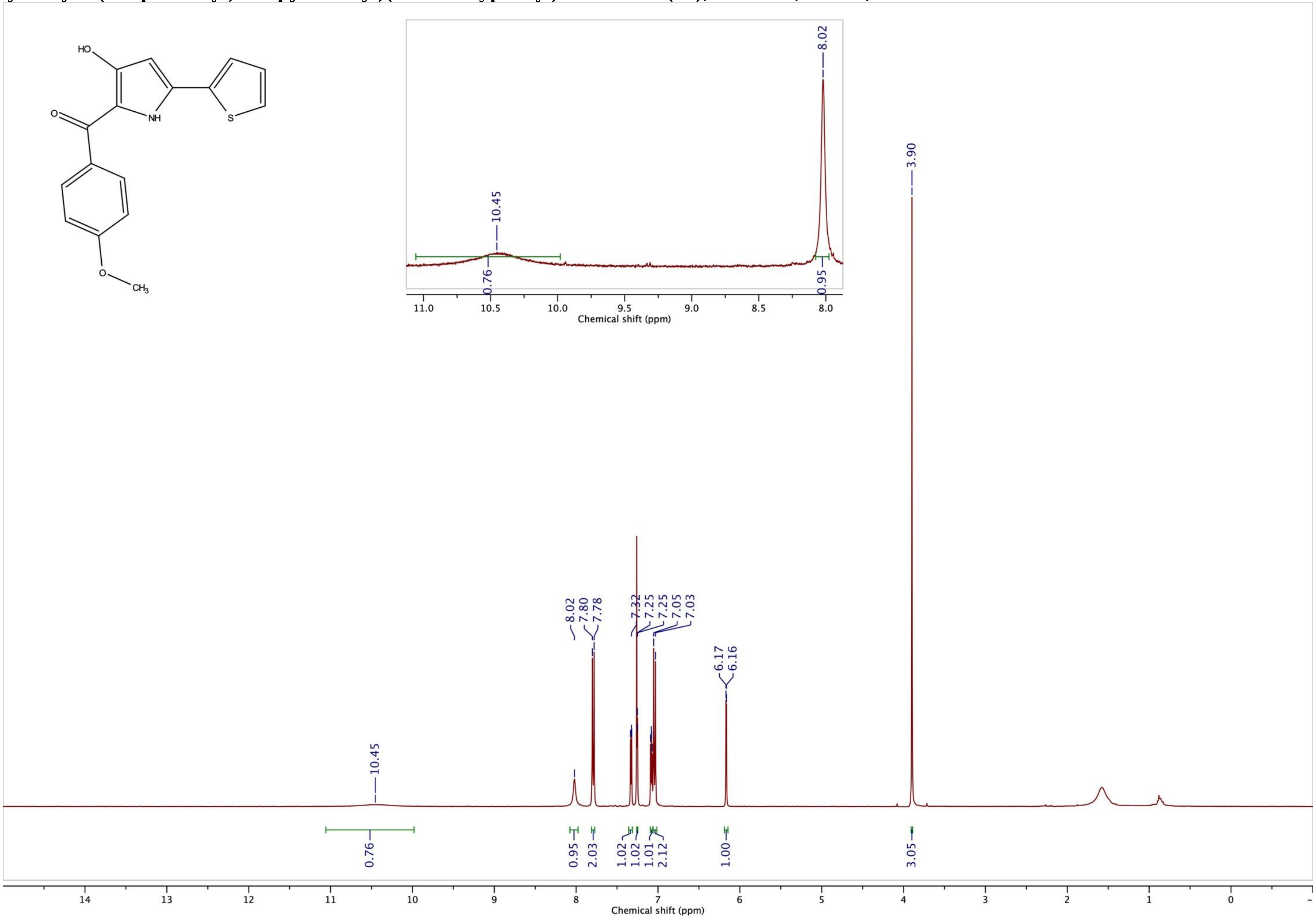

(3-Hydroxy-5-(thiophen-2-yl)-1*H*-pyrrol-2-yl)(4-methoxyphenyl)methanone (5d), $^{13}C\{^{1}H\}$ NMR, $CDCl_3$, 100 MHz

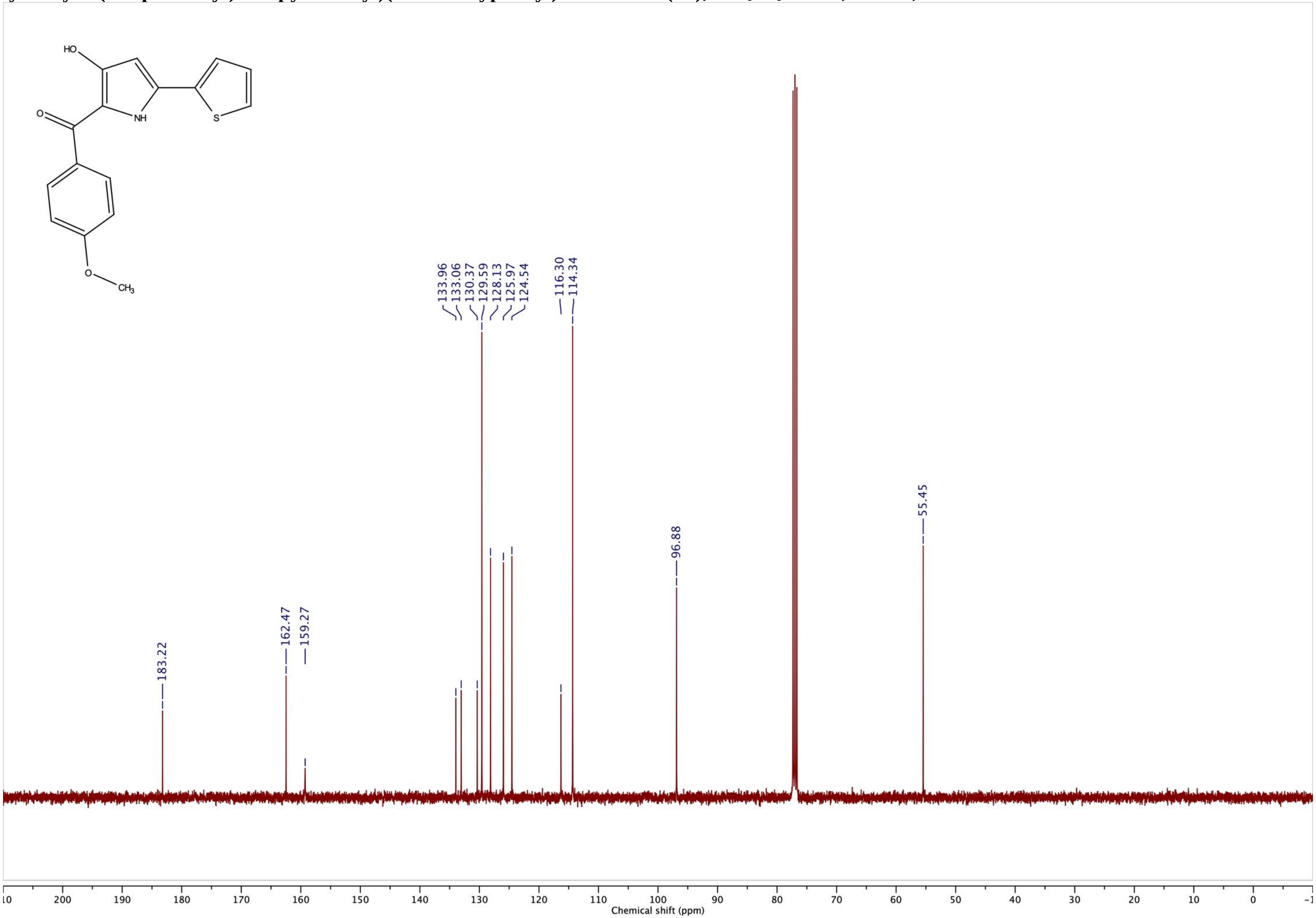

(3-Hydroxy-5-(thiophen-2-yl)-1*H*-pyrrol-2-yl)(4-methoxyphenyl)methanone (5d), DEPT, $CDCl_3$, 100 MHz

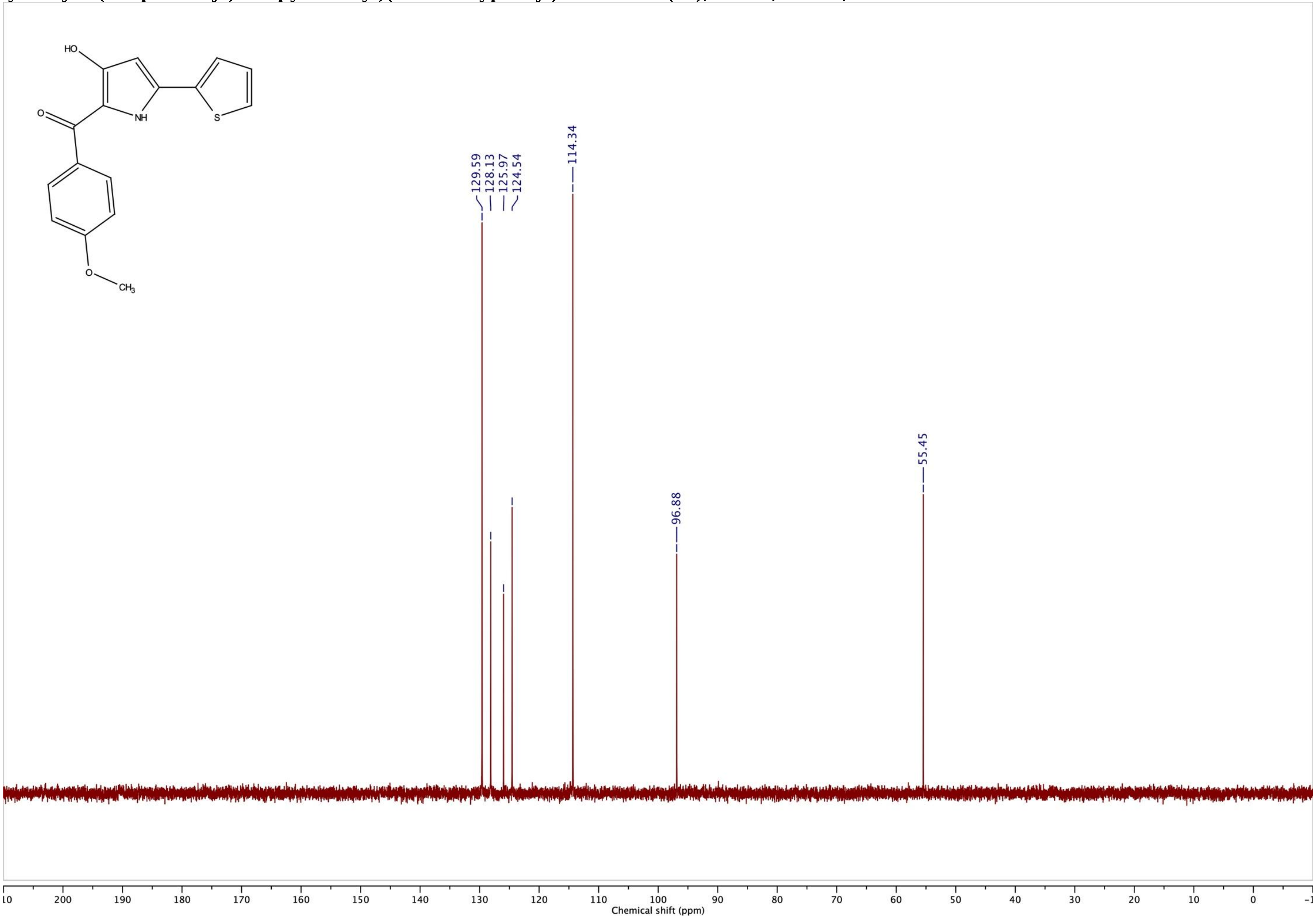

(5-(4-Chlorophenyl)-3-hydroxy-1*H*-pyrrol-2-yl)(3,4-dimethoxyphenyl)methanone (5e), ^{1}H NMR, DMSO-*d*$_6$, 400 MHz

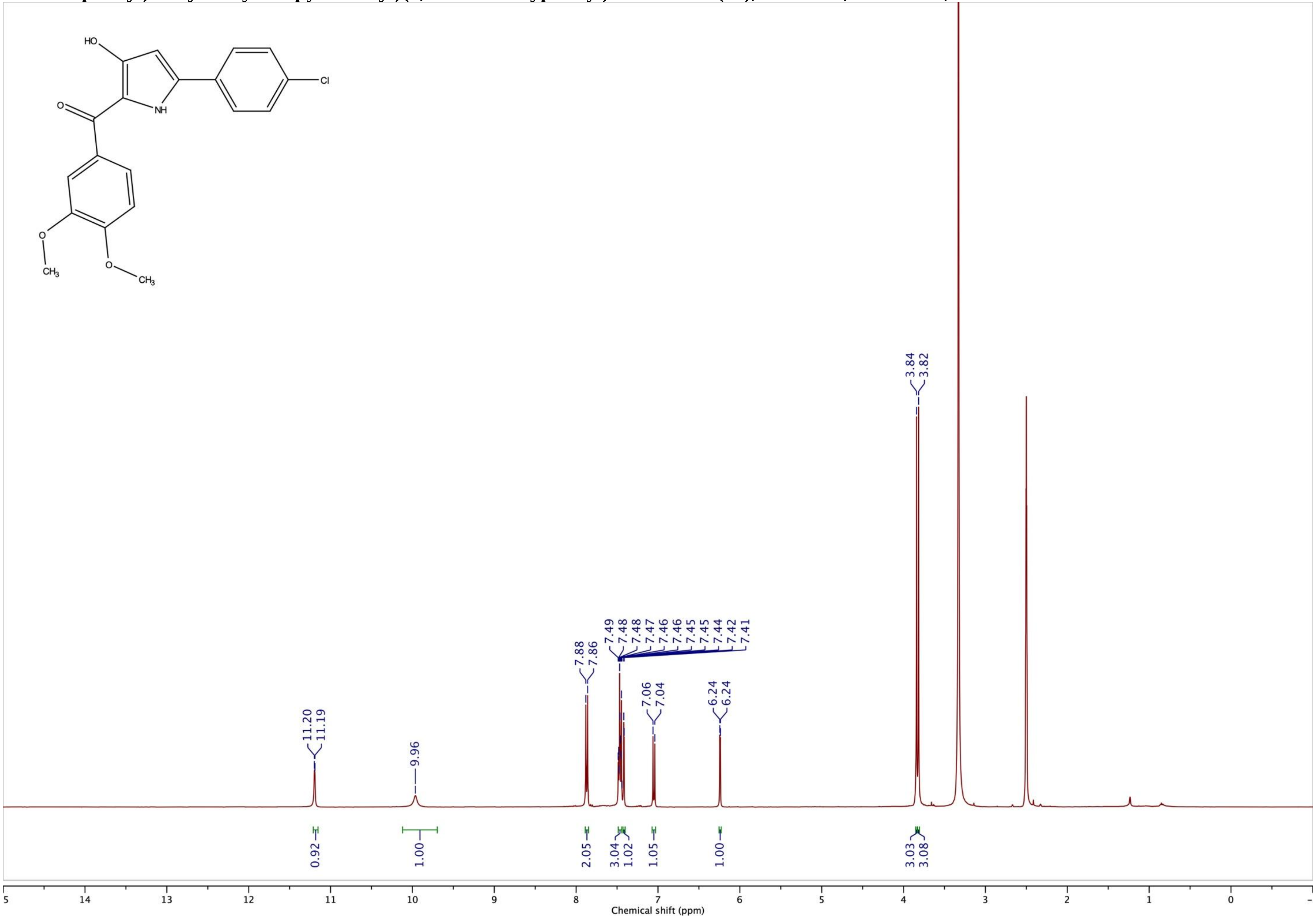

(5-(4-Chlorophenyl)-3-hydroxy-1*H*-pyrrol-2-yl)(3,4-dimethoxyphenyl)methanone (5e), $^{13}C\{^1H\}$ NMR, DMSO-*d*$_6$, 100 MHz

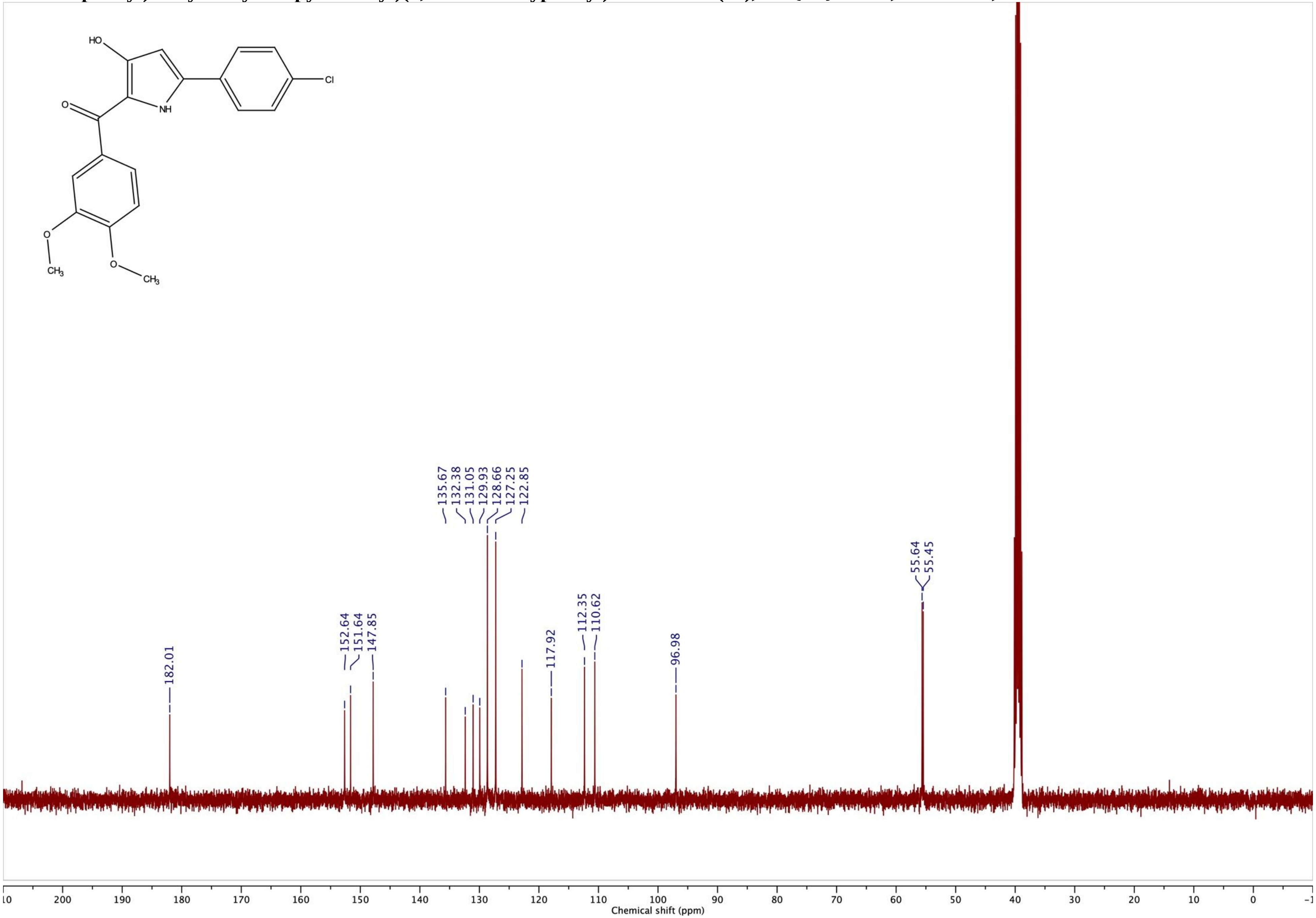

(5-(4-Chlorophenyl)-3-hydroxy-1*H*-pyrrol-2-yl)(3,4-dimethoxyphenyl)methanone (5e), DEPT, DMSO-*d*$_6$, 100 MHz

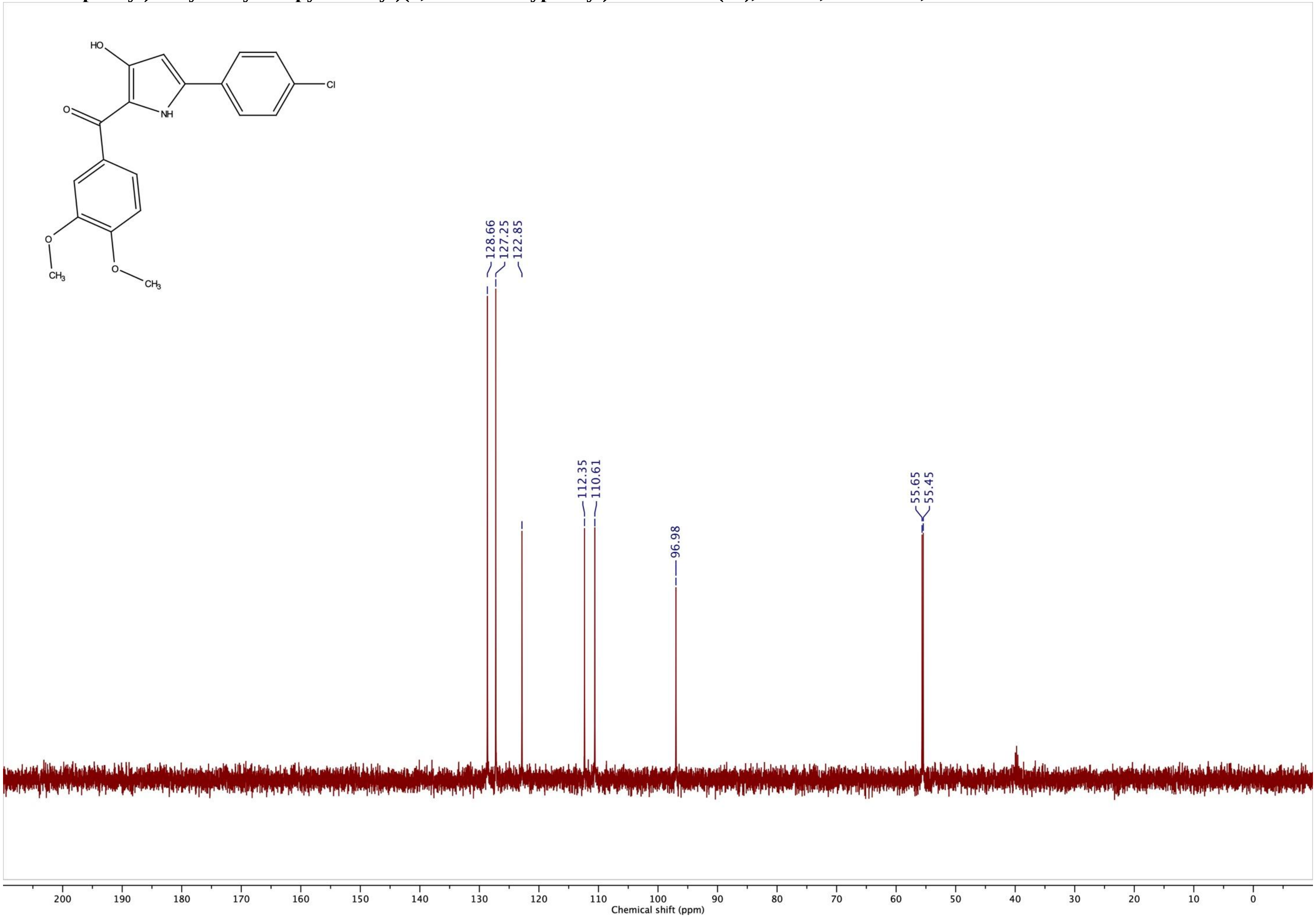

(3-Hydroxy-5-(*p*-tolyl)-1*H*-pyrrol-2-yl)(phenyl)methanone (5f), ^{1}H NMR, CDCl$_3$, 400 MHz

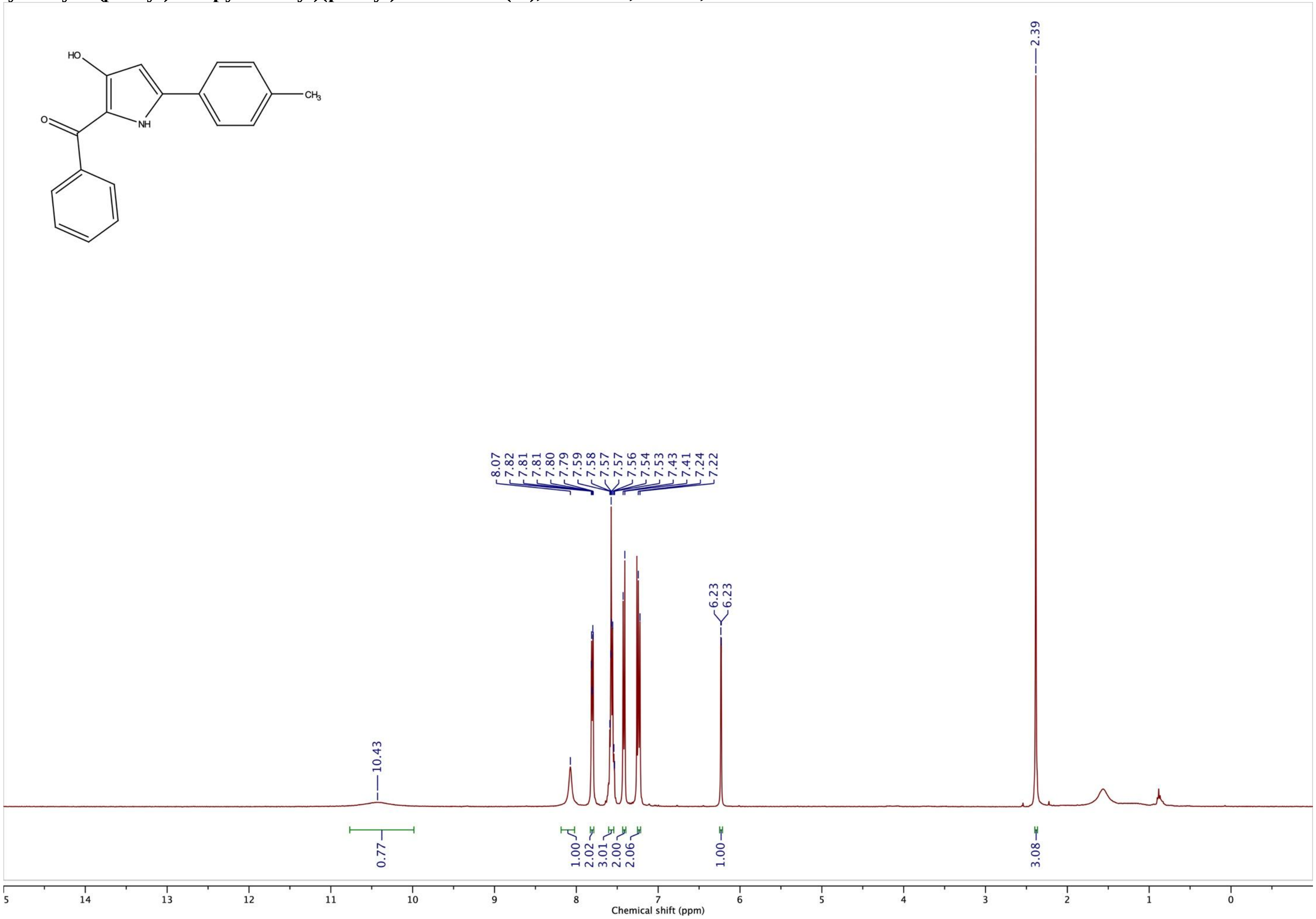

(3-Hydroxy-5-(*p*-tolyl)-1*H*-pyrrol-2-yl)(phenyl)methanone (5f), $^{13}C\{^{1}H\}$ NMR, $CDCl_3$, 100 MHz

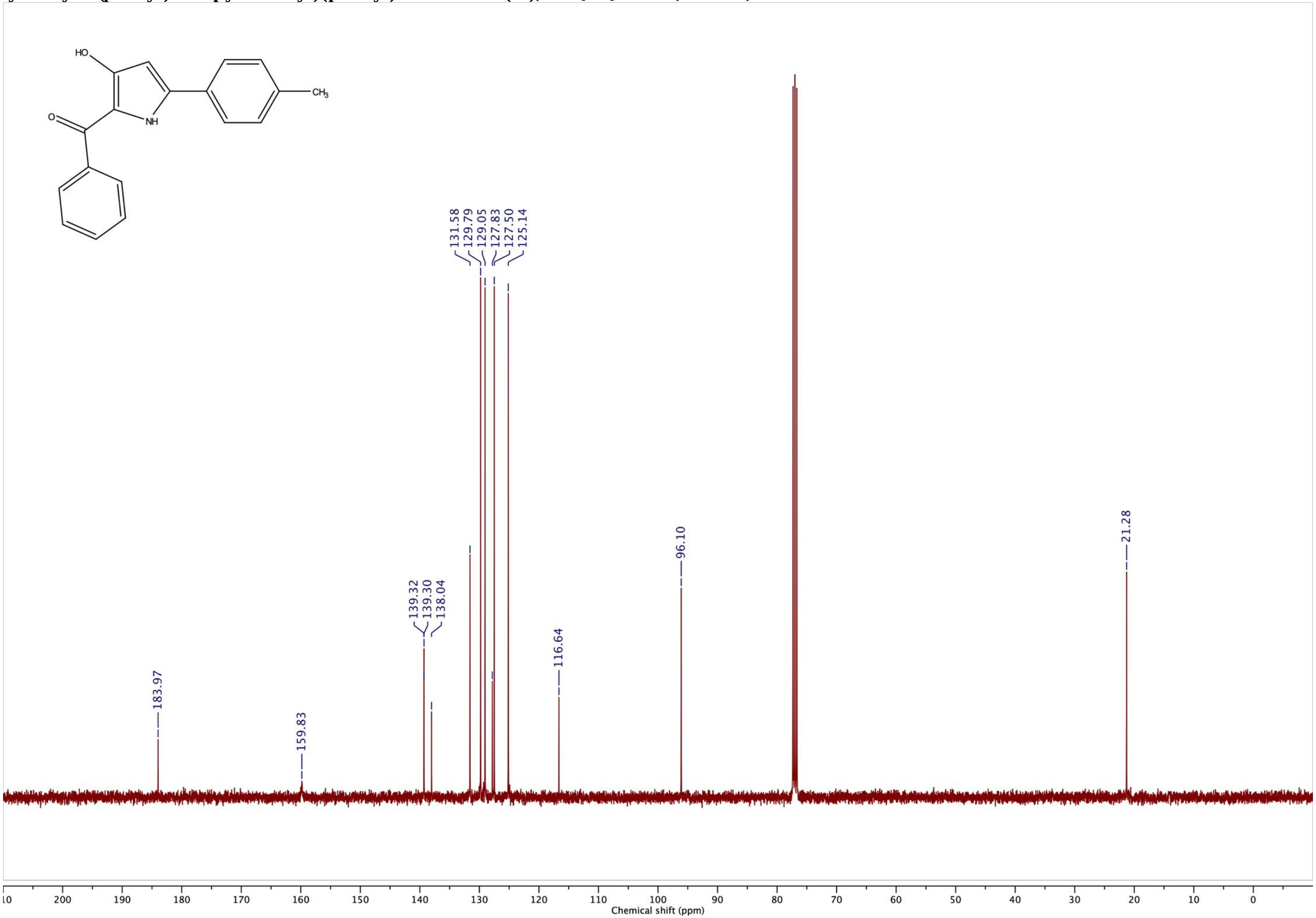

(3-Hydroxy-5-(*p*-tolyl)-1*H*-pyrrol-2-yl)(phenyl)methanone (5f), DEPT, $CDCl_3$, 100 MHz

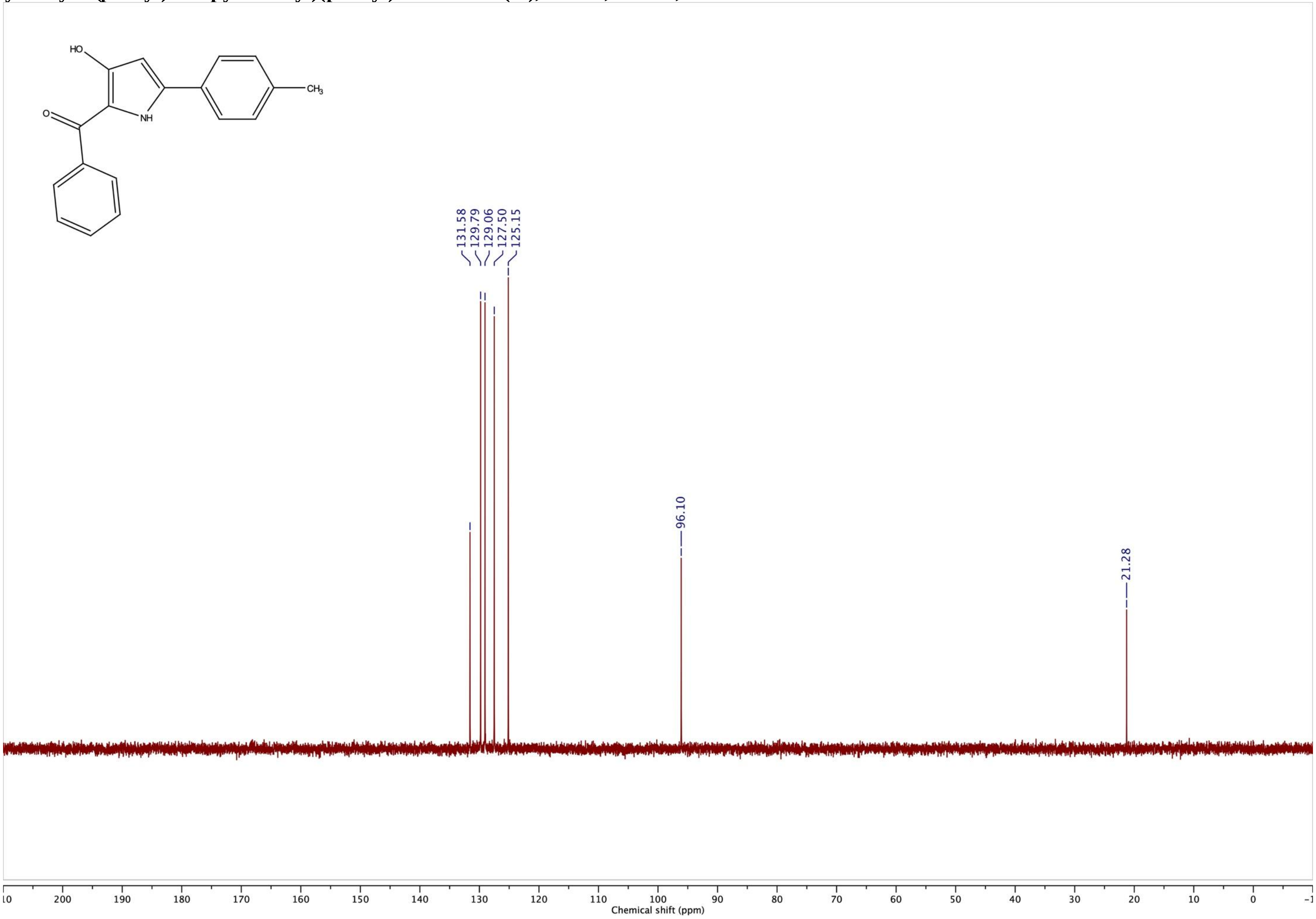

(3-Hydroxy-5-(4-methoxyphenyl)-1*H*-pyrrol-2-yl)(*p*-tolyl)methanone (5g), ^{1}H NMR, $CDCl_3$, 400 MHz

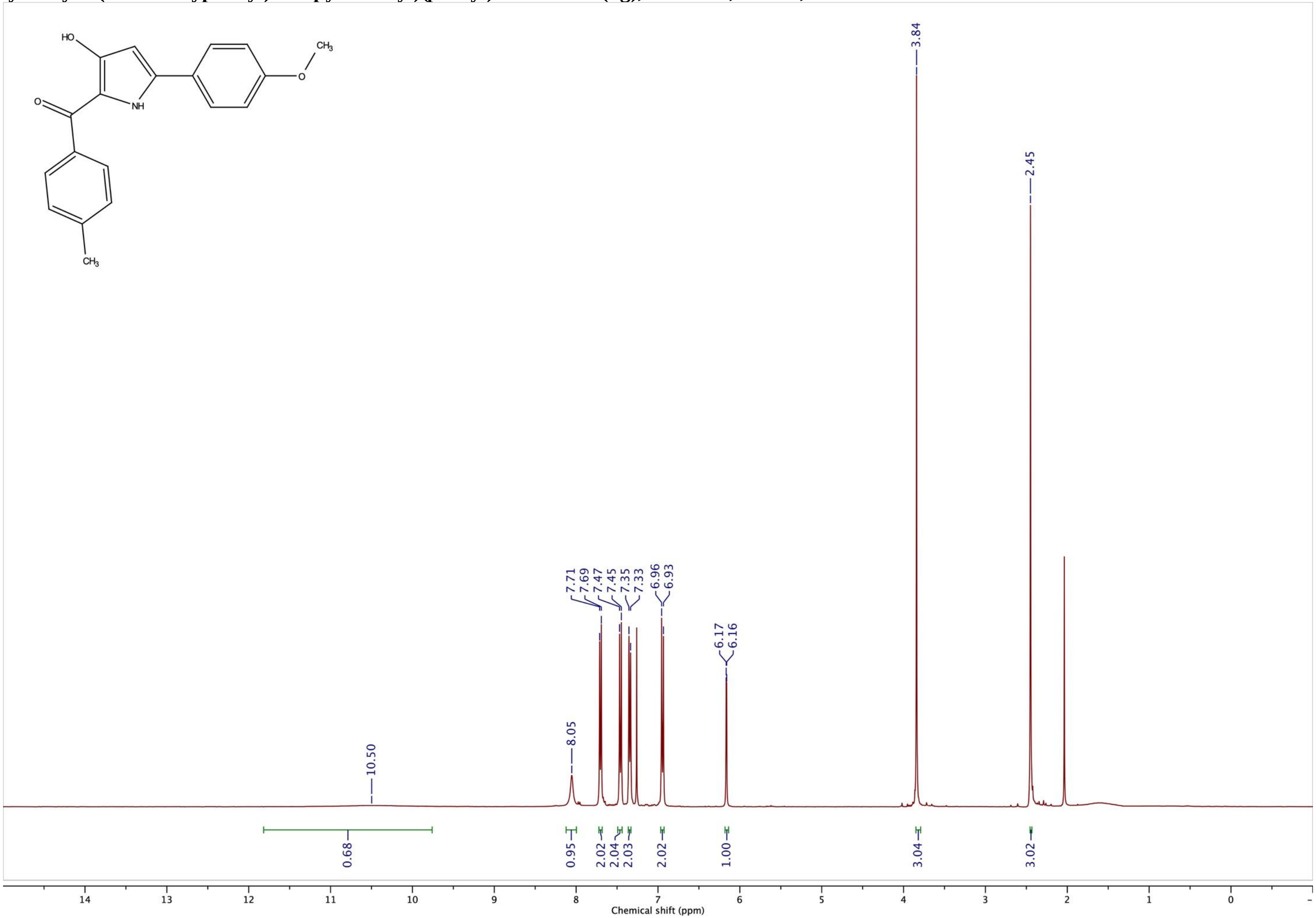

(3-Hydroxy-5-(4-methoxyphenyl)-1*H*-pyrrol-2-yl)(*p*-tolyl)methanone (5g), $^{13}C\{^{1}H\}$ NMR, $CDCl_3$, 100 MHz

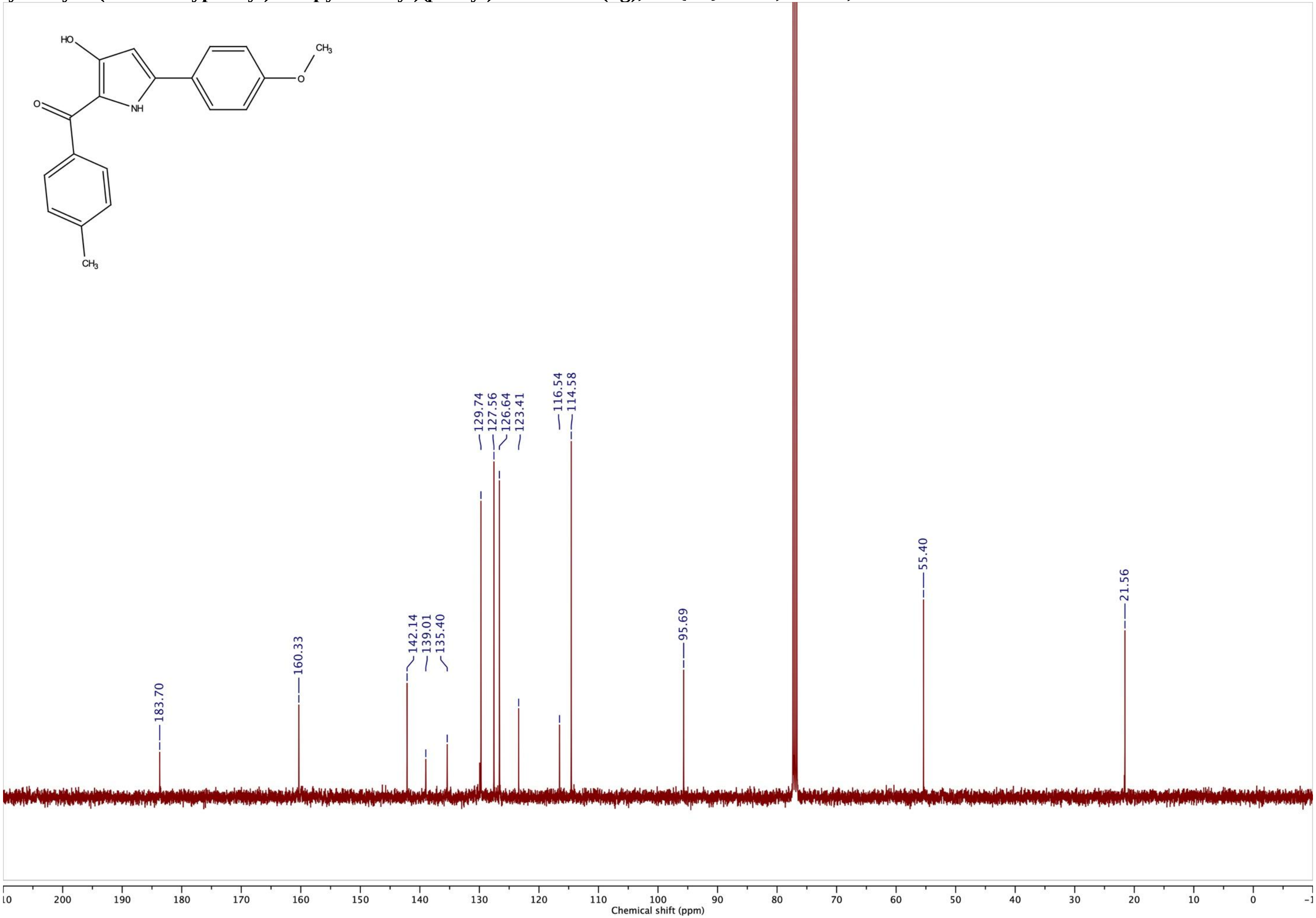

(3-Hydroxy-5-(4-methoxyphenyl)-1*H*-pyrrol-2-yl)(*p*-tolyl)methanone (5g), DEPT, $CDCl_3$, 100 MHz

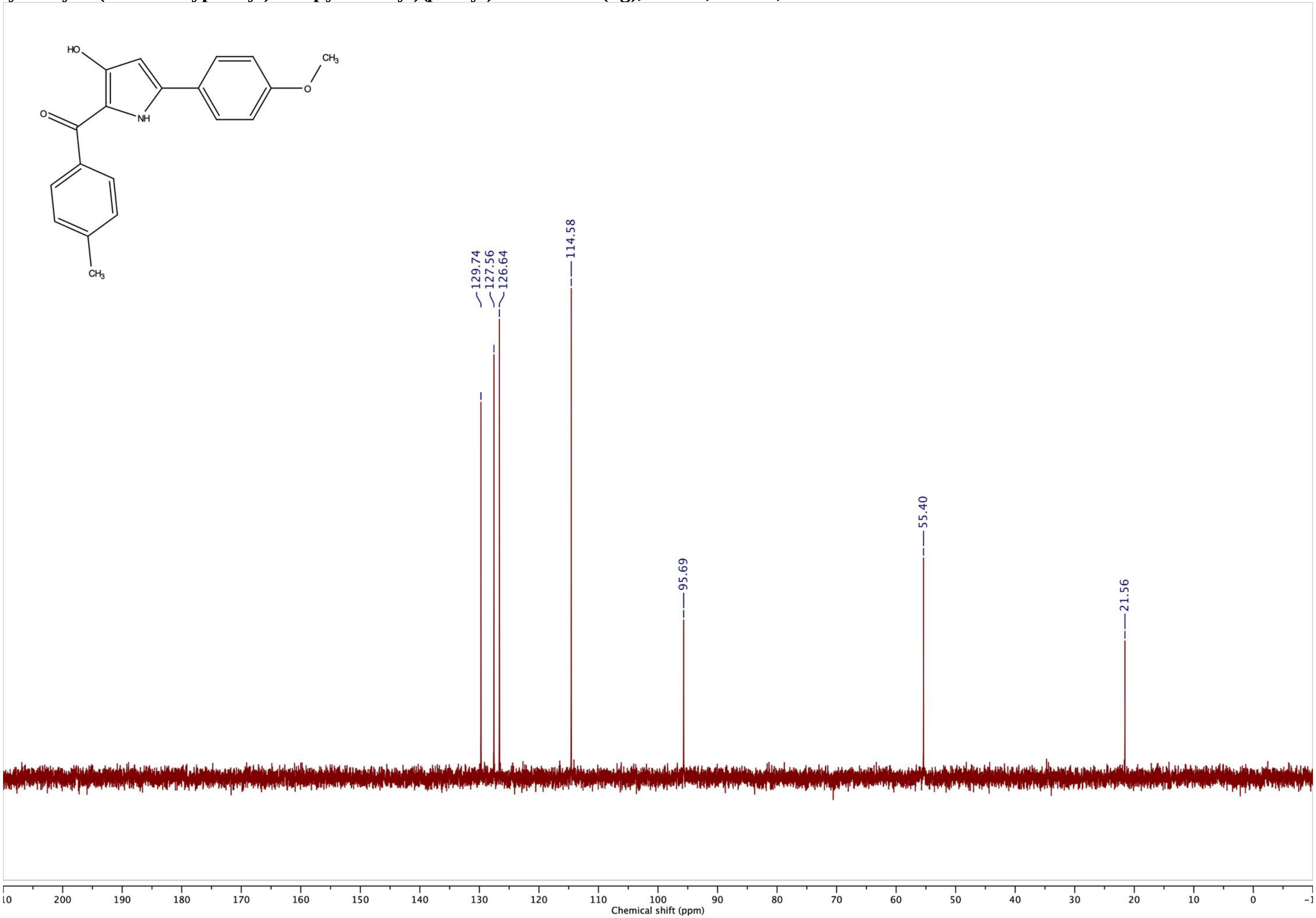

_N_-(4-(4-Hydroxy-5-(4-methylbenzoyl)-1_H_-pyrrol-2-yl)phenyl)acetamide (5h), ^{1}H NMR, DMSO-_d_$_6$, 400 MHz

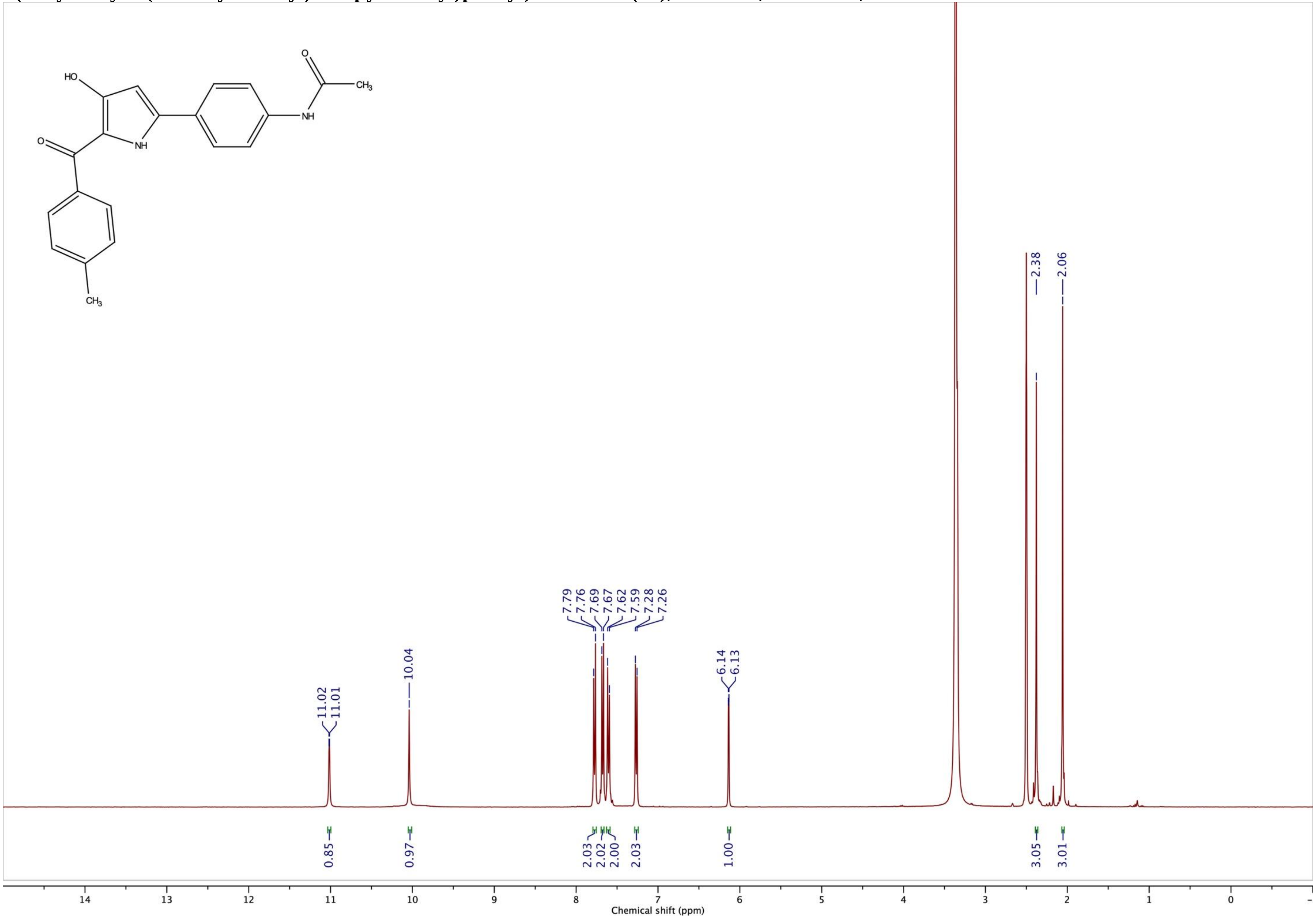

***N*-(4-(4-Hydroxy-5-(4-methylbenzoyl)-1*H*-pyrrol-2-yl)phenyl)acetamide (5h), $^{13}C\{^{1}H\}$ NMR, DMSO-d_6, 100 MHz**

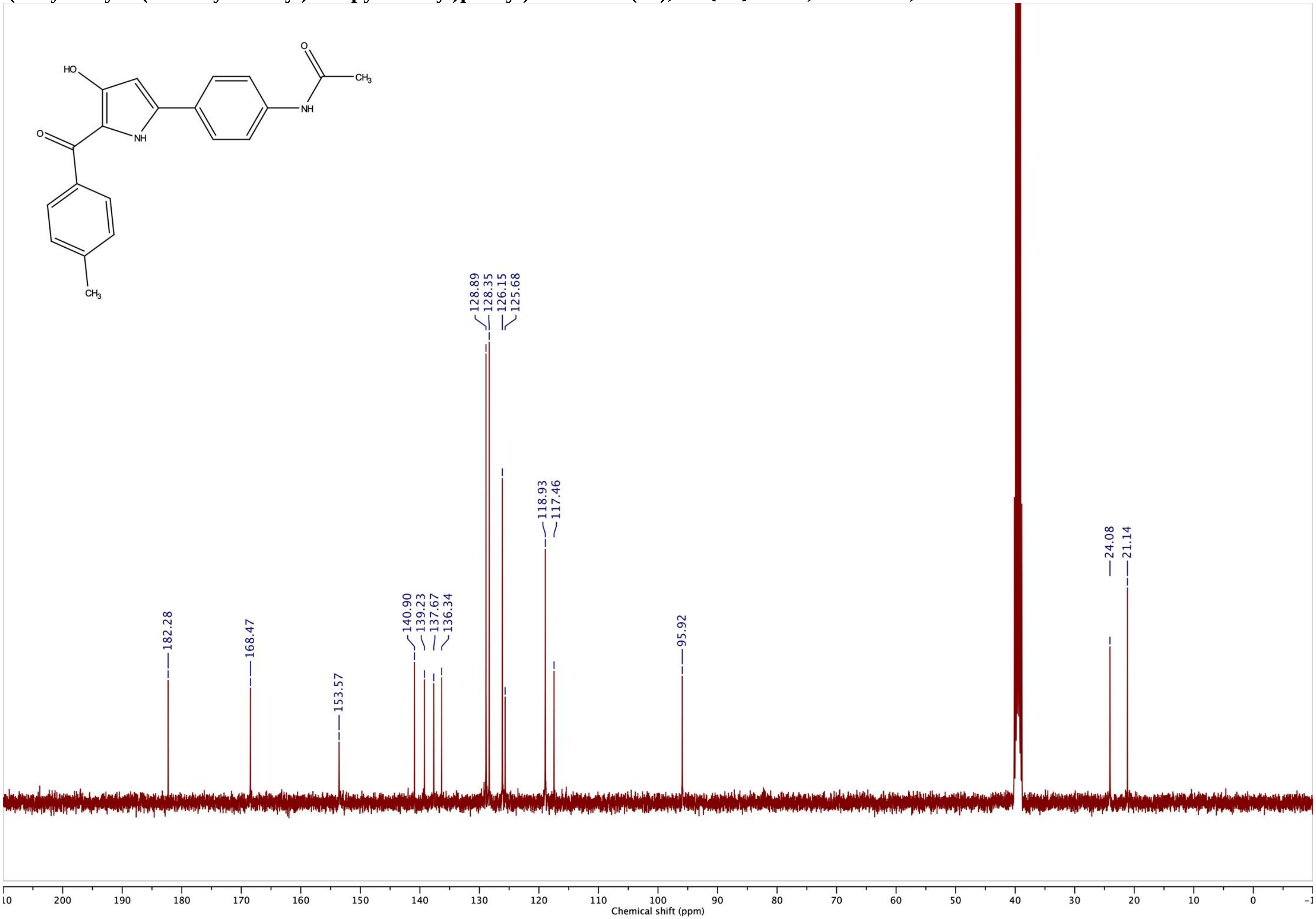

N-(4-(4-Hydroxy-5-(4-methylbenzoyl)-1H-pyrrol-2-yl)phenyl)acetamide (5h), DEPT, DMSO-d_6, 100 MHz

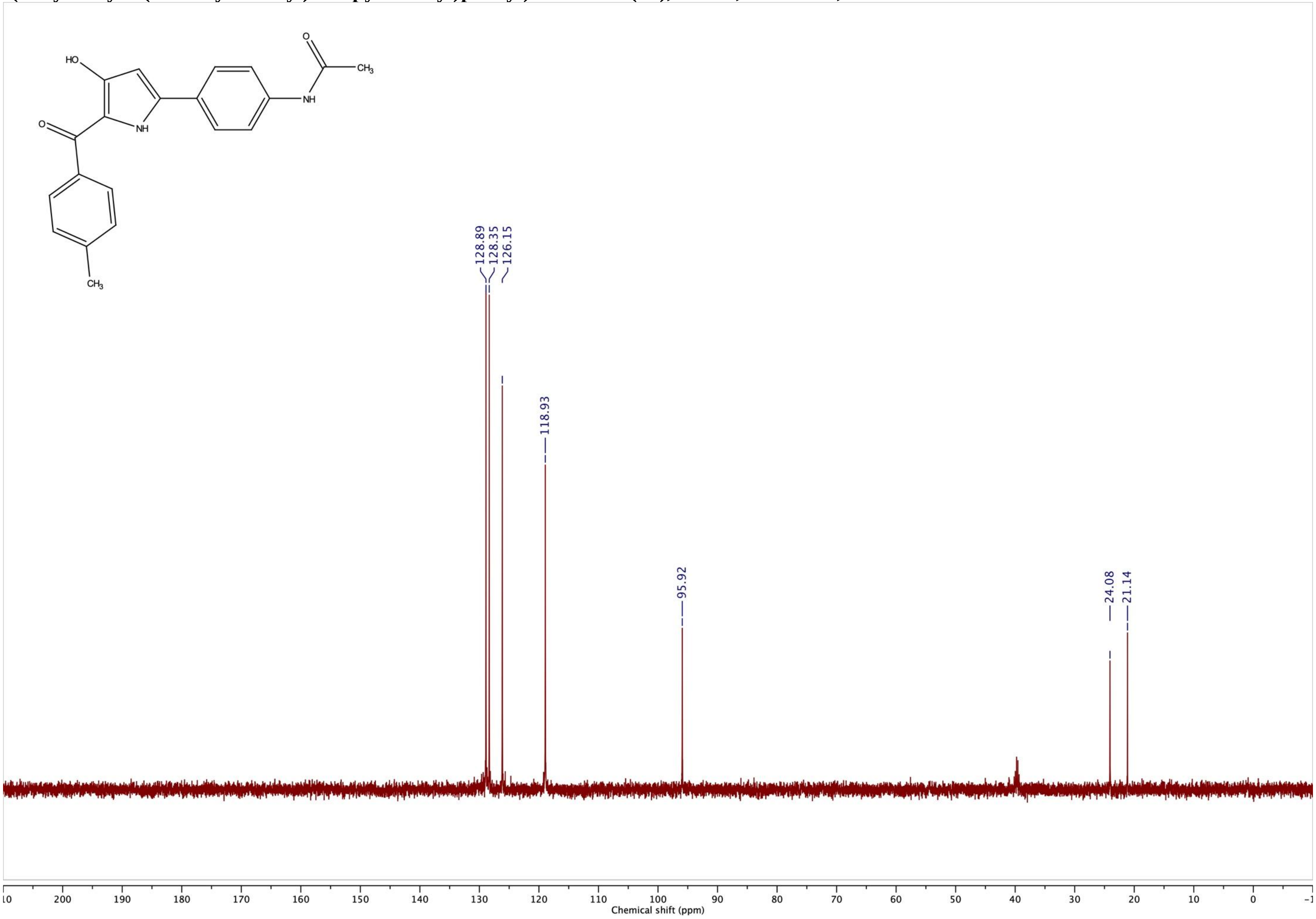

(5-(2,3-Dihydrobenzo[*b*][1,4]dioxin-6-yl)-3-hydroxy-1*H*-pyrrol-2-yl)(*p*-tolyl)methanone (5i), ^{1}H NMR, CDCl$_3$, 400 MHz

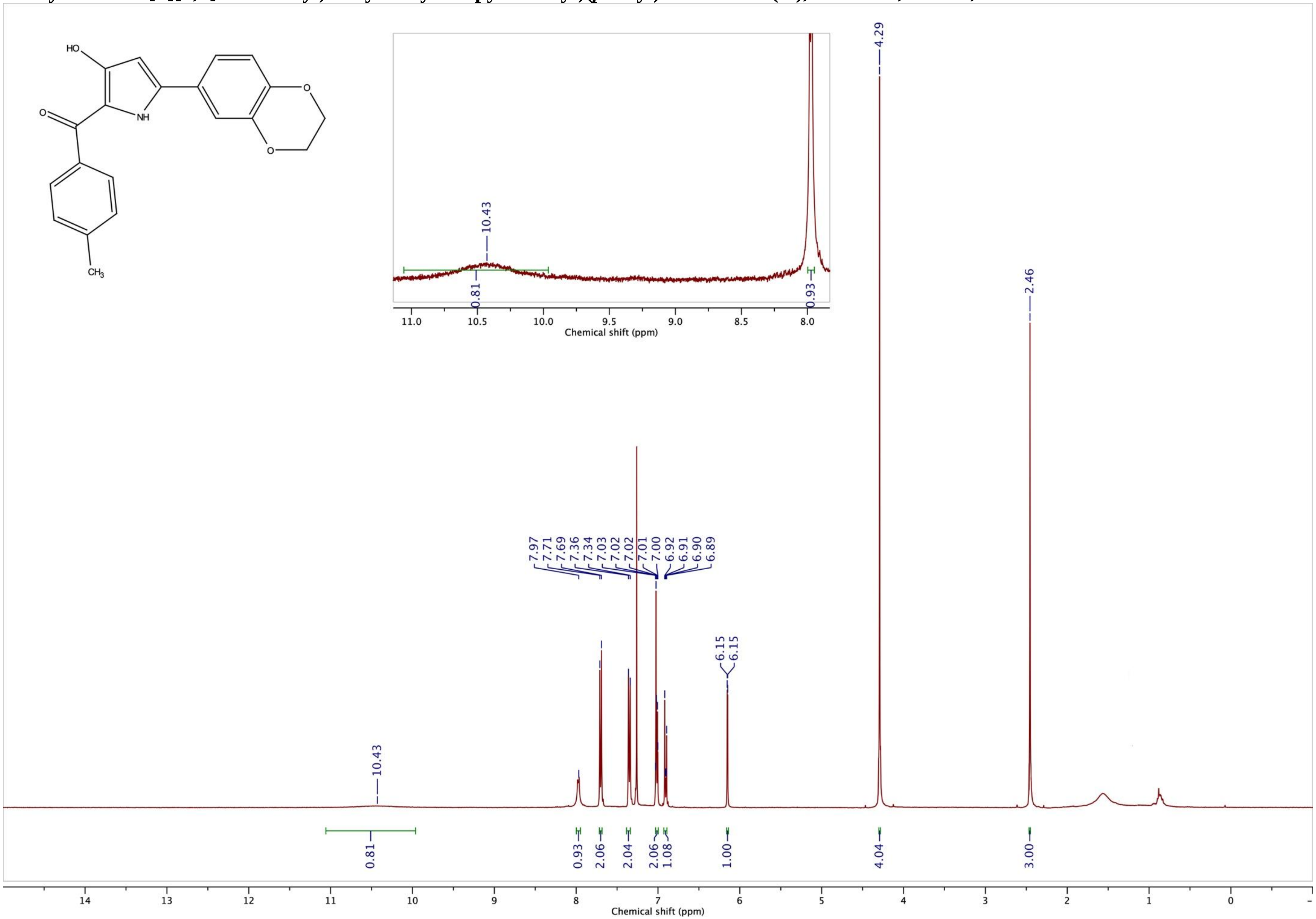

(5-(2,3-Dihydrobenzo[*b*][1,4]dioxin-6-yl)-3-hydroxy-1*H*-pyrrol-2-yl)(*p*-tolyl)methanone (5i), $^{13}C\{^1H\}$ NMR, $CDCl_3$, 100 MHz

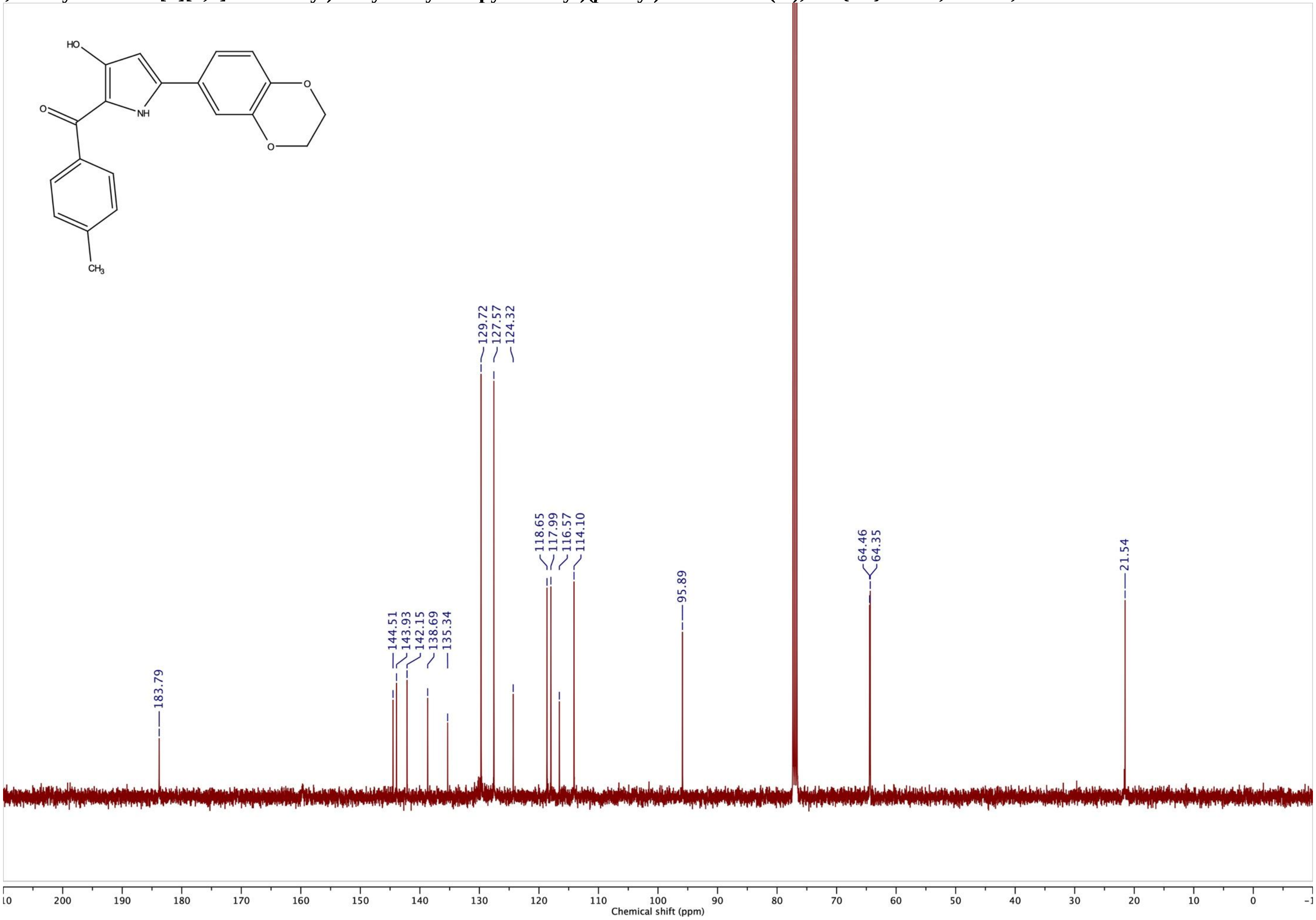

(5-(2,3-Dihydrobenzo[*b*][1,4]dioxin-6-yl)-3-hydroxy-1*H*-pyrrol-2-yl)(*p*-tolyl)methanone (5i), DEPT, $CDCl_3$, 100 MHz

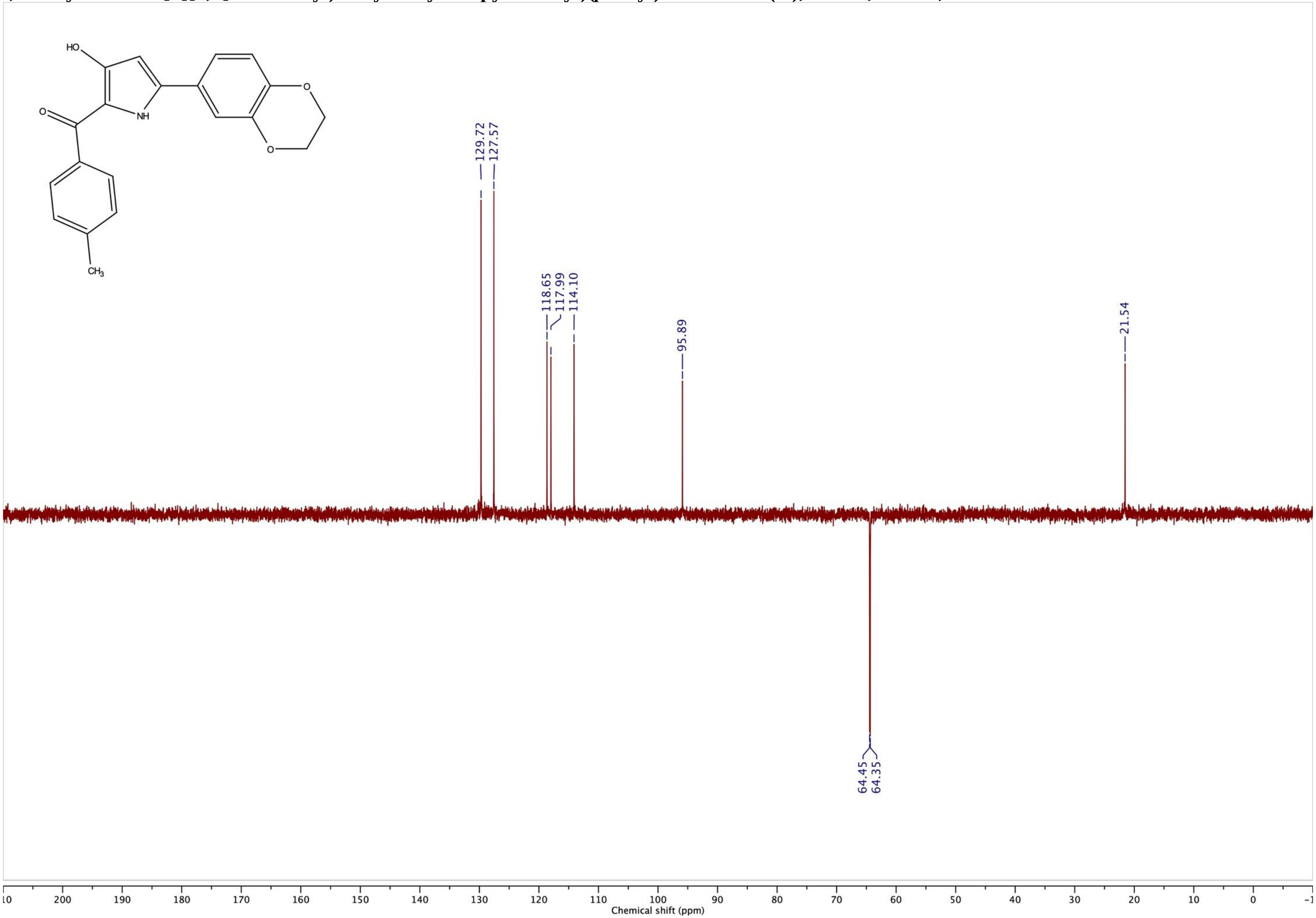

(5-(4-Bromophenyl)-3-hydroxy-1*H*-pyrrol-2-yl)(4-(*tert*-butyl)phenyl)methanone (5j), ^{1}H NMR, $CDCl_3$, 400 MHz

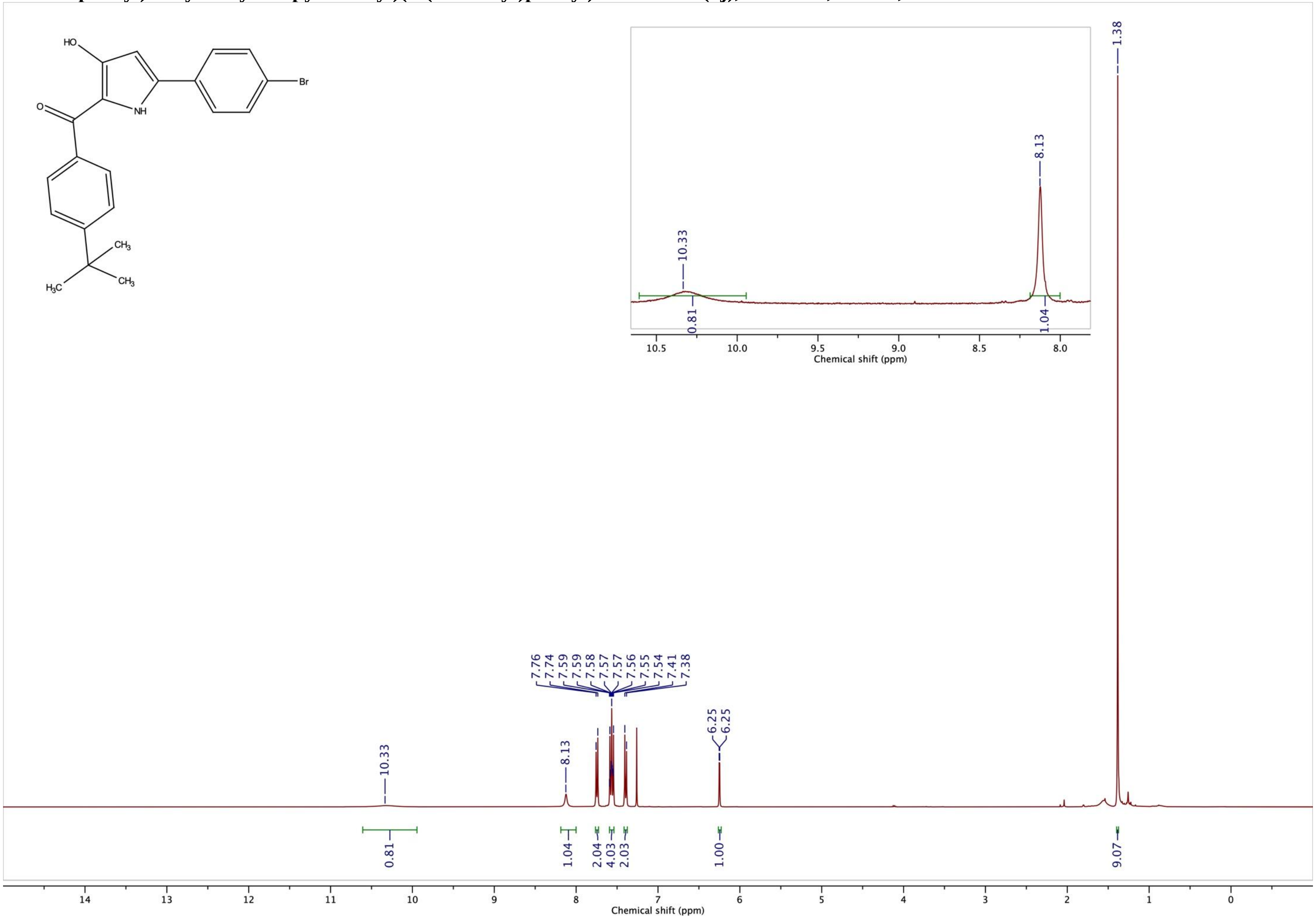

(5-(4-Bromophenyl)-3-hydroxy-1*H*-pyrrol-2-yl)(4-(*tert*-butyl)phenyl)methanone (5j), $^{13}C\{^{1}H\}$ NMR, $CDCl_3$, 100 MHz

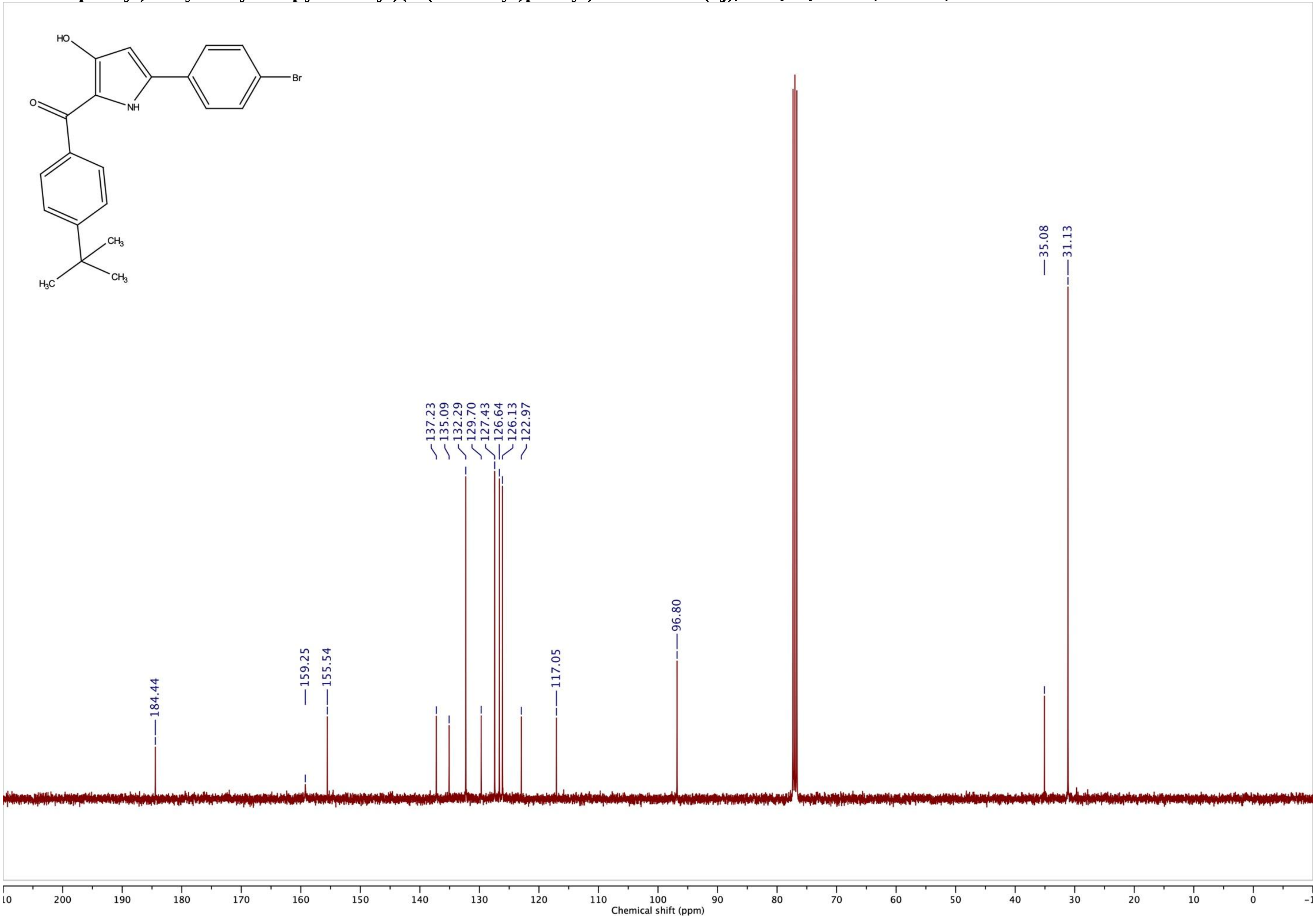

(5-(4-Bromophenyl)-3-hydroxy-1*H*-pyrrol-2-yl)(4-(*tert*-butyl)phenyl)methanone (5j), DEPT, $CDCl_3$, 100 MHz

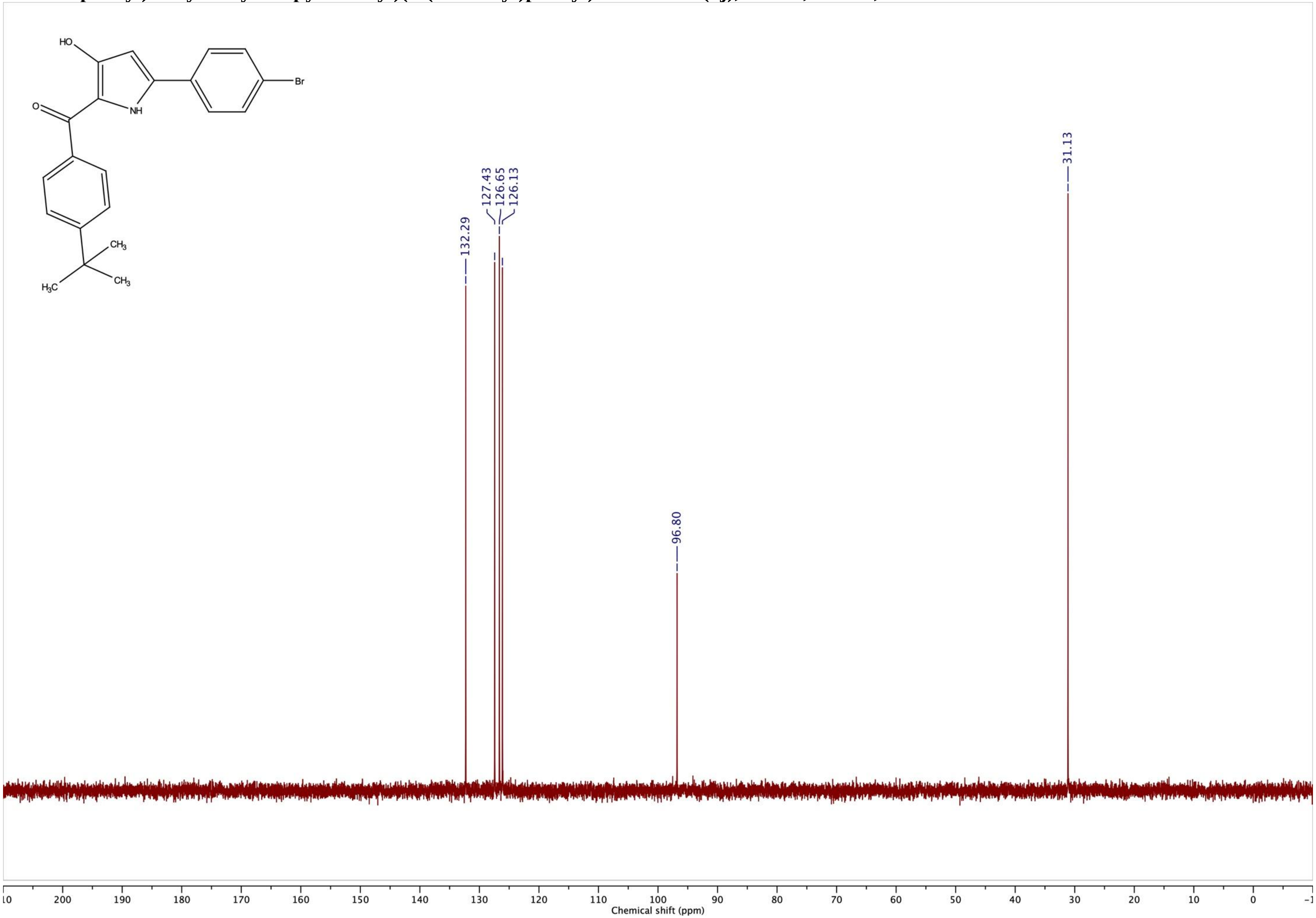

(5-(3-Bromophenyl)-3-hydroxy-1*H*-pyrrol-2-yl)(4-fluorophenyl)methanone (5k), ^{1}H NMR, $CDCl_3$, 400 MHz

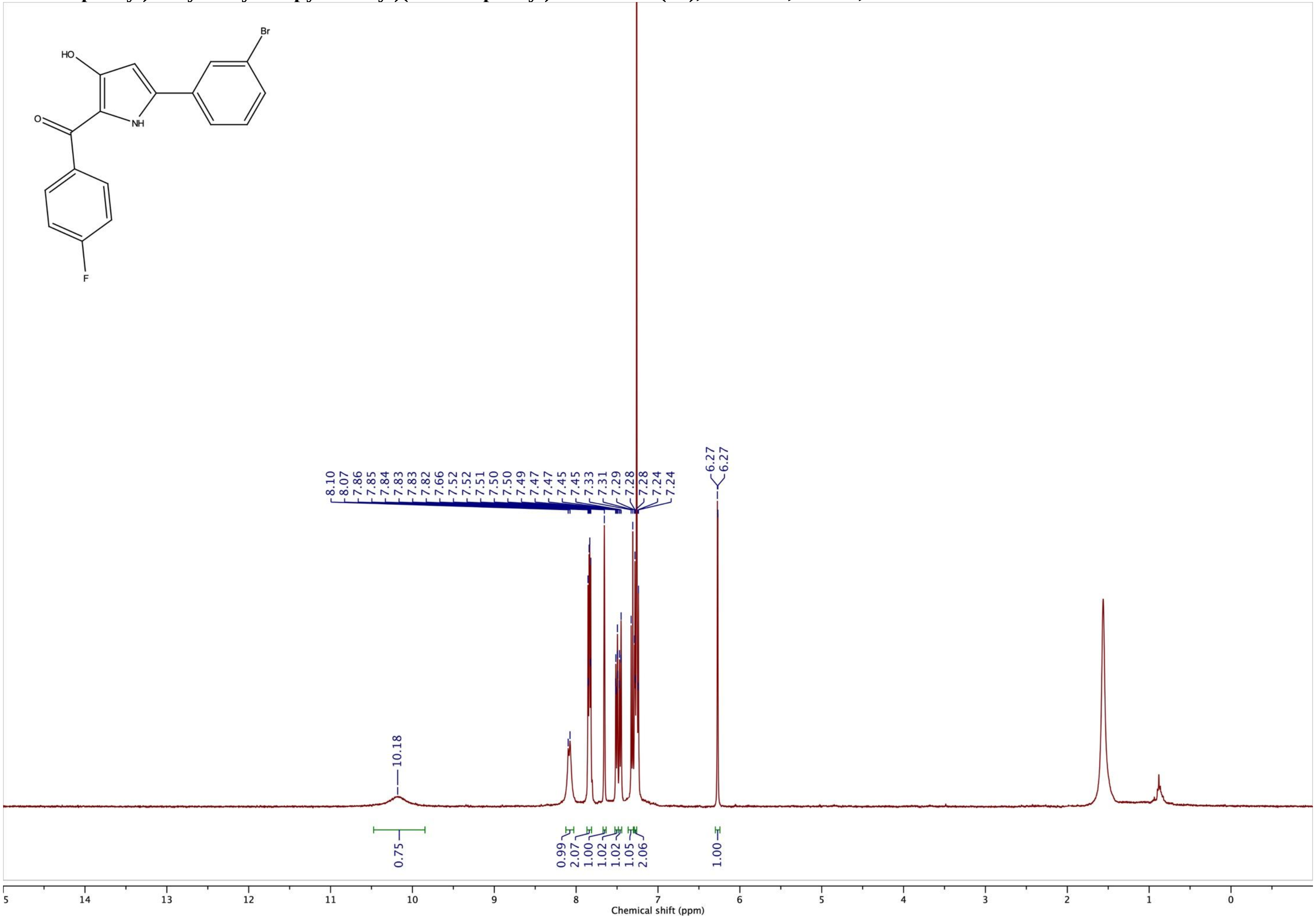

(5-(3-Bromophenyl)-3-hydroxy-1*H*-pyrrol-2-yl)(4-fluorophenyl)methanone (5k), $^{13}C\{^{1}H\}$ NMR, $CDCl_3$, 100 MHz

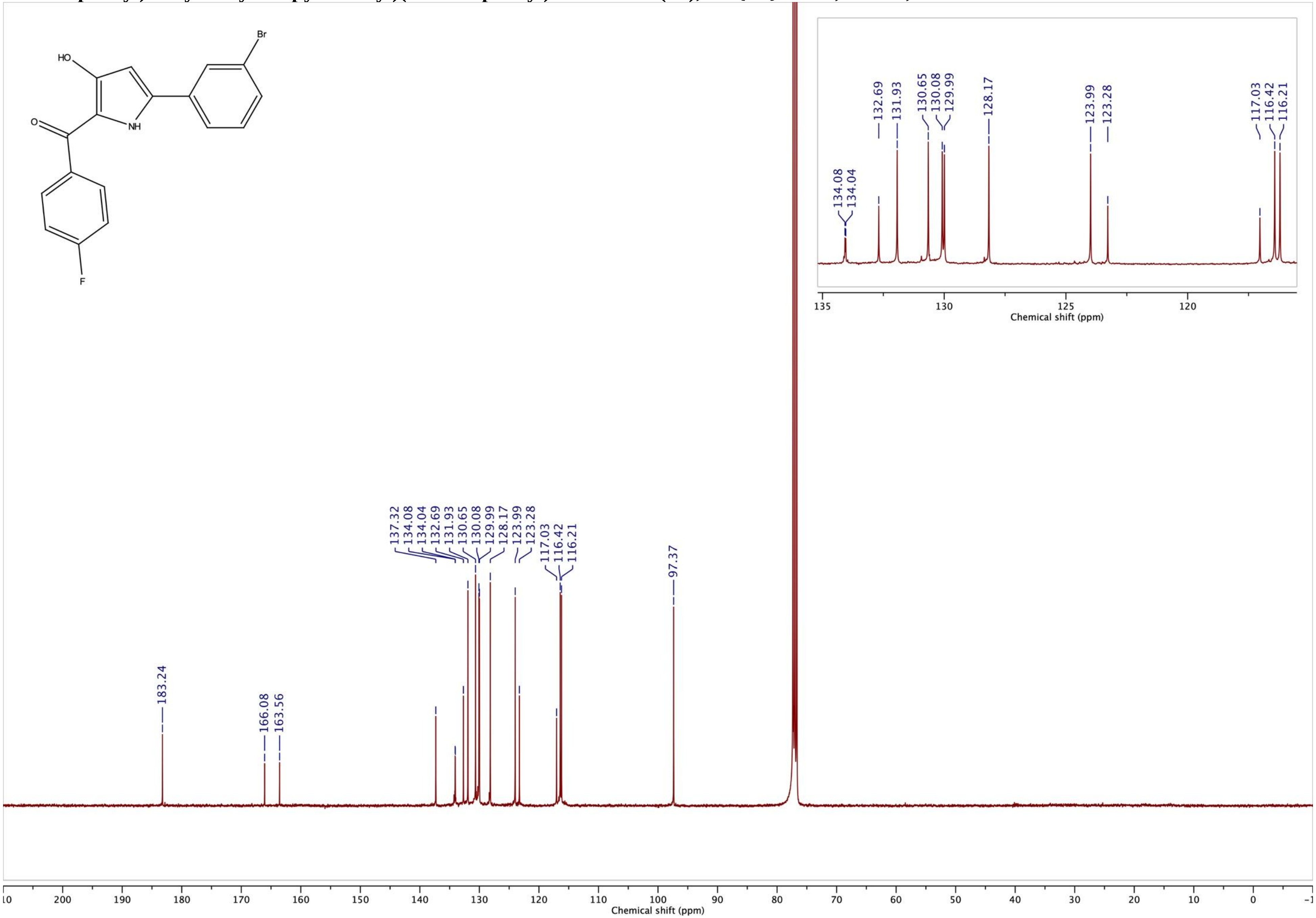

(5-(3-Bromophenyl)-3-hydroxy-1*H*-pyrrol-2-yl)(4-fluorophenyl)methanone (5k), DEPT, $CDCl_3$, 100 MHz

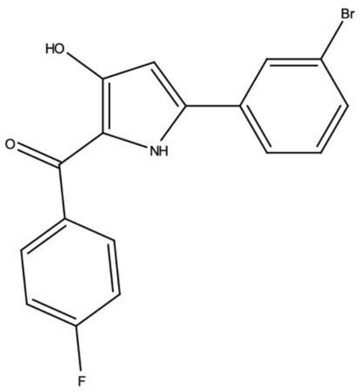

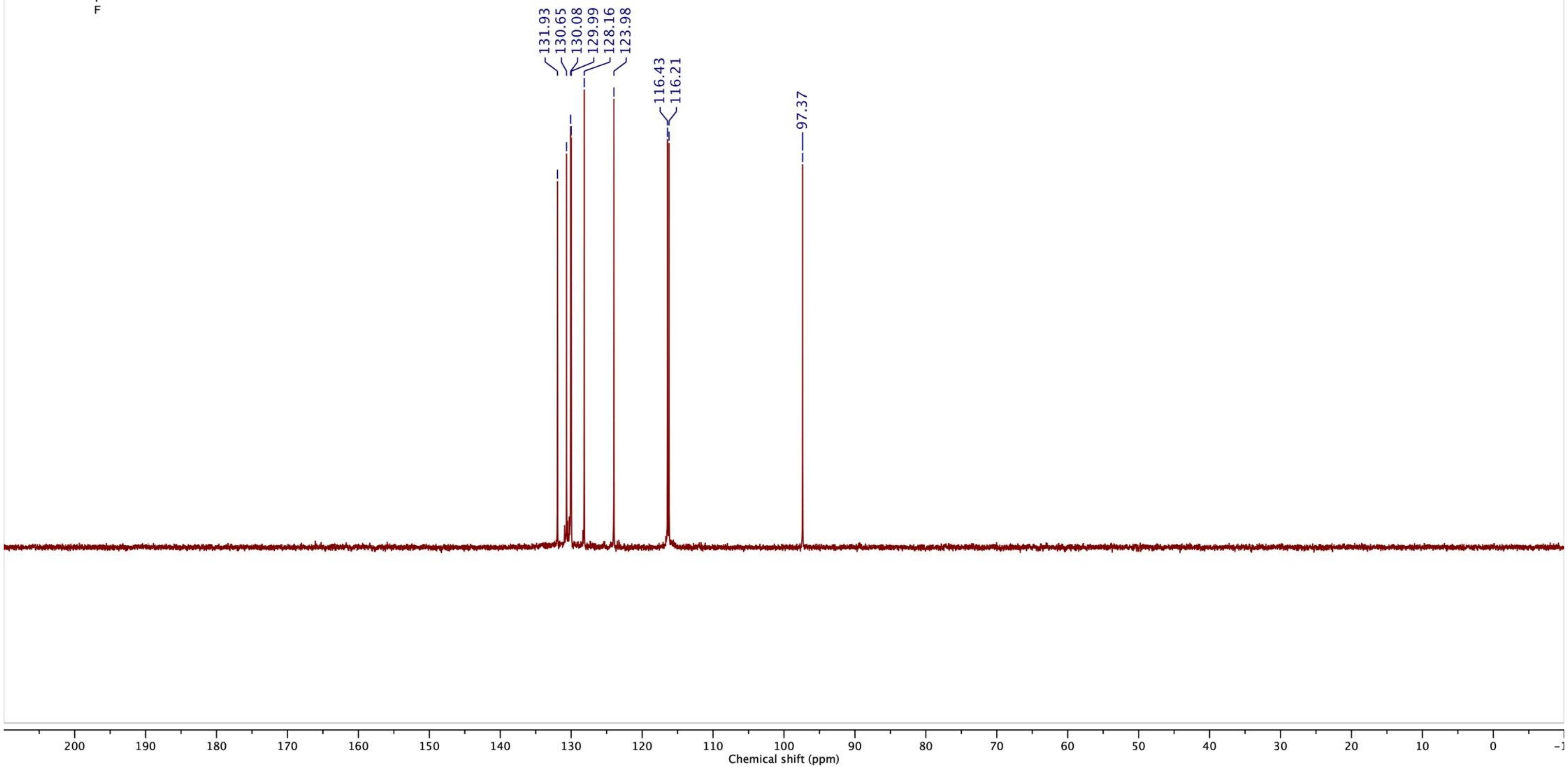

(4-Chlorophenyl)(3-hydroxy-5-(*p*-tolyl)-1*H*-pyrrol-2-yl)methanone (5l), ^{1}H NMR, $CDCl_3$, 400 MHz

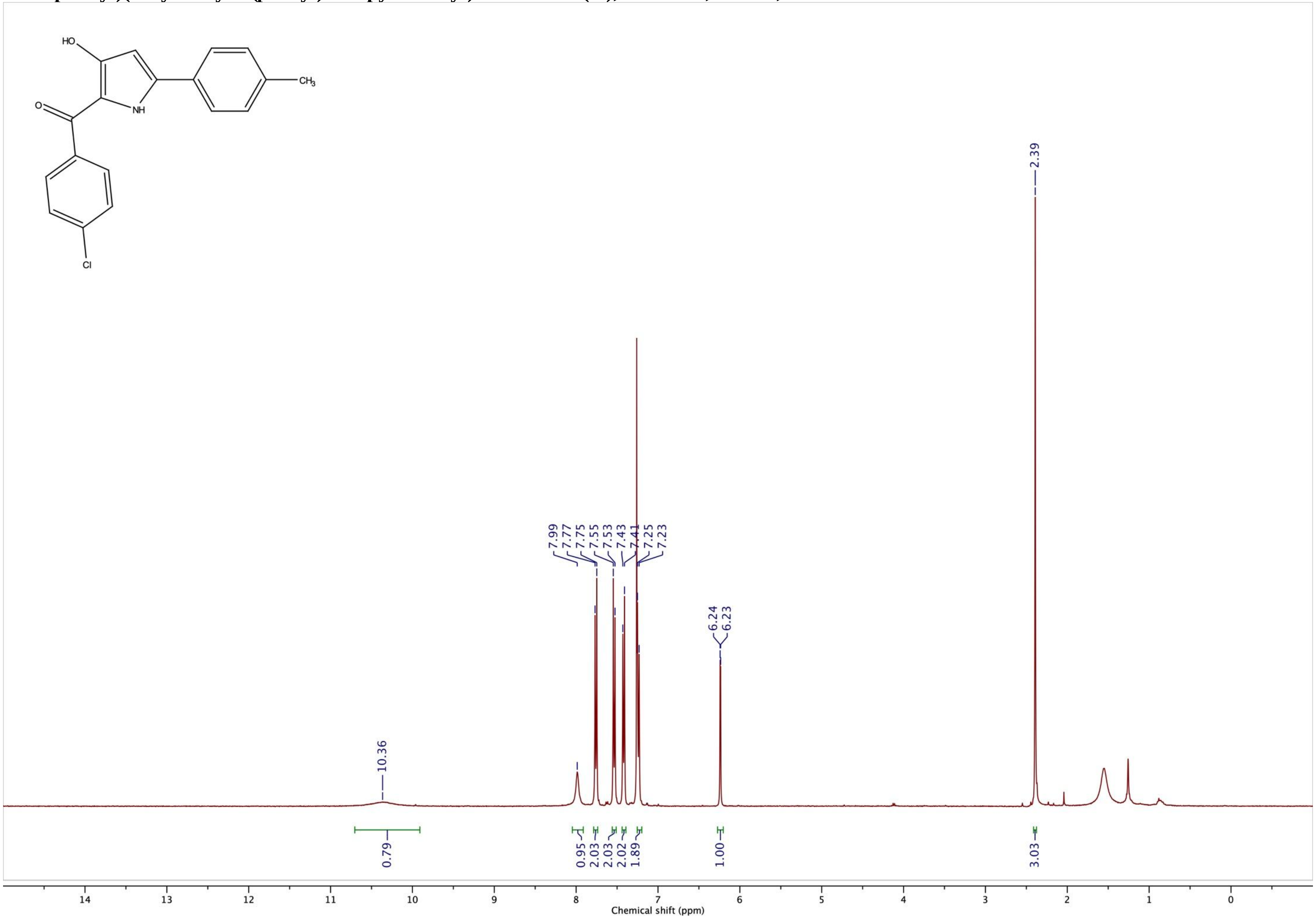

(4-Chlorophenyl)(3-hydroxy-5-(*p*-tolyl)-1*H*-pyrrol-2-yl)methanone (5l), $^{13}C\{^{1}H\}$ NMR, $CDCl_3$, 100 MHz

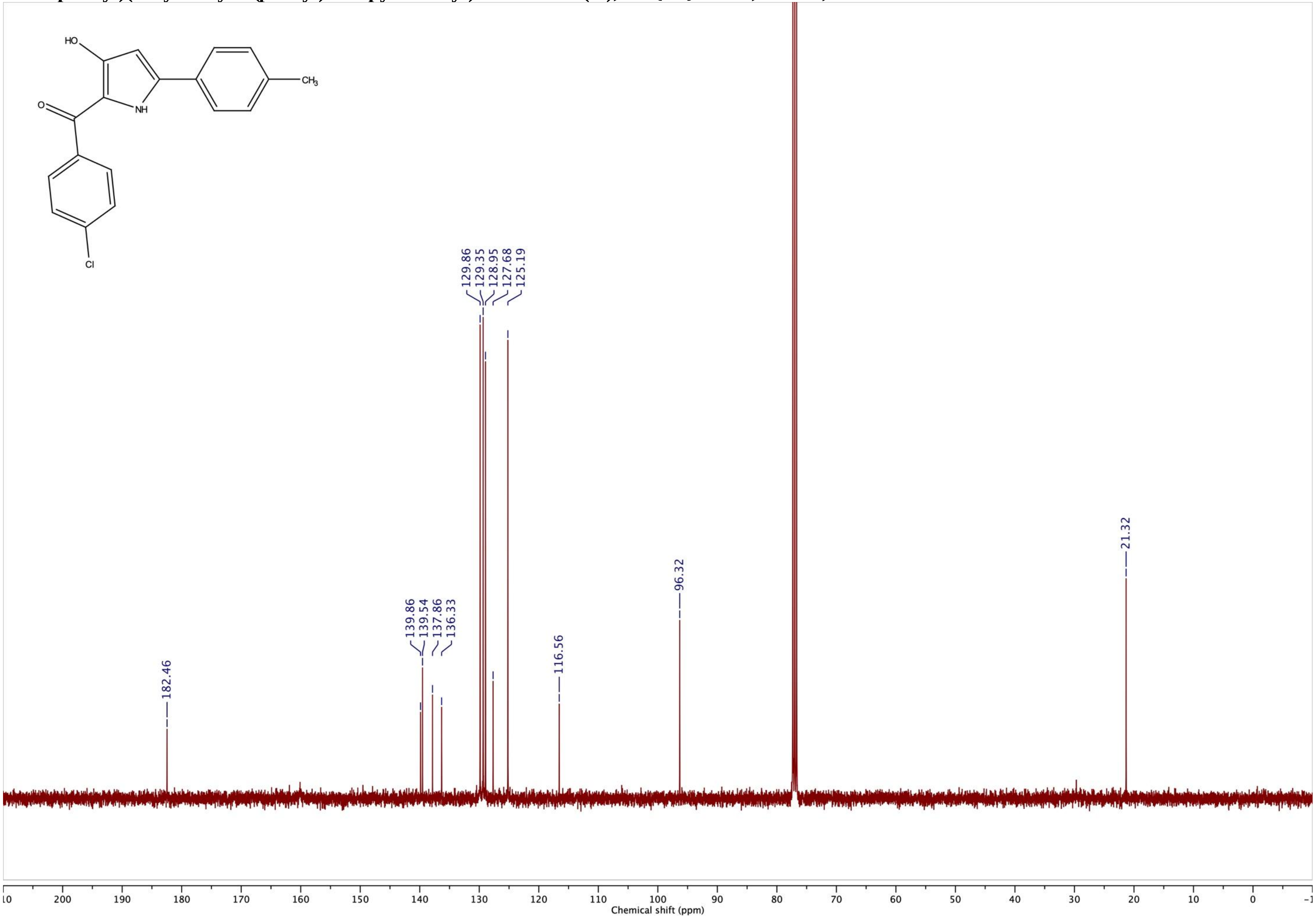

(4-Chlorophenyl)(3-hydroxy-5-(*p*-tolyl)-1*H*-pyrrol-2-yl)methanone (5l), DEPT, $CDCl_3$, 100 MHz

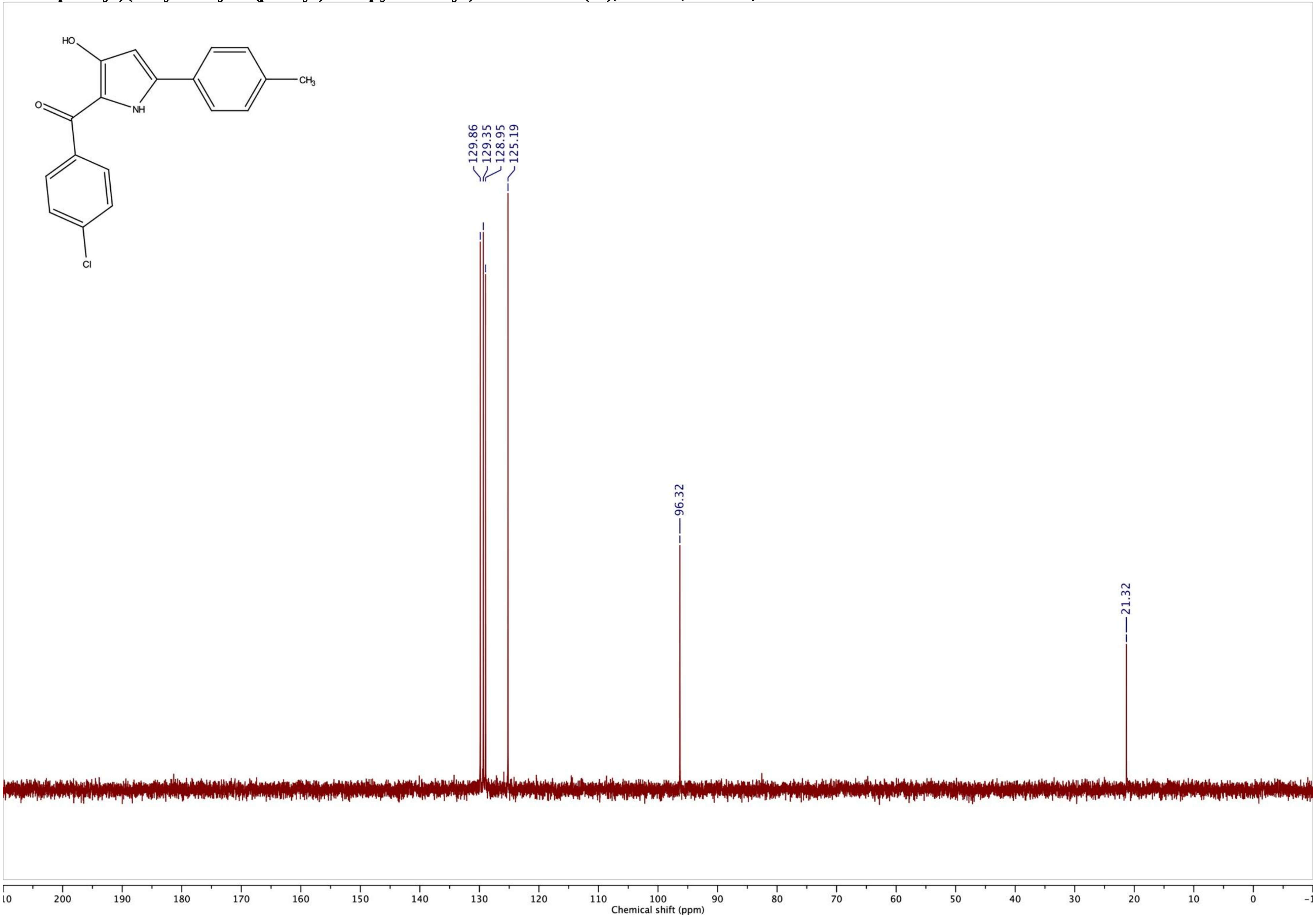

(4-Bromophenyl)(5-(2-chlorophenyl)-3-hydroxy-1*H*-pyrrol-2-yl)methanone (5m), ^{1}H NMR, $CDCl_3$, 400 MHz

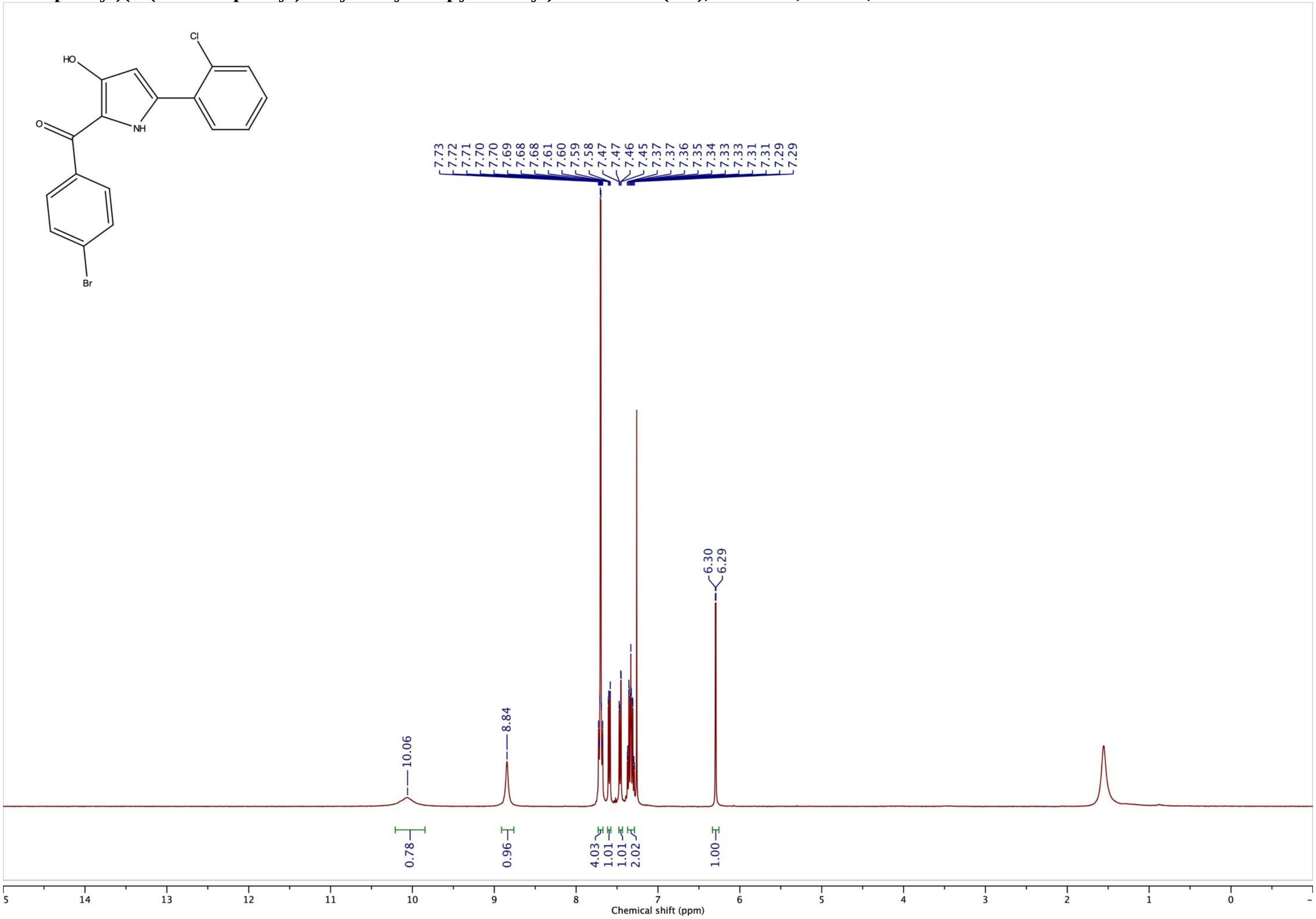

(4-Bromophenyl)(5-(2-chlorophenyl)-3-hydroxy-1*H*-pyrrol-2-yl)methanone (5m), $^{13}C\{^1H\}$ NMR, $CDCl_3$, 100 MHz

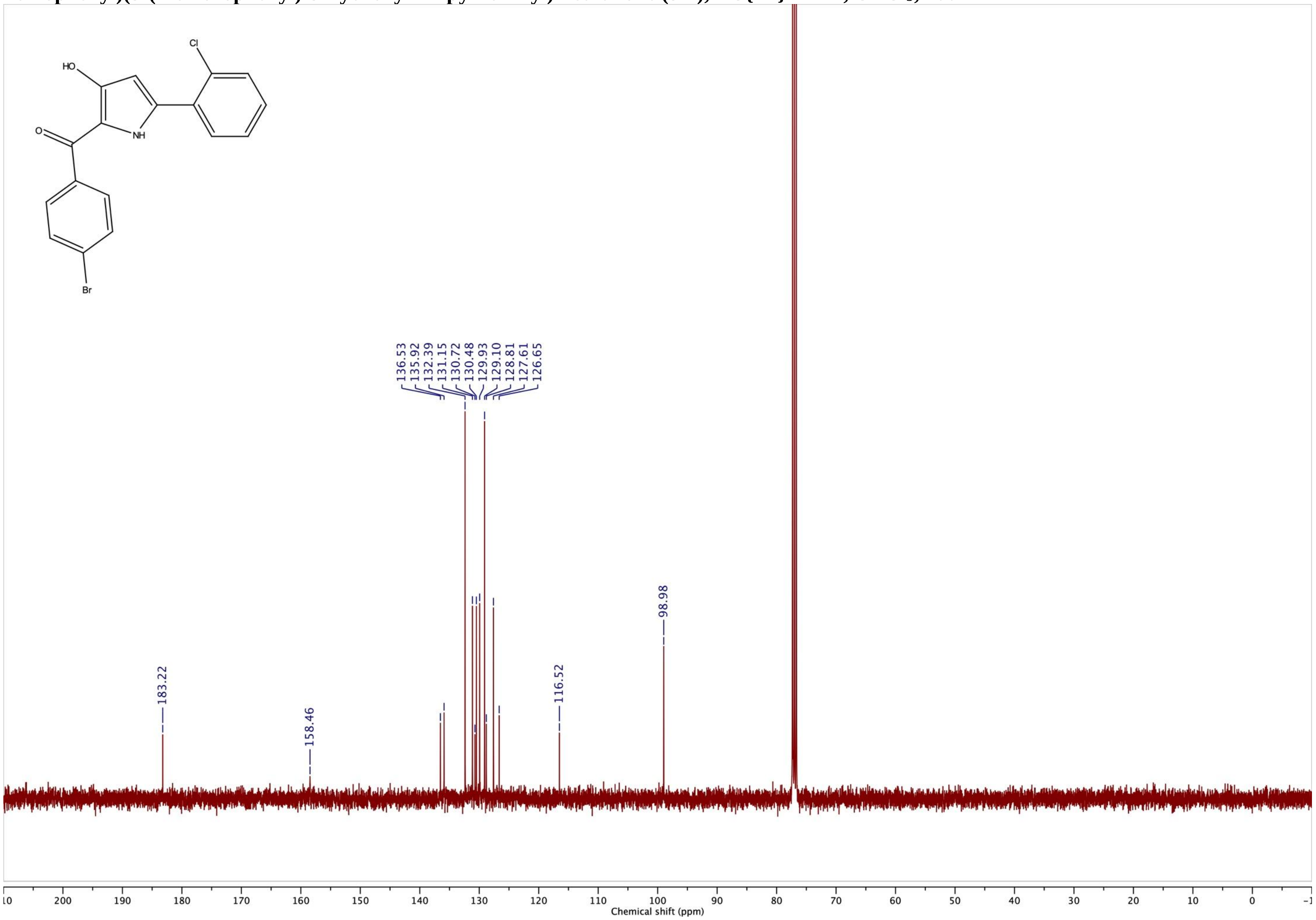

(4-Bromophenyl)(5-(2-chlorophenyl)-3-hydroxy-1*H*-pyrrol-2-yl)methanone (5m), DEPT, $CDCl_3$, 100 MHz

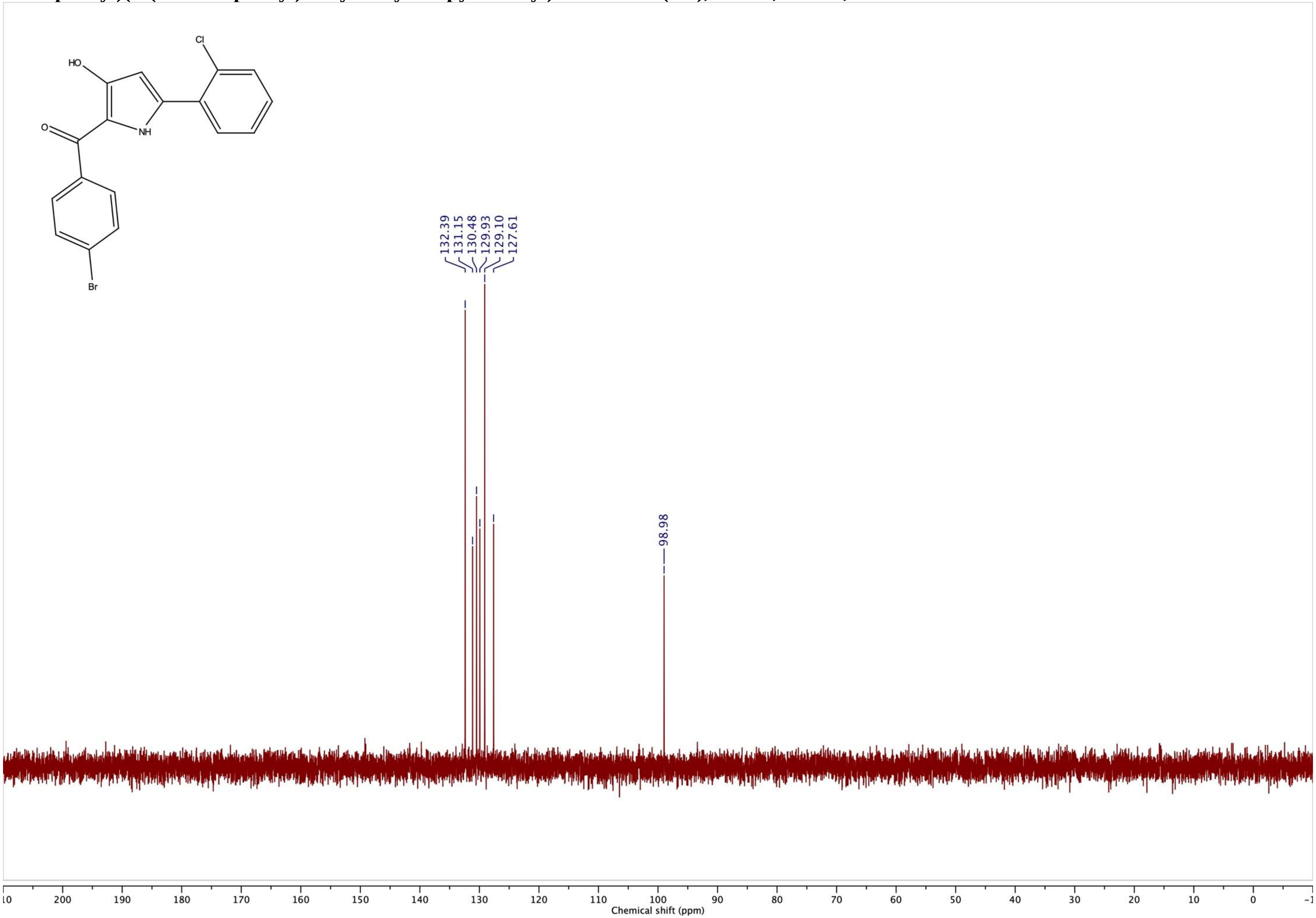

(2-Bromophenyl)(3-hydroxy-5-(*p*-tolyl)-1*H*-pyrrol-2-yl)methanone (5n), ^{1}H NMR, $CDCl_3$, 400 MHz

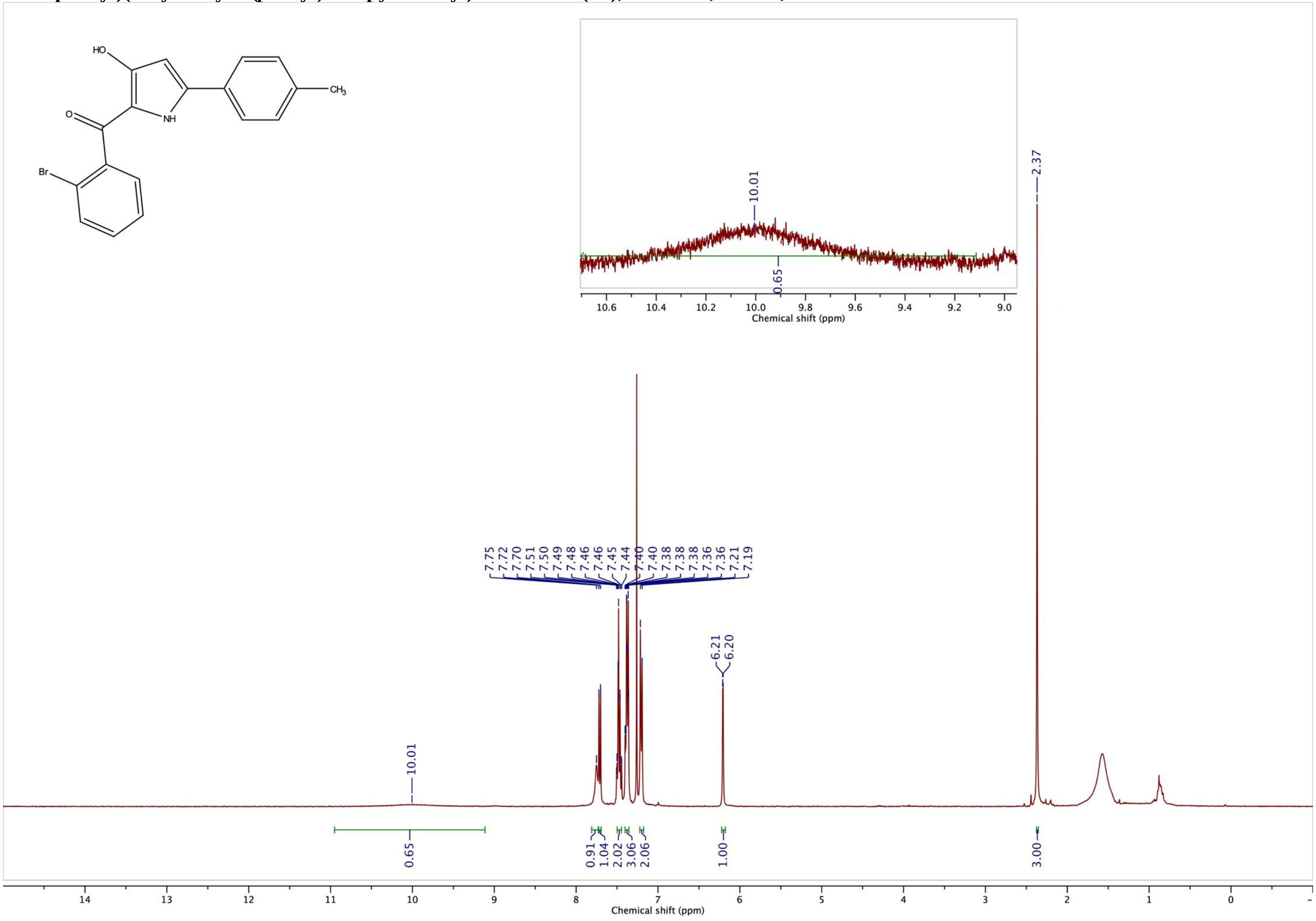

(2-Bromophenyl)(3-hydroxy-5-(*p*-tolyl)-1*H*-pyrrol-2-yl)methanone (5n), $^{13}C\{^{1}H\}$ NMR, $CDCl_3$, 100 MHz

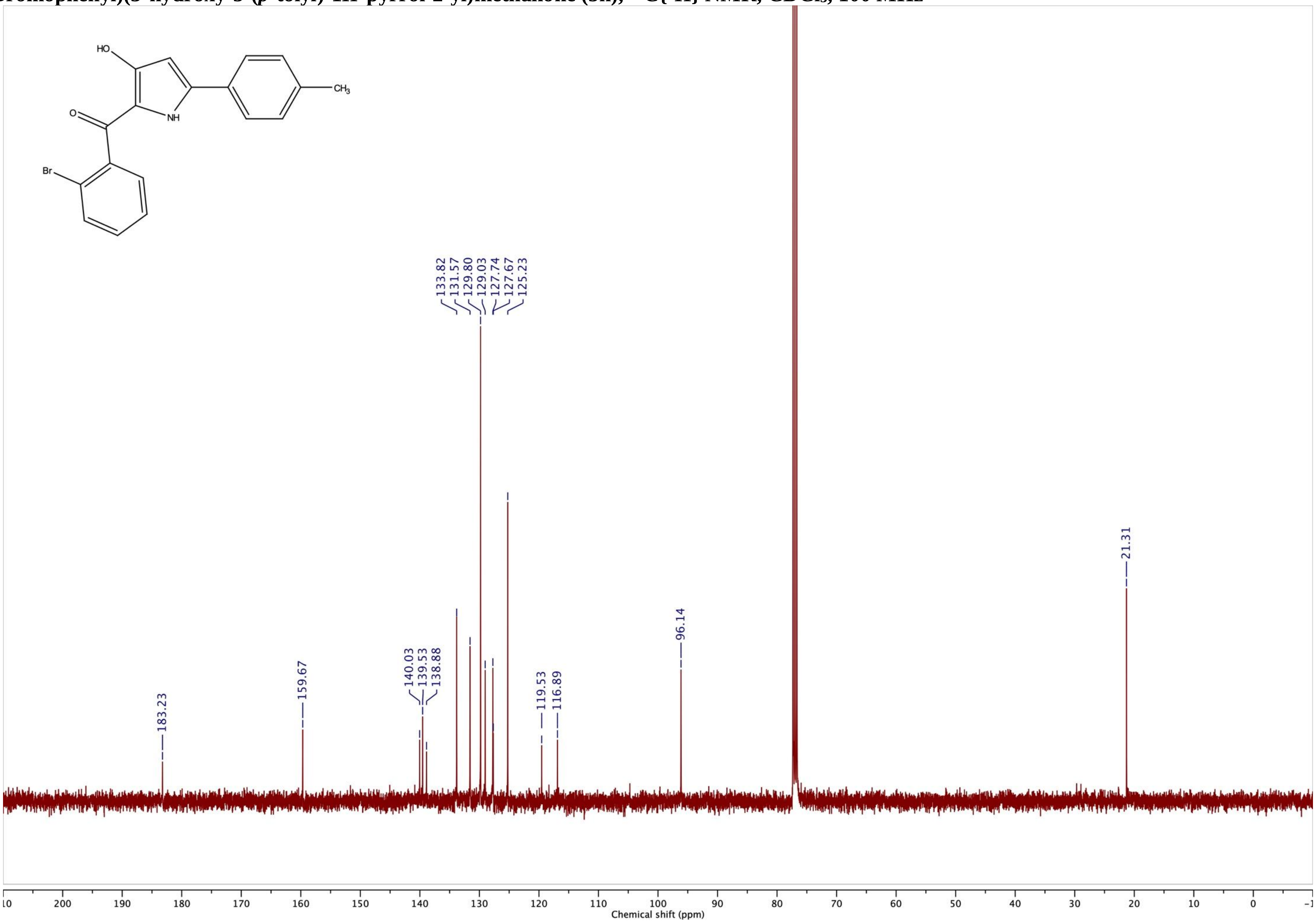

(2-Bromophenyl)(3-hydroxy-5-(*p*-tolyl)-1*H*-pyrrol-2-yl)methanone (5n), DEPT, CDCl₃, 100 MHz

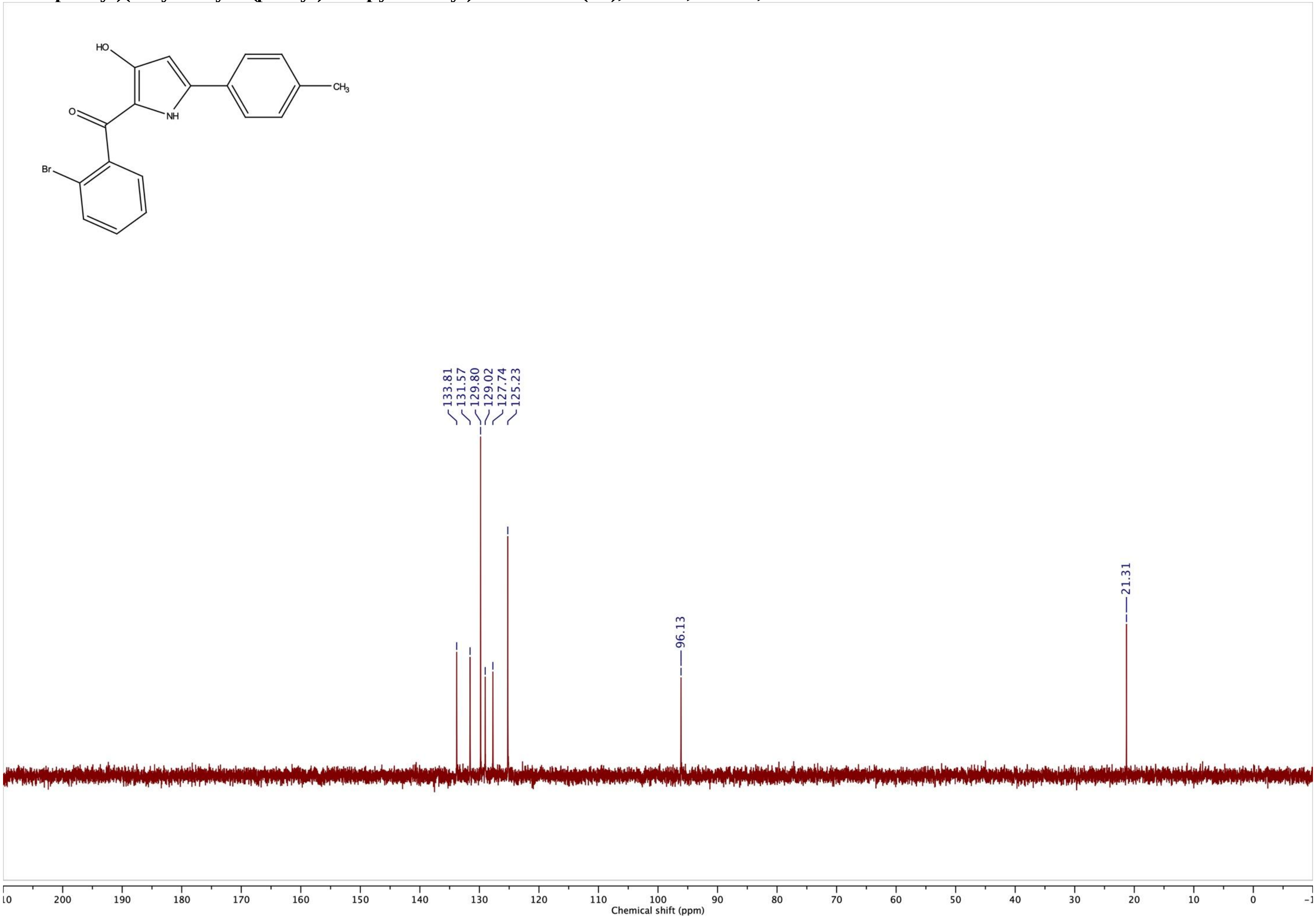

Adamantan-1-yl(5-(3,4-dimethoxyphenyl)-3-hydroxy-1*H*-pyrrol-2-yl)methanone (5o), ^{1}H NMR, $CDCl_3$, 400 MHz

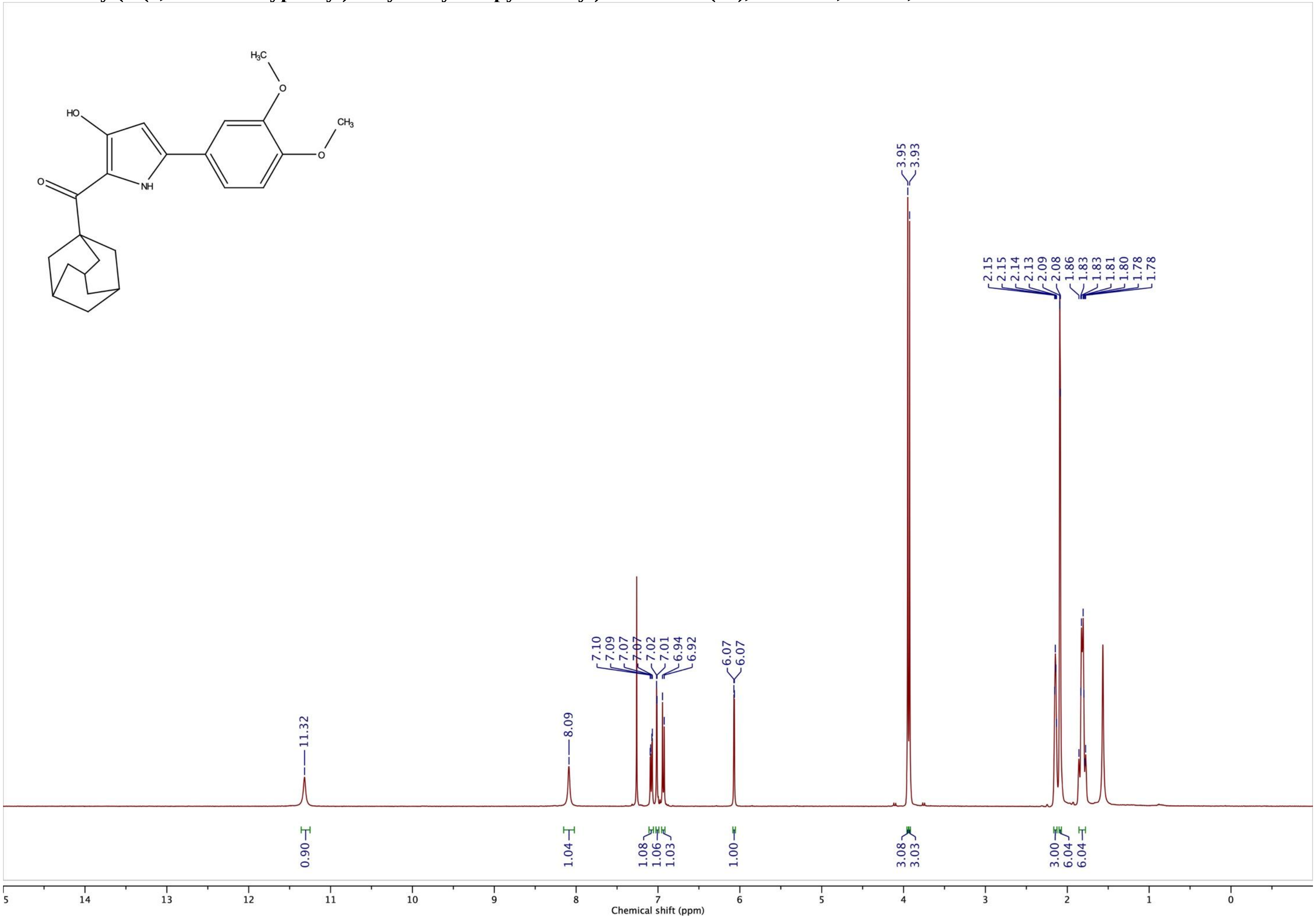

Adamantan-1-yl(5-(3,4-dimethoxyphenyl)-3-hydroxy-1*H*-pyrrol-2-yl)methanone (5o), $^{13}C\{^{1}H\}$ NMR, $CDCl_3$, 100 MHz

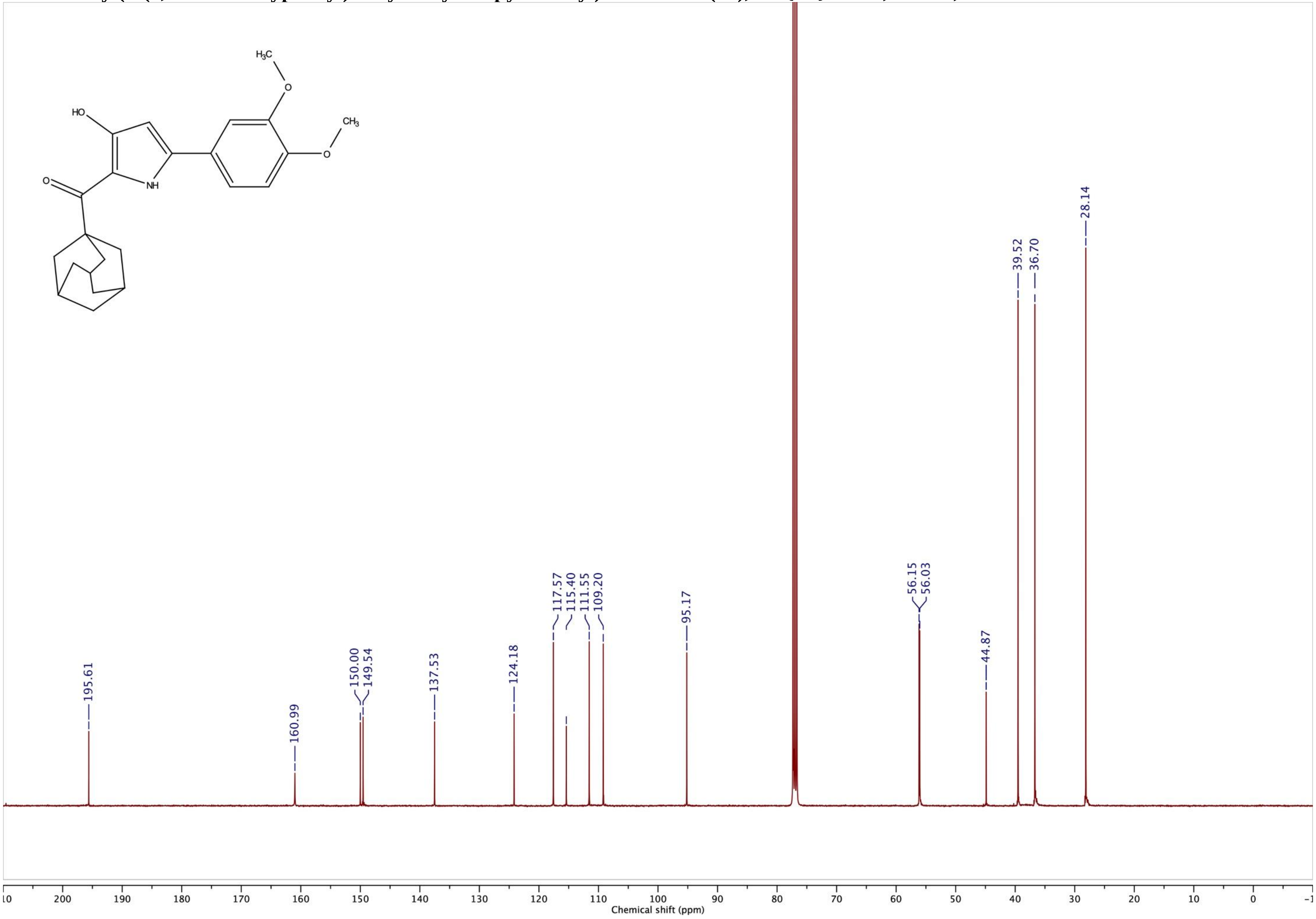

Adamantan-1-yl(5-(3,4-dimethoxyphenyl)-3-hydroxy-1*H*-pyrrol-2-yl)methanone (5o), DEPT, $CDCl_3$, 100 MHz

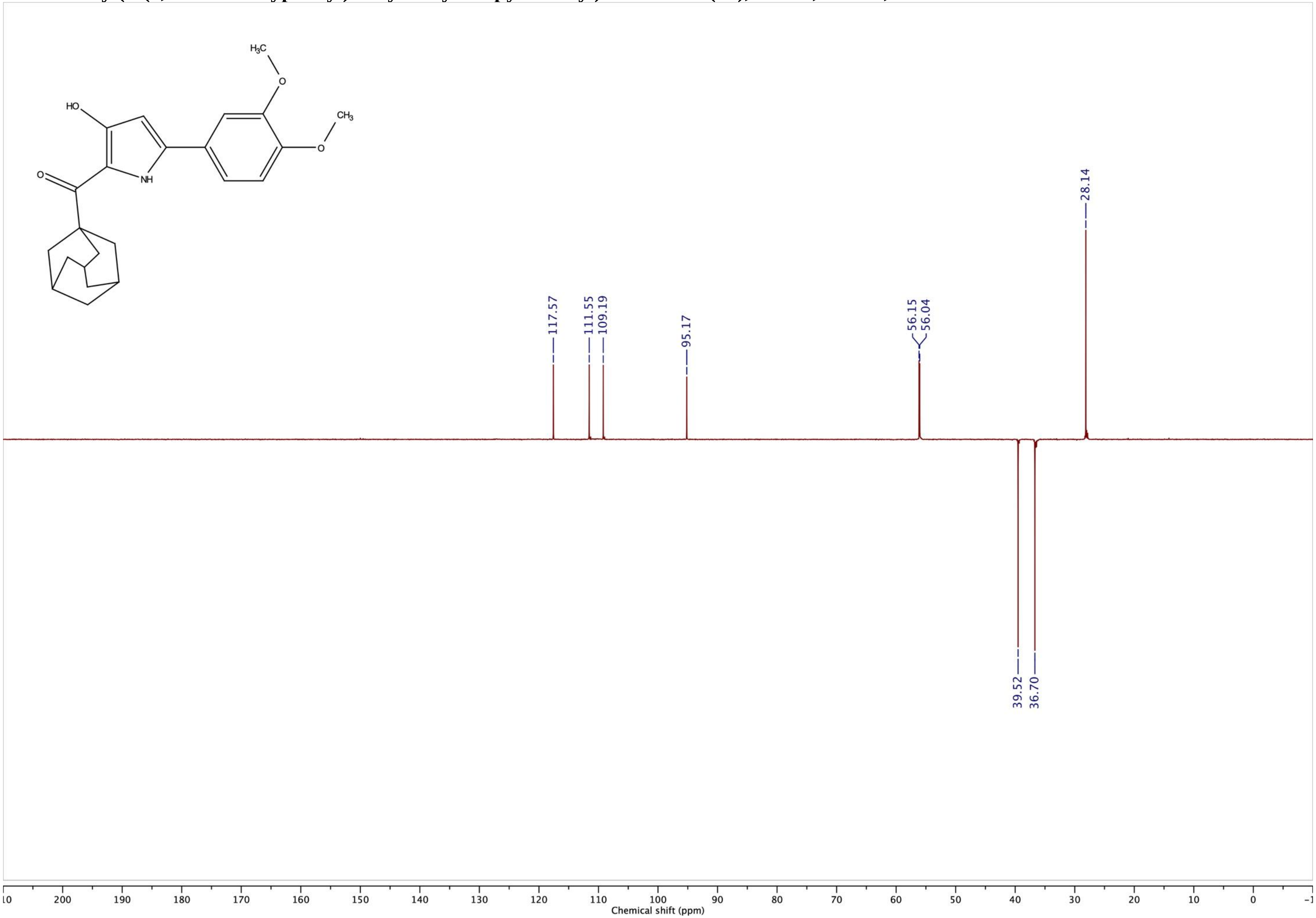

(3-Hydroxy-5-phenyl-1*H*-pyrrol-2-yl)(thiophen-2-yl)methanone (5p), ^{1}H NMR, $CDCl_3$, 400 MHz

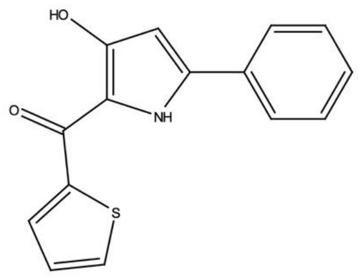

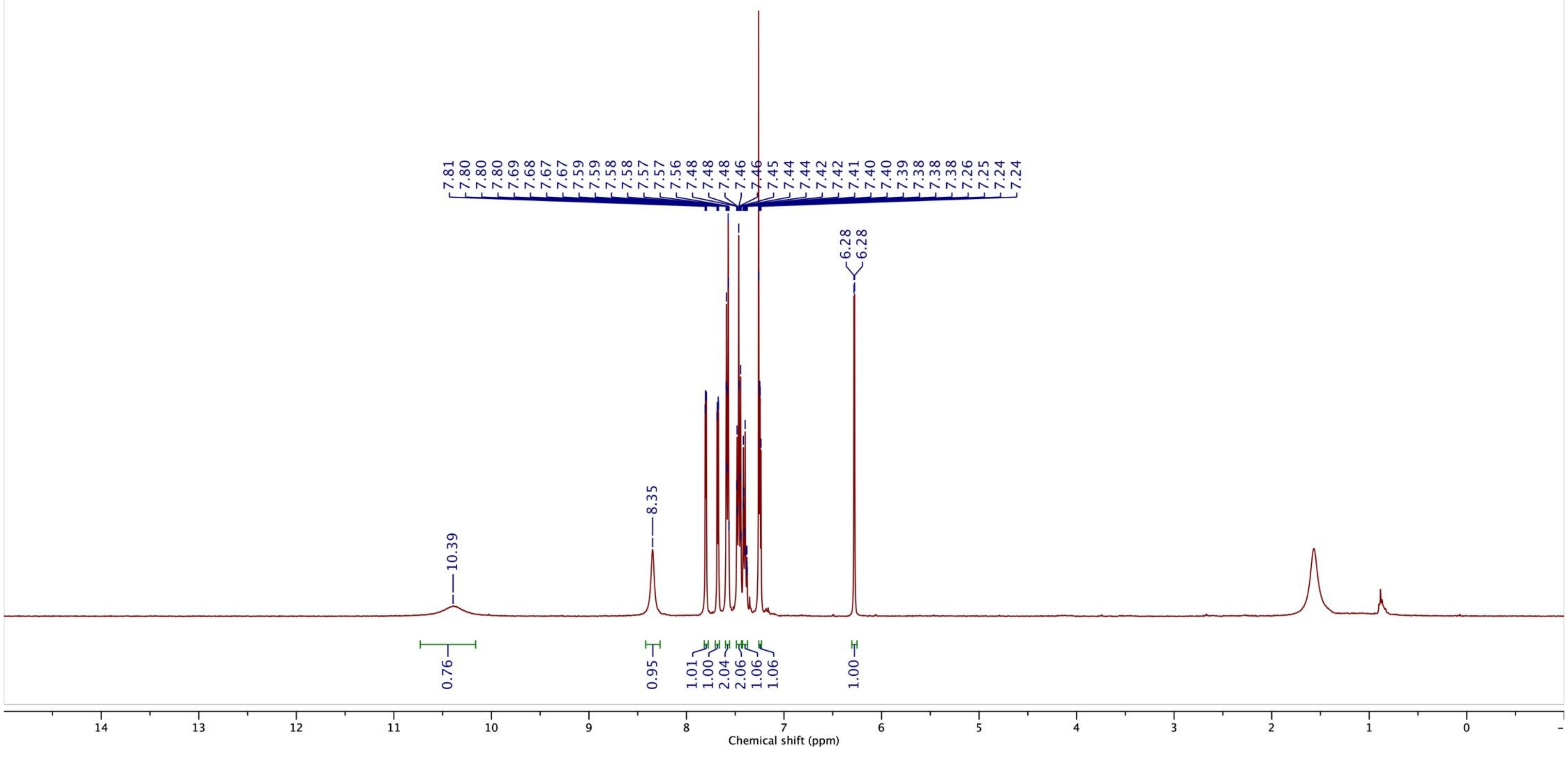

(3-Hydroxy-5-phenyl-1*H*-pyrrol-2-yl)(thiophen-2-yl)methanone (5p), $^{13}C\{^{1}H\}$ NMR, $CDCl_3$, 100 MHz

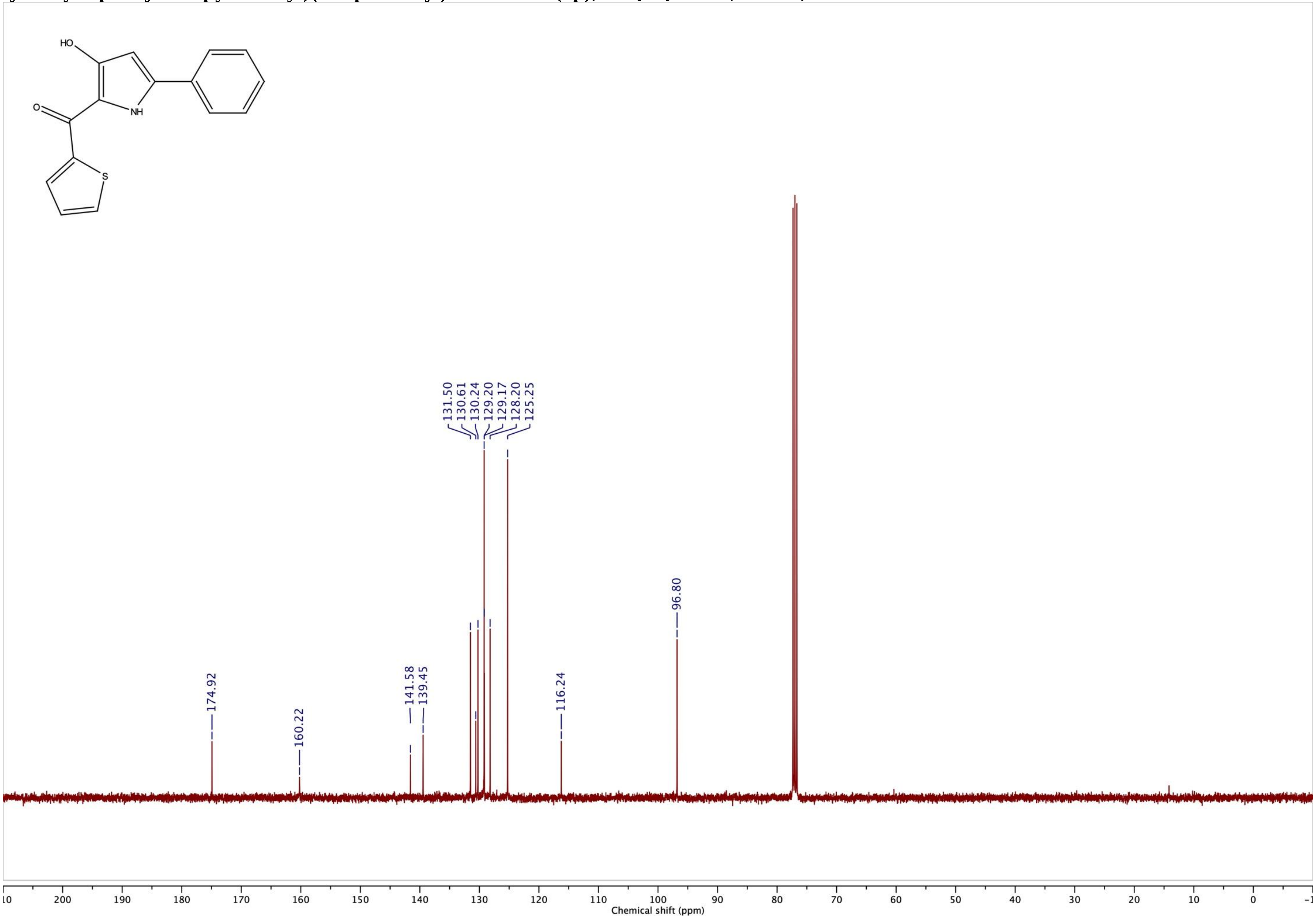

(3-Hydroxy-5-phenyl-1*H*-pyrrol-2-yl)(thiophen-2-yl)methanone (5p), DEPT, $CDCl_3$, 100 MHz

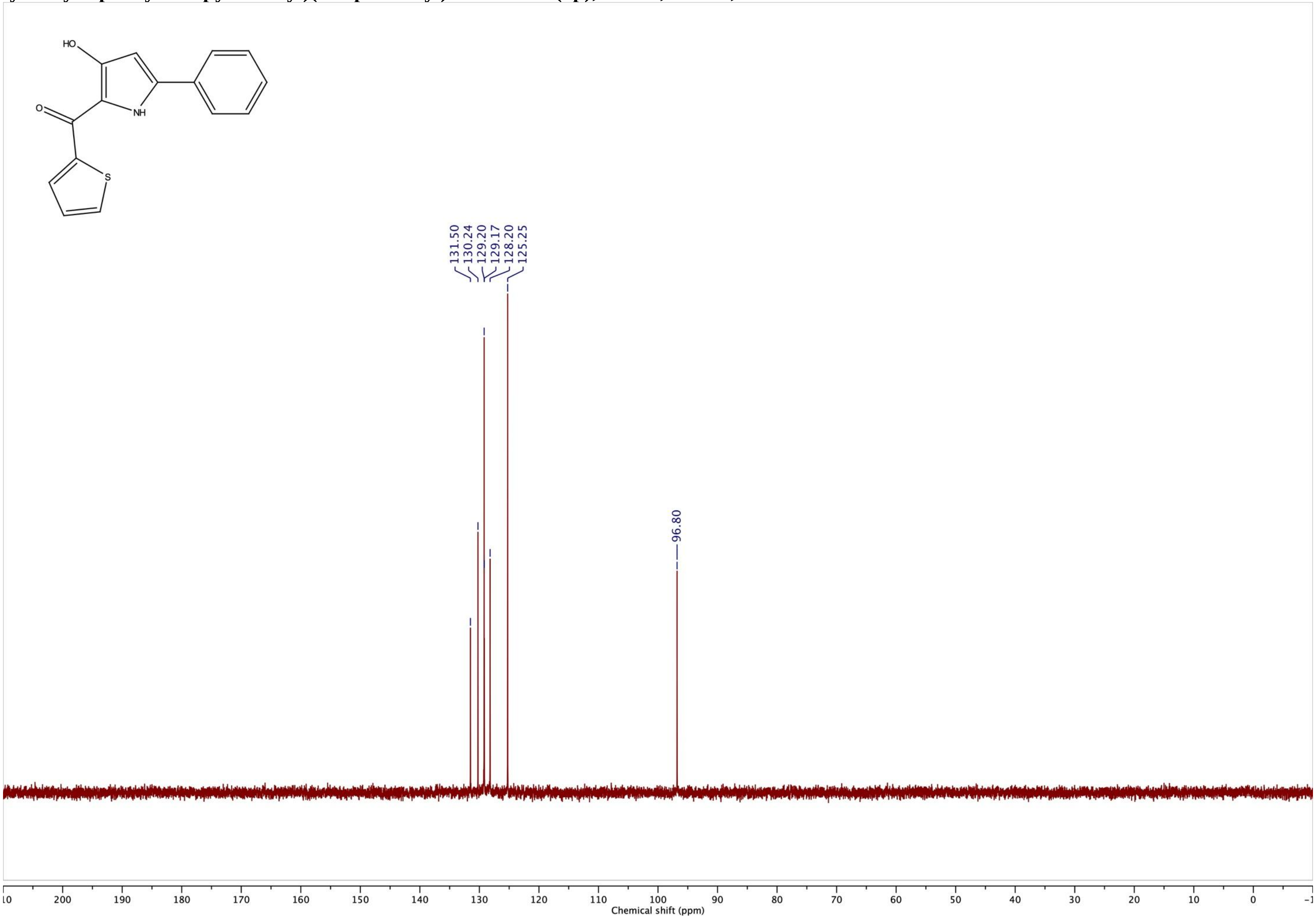

Ethyl 2-((2-benzoyl-5-(4-fluorophenyl)-1*H*-pyrrol-3-yl)oxy)acetate (6a), ^{1}H NMR, DMSO-d_6, 400 MHz

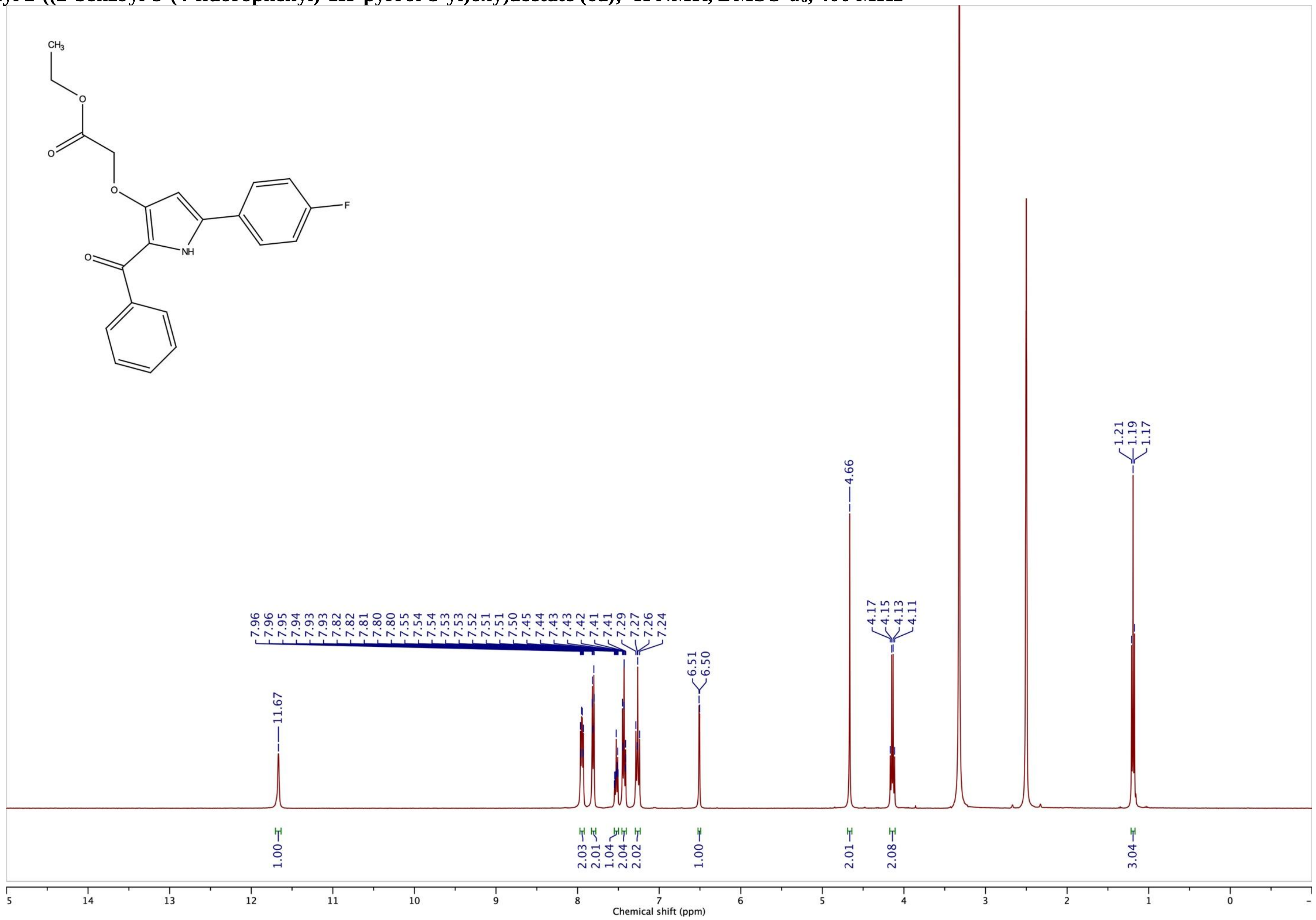

Ethyl 2-((2-benzoyl-5-(4-fluorophenyl)-1*H*-pyrrol-3-yl)oxy)acetate (6a), $^{13}C\{^{1}H\}$ NMR, DMSO-*d*$_6$, 100 MHz

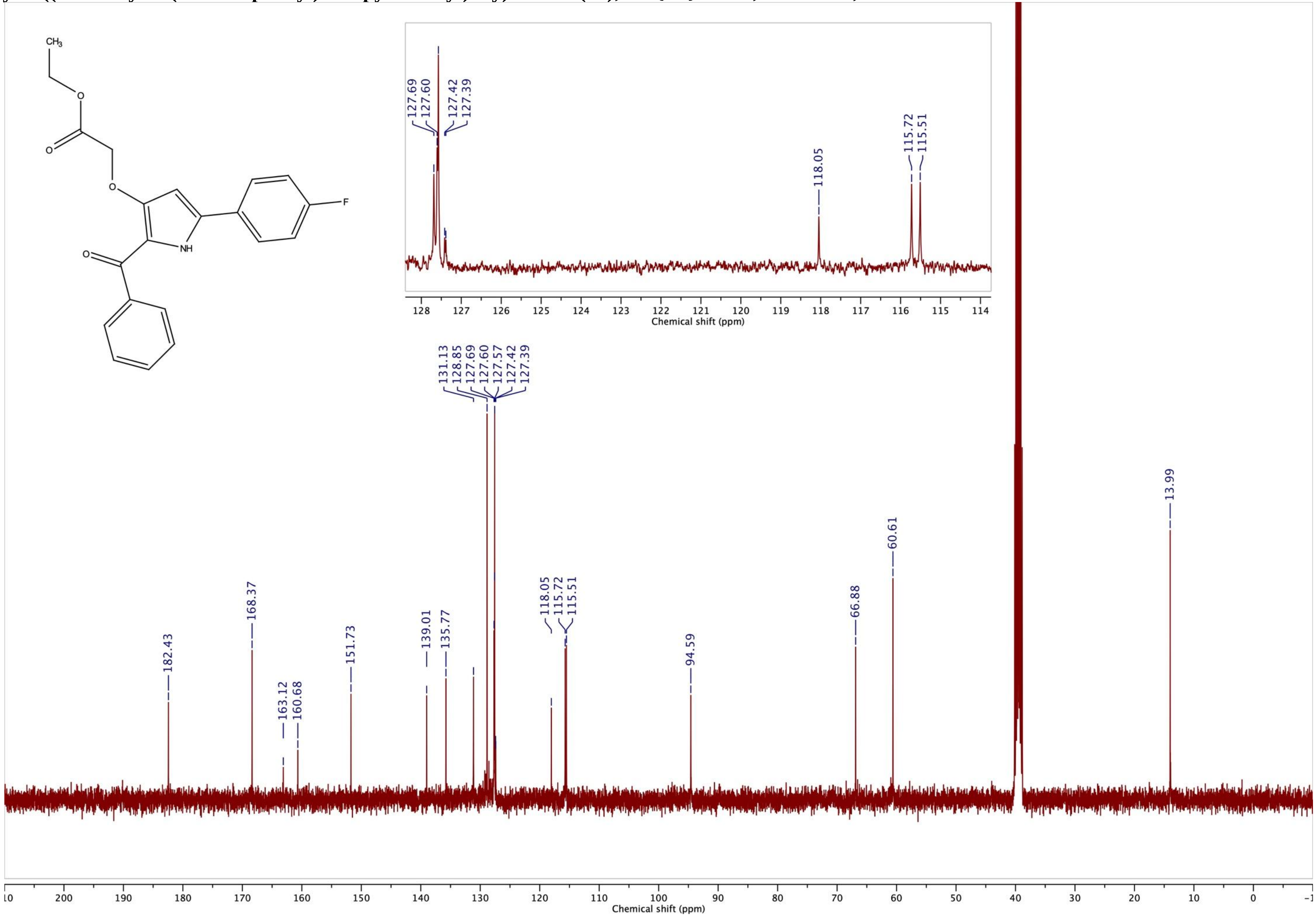

Ethyl 2-((2-benzoyl-5-(4-fluorophenyl)-1*H*-pyrrol-3-yl)oxy)acetate (6a), DEPT, DMSO-*d*$_6$, 100 MHz

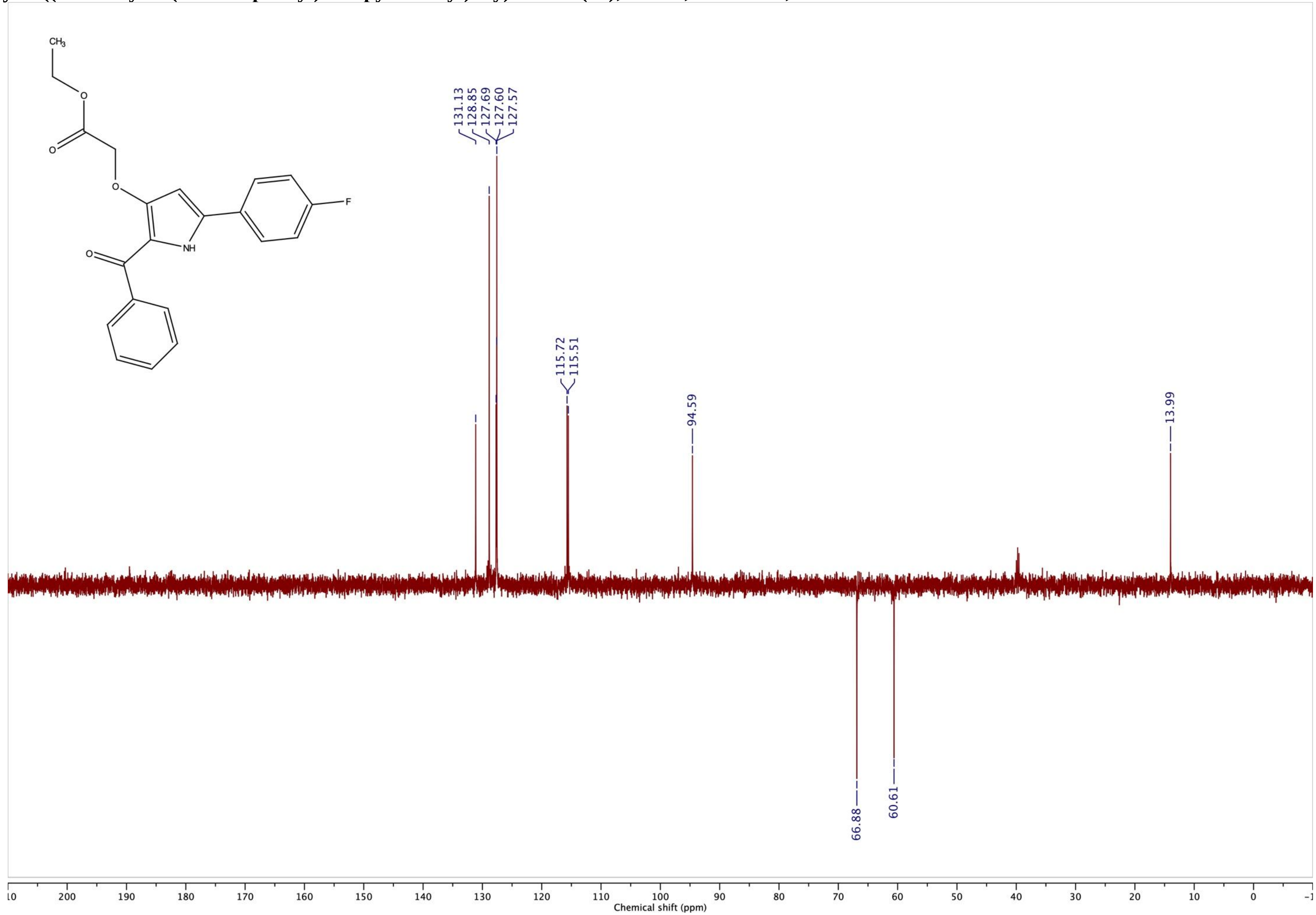

2-((2-Benzoyl-5-(4-fluorophenyl)-1*H*-pyrrol-3-yl)oxy)-1-phenylethan-1-one (6b), ^{1}H NMR, DMSO-d_6, 400 MHz

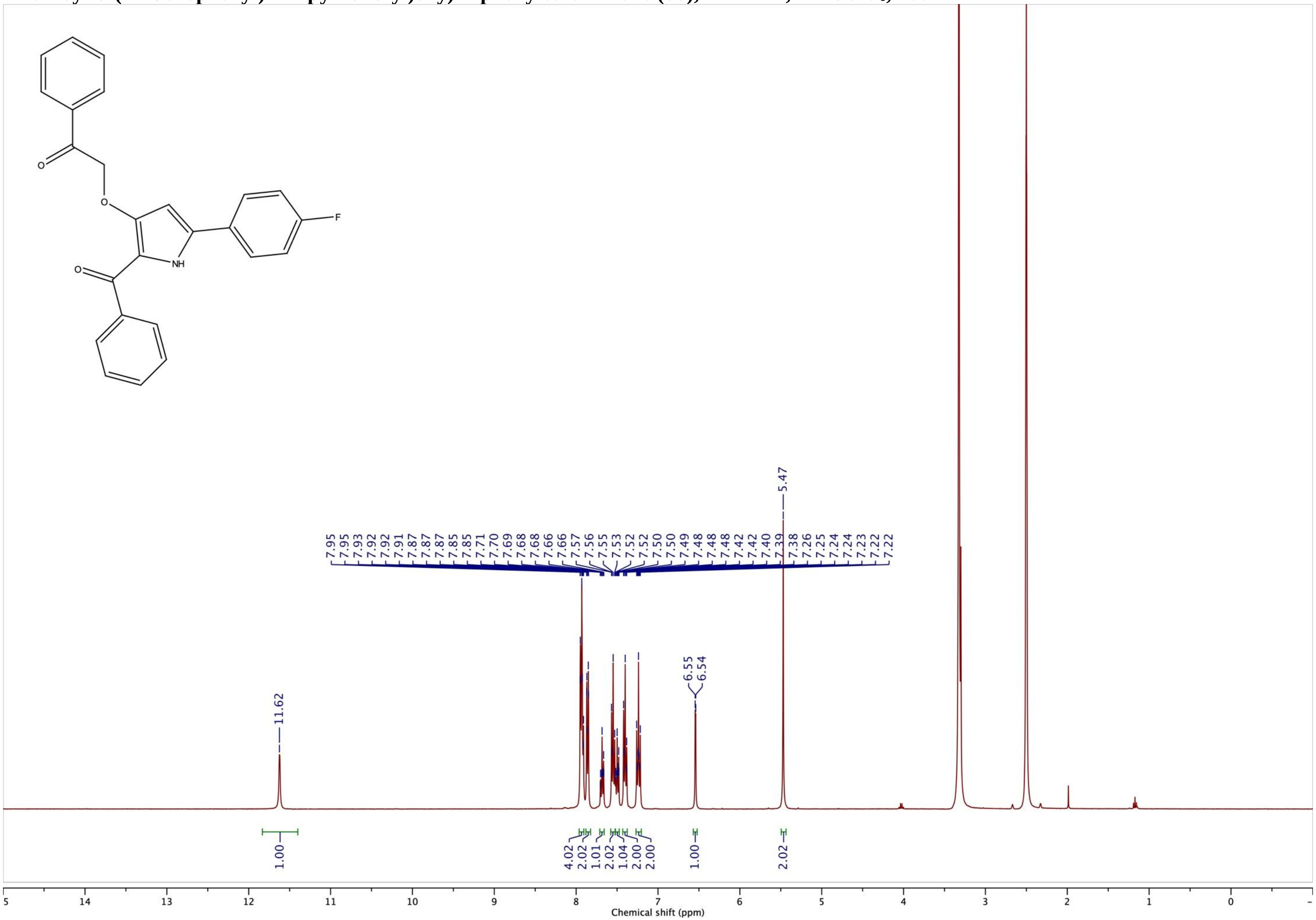

2-((2-Benzoyl-5-(4-fluorophenyl)-1H-pyrrol-3-yl)oxy)-1-phenylethan-1-one (6b), $^{13}C\{^1H\}$ NMR, DMSO-d_6, 100 MHz

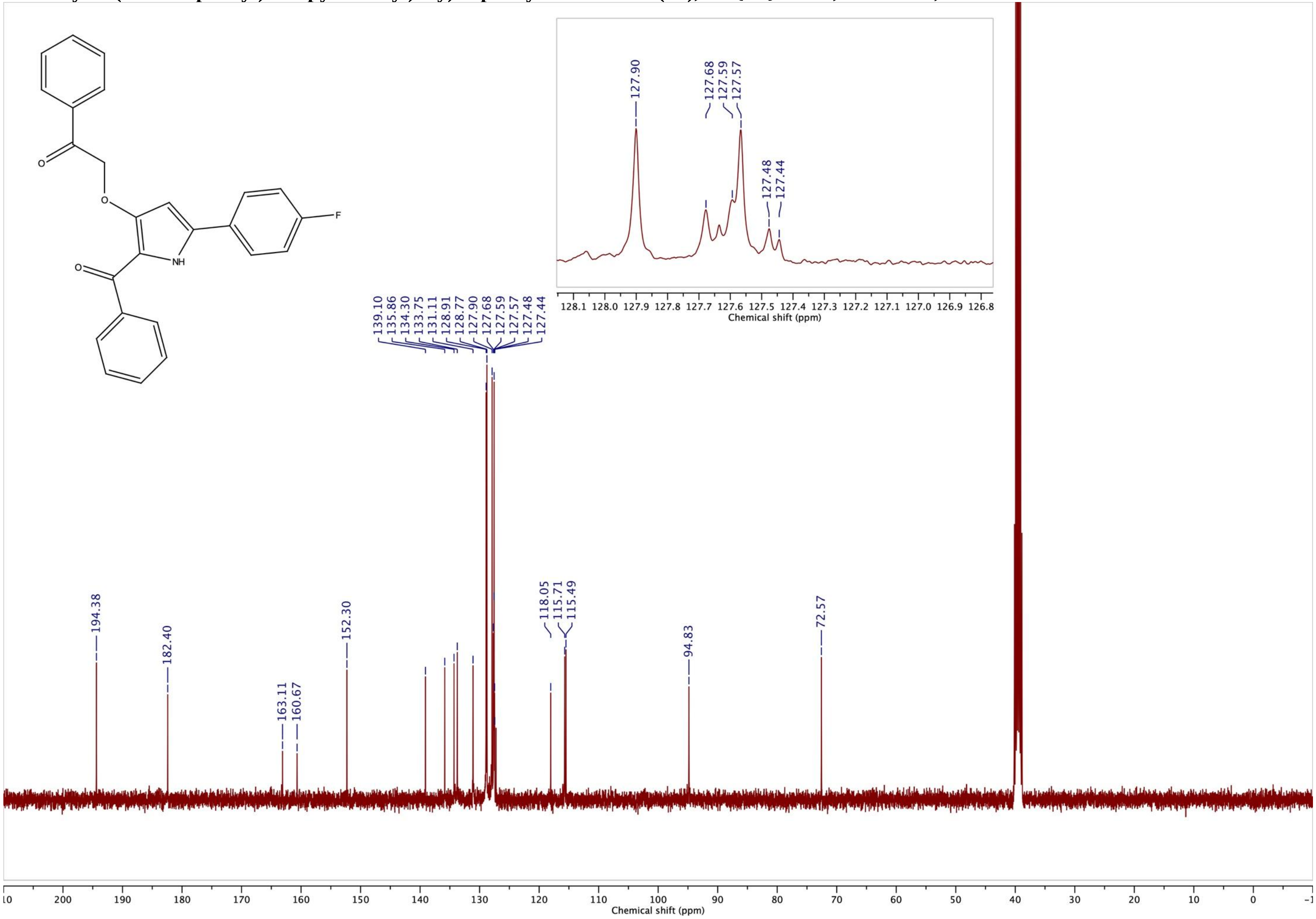

2-((2-Benzoyl-5-(4-fluorophenyl)-1*H*-pyrrol-3-yl)oxy)-1-phenylethan-1-one (6b), DEPT, DMSO-d_6, 100 MHz

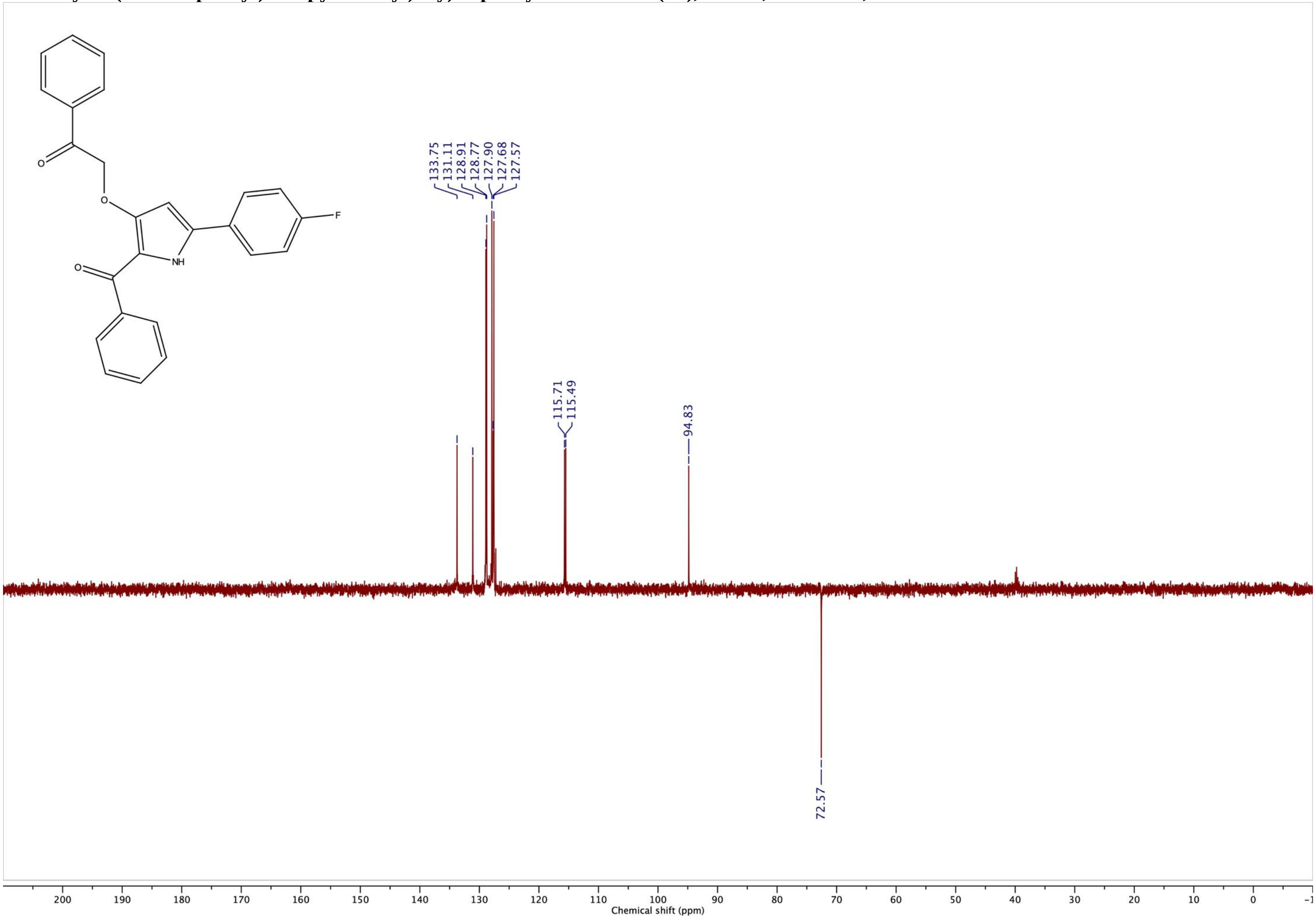

2-Benzoyl-5-(4-fluorophenyl)-1*H*-pyrrol-3-yl trifluoromethanesulfonate (7a), ^{1}H NMR, $CDCl_3$, 400 MHz

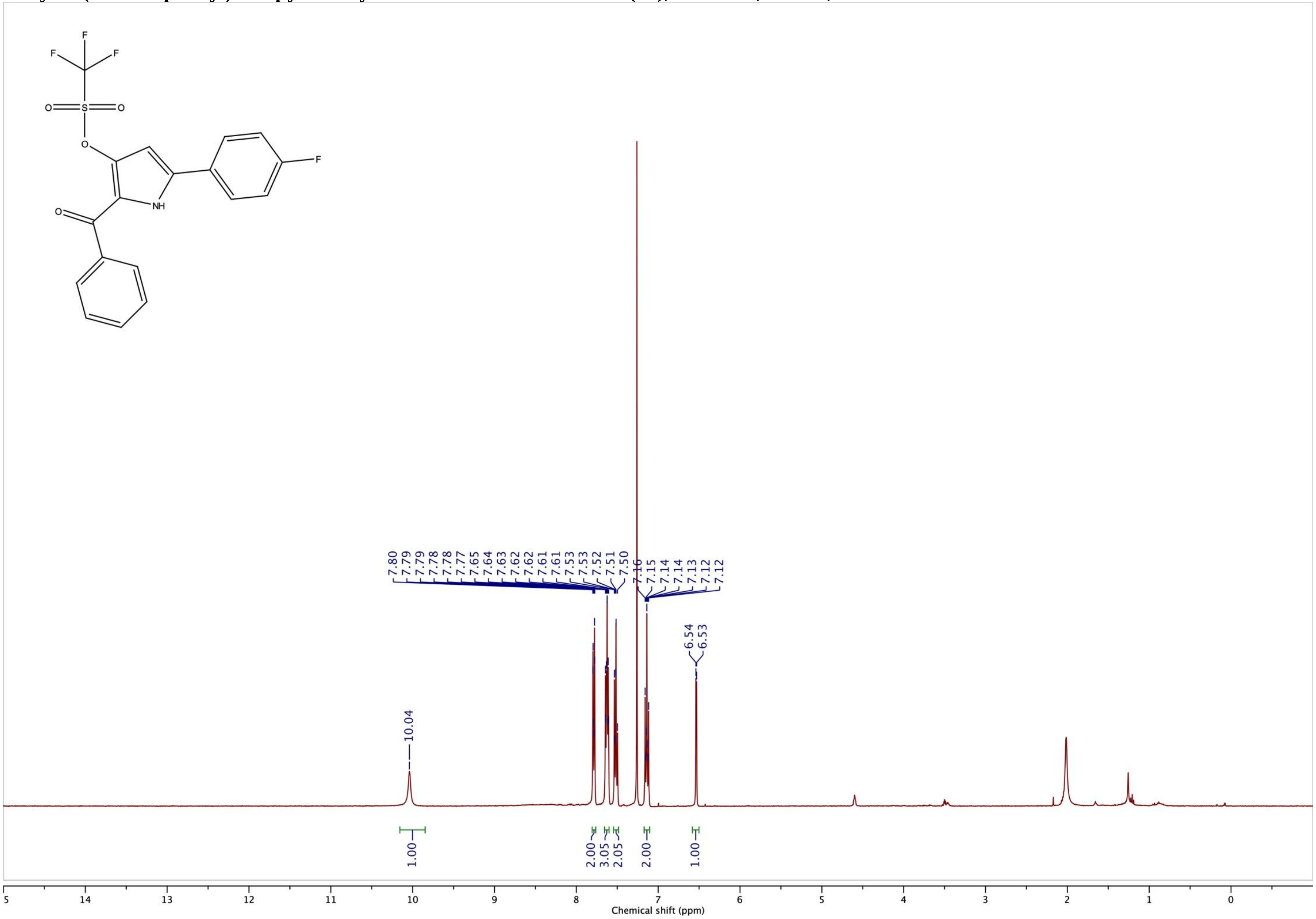

2-Benzoyl-5-(4-fluorophenyl)-1*H*-pyrrol-3-yl trifluoromethanesulfonate (7a), $^{13}C\{^{1}H\}$ NMR, $CDCl_3$, 100 MHz

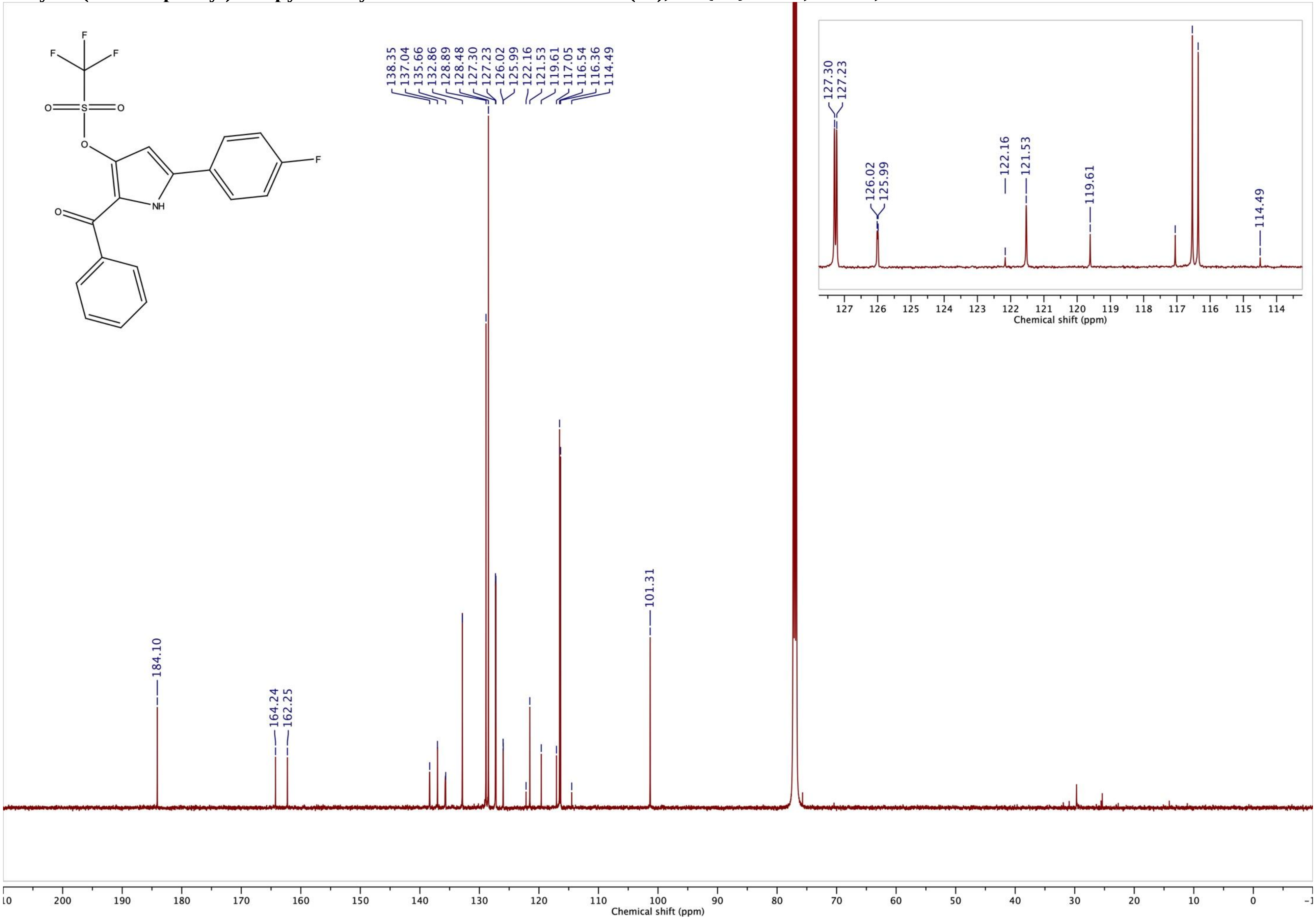

2-Benzoyl-5-(4-fluorophenyl)-1*H*-pyrrol-3-yl trifluoromethanesulfonate (7a), DEPT, $CDCl_3$, 100 MHz

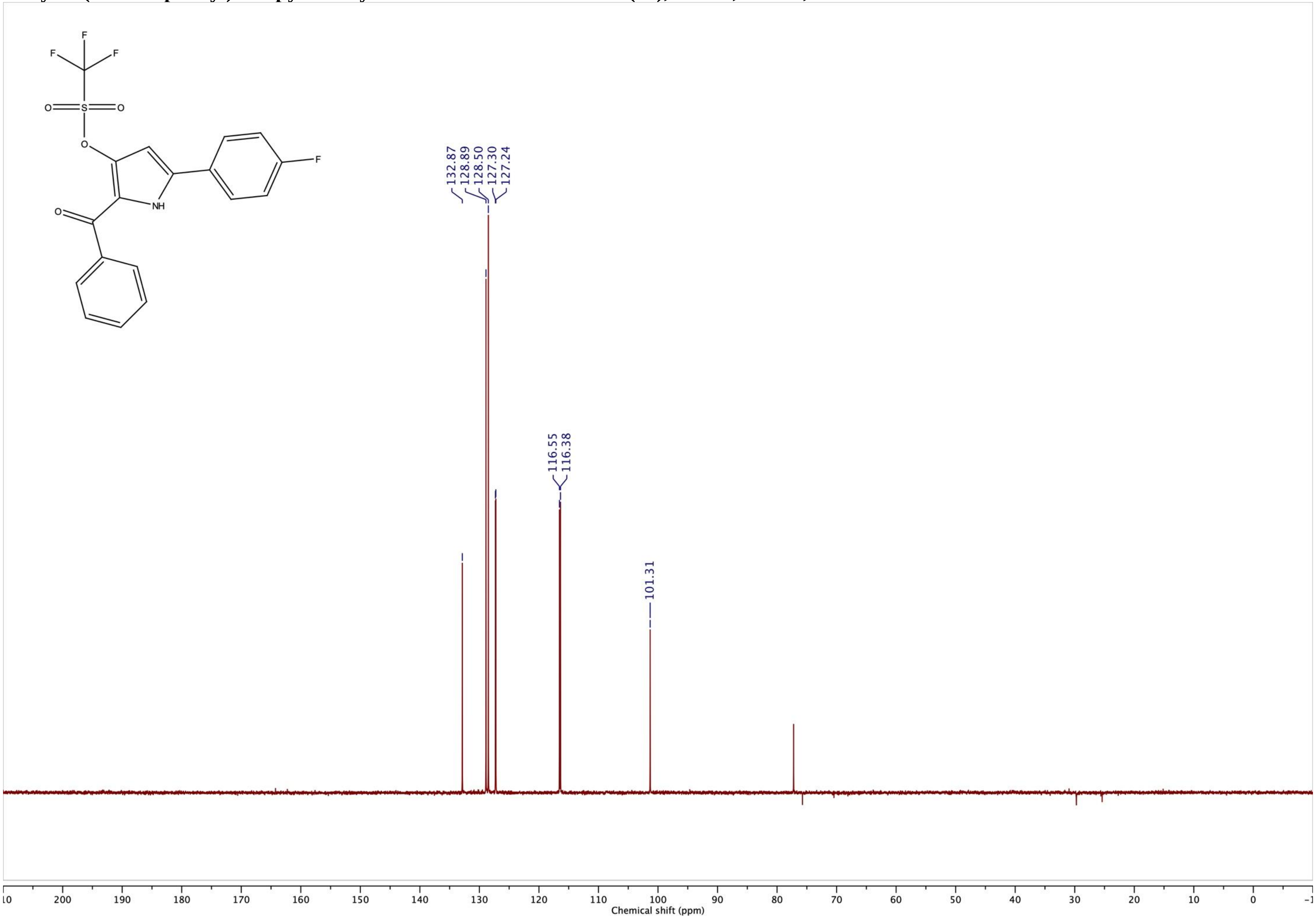

8. IR spectrum of compound 4b

(1*RR*,3*SR*,6*RR*)-4-Diazo-3-(4-iodophenyl)-1-(3-methoxyphenyl)-2-oxa-7-azabicyclo[4.1.0]heptan-5-one (4b), IR, KBr

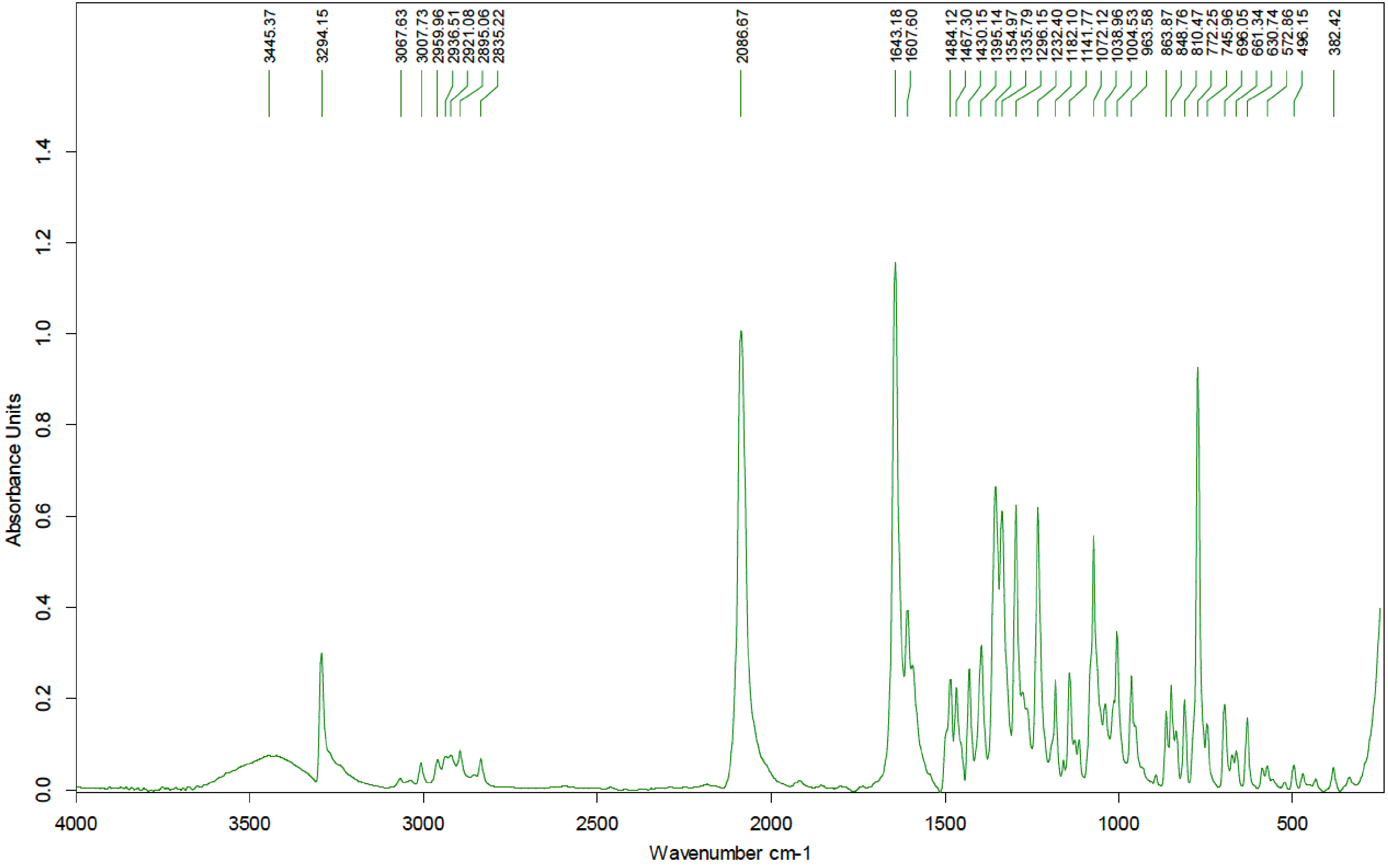

9. Computational details

All calculations were performed by using the Gaussian 16 suite of quantum chemical programs [11] at Resource center "Computer center of Saint Petersburg State University". Geometry optimizations of molecules were performed with the B3LYP-D3 [12,13] density functional method and 6-311+G(d,p) for H, C, N, O, S and LANL2DZ for Cs basis set using SMD [14] solvent model for MeCN. Stationary points on the respective potential-energy surfaces were characterized at the same level of theory by evaluating the corresponding Hessian indices. Careful verification of the unique imaginary frequencies for transition states was carried out to check whether the frequency indeed pertains to the desired reaction coordinate.

Table S8. Absolute Energies (au), Cartesian Coordinates of stationary points

Molecule **1a**

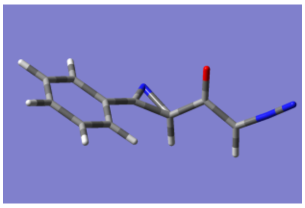

E = -624.842788, H (0K) = -624.692423,
H (298K) = -624.680396,
G (298K) = -624.732869 au.
Imaginary frequency = 0.

N	0.1535660	2.1418480	-0.1844330
C	0.9735200	0.9921690	-0.8197530
C	2.0209370	0.3681980	0.0456220
O	1.9158670	0.3165810	1.2704370
C	3.1516100	-0.1696720	-0.6881320
H	1.1935240	1.0850710	-1.8790840
N	4.0918420	-0.7633600	0.0029620
N	4.8657780	-1.2607050	0.6523620
C	-1.6417980	0.3077180	-0.1629270
C	-4.0098760	-1.1080900	0.1980380
C	-3.9393120	0.2370610	0.5749300
C	-2.7591680	0.9483820	0.3974260
C	-1.7133860	-1.0400940	-0.5404890
C	-2.8995550	-1.7454090	-0.3581770
H	-2.6921570	1.9910400	0.6865610
H	-4.8051420	0.7256760	1.0067890
H	-4.9332110	-1.6587220	0.3395890
H	-2.9588640	-2.7881400	-0.6477480
H	-0.8422100	-1.5215900	-0.9699940
H	3.2893650	-0.1254190	-1.7588600

Molecule **2c**

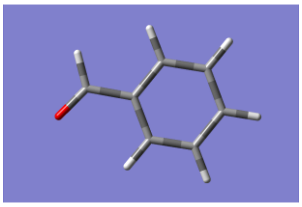

E = -345.690330, H (0K) = -345.580953,
H (298K) = -345.573677,
G (298K) = -345.611546 au.
Imaginary frequency = 0.

C	-1.9874290	0.4657850	0.0000220
O	-2.8519050	-0.3934010	-0.0000230
H	-2.2644570	1.5376200	-0.0000510
C	-0.5355200	0.2031200	0.0000150
C	0.3510190	1.2883970	0.0000100
C	-0.0361400	-1.1085550	0.0000110
C	1.7264170	1.0668920	-0.0000090
H	-0.0413170	2.3004780	0.0000190
C	1.3357370	-1.3268530	0.0000020
H	-0.7311290	-1.9404310	0.0000180
C	2.2171120	-0.2394330	-0.0000100
H	2.4125090	1.9061930	-0.0000160
H	1.7249550	-2.3387590	0.0000030
H	3.2875020	-0.4140140	-0.0000280

Molecule **Cs_2CO_3**

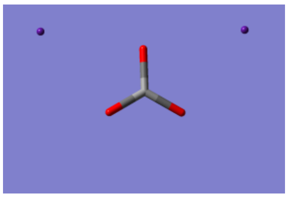

E = -303.846118, H (0K) = -303.831580,
H (298K) = -303.822719,
G (298K) = -303.872026 au.
Imaginary frequency = 0.

C	0.0006250	1.4733050	-0.0006650
O	0.0006330	0.1663930	-0.0030430
O	-1.1249490	2.1122580	-0.0827030
O	1.1263550	2.1119340	0.0837690
Cs	3.1475850	-0.3997790	-0.0041490

Molecule **$CsHCO_3$**

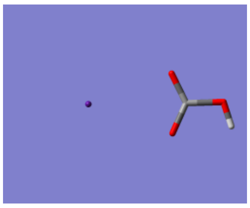

E = -284.502506, H (0K) = -284.476132,
H (298K) = -284.469202,
G (298K) = -284.510013 au.
Imaginary frequency = 0.

C	2.3080950	0.0330890	-0.0002350
O	1.7948570	-1.1099260	-0.0001710
O	1.7602120	1.1504770	-0.0000570
O	3.7175950	0.0761650	-0.0003980

Cs	-3.1479490	-0.3995760	0.0045100

H	4.0153180	-0.8433500	-0.0002100
Cs	-1.3826400	-0.0052530	0.0001200

TS **TS^{RS}-1**

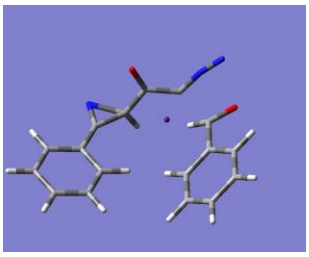

E = -989.896632, H (0K) = -989.647164,
H (298K) = -989.625111,
G (298K) = -989.703325 au.
Imaginary frequency = 1.

C	-1.8265620	-2.3073440	-0.7224110
N	-1.6632560	-3.1846970	-1.6061330
C	-0.4093350	-2.5994390	-0.8998140
C	0.4480070	-1.6735360	-1.7109300
O	-0.0350550	-1.0806090	-2.6921430
C	1.8023100	-1.5425800	-1.2576930
H	0.0884250	-3.2584330	-0.1973040
N	2.6336590	-0.7792330	-1.8582390
N	3.5449820	-0.1858410	-2.2183280
C	-2.8249690	-1.5175620	-0.0394230
C	-4.7244060	0.0224970	1.2938410
C	-5.1233290	-0.8439930	0.2706570
C	-4.1795620	-1.6158470	-0.3966670
C	-2.4271370	-0.6488770	0.9854780
C	-3.3786860	0.1186310	1.6515120
H	-4.4784130	-2.2897430	-1.1916340
H	-6.1699370	-0.9144400	-0.0028040
H	-5.4654190	0.6215640	1.8114440
H	-3.0721370	0.7887930	2.4465550
H	-1.3792370	-0.5849940	1.2525940
Cs	-0.0729820	3.0075940	-0.6787420
C	2.8210540	-2.2409860	0.3180510
O	4.0520910	-2.2862460	0.0343000
H	2.2378750	-3.1792690	0.3412960
C	2.3336040	-1.2417900	1.3486650
C	1.1870760	-1.4921010	2.1102630
C	3.0568290	-0.0646900	1.5793070
C	0.7604180	-0.5780960	3.0746130
H	0.6262340	-2.4065240	1.9470440
C	2.6277510	0.8543770	2.5352670
H	3.9588470	0.1131080	1.0047390
C	1.4759250	0.6018510	3.2864860
H	-0.1284110	-0.7870350	3.6606010
H	3.1931800	1.7656580	2.7017020
H	1.1450950	1.3138050	4.0350310

TS **TS^{RS}-1**

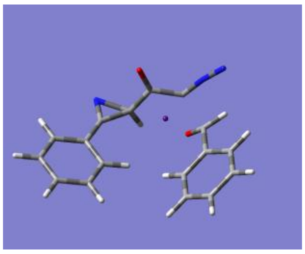

E = -989.896470, H (0K) = -989.646915,
H (298K) = -989.624862,
G (298K) = -989.703020 au.
Imaginary frequency = 1.

C	-1.8199900	-2.2405340	-0.7274560
N	-1.7130690	-3.1008760	-1.6355040
C	-0.4161940	-2.5593830	-0.9618590
C	0.4253820	-1.6281400	-1.7783280
O	-0.0739620	-0.9802400	-2.7141660
C	1.8032060	-1.5527850	-1.3721650
H	0.1077520	-3.2373740	-0.2969940
N	2.5972310	-0.7204640	-1.9253010
N	3.4292000	-0.0218690	-2.2973580
C	-2.7751560	-1.4464590	0.0113870
C	-4.5912890	0.0993250	1.4507540
C	-5.0424240	-0.7129960	0.4051160
C	-4.1404180	-1.4878320	-0.3146170
C	-2.3249320	-0.6316670	1.0587130
C	-3.2349790	0.1383640	1.7779860
H	-4.4800000	-2.1200880	-1.1273020
H	-6.0969410	-0.7391470	0.1551830
H	-5.2997050	0.7010140	2.0093440
H	-2.8878900	0.7668430	2.5902730
H	-1.2691830	-0.6106100	1.3009580
Cs	-0.0572310	3.0602540	-0.6128880
C	2.6811090	-2.4389790	0.2304240
O	2.2070140	-3.5906000	0.4257340
H	3.6805660	-2.3384340	-0.2344750
C	2.4125710	-1.3248130	1.2202900
C	3.2754250	-0.2244230	1.3041330
C	1.3169270	-1.3957390	2.0881320
C	3.0361670	0.7957220	2.2255090
H	4.1389400	-0.1710490	0.6484140
C	1.0732190	-0.3753860	3.0052840
H	0.6616090	-2.2570240	2.0314840
C	1.9312480	0.7258510	3.0766700
H	3.7140040	1.6409880	2.2837530
H	0.2162500	-0.4377800	3.6679850
H	1.7451320	1.5170940	3.7949480

Molecule **(RR,SR)-A**

Molecule **(RR,RR)-A**

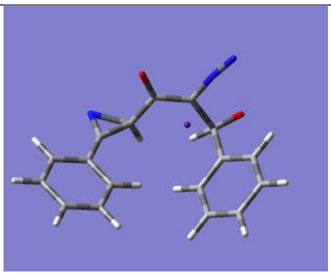

E = -989.899490, H (0K) = -989.648946,
H (298K) = -989.626744,
G (298K) = -989.704704 au.
Imaginary frequency = 0.

C	-1.7975790	-2.3933380	-0.5319030
N	-1.6337500	-3.3174470	-1.3655260
C	-0.3815780	-2.7160090	-0.6734890
C	0.4862150	-1.8462700	-1.5317960
O	0.0245320	-1.3375710	-2.5642520
C	1.8471850	-1.6383900	-1.0881190
H	0.0959400	-3.3410180	0.0713100
N	2.5835890	-0.8732130	-1.8353760
N	3.3709280	-0.2567890	-2.3712090
C	-2.7908080	-1.5539690	0.0964940
C	-4.6848410	0.0778930	1.3230980
C	-5.0769090	-0.8068910	0.3130780
C	-4.1360470	-1.6251740	-0.3007270
C	-2.3992800	-0.6662220	1.1075790
C	-3.3487110	0.1465500	1.7207900
H	-4.4294980	-2.3133370	-1.0853370
H	-6.1158810	-0.8552430	0.0077570
H	-5.4236050	0.7131780	1.7990140
H	-3.0476300	0.8313240	2.5053350
H	-1.3586500	-0.6222910	1.4050500
Cs	-0.1811540	2.9220940	-0.8173020
C	2.6525970	-2.1371690	0.2597430
O	3.9379620	-2.3543690	0.0425210
H	2.0588700	-3.0367120	0.5310820
C	2.3341450	-1.0556370	1.3134900
C	1.2390040	-1.1703500	2.1757140
C	3.1591860	0.0703960	1.4253940
C	0.9634510	-0.1794750	3.1215790
H	0.5959410	-2.0423390	2.1080140
C	2.8844800	1.0665970	2.3615160
H	4.0259710	0.1419380	0.7776190
C	1.7827290	0.9464080	3.2145500
H	0.1125800	-0.2887650	3.7864170
H	3.5319030	1.9349760	2.4334220
H	1.5727980	1.7170790	3.9486260

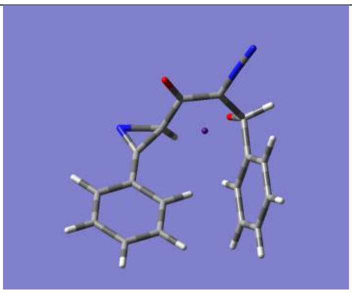

E = -989.901622, H (0K) = -989.650734,
H (298K) = -989.628761,
G (298K) = -989.705605 au.
Imaginary frequency = 0.

C	0.6366900	-1.8067410	-1.8287840
N	1.1558470	-1.7151440	-2.9684240
C	1.7253660	-0.8378080	-1.8186190
C	1.3982750	0.6215750	-1.8758020
O	0.5204650	1.0512130	-2.6339930
C	2.1541360	1.4676100	-0.9705690
H	2.7000520	-1.1002640	-1.4235330
N	1.8661610	2.7309510	-1.0000840
N	1.6746160	3.8528360	-0.9776210
C	-0.4077500	-2.4459950	-1.0639090
C	-2.4137250	-3.6482730	0.4462530
C	-2.3219850	-3.9107200	-0.9249290
C	-1.3210460	-3.3148180	-1.6830030
C	-0.5010390	-2.1830040	0.3094600
C	-1.5035190	-2.7880270	1.0624090
H	-1.2407800	-3.5111880	-2.7462330
H	-3.0317820	-4.5800700	-1.3974510
H	-3.1961390	-4.1172420	1.0326040
H	-1.5743420	-2.5882650	2.1254810
H	0.2108190	-1.5116790	0.7736650
Cs	-2.7951510	1.7579170	0.2896350
C	3.3111260	1.0082680	0.0563030
O	4.2925900	0.3540620	-0.5477560
H	3.6089860	1.9842710	0.5125960
C	2.5964460	0.2441140	1.2052320
C	1.5929430	0.8500810	1.9726020
C	2.9834990	-1.0581190	1.5217420
C	0.9855230	0.1668340	3.0252540
H	1.2853720	1.8672300	1.7452290
C	2.3788470	-1.7492080	2.5754460
H	3.7657600	-1.5141640	0.9259130
C	1.3769910	-1.1404180	3.3308200
H	0.2085040	0.6508720	3.6084180
H	2.6877470	-2.7644070	2.8036670
H	0.9036570	-1.6753520	4.1471850

Molecule **(RR,SR)-B**

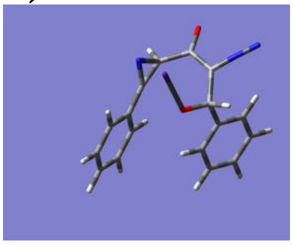

E = -989.889812, H (0K) = -989.639371,

Molecule **(RR,RR)-B**

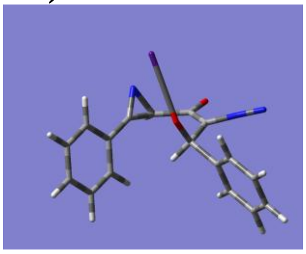

E = -989.894321, H (0K) = -989.643632,

H (298K) = -989.617256,
G (298K) = -989.695551 au.
Imaginary frequency = 0.

C	0.4592230	-1.1979440	-0.7677560
C	-0.0906100	0.9921670	1.5182440
N	-1.1859670	0.6340180	2.0359920
C	0.0875920	-0.2026750	2.3356630
C	0.0796100	-1.6428560	1.9172420
O	0.0481280	-2.4852960	2.8257110
C	0.0571170	-2.0379260	0.5180080
H	0.4603110	-0.1009650	3.3530370
H	0.1346870	-1.8619380	-1.6057580
C	2.0073320	-1.1701690	-0.8673100
N	-0.2327020	-3.2901410	0.3134980
N	-0.5059600	-4.3627440	0.0582270
C	0.5320290	2.1646910	0.9356340
O	-0.1250530	0.0030550	-0.7784260
C	1.7277570	4.4611820	-0.1035450
C	2.5115890	3.4865120	0.5150750
C	1.9165660	2.3375200	1.0314020
C	-0.2577350	3.1524700	0.3302730
C	0.3420050	4.2921840	-0.1940110
C	2.7989090	-2.3093280	-0.6688440
C	4.1854360	-2.2501850	-0.8099160
C	4.8056710	-1.0462060	-1.1555020
C	4.0244620	0.0906890	-1.3627380
C	2.6363240	0.0243510	-1.2222490
H	2.5197150	1.5671010	1.4968130
H	3.5855930	3.6164830	0.5872200
H	2.1920800	5.3516190	-0.5129750
H	-0.2667340	5.0516930	-0.6721570
H	-1.3312010	3.0169200	0.2653420
H	2.0152730	0.8981820	-1.3688320
H	4.4951580	1.0319040	-1.6291500
H	5.8842930	-0.9981680	-1.2613710
H	4.7825730	-3.1416620	-0.6471660
H	2.3373660	-3.2540870	-0.3993150
Cs	-3.2665630	0.0535640	-0.7733820

H (298K) = -989.621438,
G (298K) = -989.700244 au.
Imaginary frequency = 0.

C	1.1729010	-0.2460310	-0.0583810
C	-1.4229210	-1.0039390	-1.6112030
N	-1.9242430	0.0634340	-2.0520930
C	-0.6317320	-0.4844760	-2.7203930
C	0.5856330	0.4011550	-2.6605740
O	0.8993890	0.9842230	-3.7084710
C	1.3295980	0.5663370	-1.4292870
H	-0.7799620	-0.8898320	-3.7184960
N	2.2188530	1.5155920	-1.4400640
N	3.0132460	2.3217860	-1.3523110
C	-1.6846200	-2.0558880	-0.6559090
O	0.2125030	0.2445290	0.7151910
C	-2.1947930	-4.0799590	1.1883610
C	-1.1176100	-4.1974950	0.3092690
C	-0.8608130	-3.1876070	-0.6152600
C	-2.7760280	-1.9454940	0.2204060
C	-3.0258960	-2.9553150	1.1408250
H	-0.0272520	-3.2682820	-1.3031900
H	-0.4792980	-5.0728590	0.3444620
H	-2.3910270	-4.8651050	1.9101430
H	-3.8642390	-2.8709010	1.8229920
H	-3.4132160	-1.0694850	0.1755000
H	0.9759010	-1.2684980	-0.4509650
C	2.5750480	-0.3413930	0.5817760
C	3.6004900	-1.0797240	-0.0214380
C	2.8366820	0.3128210	1.7852540
C	4.8619160	-1.1617960	0.5665980
H	3.4068550	-1.5925390	-0.9599800
C	4.0996580	0.2373890	2.3790860
H	2.0292970	0.8704710	2.2468720
C	5.1164050	-0.5001590	1.7719490
H	5.6460110	-1.7406690	0.0892120
H	4.2892800	0.7519880	3.3158680
H	6.0967870	-0.5633320	2.2321370
Cs	-2.1513230	2.0867780	0.9295720

TS **TS^{RS}-2**

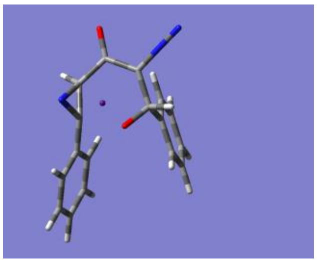

E = -989.888656, H (0K) = -989.638417,
H (298K) = -989.617102,
G (298K) = -989.693876 au.
Imaginary frequency = 1.

C	-0.1512410	1.1778140	-0.7764570
C	-0.1579040	-0.8730730	1.2237440
N	0.9538480	-0.9728400	1.8704430
C	0.0553830	0.2398620	2.1477320
C	0.5423970	1.6037230	1.7686550

TS **TS^{RR}-2**

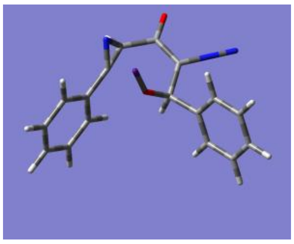

E = -989.890639, H (0K) = -989.640181,
H (298K) = -989.618908,
G (298K) = -989.695667 au.
Imaginary frequency = 1.

C	0.9582630	1.1213750	-0.3417100
C	-1.6076470	1.1376570	0.8508080
N	-1.6668300	0.7380150	2.0751620
C	-0.9980370	2.0879400	1.7790500
C	0.4718300	2.2669650	2.0097810
O	0.8526060	2.8958910	3.0050710

O	0.9367860	2.3723010	2.6542030
C	0.5116120	1.9868410	0.3651240
H	-0.4246780	0.2481250	3.1263350
H	0.2715200	1.5901140	-1.7136410
C	-1.6709000	1.4281870	-0.8497730
N	1.0147910	3.1530310	0.0797730
N	1.4589760	4.1597500	-0.1932580
C	-1.2077080	-1.8125890	0.8207900
O	0.1585350	-0.1512140	-0.6545020
C	-3.2028730	-3.6359190	0.0999880
C	-3.5475800	-2.3501980	0.5205860
C	-2.5540430	-1.4394100	0.8758310
C	-0.8637560	-3.1076430	0.4121820
C	-1.8576080	-4.0120700	0.0464270
C	-2.3745660	2.2591080	0.0235910
C	-3.7640320	2.3890930	-0.0754030
C	-4.4652950	1.6967720	-1.0599060
C	-3.7667970	0.8747420	-1.9500410
C	-2.3857980	0.7426240	-1.8411110
H	-2.8162250	-0.4329480	1.1799940
H	-4.5898020	-2.0533830	0.5644710
H	-3.9763320	-4.3418530	-0.1823610
H	-1.5860880	-5.0115570	-0.2761770
H	0.1807650	-3.3984620	0.3789600
H	-1.8486580	0.0837240	-2.5147490
H	-4.3027970	0.3313940	-2.7214000
H	-5.5428620	1.7947900	-1.1365390
H	-4.2940820	3.0305240	0.6210990
H	-1.8475050	2.8054760	0.7976610
Cs	3.3306390	-0.7236620	-0.6714860

C	1.4007540	1.7346560	1.0234270
H	-1.5558930	2.9608740	2.1191250
N	2.6631610	1.9099150	1.2851980
N	3.7658130	2.0501870	1.5094500
C	-2.5046320	1.1095520	-0.3074550
O	-0.0744810	0.2510410	-0.1359600
C	-4.2283890	1.0505740	-2.5110990
C	-3.2773390	2.0668810	-2.3923960
C	-2.4179360	2.0967550	-1.2960260
C	-3.4706210	0.1014170	-0.4198610
C	-4.3238960	0.0696310	-1.5209370
H	-1.6738010	2.8797710	-1.2030440
H	-3.2059220	2.8351480	-3.1547530
H	-4.8957440	1.0274240	-3.3656830
H	-5.0673010	-0.7158310	-1.6051990
H	-3.5521100	-0.6512610	0.3568170
H	0.6551660	1.9876270	-0.9620140
C	2.1222870	0.4398010	-1.0548060
C	2.5133520	0.8449040	-2.3329050
C	2.7812360	-0.6435590	-0.4583860
C	3.5398130	0.1798030	-3.0094140
H	2.0098220	1.6831890	-2.8045580
C	3.8066430	-1.3074660	-1.1271940
H	2.4894860	-0.9615850	0.5370930
C	4.1884920	-0.8982190	-2.4087070
H	3.8303290	0.5042600	-4.0031180
H	4.3110110	-2.1419980	-0.6513960
H	4.9859140	-1.4156960	-2.9310190
Cs	-0.5748360	-2.6692030	0.7587850

Molecule **(1RR,3SR,6RR)-Ph^{a}-C**

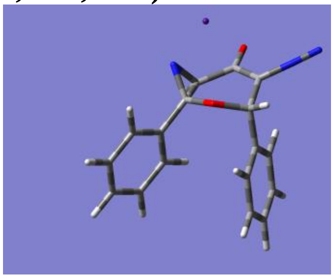

E = -989.896573, H (0K) = -989.644342,
H (298K) = -989.623206,
G (298K) = -989.699926 au.
Imaginary frequency = 0.

C	0.1127370	-1.1770410	-0.7247400
C	0.2174910	0.7668310	0.7815240
N	-0.8418900	1.0070060	1.6418100
C	0.0820690	-0.1198370	1.9619260
C	-0.4870630	-1.4871080	1.7997670
O	-0.8591550	-2.1793520	2.7502700
C	-0.5783310	-1.9275250	0.4044470
H	0.7648150	-0.0361160	2.8129160
H	-0.3678570	-1.4536540	-1.6683420
C	1.6079340	-1.4819330	-0.8506530
N	-1.1787330	-3.0529550	0.1603330
N	-1.7083480	-4.0426000	-0.0017990
C	1.3531650	1.7352180	0.6102660

Molecule **(1RR,3RR,6RR)-Ph^{e}-C**

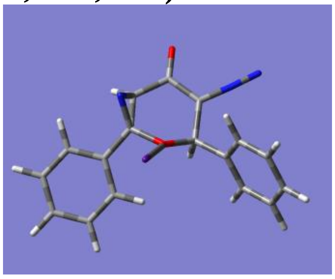

E = -989.900350, H (0K) = -989.647960,
H (298K) = -989.626931,
G (298K) = -989.702417 au.
Imaginary frequency = 0.

C	1.0606470	-1.1168580	0.6239640
C	-1.1752180	-1.1557670	-0.3747930
N	-1.2935820	-1.3483630	-1.7490010
C	-0.9240870	-2.5205070	-0.9276590
C	0.4740240	-3.0262780	-1.0534100
O	0.7577730	-4.0533650	-1.6739280
C	1.4747250	-2.1893070	-0.3863420
H	-1.6543070	-3.3227800	-0.7681620
N	2.7242480	-2.5086400	-0.5412320
N	3.7997360	-2.8259220	-0.7094850
C	-2.3604110	-0.8706120	0.5125370
O	-0.0202990	-0.3670380	0.0892010
C	-4.6257930	-0.3586490	2.1110590

O	-0.1204980	0.2168140	-0.5709670
C	3.4436130	3.5818080	0.2759990
C	3.7088550	2.3013640	0.7614060
C	2.6696830	1.3816510	0.9205800
C	1.0941120	3.0250500	0.1278080
C	2.1302500	3.9406530	-0.0434940
C	2.2844410	-2.3657840	-0.0097970
C	3.6594920	-2.5718650	-0.1553320
C	4.3680010	-1.8992300	-1.1482450
C	3.6936860	-1.0213000	-2.0020710
C	2.3255980	-0.8163830	-1.8526220
H	2.8824340	0.3797880	1.2759950
H	4.7252860	2.0139030	1.0095140
H	4.2503100	4.2955210	0.1474290
H	1.9164370	4.9352830	-0.4210570
H	0.0739310	3.3101450	-0.1090710
H	1.8074440	-0.1198820	-2.5027720
H	4.2369510	-0.4940020	-2.7789110
H	5.4353770	-2.0562390	-1.2595350
H	4.1728220	-3.2559000	0.5119330
H	1.7477790	-2.8950140	0.7690940
Cs	-3.4149110	0.7728890	-0.5993570

C	-3.3629960	0.0699490	2.5197250
C	-2.2396150	-0.1867170	1.7303950
C	-3.6362100	-1.2943040	0.1104010
C	-4.7559580	-1.0461220	0.9015340
H	-1.2642700	0.1524350	2.0550420
H	-3.2484110	0.6071840	3.4556330
H	-5.4979390	-0.1585660	2.7240850
H	-5.7329940	-1.3834870	0.5712560
H	-3.7399440	-1.8137780	-0.8356940
H	0.7276500	-1.6300170	1.5358180
C	2.1668080	-0.1474010	0.9745420
C	2.5249450	0.0623490	2.3075960
C	2.8134290	0.5803760	-0.0323890
C	3.5159950	0.9902020	2.6352560
H	2.0261100	-0.4990440	3.0907840
C	3.7994930	1.5083280	0.2930030
H	2.5421090	0.4176030	-1.0700270
C	4.1529710	1.7156890	1.6292710
H	3.7871700	1.1454730	3.6737980
H	4.2947750	2.0677220	-0.4931910
H	4.9223360	2.4370360	1.8821080
Cs	-0.6016620	2.5097760	-1.1562500

Ts **TS^{RS}-3**

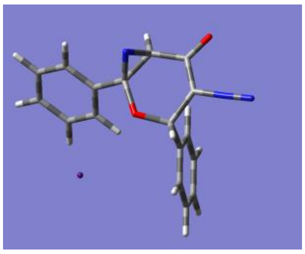

E = -989.896729, H (0K) = -989.644369,
H (298K) = -989.624250,
G (298K) = -989.695931 au.
Imaginary frequency = 1.

C	-1.5036550	-0.4707080	-0.7574950
C	-0.2836680	1.7348140	-0.5177100
N	-0.6740630	2.6000190	-1.5132010
C	-1.5636520	2.4785610	-0.3221330
C	-2.8572040	1.8056310	-0.5672060
O	-3.9464000	2.3801370	-0.4744030
C	-2.7340430	0.4037040	-0.9792060
H	-1.6274600	3.2993880	0.3995440
H	-1.4469710	-1.1845980	-1.5848850
C	-1.5696130	-1.2695620	0.5411280
N	-3.8286300	-0.1927630	-1.3489640
N	-4.8037610	-0.6783040	-1.6649850
C	0.9010460	1.9862390	0.3885880
O	-0.2898230	0.2753180	-0.7952030
C	3.0999540	2.5014330	2.0686410
C	2.2724850	1.4007460	2.3082260
C	1.1854220	1.1451980	1.4745290
C	1.7298380	3.0895540	0.1624560
C	2.8235650	3.3449080	0.9925440
C	-1.9709670	-0.6833340	1.7471460
C	-1.9347030	-1.4125520	2.9342000
C	-1.5008940	-2.7403600	2.9302270

Molecule **(1RR,3SR,6RR)-Ph^{e}-C**

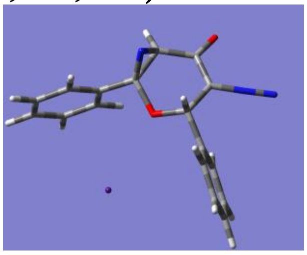

E = -989.908510, H (0K) = -989.656094,
H (298K) = -989.635071,
G (298K) = -989.709864 au.
Imaginary frequency = 0.

C	1.5841020	0.6321950	0.7419480
C	-0.4689660	1.9563990	0.5910920
N	-0.0774050	2.7479940	1.6300830
C	0.2761490	3.2079120	0.2432860
C	1.6752130	3.0953750	-0.2167420
O	2.2640960	3.9957690	-0.8273710
C	2.3182400	1.8069280	0.0894580
H	-0.2478070	4.0607120	-0.1946400
H	1.6330180	0.7580810	1.8273090
C	2.1290040	-0.7295730	0.3689890
N	3.5608790	1.6783780	-0.2612840
N	4.6478780	1.6058850	-0.5785140
C	-1.8898400	1.6991380	0.1677230
O	0.2231720	0.6553230	0.3224690
C	-4.5171540	1.0791710	-0.5935400
C	-3.5366780	1.2889980	-1.5659800
C	-2.2289960	1.5931550	-1.1857630
C	-2.8794160	1.5025670	1.1355740
C	-4.1857200	1.1885560	0.7588130
C	2.3018120	-1.0880650	-0.9742290
C	2.7053810	-2.3775100	-1.3132940
C	2.9400990	-3.3247380	-0.3128410

C	-1.1090250	-3.3351170	1.7309000
C	-1.1449440	-2.6015500	0.5436400
H	0.5546510	0.2843940	1.6587260
H	2.4760540	0.7404840	3.1450030
H	3.9484550	2.6986260	2.7149870
H	3.4599270	4.2016090	0.7962030
H	1.5061270	3.7457920	-0.6710660
H	-0.8428830	-3.0688040	-0.3887110
H	-0.7793880	-4.3684530	1.7174600
H	-1.4755310	-3.3078060	3.8541270
H	-2.2453020	-0.9452730	3.8624750
H	-2.3076380	0.3472760	1.7629020
Cs	2.4504600	-1.4555380	-1.1543770

C	2.7771440	-2.9720810	1.0265140
C	2.3742780	-1.6785000	1.3646940
H	-1.4629460	1.7404790	-1.9397960
H	-3.7905660	1.2134740	-2.6181180
H	-5.5331300	0.8383880	-0.8874920
H	-4.9444420	1.0307660	1.5181740
H	-2.6194320	1.5932610	2.1849700
H	2.2373990	-1.4075330	2.4065210
H	2.9598290	-3.7017420	1.8079030
H	3.2502660	-4.3297220	-0.5776390
H	2.8332240	-2.6462140	-2.3563160
H	2.1074520	-0.3589320	-1.7534780
Cs	-1.4816070	-2.1728200	-0.0520800

Molecule **(1RR,3SR,6RR)-Ph^{e}-4**

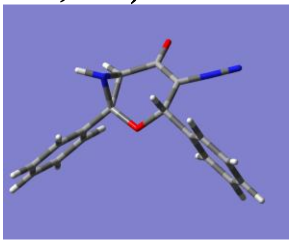

E = -970.552904, H (0K) = -970.286192,
H (298K) = -970.267656,
G (298K) = -970.334625 au.
Imaginary frequency = 0.

C	1.0249150	0.1765050	0.7111610
O	-0.2422250	-0.3503830	0.2745100
C	-1.3595630	0.4822830	0.4898030
N	-1.3007930	1.3387460	1.6513880
C	-1.2202570	1.9570880	0.2885800
C	0.1112250	2.4991300	-0.1161440
O	0.2340170	3.5985120	-0.6519770
C	1.2083960	1.5718880	0.1104530
H	-2.0713290	2.5226060	-0.0713930
H	-2.2257710	1.4923480	2.0463210
H	1.0113810	0.2590430	1.7998190
C	2.1039470	-0.7915990	0.2879760
N	2.3959860	1.9603210	-0.2689130
N	3.4100340	2.3273730	-0.5992970
C	-2.6130200	-0.2418280	0.1136270
C	-3.1963310	-1.1258140	1.0267300
C	-4.3524920	-1.8232150	0.6818390
C	-4.9278580	-1.6428990	-0.5778900
C	-4.3447500	-0.7636800	-1.4909550
C	-3.1876140	-0.0644480	-1.1463340
C	2.2223110	-1.1782870	-1.0518670
C	3.2342380	-2.0521350	-1.4410320
C	4.1409750	-2.5393360	-0.4959810
C	4.0288130	-2.1521850	0.8386270
C	3.0100830	-1.2813650	1.2299610
H	-2.7472460	-1.2620280	2.0048500
H	-4.8045200	-2.5047910	1.3939030
H	-5.8284800	-2.1847190	-0.8452560
H	-4.7899590	-0.6208150	-2.4693600
H	-2.7321980	0.6211230	-1.8523190
H	1.5207740	-0.7947820	-1.7842630

Molecule **(1RR,3RR,6RR)-Ph^{e}-4**

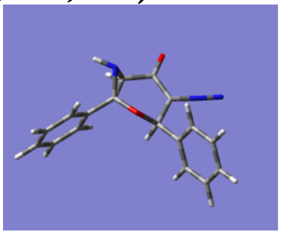

E = -970.549330, H (0K) = -970.282605,
H (298K) = -970.263980,
G (298K) = -970.331523 au.
Imaginary frequency = 0.

C	0.7570210	0.0386370	-0.3441010
O	-0.1925030	-0.1582020	0.7151830
C	-1.3683750	0.6178040	0.5623670
N	-1.4709080	1.6631410	1.5683120
C	-1.2039580	2.0318530	0.1473280
C	0.1729230	2.5688690	-0.1401420
O	0.4062800	3.7724980	-0.1951380
C	1.1499590	1.5189980	-0.3480560
H	-2.0003200	2.5130800	-0.4098330
H	-2.4474970	1.8541610	1.7873830
N	2.3888480	1.8759190	-0.5585510
N	3.4485550	2.2167800	-0.7388100
C	-2.5604930	-0.1964460	0.1679790
C	-3.1840060	-1.0129790	1.1184450
C	-4.2707420	-1.8050220	0.7547510
C	-4.7396300	-1.7888530	-0.5612860
C	-4.1191130	-0.9772560	-1.5107590
C	-3.0304620	-0.1828140	-1.1473860
H	-2.8196950	-1.0232820	2.1402280
H	-4.7533690	-2.4325400	1.4959590
H	-5.5867220	-2.4046650	-0.8429730
H	-4.4812280	-0.9605060	-2.5327970
H	-2.5486240	0.4510370	-1.8834140
C	1.9304390	-0.8835830	-0.1236350
C	2.6048270	-0.8823570	1.1027170
C	2.3684360	-1.7199650	-1.1517570
C	3.7030480	-1.7163370	1.2973430
H	2.2666350	-0.2299870	1.8999490
C	3.4737420	-2.5504990	-0.9587160
H	1.8462080	-1.7212380	-2.1026460
C	4.1407470	-2.5507360	0.2656430

H	3.3188670	-2.3513940	-2.4799270
H	4.9305980	-3.2173770	-0.8007550
H	4.7293820	-2.5284430	1.5758630
H	2.9194740	-0.9814990	2.2686860

H	4.2198970	-1.7141360	2.2506300
H	3.8089860	-3.1970290	-1.7621160
H	4.9977820	-3.1976480	0.4175230
H	0.2760810	-0.1983630	-1.2992980

Molecule **D**

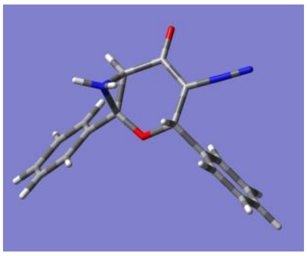

E = -970.995110, H (0K) = -970.714767,
H (298K) = -970.696023,
G (298K) = -970.763555 au.
Imaginary frequency = 0.

C	1.0832040	0.2281820	0.7360830
O	-0.2356600	-0.3221730	0.4224820
C	-1.3310160	0.4890770	0.4184980
N	-1.4584340	1.4934250	1.5970020
C	-1.2020240	1.9487430	0.1876420
C	0.1597060	2.5182820	-0.1478370
O	0.2611630	3.6364630	-0.6344980
C	1.2360660	1.5875020	0.0679960
H	-2.0327600	2.4907420	-0.2438260
H	-2.3934860	1.6686170	1.9641020
H	1.1325220	0.3392450	1.8227320
C	2.1075110	-0.7842980	0.2913270
N	2.4350960	1.9780850	-0.3030270
N	3.4464100	2.3390520	-0.6300760
C	-2.5765110	-0.2580270	0.0793320
C	-3.0500400	-1.2349710	0.9601360
C	-4.1889780	-1.9635150	0.6291170
C	-4.8460720	-1.7228460	-0.5798830
C	-4.3667220	-0.7509210	-1.4576290
C	-3.2305390	-0.0128510	-1.1286770
C	2.2156980	-1.1304090	-1.0603820
C	3.1667030	-2.0626820	-1.4648910
C	4.0182050	-2.6475260	-0.5238800
C	3.9139890	-2.3004750	0.8222620
C	2.9566680	-1.3708730	1.2306390
H	-2.5332420	-1.4167560	1.8959600
H	-4.5641300	-2.7169800	1.3120270
H	-5.7325410	-2.2923660	-0.8357420
H	-4.8755750	-0.5645220	-2.3963380
H	-2.8543500	0.7436720	-1.8075370
H	1.5578590	-0.6711710	-1.7902580
H	3.2469230	-2.3315880	-2.5121040
H	4.7604970	-3.3712210	-0.8418030
H	4.5730840	-2.7526270	1.5547150
H	2.8701410	-1.1002190	2.2775460
H	-0.7155540	1.5925770	2.2902560

TS **TS^{D-E}**

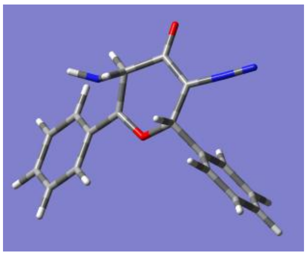

E = -970.993259, H (0K) = -970.714447,
H (298K) = -970.695844,
G (298K) = -970.761732 au.
Imaginary frequency = 1.

C	1.0784030	0.2428660	0.6916470
O	-0.2325130	-0.3350960	0.3416560
C	-1.3184010	0.4240150	0.2800250
N	-1.4725930	1.5569870	1.6606530
C	-1.2299630	1.8979910	0.2406890
C	0.1115400	2.5173620	-0.1228660
O	0.1769720	3.6529850	-0.5705840
C	1.2113520	1.6040830	0.0411740
H	-2.0457230	2.4460700	-0.2130950
H	-2.4075140	1.7434390	2.0104870
H	1.0682180	0.3394050	1.7799260
C	2.1321530	-0.7479290	0.2764000
N	2.3947130	2.0103660	-0.3629460
N	3.3868000	2.3954700	-0.7187720
C	-2.5603640	-0.3285510	0.0185130
C	-2.6844200	-1.6299770	0.5279810
C	-3.8396370	-2.3611880	0.2806180
C	-4.8707410	-1.8051140	-0.4807480
C	-4.7451240	-0.5145780	-0.9934560
C	-3.5942240	0.2288500	-0.7433920
C	2.2816140	-1.0958400	-1.0709390
C	3.2647700	-2.0062790	-1.4471290
C	4.1078960	-2.5639840	-0.4825810
C	3.9624990	-2.2139600	0.8590730
C	2.9716440	-1.3086670	1.2401190
H	-1.8829200	-2.0574550	1.1172840
H	-3.9377050	-3.3636520	0.6805460
H	-5.7701960	-2.3786910	-0.6743130
H	-5.5407120	-0.0848240	-1.5906520
H	-3.5062370	1.2263250	-1.1548710
H	1.6305860	-0.6565090	-1.8191500
H	3.3766060	-2.2786120	-2.4904770
H	4.8760700	-3.2697050	-0.7785640
H	4.6153540	-2.6456580	1.6091530
H	2.8514590	-1.0364610	2.2831920
H	-0.7193790	1.6988380	2.3286590

Molecule **E**

TS **TS^{E-F}**

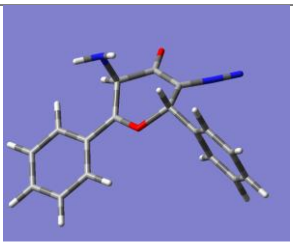

E = -971.006780, H (0K) = -970.726084,
H (298K) = -970.706863,
G (298K) = -970.773777 au.
Imaginary frequency = 0.

C	1.0999310	0.3454240	0.6947360
O	-0.2170490	-0.3353090	0.4353170
C	-1.3068960	0.3227000	0.2277510
N	-1.4969210	2.2720970	1.6880520
C	-1.3098010	1.8351150	0.2983980
C	-0.0284950	2.4495490	-0.2882620
O	-0.0547160	3.5258370	-0.8626480
C	1.1324000	1.6165700	-0.1062790
H	-2.1255150	2.2282740	-0.3016900
H	-2.3924990	1.9481260	2.0451940
H	1.0704990	0.5494010	1.7667050
C	2.1756480	-0.6495950	0.3735010
N	2.2600700	2.0131270	-0.6542970
N	3.1917130	2.4084430	-1.1383150
C	-2.4885090	-0.4630130	0.0020180
C	-2.3836200	-1.8720120	-0.1004060
C	-3.5142130	-2.6344950	-0.3285810
C	-4.7636180	-2.0145760	-0.4478450
C	-4.8823940	-0.6272920	-0.3390190
C	-3.7565690	0.1506680	-0.1196830
C	2.3350910	-1.1203230	-0.9353820
C	3.3355440	-2.0442570	-1.2212370
C	4.1840480	-2.4928050	-0.2059590
C	4.0272460	-2.0214370	1.0968400
C	3.0188640	-1.1036400	1.3893850
H	-1.4167740	-2.3496480	-0.0124460
H	-3.4311670	-3.7108350	-0.4170110
H	-5.6470670	-2.6167630	-0.6273300
H	-5.8531200	-0.1552860	-0.4272400
H	-3.8694570	1.2222720	-0.0275010
H	1.6789550	-0.7667730	-1.7238720
H	3.4561100	-2.4122700	-2.2336310
H	4.9658400	-3.2092890	-0.4319820
H	4.6843300	-2.3692620	1.8856610
H	2.8888780	-0.7384980	2.4022080
H	-0.7668660	1.9411630	2.3136300

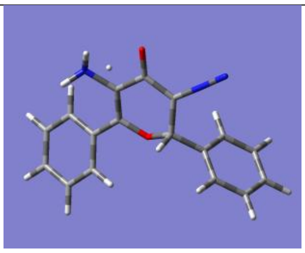

E = -970.936867, H (0K) = -970.662096,
H (298K) = -970.643001,
G (298K) = -970.709952 au.
Imaginary frequency = 1.

C	1.0696740	0.1390130	0.6272020
O	-0.1839060	-0.3970490	0.0442630
C	-1.2904220	0.3246790	0.0376750
N	-2.4103220	2.6051800	0.1744360
C	-1.2141700	1.7304390	0.0466090
C	0.0852120	2.4579110	0.1219890
O	0.1217070	3.6842290	0.0796240
C	1.2218330	1.5754920	0.1905760
H	-1.8328800	2.0924920	-0.9619970
H	-3.1905500	2.2085860	0.6917250
H	0.9087810	0.0888380	1.7069970
C	2.1912060	-0.7712670	0.2168150
N	2.4142250	2.0952310	0.0374550
N	3.4256000	2.5672350	-0.1023410
C	-2.5062780	-0.4838930	-0.0341300
C	-2.4935740	-1.7409250	0.6024150
C	-3.6261410	-2.5440340	0.5820700
C	-4.7728300	-2.1192600	-0.0906910
C	-4.7859350	-0.8847800	-0.7430490
C	-3.6665150	-0.0632380	-0.7114070
C	2.5574450	-0.8815850	-1.1296070
C	3.6043070	-1.7217660	-1.4973920
C	4.2952520	-2.4463970	-0.5228850
C	3.9339270	-2.3338890	0.8190050
C	2.8789650	-1.4996030	1.1892850
H	-1.6050550	-2.0692640	1.1258610
H	-3.6140460	-3.5015790	1.0890340
H	-5.6539600	-2.7504720	-0.1108820
H	-5.6689790	-0.5632370	-1.2823000
H	-3.6938600	0.8752340	-1.2496210
H	2.0258730	-0.3126920	-1.8848370
H	3.8846520	-1.8088960	-2.5409700
H	5.1142260	-3.0960510	-0.8110690
H	4.4691580	-2.8945810	1.5768600
H	2.5924260	-1.4100060	2.2316280
H	-2.1364490	3.5492700	0.4347550

Molecule **F**

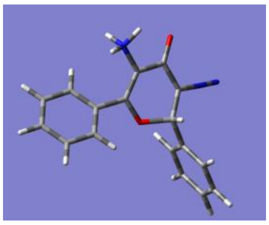

E = -971.038113, H (0K) = -970.755845,
H (298K) = -970.736808,

Molecule **G**

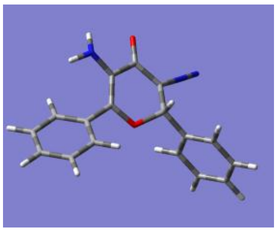

E = -970.587206, H (0K) = -970.320524,
H (298K) = -970.301313,

G (298K) = -970.803306 au.
Imaginary frequency = 0.

C	-1.0798780	0.1575000	-0.6181510
O	0.1514750	-0.4268430	-0.0695220
C	1.2746150	0.3071490	-0.1291540
N	2.4164460	2.4845230	-0.4475980
C	1.2163700	1.6600170	-0.2925360
C	-0.0176050	2.4303810	-0.1494260
O	-0.0115940	3.6590110	-0.0303750
C	-1.1894540	1.5794130	-0.1206980
H	2.1680430	3.3520690	-0.9399470
H	2.8274830	2.7702280	0.4492010
H	-0.9346070	0.1674200	-1.7032320
C	-2.2287370	-0.7379170	-0.2474290
N	-2.3366930	2.0982160	0.2186700
N	-3.3166010	2.5718200	0.5128200
C	2.4908490	-0.5122710	0.0111570
C	2.5670070	-1.7264550	-0.6902560
C	3.7024660	-2.5216540	-0.5822410
C	4.7601680	-2.1248640	0.2386140
C	4.6794220	-0.9304760	0.9548530
C	3.5511870	-0.1225800	0.8424600
C	-2.5469060	-0.9612740	1.0972350
C	-3.6201750	-1.7823690	1.4315520
C	-4.3857390	-2.3776460	0.4258540
C	-4.0722800	-2.1535600	-0.9140810
C	-2.9921800	-1.3369120	-1.2510430
H	1.7430360	-2.0327190	-1.3230170
H	3.7618680	-3.4518050	-1.1354360
H	5.6420610	-2.7494800	0.3253930
H	5.4898460	-0.6309880	1.6091290
H	3.4855420	0.7830010	1.4335780
H	-1.9554330	-0.4940780	1.8771880
H	-3.8619070	-1.9559680	2.4740910
H	-5.2236750	-3.0140520	0.6880730
H	-4.6636310	-2.6151240	-1.6968030
H	-2.7429760	-1.1641460	-2.2924470
H	3.1484590	2.0174950	-0.9940870

G (298K) = -970.368948 au.
Imaginary frequency = 0.

C	1.0287000	0.1389060	0.5815530
O	-0.1712130	-0.4004300	-0.0122360
C	-1.3059510	0.3675390	0.1066870
N	-2.3560770	2.5382720	0.5943550
C	-1.2590570	1.7208340	0.2915600
C	0.0178010	2.4400420	0.1234940
O	0.0639980	3.6659120	-0.0290780
C	1.1782850	1.5675720	0.1044780
H	-2.2365950	3.4821650	0.2438990
H	0.8671300	0.1509180	1.6666440
C	2.1891130	-0.7571700	0.2377560
N	2.3369970	2.0640960	-0.2161300
N	3.3344930	2.5199340	-0.4917020
C	-2.5252240	-0.4552100	-0.0021450
C	-2.5875380	-1.6855650	0.6724120
C	-3.7230380	-2.4856690	0.5786980
C	-4.8071150	-2.0778380	-0.2005910
C	-4.7458260	-0.8666670	-0.8916740
C	-3.6140980	-0.0606520	-0.7957360
C	2.4820840	-1.0538960	-1.0981490
C	3.5716970	-1.8626560	-1.4111040
C	4.3819310	-2.3713230	-0.3928650
C	4.0952670	-2.0727860	0.9385510
C	2.9980940	-1.2694750	1.2534520
H	-1.7448450	-2.0053480	1.2736180
H	-3.7616340	-3.4282580	1.1134540
H	-5.6900820	-2.7028250	-0.2748740
H	-5.5755730	-0.5533590	-1.5154790
H	-3.5663600	0.8619030	-1.3631430
H	1.8559100	-0.6530540	-1.8874450
H	3.7919120	-2.0934300	-2.4474730
H	5.2324020	-2.9978560	-0.6382930
H	4.7205230	-2.4663110	1.7322430
H	2.7710530	-1.0396650	2.2890920
H	-3.2647560	2.1692170	0.3543430

TS **TS^{F-H}**

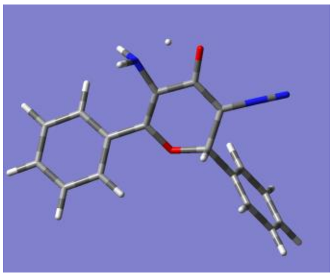

E = -971.015724, H (0K) = -970.739032,
H (298K) = -970.720475,
G (298K) = -970.786488 au.
Imaginary frequency = 1.

C	-1.0680550	0.1419400	-0.6241480
O	0.1693390	-0.4208770	-0.0768200
C	1.3025440	0.3079350	-0.0998970
N	2.2535680	2.6801690	-0.2187220
C	1.2298920	1.6707510	-0.1990100

Molecule **H**

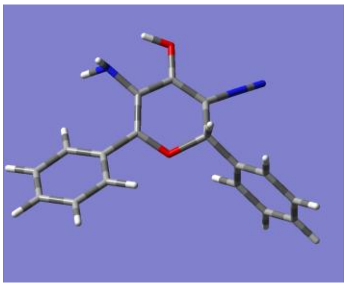

E = -971.021776, H (0K) = -970.742271,
H (298K) = -970.722968,
G (298K) = -970.790481 au.
Imaginary frequency = 0.

C	1.0717510	0.1409930	0.6390910
O	-0.1753170	-0.4221450	0.1105380
C	-1.2925080	0.3156370	0.1398170
N	-2.3397530	2.6057270	0.3572210
C	-1.2480110	1.6950370	0.2591910

C	-0.0318390	2.3593830	-0.1227560
O	0.0689050	3.6334980	-0.0249800
C	-1.2010820	1.5805050	-0.1480020
H	1.2602410	3.6253140	-0.1131870
H	2.8648700	2.6900390	0.5937130
H	-0.9371440	0.1460560	-1.7104670
C	-2.2060680	-0.7568310	-0.2293220
N	-2.3771670	2.1220610	0.0764950
N	-3.3899380	2.5657110	0.2686990
C	2.5090590	-0.5181610	0.0086570
C	2.4950230	-1.8011670	-0.5681670
C	3.6251920	-2.6080210	-0.5072520
C	4.7741450	-2.1557400	0.1435610
C	4.7885890	-0.8920210	0.7359510
C	3.6668580	-0.0729580	0.6675450
C	-2.5243730	-0.9439820	1.1206520
C	-3.5861730	-1.7709490	1.4759470
C	-4.3390850	-2.4071630	0.4858240
C	-4.0255500	-2.2179550	-0.8594170
C	-2.9572250	-1.3950950	-1.2178070
H	1.6049470	-2.1513950	-1.0749520
H	3.6096760	-3.5895960	-0.9665120
H	5.6536630	-2.7877050	0.1936790
H	5.6725730	-0.5455380	1.2585220
H	3.6910110	0.8876510	1.1623250
H	-1.9423160	-0.4451710	1.8880630
H	-3.8289000	-1.9173480	2.5223760
H	-5.1682200	-3.0480510	0.7644870
H	-4.6079470	-2.7108770	-1.6295450
H	-2.7074700	-1.2478430	-2.2629980
H	2.8127020	2.6950790	-1.0705540

C	0.0109340	2.3420260	0.1398490
O	0.0445450	3.6460490	0.0047100
C	1.1796400	1.5679310	0.1402290
H	-0.9080370	3.9273160	0.0626770
H	-2.9958860	2.5569340	-0.4130830
H	0.9515470	0.1587930	1.7264710
C	2.2061060	-0.7597840	0.2417800
N	2.3437850	2.1054150	-0.1577320
N	3.3478600	2.5410110	-0.4024760
C	-2.5011530	-0.5022960	0.0032120
C	-2.5533110	-1.7347450	0.6782950
C	-3.6869620	-2.5335170	0.5834550
C	-4.7651470	-2.1265480	-0.2042990
C	-4.7070210	-0.9175520	-0.9001000
C	-3.5851410	-0.1029150	-0.7944620
C	2.4981470	-0.9787330	-1.1093940
C	3.5584210	-1.8075760	-1.4647970
C	4.3354150	-2.4133330	-0.4742580
C	4.0478860	-2.1920460	0.8719220
C	2.9812890	-1.3675710	1.2310790
H	-1.7147420	-2.0484880	1.2873860
H	-3.7287390	-3.4735630	1.1211690
H	-5.6462980	-2.7534630	-0.2817010
H	-5.5337750	-0.6117650	-1.5305570
H	-3.5386890	0.8134700	-1.3674360
H	1.8974420	-0.5034990	-1.8774030
H	3.7809200	-1.9793240	-2.5118150
H	5.1630300	-3.0559710	-0.7533280
H	4.6490190	-2.6615850	1.6422260
H	2.7513030	-1.1957400	2.2769850
H	-2.8461760	2.5205330	1.2336880

TS **TS^{H-I}**

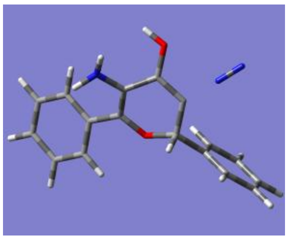

E = -970.977499, H (0K) = -970.702339,
H (298K) = -970.682449,
G (298K) = -970.750989 au.
Imaginary frequency = 1.

C	-1.0666370	0.1759900	-0.8489540
O	0.1901550	-0.4710700	-0.3877890
C	1.2821040	0.2535330	-0.3696300
N	2.3455230	2.3928560	-0.8800080
C	1.2322620	1.6710580	-0.5660740
C	0.0165350	2.2853550	-0.1875000
O	-0.0483060	3.5479480	0.2870910
C	-1.1434370	1.5939660	-0.4634640
H	0.8072220	3.8370060	0.6456750
H	2.2330200	3.3755780	-1.0943890
H	-0.9730320	0.1781820	-1.9411270
C	-2.2167600	-0.7052840	-0.4288490
N	-2.2505510	1.7917030	1.0039130

Molecule **I**

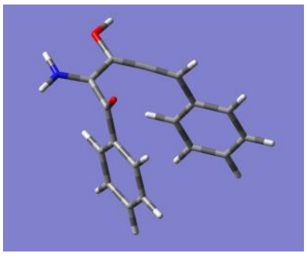

E = -861.506641, H (0K) = -861.239191,
H (298K) = -861.220669,
G (298K) = -861.286481 au.
Imaginary frequency = 0.

C	-0.3486350	-1.9372600	0.8939350
O	2.4803520	0.5750650	1.9803980
C	2.0611790	0.5795210	0.8391280
N	3.8308410	-0.2429870	-0.6353980
C	2.6761970	-0.4697900	-0.0950840
C	1.9828380	-1.7155720	-0.2910010
O	2.6486520	-2.6047560	-1.0939060
C	0.8018800	-1.8803730	0.2802310
H	2.1370890	-3.4239740	-1.1835510
H	4.2919060	-0.9495950	-1.2051870
H	-0.3470110	-2.3490710	1.9045440
C	-1.6419440	-1.4709430	0.3751020
C	1.0240470	1.4716570	0.3061250

N	-3.0691370	2.3923430	1.4385710
C	2.4943030	-0.5062660	-0.0894400
C	2.5294660	-1.8719800	-0.4393880
C	3.6518210	-2.6357300	-0.1511240
C	4.7414920	-2.0585330	0.5046040
C	4.7085210	-0.7107060	0.8697130
C	3.5991950	0.0681400	0.5705500
C	-2.2491410	-1.2814080	0.8444510
C	-3.3460610	-2.0505030	1.2295800
C	-4.4141740	-2.2379510	0.3517040
C	-4.3803430	-1.6614360	-0.9186010
C	-3.2806380	-0.9003970	-1.3117010
H	1.6870190	-2.3173840	-0.9525190
H	3.6793840	-3.6802850	-0.4379330
H	5.6154430	-2.6584000	0.7321280
H	5.5481050	-0.2677640	1.3921700
H	3.5757200	1.1058600	0.8739880
H	-1.4231840	-1.1329510	1.5296590
H	-3.3659260	-2.4999640	2.2159660
H	-5.2676300	-2.8343480	0.6544330
H	-5.2052940	-1.8085430	-1.6064560
H	-3.2499680	-0.4559000	-2.3005780
H	3.0724330	1.9384750	-1.4180340

C	0.2856740	2.2576020	1.2073440
C	-0.7115740	3.0976020	0.7332730
C	-0.9711300	3.1695300	-0.6394160
C	-0.2351090	2.3980920	-1.5387650
C	0.7563280	1.5430610	-1.0700610
C	-1.7764120	-0.9419180	-0.9191650
C	-3.0089520	-0.4744280	-1.3569480
C	-4.1227830	-0.5298550	-0.5134190
C	-3.9981070	-1.0582420	0.7713290
C	-2.7639340	-1.5276300	1.2152390
H	0.4950560	2.1895150	2.2679940
H	-1.2909420	3.6952050	1.4272700
H	-1.7524940	3.8257730	-1.0059900
H	-0.4410200	2.4541060	-2.6010060
H	1.3154750	0.9397550	-1.7757170
H	-0.9145150	-0.8935520	-1.5745670
H	-3.1040430	-0.0627940	-2.3553900
H	-5.0827980	-0.1629050	-0.8589530
H	-4.8593280	-1.1031030	1.4283320
H	-2.6620070	-1.9336880	2.2159090
H	4.3093390	0.6464480	-0.5149200

Molecule **J**

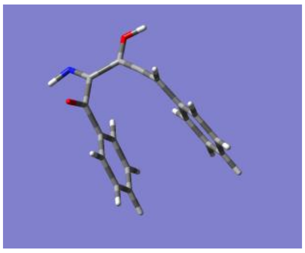

E = -861.051749, H (0K) = -860.797530,
H (298K) = -860.779200,
G (298K) = -860.845253 au.
Imaginary frequency = 0.

C	-0.2682480	-2.0302320	0.7889970
O	2.4072130	0.5569610	2.0694500
C	2.0651370	0.5401550	0.8981830
N	3.9369990	-0.1696550	-0.5372150
C	2.7793690	-0.4152470	-0.0603430
C	2.0450500	-1.6510000	-0.3907520
O	2.6682340	-2.4646890	-1.3047890
C	0.8824460	-1.8916920	0.1777200
H	2.1206250	-3.2511780	-1.4479350
H	-0.2839180	-2.5501670	1.7476500
C	-1.5646410	-1.5108320	0.3192380
C	1.0082490	1.4229270	0.3452580
C	0.1496710	2.0923440	1.2308150
C	-0.8587370	2.9113140	0.7383730
C	-1.0090710	3.0818800	-0.6410540
C	-0.1539930	2.4257740	-1.5263940
C	0.8456520	1.5893110	-1.0376390
C	-1.7060650	-0.8762170	-0.9256490
C	-2.9355150	-0.3586470	-1.3155090
C	-4.0458570	-0.4682100	-0.4727070
C	-3.9176440	-1.1036320	0.7623470

TS **TS**[J-K]

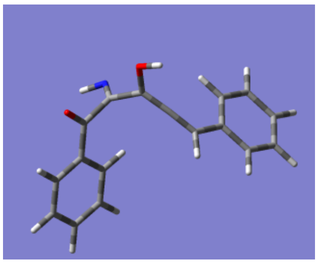

E = -861.040330, H (0K) = -860.786459,
H (298K) = -860.768733,
G (298K) = -860.833723 au.
Imaginary frequency = 1.

C	-1.5672400	0.1169330	-1.1700290
O	3.1989530	2.0396020	-0.3903160
C	2.4563380	1.1871280	0.0694720
N	0.8718380	1.7151890	1.8908660
C	1.1173450	1.6331990	0.6480890
C	0.0686880	1.9950580	-0.3625430
O	0.1434280	3.3092060	-0.7618100
C	-0.7693140	1.0881700	-0.7874180
H	-0.4967720	3.4631130	-1.4741220
H	-1.2745820	-0.4893770	-2.0280940
C	-2.8497590	-0.2486440	-0.5429560
C	2.7876040	-0.2587910	0.1025730
C	4.0487300	-0.6740400	-0.3556500
C	4.3830070	-2.0218880	-0.3518400
C	3.4605450	-2.9693740	0.1037900
C	2.2044920	-2.5651050	0.5561290
C	1.8665190	-1.2141360	0.5584720
C	-3.3387840	0.4097470	0.5983100
C	-4.5497730	0.0307560	1.1663370
C	-5.2955690	-1.0124570	0.6077520

C	-2.6853260	-1.6224610	1.1559720
H	0.2754320	1.9506870	2.2975520
H	-1.5301870	3.4152850	1.4242150
H	-1.7967750	3.7213810	-1.0237060
H	-0.2734540	2.5557530	-2.5958020
H	1.4979290	1.0733410	-1.7324680
H	-0.8471420	-0.7847750	-1.5802770
H	-3.0304020	0.1357240	-2.2759890
H	-5.0033210	-0.0618920	-0.7794930
H	-4.7748110	-1.1930590	1.4205920
H	-2.5837110	-2.1109100	2.1196070
H	4.2843060	0.7360080	-0.2118670

C	-4.8191130	-1.6734180	-0.5241220
C	-3.6048290	-1.2947790	-1.0951650
H	4.7531250	0.0691860	-0.7095590
H	5.3582730	-2.3391760	-0.7031070
H	3.7225300	-4.0216480	0.1043320
H	1.4890370	-3.3000310	0.9065240
H	0.8868740	-0.9108600	0.9071260
H	-2.7628830	1.2175930	1.0370820
H	-4.9154420	0.5467740	2.0474540
H	-6.2394680	-1.3057460	1.0538280
H	-5.3910790	-2.4837000	-0.9627660
H	-3.2350350	-1.8108530	-1.9752850
H	1.6701860	1.3977870	2.4503290

Molecule **K**

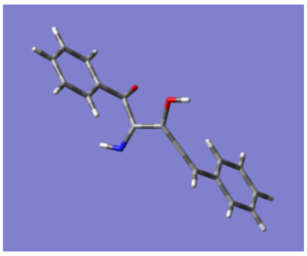

E = -861.046387, H (0K) = -860.792411,
H (298K) = -860.773932,
G (298K) = -860.841986 au.
Imaginary frequency = 0.

C	2.5755860	-1.4084560	0.2307500
O	-1.5317850	1.9490730	-0.0145470
C	-1.8836640	0.7909590	-0.1659630
N	-0.6377200	-0.7196080	-1.6432480
C	-0.7951860	-0.2387640	-0.4735150
C	0.1313560	-0.5578320	0.6413800
O	-0.4486980	-0.3181080	1.8729380
C	1.3495260	-1.0031400	0.4538160
H	0.2016300	-0.4790470	2.5722950
H	2.7620340	-2.4808180	0.1579550
C	3.7575880	-0.5417440	0.0645050
C	-3.2989110	0.3524230	-0.1384280
C	-4.3077730	1.3227320	-0.0230730
C	-5.6416470	0.9393980	0.0238420
C	-5.9817060	-0.4159650	-0.0372360
C	-4.9850470	-1.3857840	-0.1465300
C	-3.6463330	-1.0055140	-0.1998070
C	3.6712570	0.8591760	0.1296780
C	4.8076450	1.6431390	-0.0341520
C	6.0502860	1.0447170	-0.2666230
C	6.1463550	-0.3448330	-0.3339420
C	5.0079610	-1.1330120	-0.1697080
H	-4.0311900	2.3690800	0.0266140
H	-6.4187000	1.6905380	0.1087790
H	-7.0237460	-0.7135910	0.0012820
H	-5.2495020	-2.4359660	-0.1907750
H	-2.8781630	-1.7646350	-0.2846010
H	2.7097200	1.3284780	0.3095350
H	4.7277900	2.7234690	0.0185550
H	6.9346070	1.6592980	-0.3936840
H	7.1061230	-0.8165270	-0.5141460

TS $\mathbf{TS^{K-L}}$

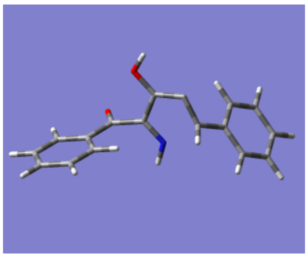

E = -861.010581, H (0K) = -860.757164,
H (298K) = -860.739721,
G (298K) = -860.804310 au.
Imaginary frequency = 1.

C	1.7466030	-0.3572950	1.1220520
O	-1.4985450	1.5120980	-1.7253960
C	-1.6074460	0.7000140	-0.8212600
N	0.4292890	-0.5749040	-0.2965330
C	-0.3687140	0.3841140	-0.0117370
C	0.0384830	1.1918760	1.1487540
O	-0.8118150	2.2168430	1.4899130
C	1.1779960	0.7806670	1.7187260
H	-0.4676860	2.6407530	2.2879820
H	1.5115500	-1.3093290	1.6037600
C	3.0569740	-0.3572120	0.4198580
C	-2.8684740	0.0144360	-0.4650360
C	-4.0282000	0.2936010	-1.2058910
C	-5.2248200	-0.3343970	-0.8861750
C	-5.2753340	-1.2429400	0.1759730
C	-4.1271420	-1.5229200	0.9175830
C	-2.9246940	-0.8973380	0.5998070
C	3.6145570	0.8284130	-0.0787920
C	4.8218180	0.8001470	-0.7705660
C	5.4901290	-0.4112430	-0.9683050
C	4.9438780	-1.5949240	-0.4710820
C	3.7322880	-1.5675440	0.2175670
H	-3.9757250	1.0006860	-2.0250520
H	-6.1194850	-0.1192090	-1.4591900
H	-6.2110870	-1.7314430	0.4243200
H	-4.1686830	-2.2259010	1.7414810
H	-2.0366460	-1.1188500	1.1807650
H	3.1004240	1.7693830	0.0818060
H	5.2455580	1.7221490	-1.1531900
H	6.4331780	-0.4301820	-1.5034320

H	5.0843080	-2.2141360	-0.2230990
H	-1.3607690	-0.3816110	-2.2830630

H	5.4600230	-2.5371410	-0.6186260
H	3.3043900	-2.4879530	0.6013720
H	0.2146280	-1.2248890	-1.0512580

Molecule **L**

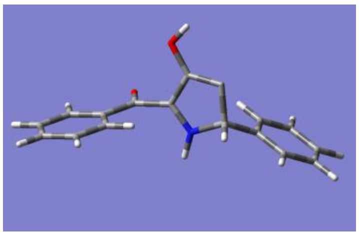

E = -861.025288, H (0K) = -860.769697,
H (298K) = -860.752187,
G (298K) = -860.817127 au.
Imaginary frequency = 0.

C	1.5638560	-0.7696140	0.6356760
O	-1.4539050	2.0970320	-0.8359160
C	-1.5848300	0.9783360	-0.3665010
N	0.4738080	-0.4009200	-0.3145480
C	-0.3762390	0.3762780	0.3065300
C	0.0675980	0.5962840	1.6516170
O	-0.6989250	1.3887730	2.4707240
C	1.2419130	-0.0517220	1.9162290
H	-0.2602310	1.4265490	3.3323750
H	1.4595930	-1.8487750	0.8053390
C	2.9311980	-0.4861340	0.0373110
C	-2.8373370	0.1995560	-0.3904270
C	-3.9656900	0.7456630	-1.0245740
C	-5.1548810	0.0304930	-1.0602690
C	-5.2296290	-1.2316490	-0.4617490
C	-4.1133860	-1.7784160	0.1721260
C	-2.9173730	-1.0673480	0.2085830
C	3.2276550	0.7844250	-0.4696690
C	4.4880390	1.0544350	-0.9965170
C	5.4684990	0.0585820	-1.0170470
C	5.1776660	-1.2074950	-0.5120380
C	3.9107970	-1.4799750	0.0098210
H	-3.8941720	1.7247420	-1.4827420
H	-6.0251380	0.4506700	-1.5511490
H	-6.1601960	-1.7875430	-0.4899300
H	-4.1749470	-2.7554970	0.6367910
H	-2.0553070	-1.4979930	0.7052100
H	2.4703740	1.5612880	-0.4491430
H	4.7074520	2.0414950	-1.3888610
H	6.4503910	0.2701530	-1.4259720
H	5.9317960	-1.9868540	-0.5271760
H	3.6850060	-2.4678000	0.3975810
H	0.3842280	-0.7206170	-1.2735600

TS $\mathbf{TS}^{L\text{-}5}$

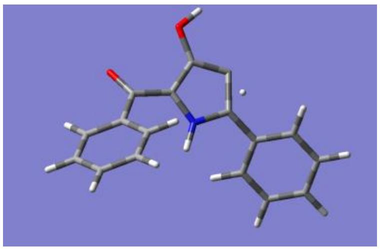

E = -861.007135, H (0K) = -860.754282,
H (298K) = -860.737313,
G (298K) = -860.799655 au.
Imaginary frequency = 1.

C	1.5243940	0.6740660	0.2706530
O	-2.6416740	1.9967670	-1.0532050
C	-2.0474650	1.0924950	-0.4812900
N	0.2719120	0.2049930	-0.1253550
C	-0.6060520	1.2508850	-0.1642530
C	0.0952530	2.4014240	0.1938000
O	-0.4783740	3.6296610	0.2939170
C	1.4761780	2.1331210	0.4227700
H	0.1999770	4.2354930	0.6292530
H	1.4698840	1.0445460	1.4238980
C	2.7350620	-0.1648120	0.0529380
C	-2.7400650	-0.1735820	-0.0984430
C	-3.8287030	-0.6003940	-0.8735330
C	-4.5229250	-1.7549530	-0.5295840
C	-4.1523100	-2.4815430	0.6052390
C	-3.0827970	-2.0527060	1.3915740
C	-2.3708530	-0.9085600	1.0379530
C	2.7772480	-1.0844940	-1.0018010
C	3.9222060	-1.8515670	-1.2202770
C	5.0345290	-1.7066020	-0.3927760
C	4.9968010	-0.7886070	0.6592450
C	3.8548700	-0.0247580	0.8831450
H	-4.1142560	-0.0252190	-1.7462880
H	-5.3532360	-2.0890630	-1.1414500
H	-4.6984990	-3.3782920	0.8763100
H	-2.8031770	-2.6072610	2.2800970
H	-1.5492300	-0.5763830	1.6613520
H	1.9270190	-1.1941210	-1.6654510
H	3.9434090	-2.5569880	-2.0436320
H	5.9241830	-2.3019880	-0.5652140
H	5.8559530	-0.6719970	1.3105060
H	3.8270200	0.6809460	1.7059990
H	0.0357220	-0.7625300	-0.3057500

Molecule **5**

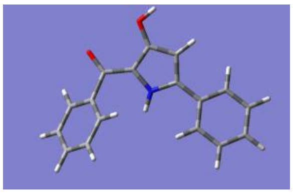

E = -861.126354, H (0K) = -860.868723,
H (298K) = -860.851628,

Molecule $\mathbf{MeSO_3H}$

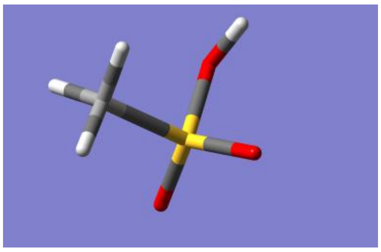

G (298K) = -860.913799 au.
Imaginary frequency = 0.

C	1.5107370	0.7280310	0.0304900
O	-2.8426840	2.2006980	-0.2465370
C	-2.1017840	1.2233440	-0.0938150
N	0.2288630	0.2939300	-0.0784080
C	-0.6672150	1.3586580	0.0112530
C	0.1269740	2.5027120	0.2058460
O	-0.3685340	3.7500980	0.3787930
C	1.4766590	2.1132530	0.2253940
H	0.3633130	4.3544090	0.5708270
H	2.3266730	2.7579000	0.3902970
C	2.6624650	-0.1725260	-0.0461270
C	-2.7029270	-0.1552050	-0.0287000
C	-3.7487010	-0.4695040	-0.9070520
C	-4.3603070	-1.7188550	-0.8549260
C	-3.9503800	-2.6597830	0.0930530
C	-2.9247310	-2.3465430	0.9849210
C	-2.2961320	-1.1030650	0.9201690
C	2.5215620	-1.5565230	0.1557990
C	3.6266120	-2.4001480	0.0789220
C	4.8931680	-1.8801000	-0.1919830
C	5.0452010	-0.5059770	-0.3874990
C	3.9424090	0.3403420	-0.3187180
H	-4.0716330	0.2699830	-1.6307920
H	-5.1582400	-1.9589940	-1.5489490
H	-4.4323490	-3.6301850	0.1385510
H	-2.6143670	-3.0675470	1.7330230
H	-1.5078980	-0.8629850	1.6243850
H	1.5507780	-1.9784210	0.3917000
H	3.4987070	-3.4648920	0.2400350
H	5.7529000	-2.5383430	-0.2483050
H	6.0246320	-0.0923960	-0.6013100
H	4.0716950	1.4028220	-0.4891720
H	-0.0381630	-0.6438960	-0.3416430

E = -664.447980, H (0K) = -664.387135,
H (298K) = -664.380354,
G (298K) = -664.416068 au.
Imaginary frequency = 0.

S	0.0883790	-0.1410230	0.0441500
O	0.4277220	-1.1894970	-0.9077020
O	0.4855940	-0.2706130	1.4445160
O	0.7887560	1.2085290	-0.5687100
H	0.8402070	1.9020660	0.1149150
C	-1.6548970	0.2418200	-0.0576790
H	-2.1916190	-0.6333280	0.3110650
H	-1.8527130	1.1104480	0.5701390
H	-1.8971320	0.4389180	-1.1012880

Molecule **$MeSO_3^-$**

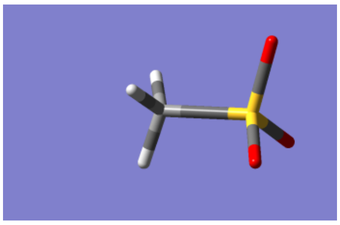

E = -664.003086, H (0K) = -663.953453,
H (298K) = -663.947384,
G (298K) = -663.981799 au.
Imaginary frequency = 0.

S	-0.1563910	0.0001110	-0.0001270
O	-0.5600170	-1.1022400	-0.9252640
O	-0.5566720	1.3531610	-0.4929920
O	-0.5613100	-0.2489510	1.4169430
C	1.6539450	-0.0018500	0.0013390
H	2.0014520	0.1824560	-1.0156440
H	2.0013550	0.7864610	0.6698720
H	1.9997700	-0.9753550	0.3502790

Molecule **N_2**

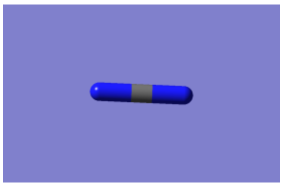

E = -109.554666, H (0K) = -109.549088,
H (298K) = -109.545783,
G (298K) = -109.567519 au.
Imaginary frequency = 0.

N	0.0000000	0.0000000	0.5475300
N	0.0000000	0.0000000	-0.5475300

Molecule **(1RR,3SR,6RR)-Ph^e-4^{inv-}**

TS N-inversion (1RR,3SR,6RR)-Ph^e 4

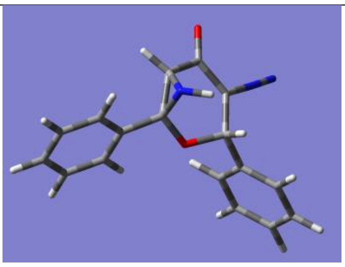

E = -970.550885, H (0K) = -970.284110,
H (298K) = -970.265633,
G (298K) = -970.332465 au.
Imaginary frequency = 0.

C	-1.0379720	0.3132830	-0.7318390
O	0.2257480	-0.3048000	-0.4365300
C	1.3767990	0.4943400	-0.5056910
N	1.4989980	1.5373480	-1.4947330
C	1.2854910	1.9432660	-0.0778440
C	-0.0302460	2.4484280	0.4125460
O	-0.1118030	3.4646830	1.1006310
C	-1.1613260	1.5997030	0.0802000
H	2.1389730	2.4208270	0.3899150
H	0.6688970	1.6549590	-2.0714370
H	-1.0746370	0.5536200	-1.7989340
C	-2.1278920	-0.6856870	-0.4213590
N	-2.3289580	1.9627690	0.5390820
N	-3.3234170	2.3056900	0.9451140
C	2.5913970	-0.3194780	-0.1720510
C	3.8455870	0.0380160	-0.6775550
C	4.9783180	-0.6980130	-0.3326610
C	4.8704420	-1.7965300	0.5208140
C	3.6207530	-2.1549210	1.0276580
C	2.4856930	-1.4210560	0.6855490
C	-2.1979610	-1.2812150	0.8428510
C	-3.2184910	-2.1826650	1.1338650
C	-4.1816830	-2.4864710	0.1681220
C	-4.1174120	-1.8892090	-1.0898850
C	-3.0895880	-0.9917880	-1.3850170
H	3.9305880	0.8888250	-1.3423730
H	5.9447420	-0.4138320	-0.7345330
H	5.7519910	-2.3692190	0.7871510
H	3.5270470	-3.0081220	1.6906310
H	1.5182980	-1.7056970	1.0790830
H	-1.4532020	-1.0382810	1.5923140
H	-3.2663120	-2.6455970	2.1132750
H	-4.9779930	-3.1860380	0.3973190
H	-4.8621090	-2.1220260	-1.8428220
H	-3.0356780	-0.5291550	-2.3648650

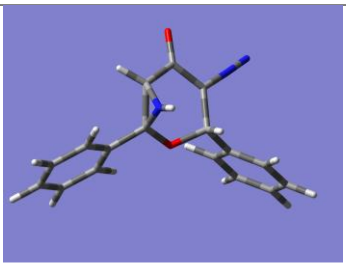

E = -970.532870, H (0K) = -970.268326,
H (298K) = -970.249918,
G (298K) = -970.316259 au.
Imaginary frequency = 1.

C	1.0151860	0.2771680	0.7048350
O	-0.2210850	-0.3489470	0.3323560
C	-1.3766290	0.4492150	0.5161890
N	-1.3806750	1.4467730	1.4576440
C	-1.2875440	1.9709460	0.1427420
C	0.0264280	2.4569410	-0.3897440
O	0.1113470	3.4632560	-1.0916550
C	1.1524190	1.5904890	-0.0668670
H	-2.1526110	2.4606760	-0.3033160
H	0.9898480	0.4971480	1.7752230
C	2.1377310	-0.6915420	0.4146160
N	2.3151580	1.9401210	-0.5459960
N	3.3057650	2.2806090	-0.9659760
C	-2.6041650	-0.3388930	0.1639420
C	-3.8323650	-0.0086960	0.7453150
C	-4.9866390	-0.7107460	0.4010190
C	-4.9245800	-1.7507200	-0.5273260
C	-3.7005050	-2.0837480	-1.1092880
C	-2.5448590	-1.3825870	-0.7674230
C	2.2604960	-1.2729970	-0.8524580
C	3.3132600	-2.1431790	-1.1242200
C	4.2572720	-2.4316270	-0.1352530
C	4.1409990	-1.8497040	1.1263140
C	3.0813740	-0.9830680	1.4008600
H	-3.8799280	0.7974220	1.4686350
H	-5.9326590	-0.4480410	0.8617680
H	-5.8219250	-2.2984630	-0.7934990
H	-3.6436350	-2.8915760	-1.8308150
H	-1.5971130	-1.6456480	-1.2195200
H	1.5303930	-1.0429510	-1.6201180
H	3.4008250	-2.5937670	-2.1067340
H	5.0784470	-3.1069700	-0.3490380
H	4.8701020	-2.0707170	1.8979520
H	2.9880830	-0.5322920	2.3833920
H	-1.4009440	1.7046700	2.4312370

Molecule ***cis*-2-oxa-7-azabicyclo[4.1.0]heptane**

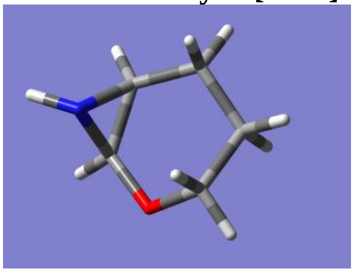

E = -325.990609, H (0K) = -325.851137,

***trans*-2-oxa-7-azabicyclo[4.1.0]heptane**

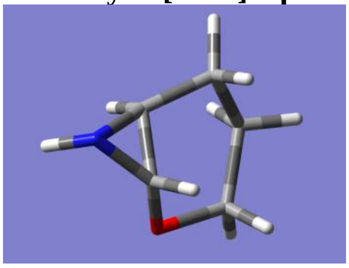

E = -325.922061, H (0K) = -325.783325,

H (298K) = -325.844379,
G (298K) = -325.880552 au.
Imaginary frequency = 0.

C	0.9212120	0.8515370	0.3167410
C	1.0122680	-0.6249100	0.4409700
C	-1.2170920	-0.8753480	-0.4058490
C	-1.5747170	0.4964140	0.1487750
C	-0.4155250	1.4917450	-0.0289850
H	1.7082640	-1.0711860	1.1428630
H	1.6012950	1.4396850	0.9249890
H	-0.9806840	-0.8156800	-1.4747730
H	-2.0342090	-1.5857420	-0.2729650
H	-2.4757620	0.8625830	-0.3517700
H	-1.8110220	0.3866280	1.2129430
H	-0.3827910	1.8501290	-1.0631120
H	-0.5756230	2.3651750	0.6088250
N	1.5480990	0.0164290	-0.7535500
H	2.5628660	0.0620470	-0.7080780
O	-0.1007390	-1.4556590	0.3032520

H (298K) = -325.776538,
G (298K) = -325.812606 au.
Imaginary frequency = 0.

C	-0.7696670	0.7147130	-0.3378310
C	-0.8187910	-0.5465680	0.3837020
C	1.3427750	-0.9298080	0.0954420
C	1.5339200	0.6043630	-0.1812510
C	0.3480070	1.5590980	0.2356960
H	-0.6033320	0.5478290	-1.3996970
H	1.5434520	-1.1313800	1.1542100
H	2.0530130	-1.4985250	-0.5050310
H	2.4588980	0.8904920	0.3288730
H	1.7052460	0.7444650	-1.2527710
H	0.2790090	1.6680760	1.3204160
H	0.4865010	2.5479060	-0.2111060
N	-2.0970520	0.0994170	0.0565140
H	-2.5149570	-0.2311720	-0.8123920
O	0.0157050	-1.5160660	-0.2070000
H	-0.6715670	-0.5158630	1.4633620

Molecule ***trans*-fused-Ph^{e}-4**

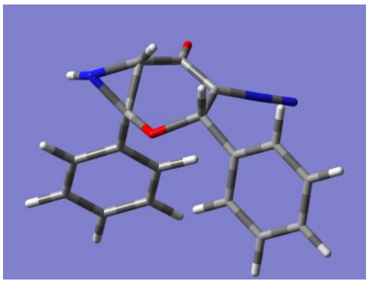

E = -970.479655, H (0K) = -970.212809,
H (298K) = -970.194572,
G (298K) = -970.258970 au.
Imaginary frequency = 0.

C	1.0017440	0.9140460	0.8519090
O	-0.0827890	0.2751170	1.6330870
C	-1.3346540	0.6973550	1.1494330
N	-2.2436460	1.6700680	1.7827320
C	-1.2716060	2.1617170	0.8076980
C	-0.8584540	2.1746910	-0.6310890
O	-1.4030790	2.4923220	-1.6675430
C	0.4866260	1.5239580	-0.4892650
H	-3.1867440	1.5309570	1.4248240
H	1.3709030	1.7392250	1.4660750
C	2.1219700	-0.0780000	0.6432400
N	1.1876660	1.3581400	-1.5732800
N	1.7648860	1.2442870	-2.5380520
C	-2.0334360	-0.3917360	0.3569330
C	-3.2392170	-0.9127010	0.8478760
C	-3.8845710	-1.9628600	0.1970630
C	-3.3354880	-2.5116290	-0.9606780
C	-2.1289340	-2.0124560	-1.4499860
C	-1.4777160	-0.9714930	-0.7912040
C	1.8683080	-1.4370880	0.4383980
C	2.9221570	-2.3150870	0.1889570
C	4.2344570	-1.8426500	0.1357840
C	4.4912200	-0.4870280	0.3424660
C	3.4388880	0.3906920	0.6005300

H	-3.6780540	-0.5121980	1.7549970	
H	-4.8131660	-2.3521040	0.5993950	
H	-3.8382150	-3.3249940	-1.4720460	
H	-1.6861360	-2.4359710	-2.3446170	
H	-0.5334990	-0.6243660	-1.1830000	
H	0.8525310	-1.8065260	0.4847300	
H	2.7174510	-3.3690460	0.0359210	
H	5.0520290	-2.5277050	-0.0599090	
H	5.5086070	-0.1133560	0.3098210	
H	3.6392620	1.4443930	0.7661110	
H	-0.4881290	2.6663330	1.3651400	

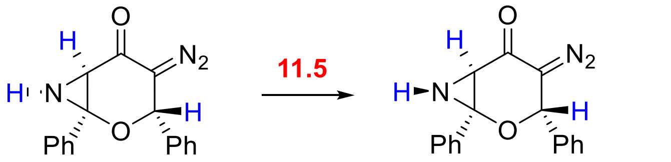

(1*RR*,3*SR*,6*RR*)-**Ph^{e}-4** (1*RR*,3*SR*,6*RR*)-**Ph^{e}-4^{inv}**

0.0 **1.4** (kcal/mol)

Figure S2: Relative Gibbs free energies of the (1*RR*,3*SR*,6*RR*)-**Ph^{e}-4** isomer and isomer with inverted azirine nitrogen (1*RR*,3*SR*,6*RR*)-**Ph^{e}-4^{inv}**, and the transition state for inversion (**TS N**-inversion (1*RR*,3*SR*,6*RR*)-**Ph^{e}-4**) (in kcal/mol, 298 K, DFT B3LYP-D3/6-311+G(d,p) level of theory with a SMD solvent model for MeCN).

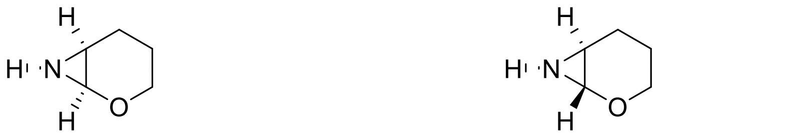

cis-2-oxa-7-azabicyclo[4.1.0]heptane *trans*-2-oxa-7-azabicyclo[4.1.0]heptane

0.0 **42.6** (kcal/mol)

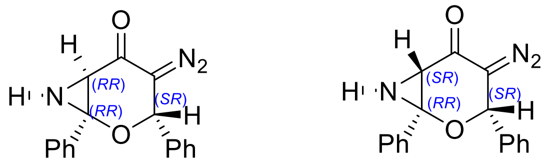

(1*RR*,3*SR*,6*RR*)-**Ph^{e}-4** *trans*-fused-**Ph^{e}-4**

0.0 **47.4** (kcal/mol)

Figure S3: Relative Gibbs free energies of *cis*-2-oxa-7-azabicyclo[4.1.0]heptane isomer/*trans*-2-oxa-7-azabicyclo[4.1.0]heptane isomer and (1*RR*,3*SR*,6*RR*)-**Ph^{e}-4** isomer/*trans*-fused-**Ph^{e}-4**, (in kcal/mol, 298 K, DFT B3LYP-D3/6-311+G(d,p) level of theory with a SMD solvent model for MeCN).

References

1. Sakharov, P. A.; Novikov, M. S.; Khlebnikov, A. F. *J. Org. Chem.* **2018**, *83*, 8304−8314. doi:10.1021/acs.joc.8b01004.
2. Bodunov V. A., Galenko E. E., Sakharov P.A., Novikov M. S., Khlebnikov A. F. *J. Org. Chem.* **2019**, *84*, 10388–10401. doi:10.1021/acs.joc.9b01573.
3. Galenko E. E., Bodunov V. A., Kryukova M. A., Novikov M. S., Khlebnikov A. F. *J. Org. Chem.* **2021**, *86*, 4098–4111. doi 10.1021/acs.joc.0c02928.
4. Zanakhov, T. O.; Galenko, E. E.; Novikov, M. S.; Khlebnikov, A. F. *J. Org. Chem.* **2022**, *87*, 15598–15607. doi 10.1021/acs.joc.2c02177.
5. Park, K. K.; Jeong, J. *Tetrahedron* **2005**, *61*, 545–553. doi:10.1016/j.tet.2004.11.022.
6. Tong, Z.; Tang, Z.; Au, C.-T.; Qiu, R. *J. Org. Chem.* **2020**, *85*, 8533–8543. doi:10.1021/acs.joc.0c00858.
7. Zanakhov, T. O.; Galenko, E. E.; Novikov, M. S.; Khlebnikov, A. F. *J. Org. Chem.* **2023**, *88*, 13191−13204. doi:10.1021/acs.joc.3c01413.
8. Dolomanov, O. V.; Bourhis, L. J.; Gildea, R. J.; Howard, J .A. K.; Puschmann, H. *J. Appl. Cryst.* **2009**, *42*, 339.
9. Sheldrick, G. M. *Acta Cryst.* **2015**, *A71*, 3–9. doi: 10.1107/S2053273314026370.
10. Sheldrick, G. M. *Acta Cryst.* **2015**, *C71*, 3-8. doi: 10.1107/S2053229614024218.
11. Gaussian 09, Revision D.01, M. J. Frisch, G. W. Trucks, H. B. Schlegel, G. E. Scuseria, M. A. Robb, J. R. Cheeseman, G. Scalmani, V. Barone, B. Mennucci, G. A. Petersson, H. Nakatsuji, M. Caricato, X. Li, H. P. Hratchian, A. F. Izmaylov, J. Bloino, G. Zheng, J. L. Sonnenberg, M. Hada, M. Ehara, K. Toyota, R. Fukuda, J. Hasegawa, M. Ishida, T. Nakajima, Y. Honda, O. Kitao, H. Nakai, T. Vreven, J. A. Montgomery, Jr., J. E. Peralta, F. Ogliaro, M. Bearpark, J. J. Heyd, E. Brothers, K. N. Kudin, V. N. Staroverov, T. Keith, R. Kobayashi, J. Normand, K. Raghavachari, A. Rendell, J. C. Burant, S. S. Iyengar, J. Tomasi, M. Cossi, N. Rega, J. M. Millam, M. Klene, J. E. Knox, J. B. Cross, V. Bakken, C. Adamo, J. Jaramillo, R. Gomperts, R. E. Stratmann, O. Yazyev, A. J. Austin, R. Cammi, C. Pomelli, J. W. Ochterski, R. L. Martin, K. Morokuma, V. G. Zakrzewski, G. A. Voth, P. Salvador, J. J. Dannenberg, S. Dapprich, A. D. Daniels, O. Farkas, J. B. Foresman, J. V. Ortiz, J. Cioslowski, and D. J. Fox, Gaussian, Inc., Wallingford CT, **2013**..
12. (a) Becke, A. D. *J. Chem. Phys.* **1993**, *98*, 5648–5652. doi: 10.1063/1.464913. (b) Becke, A. D. *Phys. Rev. A* **1988**, *38*, 3098–3100. doi: 10.1103/PhysRevA.38.3098. (c) Lee, C.; Yang, W.; Parr, R. G. *Phys. Rev. B* **1988**, *37*, 785–789. doi: 10.1103/PhysRevB.37.785.
13. (a) Grimme, S.; Antony, J.; Ehrlich, S.; Krieg, H. *J. Chem. Phys.* **2010**, *132*, 154104. doi: 10.1063/1.3382344. (b) Grimme, S.; Ehrlich, S.; Goerigk, L. *J. Comput. Chem.* **2011**, *32*, 1456–1465. doi: 10.1002/jcc.21759.
14. Marenich, A. V.; Cramer, C. J.; Truhlar, D. G. *J. Phys. Chem. B*, **2009,** *113* , 6378-6396. doi: 10.1021/jp810292n.
